# Acknowledging and Addressing Microaggressions: A Virtual Experiential Learning Approach for Faculty

**DOI:** 10.15766/mep_2374-8265.11436

**Published:** 2024-09-04

**Authors:** Jenna Essakow, April Edwell, Elisabeth Smith, Sneha Daya

**Affiliations:** 1 Assistant Professor, Department of Pediatrics, Monroe Carell Jr. Children's Hospital at Vanderbilt; 2 Assistant Adjunct Professor, Department of Pediatrics, University California, San Francisco School of Medicine; 3 Simulation Administrator, Kanbar Center, University of California, San Francisco School of Medicine; 4 Associate Professor, Departments of Internal Medicine and Pediatrics, Medstar Georgetown University Hospital

**Keywords:** Diversity, Equity, Inclusion, Communication Skills, Microaggression, Feedback, LGBTQ+, Simulation, Faculty Development

## Abstract

**Introduction:**

Although the ACGME and other accrediting organizations are increasingly emphasizing the importance of clinical learning environments that value diversity, equity, and inclusion, faculty development surrounding behavioral skills that promote inclusivity in the learning environment still needs cultivation. We designed a virtual longitudinal faculty development curriculum focused on direct observation, feedback, and practice of behavioral skills to acknowledge and address microaggressions in the learning environment.

**Methods:**

We used Kern's six steps of curriculum development to create four voluntary virtual workshops offered twice throughout the academic year, with topics including: (1) recognizing and naming microaggressions, (2) apologizing when harm has been experienced, (3) setting expectations surrounding microaggressions, and (4) debriefing microaggressions. Participant learners included residency program directors, associate program directors, and other leaders across all medical and surgical departments from one institution.

**Results:**

Thirty-one faculty from 10 departments participated in this yearlong curriculum. Pre- and postworkshop surveys analyzed participants’ self-assessments of confidence and comfort in applying learned skills. Participants were more confident in openly naming bias, delivering expectations surrounding microaggressions, and debriefing microaggressions with learners. Participants also reported greater comfort in apologizing to learners when harm has occurred in public, in person, and electronically.

**Discussion:**

To create an inclusive learning environment, faculty can increase their comfort and confidence with addressing bias and microaggressions through practice and feedback. Our curriculum demonstrates how experiential learning allows for continual practice to solidify a new skill.

## Educational Objectives

By the end of this activity, learners will be able to:

1. Acknowledge microaggressions in the learning environment.
2. Structure apologies as the source of a microaggression in the learning environment.
3. Compose expectations surrounding microaggressions with learners and teams.
4. Facilitate debriefs with learners after witnessing or experiencing a microaggression.

## Introduction

Microaggressions and bias have a clear presence in the history of medicine but are still pervasive in current clinical learning environments.^1–3^ A recent study demonstrated that the proportion of physicians who reported experiencing racial and ethnic discrimination during their medical career was substantial among racially minoritized physicians compared to those of their White peers.^4^ Similarly, in another report, residents who identified as Black, Hispanic, or Native American routinely experienced racial and ethnic bias in their work environment but rarely reported the experience. In this same report, most residency programs lacked institutional systems to promote diversity and subsequently relied on their trainees to fulfill these tasks.^5^ Specifically with regard to health professions students, microaggressions detract from academic performance and affect overall well-being, especially when unmitigated in the culture of the learning environment.^6^ The downstream effects of microaggressions, especially when underaddressed, can lead to significant learner harm, making faculty development in this topic a priority.

In review of *MedEdPORTAL* and PubMed literature, many curricula surrounding microaggressions are directed towards trainees and medical students,^2,7–10^ while others are designed to engage multiple levels of learners, from students to faculty and staff.^11,12^ Existing faculty-specific curricula focus on raising awareness of personal and systemic bias,^13–15^ but few of these provide the opportunity for faculty in positions of situational power to intentionally practice behavioral skills related to addressing microaggressions in their respective learning environments. Dedicating efforts to train these particular individuals on behavioral skills, such as acknowledging and debriefing microaggressions, can potentially improve their confidence in handling these situations, decrease stress, and improve the work and learning environment.^16–18^ To teach specific behavioral skills surrounding microaggressions, experiential learning can offer faculty leaders the opportunity to practice applying actions and words that mitigate the harms of bias and promote inclusionary practices. Simulation is one such experiential educational strategy for health professions education that can teach communication or procedural competence through direct observation and feedback. Simulation allows for deliberate practice, defined as “intentional behaviors with opportunities for guided repeat practice and self-reflection,” and thus can be adapted to teach behavioral skills that promote diversity, equity, and inclusion (DEI).^19^

We hypothesized that deliberate practice in skills related to bias would improve participants' confidence and comfort with acknowledging and addressing microaggressions and empower faculty to develop strategies that support an inclusive learning environment. We also believe that faculty role modeling of these skills will encourage trainees to do the same, facilitating a culture of respect, trust, and belonging.

## Methods

### Curriculum Development

We used Kern's six-step approach for curriculum development as the basis for this curriculum (Figure).^20,21^ After identifying our main problem, we conducted a needs assessment including informal surveys of faculty, focus groups, and analysis of past workshop results. We reviewed relevant literature on communication best practices surrounding microaggressions and reflected upon prior educational experiences of facilitators to design the content. From this, we determined that the appropriate audience for this curriculum would be faculty members who interact with trainees and medical students frequently. However, we decided that our initial pilot study would involve program directors and associate program directors of all GME programs, as they hold prominent situational power in the learning environment.

**Figure.**
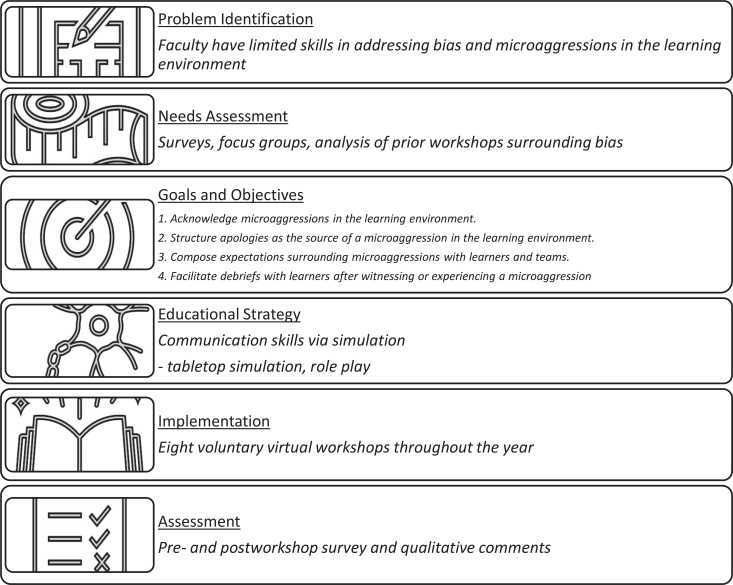
Graphic representation of the development of this curriculum using Kern's 6-step approach to curriculum development.

Based on our needs assessments, we developed goals and objectives for the curriculum. The overall goal of our curriculum was to equip faculty with comfort and confidence surrounding the skills to acknowledge and address microaggressions. We formulated specific learning objectives for each workshop topic.

In an effort to utilize experiential learning to teach these skills, we leveraged some simulation techniques. Simulation is defined as “any means of education that incorporates experiential learning in which participants learn by doing.”^22^ We primarily used tabletop simulation and role-play for the simulated scenarios. Tabletop simulation involves case-based scenarios where participants follow a hypothetical case to determine the next words or actions that they would use to address the events in the scenario. Role-playing involves participants themselves playing a specific role in a scenario with another individual in order to practice behavioral skills that they would use in a real-life scenario with the potential to start, stop, or pause the scenario at any moment. We chose these modalities as they are easy ways to practice communication in a virtual space.

### Curricular Implementation

We implemented the curriculum using a virtual platform. The decision to make this curriculum voluntary was intentional in order to maintain an environment where participants felt ready to engage in the learning material. Participants chose which sessions to attend based on their personal expertise in order to highlight that every individual is in a different place in their lived experience and their DEI knowledge and skills.

### Funding

The authors of this curriculum applied for and were awarded an institutional educational grant. Funding was used to pay for staff support, time and training for simulation staff, and faculty time for the development of this project.

### Equipment/Environment

Per participant preference, the workshops were conducted over Zoom, a cloud-based video communication application that allows for virtual video and audio conferencing as well as screen-sharing for PowerPoint presentations and videos.

### Personnel

#### Facilitators

The authors, who developed the cases, served as facilitators of each workshop in order to maintain continuity throughout the curriculum. At least one facilitator is needed to lead each workshop. The ideal facilitator for this curriculum should have knowledge, experience, and comfort with DEI content and should review all the information presented in the facilitator guides prior to leading a workshop. In addition, they should have adequate skills in facilitating small-group learning, delivering formative feedback, and debriefing specific DEI topics. Our facilitators had prior experience with curriculum development in DEI topics and facilitation with health care professions learners. Two facilitators had received formal training in trauma-informed care and de-escalation techniques. Facilitators who have not had as much prior experience in DEI training can be encouraged to take courses related to medical education and inclusive learning environments prior to engaging in this work. Before each workshop, facilitators conducted prebriefs to discuss potential questions and topics that may arise during the workshop. After each workshop, facilitators debriefed and reflected on what elements went well and what could be changed to improve the session. Because this curriculum was developed virtually, cofacilitation with faculty at other sites or institutions was feasible. We intended for cross-collaboration and cofacilitation to provide a broad array of facilitator backgrounds for this curriculum.

#### Participants

All GME residency and fellowship program directors and associate program directors were invited to participate in the workshops. Invitations were extended via email. No prerequisite knowledge or experience was required prior to participation, but advertisements for each workshop described the intended audience for each workshop (Appendix A).

### Implementation

For each of the four workshops, a facilitator guide, PowerPoint, participant skills handout, and pre- and postsurveys were created. We planned for 10–15 participants per workshop. Each workshop began with a review of community agreements, which set forth operational behaviors that each participant acknowledges and voluntarily agrees to during the educational sessions in order to build trust and community. Each workshop generally included topic-related ice breakers, review of critical definitions, didactic content and principles, interactive experiential exercises, and large-group debriefing. Workshop length varied from 2–3 hours. Each of the four workshops included time for a facilitated experiential activity in small groups: (1) 45 minutes, (2) 25 minutes, (3) 30 minutes, and (4) 50 minutes, respectively. Three facilitators presented key content and were present for small-group activities with participants.

All facilitator guides, participant skills handouts, PowerPoint presentations, and pre- and postsurveys for the workshops can be found in the appendices (Appendices B–Q). Table 1 describes the goals and objectives, interactive exercises, and simulation modality used for each workshop.

**Table 1.**
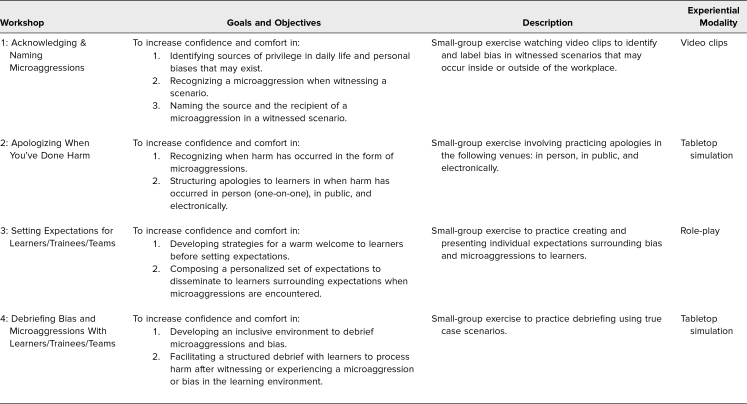
Description of Each Workshop and Corresponding Experiential Content and Modality

### Debriefing

Facilitator guides contained debriefing questions for learners to begin intentional discussions surrounding these subjects. In each of the workshops, facilitators had a presence in small-group work to help stimulate active communication, reflective pauses, and group brainstorming for challenging moments. Because facilitators had the unique role of also offering feedback to participants, the facilitators remained the same for the entire curriculum to develop rapport, continuity, and relational trust.

### Assessment

We utilized the first level of Kirkpatrick's four levels of training evaluation to structure our evaluation plan.^20^ We developed pre- and postworkshop surveys based on the educational objectives. Each survey assessed participants’ confidence and comfort with the specific skills developed during the workshops. We used confidence and comfort as a means of assessing self-efficacy, as we anticipated that improved self-efficacy with these skills would promote real-life practice in this work.^23^ Each survey included demographic questions, questions with a 5-point Likert scale (1 = *strongly disagree*, 5 = *strongly agree*), and free-text opportunities for feedback and commentary.

We distributed the surveys using Qualtrics. Participants were given allotted time at the beginning and end of each workshop to complete the pre- and postsurveys. Each participant was assigned a unique identifier that allowed for confidentiality but gave the authors the ability to match pre- and postsurveys for each workshop as well as longitudinally throughout the curriculum. We averaged objective scores and calculated standard deviations. The University of California, San Francisco Institutional Review Board reviewed and exempted the project from further review (IRB 20-30397).

## Results

### Demographics of Participants

We conducted eight workshops (all four topics presented twice) between August 2021 and June 2022, consisting of four to 10 participants for each workshop. A total of 31 faculty from 10 different departments participated.

Eight participants attended one other workshop and four participants attended greater than two other workshops. No participant attended every single workshop. In total, 80% of participants identified as female. The predominant race represented by participant self-identification were White (66% or greater) followed by Asian (15–25%) in each workshop.

When asked what identities participants resonated with, we received free-text responses such as: “cisgender,” “Jewish,” “first generation,” “immigrant,” “bilingual,” “bicultural,” “tricultural,” “sexual minority community member,” “mother,” “bisexual woman,” and “LGBTQ ally.” This free-text space gave participants the opportunity to share the other identities and lenses that were important to them when engaging in this work.

For each workshop, completion rates of pre- and postsurveys were upwards of 80% of participants. Mean scores of confidence and comfort surrounding specific behavioral skills after participation in the workshop increased after each of the workshops. Completed survey results as well as participant feedback from each workshop are summarized in Tables 2 and 3.

**Table 2.**
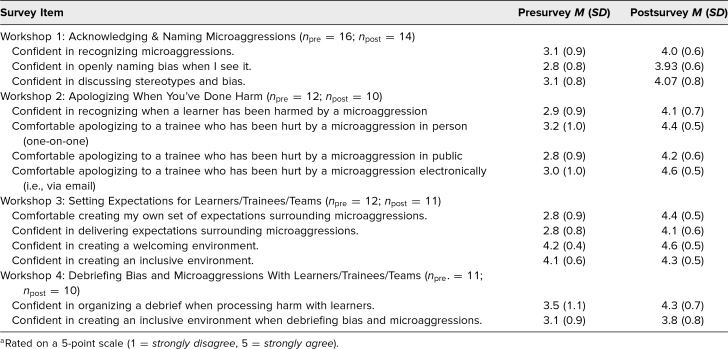
Participant Rating of Workshop Objectives on Pre- and Postsurveys^a^

**Table 3.**
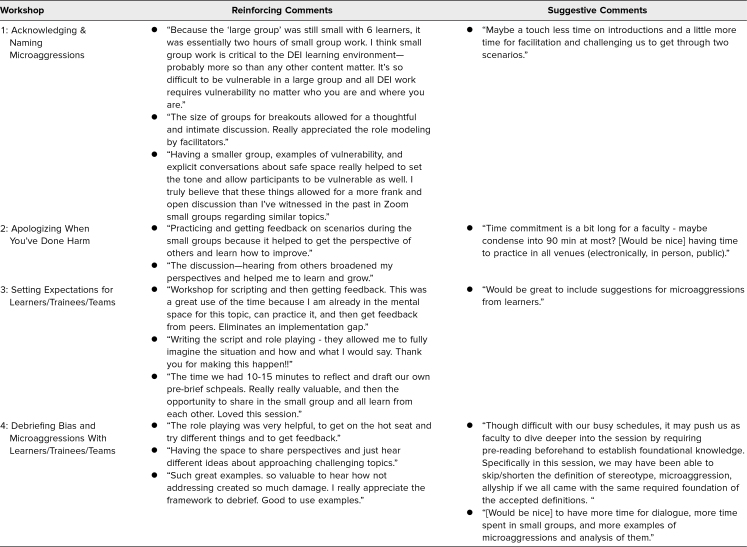
Participant Postsurvey Workshop Feedback

The most noticeable change in pre- and postsurvey scores occurred with the workshop topic surrounding apologizing to learners. After the workshop, eight of 10 participants of this workshop answered *agree* or *strongly agree* when asked if they felt confident in their ability to recognize when a learner has been harmed by a microaggression in the clinical learning environment. All 10 of the postsurvey respondents selected *agree* or *strongly agree* when asked if they felt comfortable apologizing to a trainee who had been hurt by a microaggression in person (one-on-one), nine of 10 (90%) respondents selected *agree* or *strongly agree* when asked if they felt comfortable apologizing to a trainee in public, and all 10 postsurvey respondents selected *agree* or *strongly agree* when asked if they felt comfortable apologizing to a trainee electronically.

## Discussion

This work adds to the literature by presenting a novel experiential curriculum to help faculty leaders create an inclusive learning environment that values DEI by acknowledging and addressing bias in the form of microaggressions. We were able to execute a longitudinal curriculum that goes beyond recognizing bias and moves toward definitive actions that can promote change. In the process of creating this curriculum, we learned several lessons.

Early in the development of this curriculum, we realized that there was a shift in participant preference for educational venues. Although we instinctively thought that forming the sense of belonging and community needed for this this type of learning would occur best in person, most participants preferred virtual workshops. We adjusted the workshop formats for a virtual setting by allowing more discussion time, pauses, and small-group activity time. Virtual delivery made continuity among facilitators important so that skills practiced in this setting could be debriefed with familiarity at each workshop session. Community agreements set forth at each workshop were useful for guidelines surrounding operational and relational behaviors in the virtual learning environment.

We also learned the importance of facilitator selection and preparation. Challenging questions, situations, and discussions took place during each workshop, and multiple facilitators with varying skill sets can partner with each other to help navigate these conversations. In addition to co-facilitation and cross-collaboration that can help nurture facilitation skills, it is helpful for facilitators to engage in relationship-centered communication training, trauma-informed care training, and de-escalation training. Rehearsing workshop facilitation with contingency plans in a facilitator prebriefing prior to each workshop helped prepare facilitators. Importantly, facilitators in our curriculum consistently debriefed amongst themselves after each workshop to reflect upon successes, challenges, and opportunities for improvement.

Regarding topic selection, we decided to start this curriculum with foundational topics, such as acknowledging and naming bias and microaggressions, and gradually moved towards more complex skills such as debriefing microaggressions. We suggest this iterative format so that faculty members who have not had significant exposure to bias awareness can work on this before engaging in other behavioral skills. We found that one of the most important workshops dealt with apologizing when a learner has experienced harm, a skill that no participant had ever formally learned in an anti-bias training. This might be a workshop that can benefit every faculty member who interacts with learners. The work involved with setting expectations and debriefing bias and microaggressions in the learning environment involves longer skills lessons with practice time. Being explicit about the intended audience for each workshop can be helpful for learners to gain the most from each workshop.

In terms of implementation, we learned that small-group learning was the best way to practice these DEI-related skills. Participants had the opportunity to rehearse exact words they would use in a space where they could make mistakes, obtain facilitator feedback, and have opportunities to try again. This rehearsal and repetition becomes important for developing confidence and comfort with using these skills in reality. Based on survey results as well as feedback, participants found the workshops beneficial in helping gain comfort and confidence surrounding skills of acknowledging and addressing microaggressions. Prior literature has found a strong correlation between self-efficacy and work-related performance.^24^ More recently, studies related to DEI show a positive relationship between diversity self-efficacy beliefs and desirable outcomes from diversity training.^25^ We hope that improvement in participants’ own comfort and confidence in the skills learned and acquired will translate to utilization of these skills in real-life scenarios.

### Limitations

There are many limitations to this curriculum. Workshop topics are important to consider as different institutions have varying needs in terms of DEI-related behavioral skills. We chose these universal topics, but others may stand out when conceptualizing a similar simulation curriculum at a different health care institution.

Similarly, funding is not equal across all national institutions. Our facilitators had specific time and funding dedicated to producing and executing this curriculum, which might not be the case for other course leaders. We recommend applying for grants and scholarships from both national organizations and one's own institution. For participants, we offered maintenance of certification credits, which can be applied at other institutions.

In terms of feasibility, participants of this curriculum came from varying clinical sites throughout the institution, making in-person workshops a challenge. Our participants preferred learning in a virtual space, but this curriculum can be easily adapted to in-person learning in order to foster community building. Regarding timing and duration of each workshop, we understand that 2–3 hours of workshop time is a large time commitment for busy faculty. Each of these workshops can be separated into two sessions to better adhere to scheduling constraints. Workshops can also be adjusted by offering prework for some of the material (definition review, video viewing) before the start of the session.

We acknowledge that most of the small number of participants in this curriculum came from a homogenous self-identified social category (predominantly White females). We advertised broadly and repeatedly via email, but in order to recruit more participants, there could be an added benefit from sending personalized messages to departmental leadership. Advertising on social media, physical bulletins in workspaces, newsletters, and via text message could be helpful in reaching broader audiences as well. The decision to not make this curriculum mandatory does not equate to lack of prioritization by the institution. The voluntary nature of this curriculum gave participants the opportunity to deeply reflect on where they stand in their understanding of the various topics so that they can gain the most skills from the workshops that they chose to attend. However, it is reasonable to assume that those who attended workshops might be more invested in the curriculum and dedicated to learning the skills, resulting in more positive feedback in surveys. If the curriculum became mandatory, we might not receive the same positive feedback.

Survey results are filled out by participants and thus are subject to bias. Having a more objective way to measure outcomes would improve the accountability of the results. Although we believe that self-efficacy directly correlates to real-life practice of these critical skills, the limit of this assessment is the lack of measurement of the impact on the learning environment. In the future, assessing the impact of these behavioral changes on learners will be important to determine whether this curriculum has the intended effect. To examine retention of these skills for faculty, follow-up interval surveys about confidence and utilization of these skills might help measure sustained behavior change.

### Next Steps

We initially envisioned that leadership of all residency and fellowship programs across the university would partake in this training with the goal of eventually expanding this curriculum to faculty from all professional schools on campus. We want to work towards training entire departments within institutions, which could be done in the form of faculty retreats or monthly educational sessions.

While we start with skills in acknowledging and addressing microaggressions, future iterations of this curriculum will include additional skills to for cultivating inclusive learning environments. We have already initiated standardized patient training for future workshops where participants will be able to practice addressing microaggressions and utilizing inclusionary behaviors, such as microaffirmations, to help shape positivity and belonging in the learning environment.^26^ We also have considered tailoring this training to an interprofessional space to learn how health care team members can address microaggressions at the bedside while still developing trusting relationships with patients.

As we continue to develop more curricula surrounding communication and change in behavioral skills that promote DEI, we hope that we can find avenues to create belonging in our learning environments so that all our learners can thrive.

## Appendices


Sample Flier.pptxWorkshop 1 - Slides.pptxWorkshop 1 - Facilitator GuideWorkshop 1 - Participant Handout.docxWorkshop 1 - Pre- and Postsurvey.docxWorkshop 2 - Slides.pptxWorkshop 2 - Facilitator Guide.docxWorkshop 2 - Participant Handout.docxWorkshop 2 - Pre- and Postsurvey.docxWorkshop 3 - Slides.pptxWorkshop 3 - Facilitator Guide.docxWorkshop 3 - Participant Handout.docxWorkshop 3 - Pre- and Postsurvey.docxWorkshop 4 - Slides.pptxWorkshop 4 - Facilitator Guide.docxWorkshop 4 - Participant Handout.docxWorkshop 4 - Pre- and Postsurvey.docx
*All appendices are peer reviewed as integral parts of the Original Publication.*
