# Supplementary material for: Acknowledging and Addressing Microaggressions: A Virtual Experiential Learning Approach for Faculty
Source: MedEdPORTAL. 2024 Sep 4;20:11436. doi: 10.15766/mep_2374-8265.11436 (PMC11374130; doi:10.15766/mep_2374-8265.11436)
Supplement: Supplementary file 1 — Sample Flier.pptxWorkshop 1 - Slides.pptxWorkshop 1 - Facilitator GuideWorkshop 1 - Participant Handout.docxWorkshop 1 - Pre- and Postsurvey.docxWorkshop 2 - Slides.pptxWorkshop 2 - Facilitator Guide.docxWorkshop 2 - Participant Handout.docxWorkshop 2 - Pre- and Postsurvey.docxWorkshop 3 - Slides.pptxWorkshop 3 - Facilitator Guide.docxWorkshop 3 - Participant Handout.docxWorkshop 3 - Pre- and Postsurvey.docxWorkshop 4 - Slides.pptxWorkshop 4 - Facilitator Guide.docxWorkshop 4 - Participant Handout.docxWorkshop 4 - Pre- and Postsurvey.docx [file mep_2374-8265.11436-s001.zip › A. Sample Flier.pptx]

## Slide 1
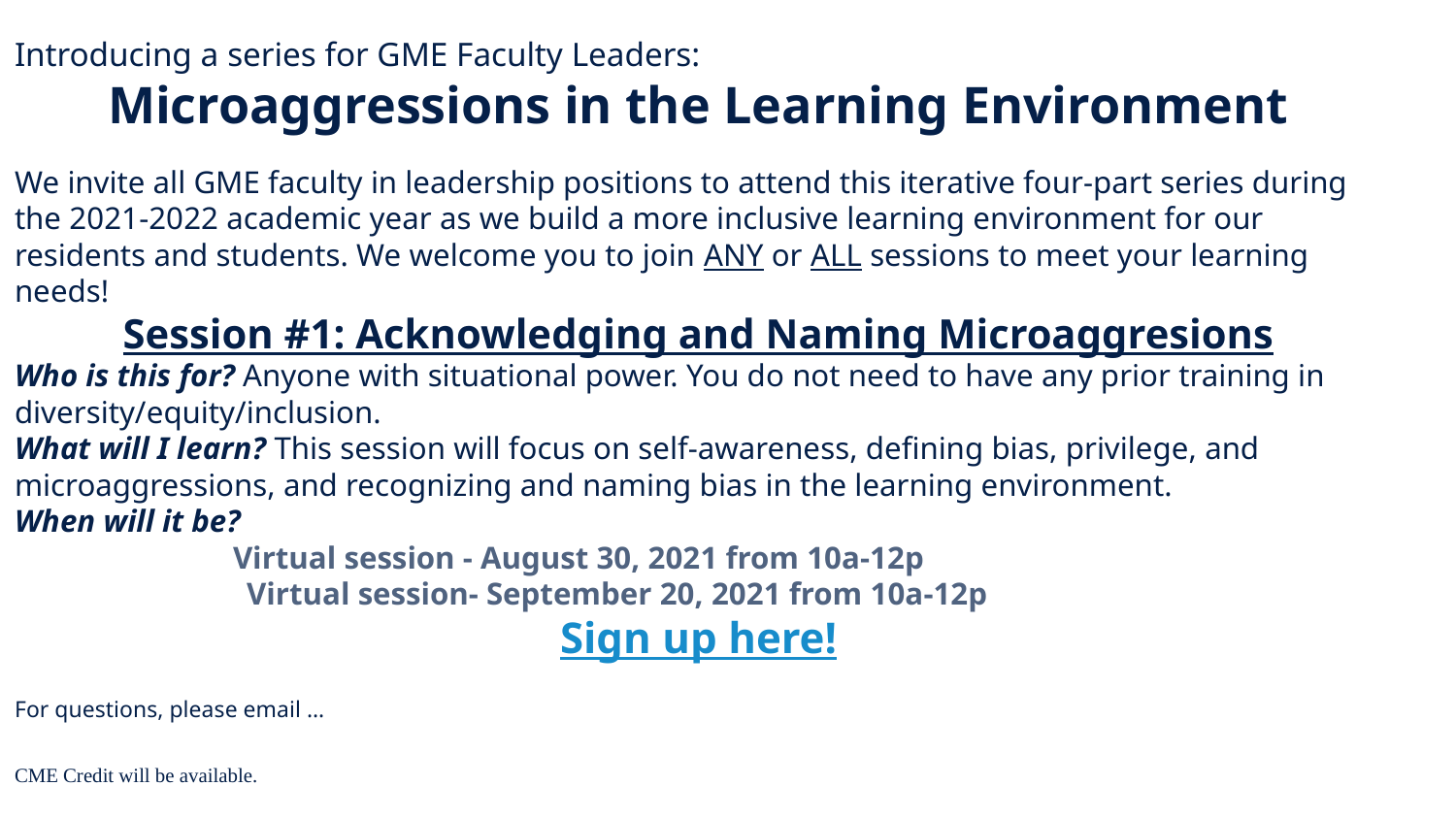

Introducing a series for GME Faculty Leaders:
Microaggressions in the Learning Environment
We invite all GME faculty in leadership positions to attend this iterative four-part series during the 2021-2022 academic year as we build a more inclusive learning environment for our residents and students. We welcome you to join ANY or ALL sessions to meet your learning needs!
Session #1: Acknowledging and Naming Microaggresions
Who is this for? Anyone with situational power. You do not need to have any prior training in diversity/equity/inclusion.
What will I learn? This session will focus on self-awareness, defining bias, privilege, and microaggressions, and recognizing and naming bias in the learning environment.
When will it be?
Virtual session - August 30, 2021 from 10a-12p
 Virtual session- September 20, 2021 from 10a-12p
Sign up here!
For questions, please email …
CME Credit will be available.
