# Supplementary material for: Acknowledging and Addressing Microaggressions: A Virtual Experiential Learning Approach for Faculty
Source: MedEdPORTAL. 2024 Sep 4;20:11436. doi: 10.15766/mep_2374-8265.11436 (PMC11374130; doi:10.15766/mep_2374-8265.11436)
Supplement: Supplementary file 1 — Sample Flier.pptxWorkshop 1 - Slides.pptxWorkshop 1 - Facilitator GuideWorkshop 1 - Participant Handout.docxWorkshop 1 - Pre- and Postsurvey.docxWorkshop 2 - Slides.pptxWorkshop 2 - Facilitator Guide.docxWorkshop 2 - Participant Handout.docxWorkshop 2 - Pre- and Postsurvey.docxWorkshop 3 - Slides.pptxWorkshop 3 - Facilitator Guide.docxWorkshop 3 - Participant Handout.docxWorkshop 3 - Pre- and Postsurvey.docxWorkshop 4 - Slides.pptxWorkshop 4 - Facilitator Guide.docxWorkshop 4 - Participant Handout.docxWorkshop 4 - Pre- and Postsurvey.docx [file mep_2374-8265.11436-s001.zip › B. Workshop 1 - Slides.pptx]

## Slide 1
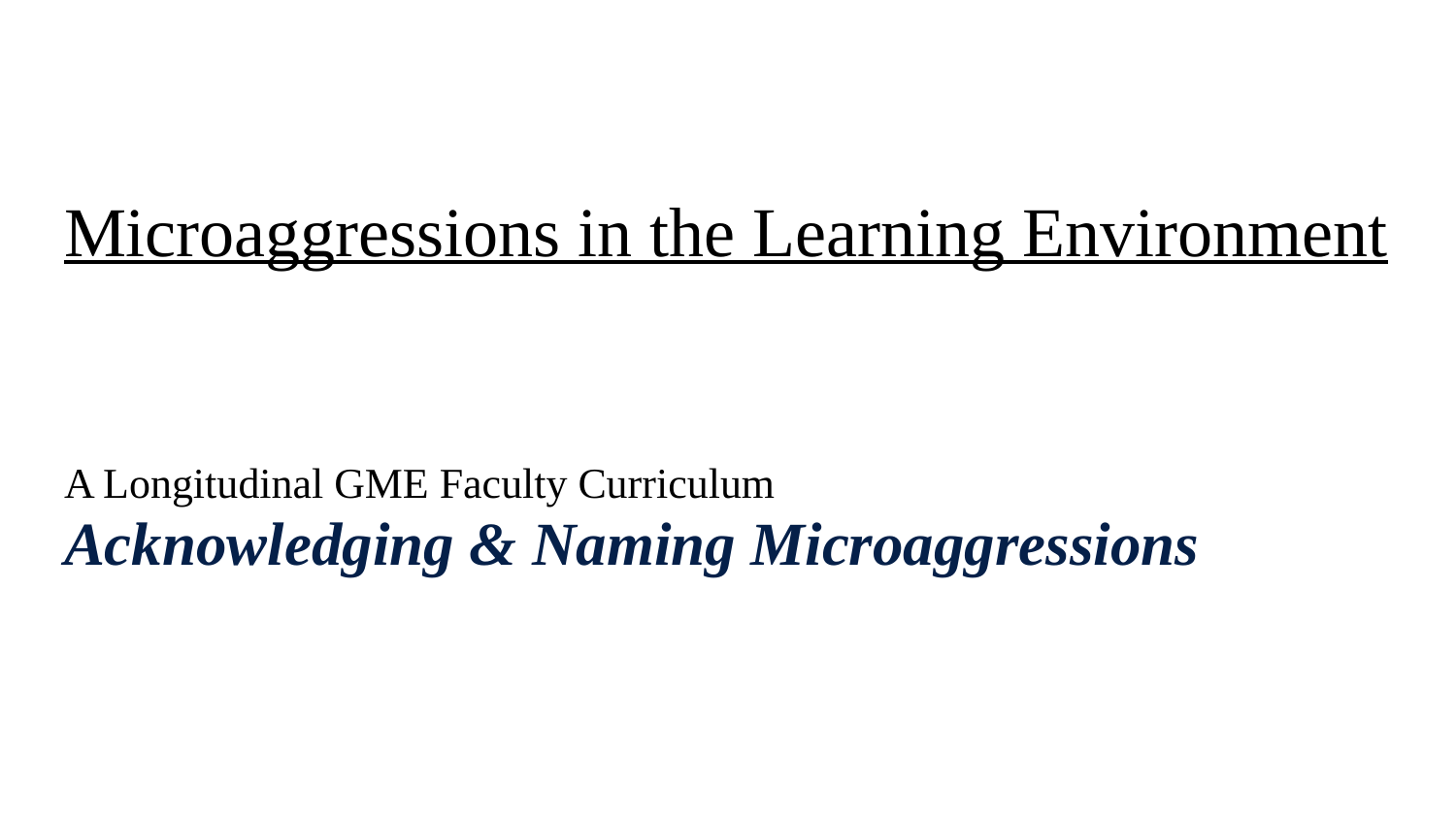

# Microaggressions in the Learning Environment
A Longitudinal GME Faculty Curriculum
Acknowledging & Naming Microaggressions

## Slide 2
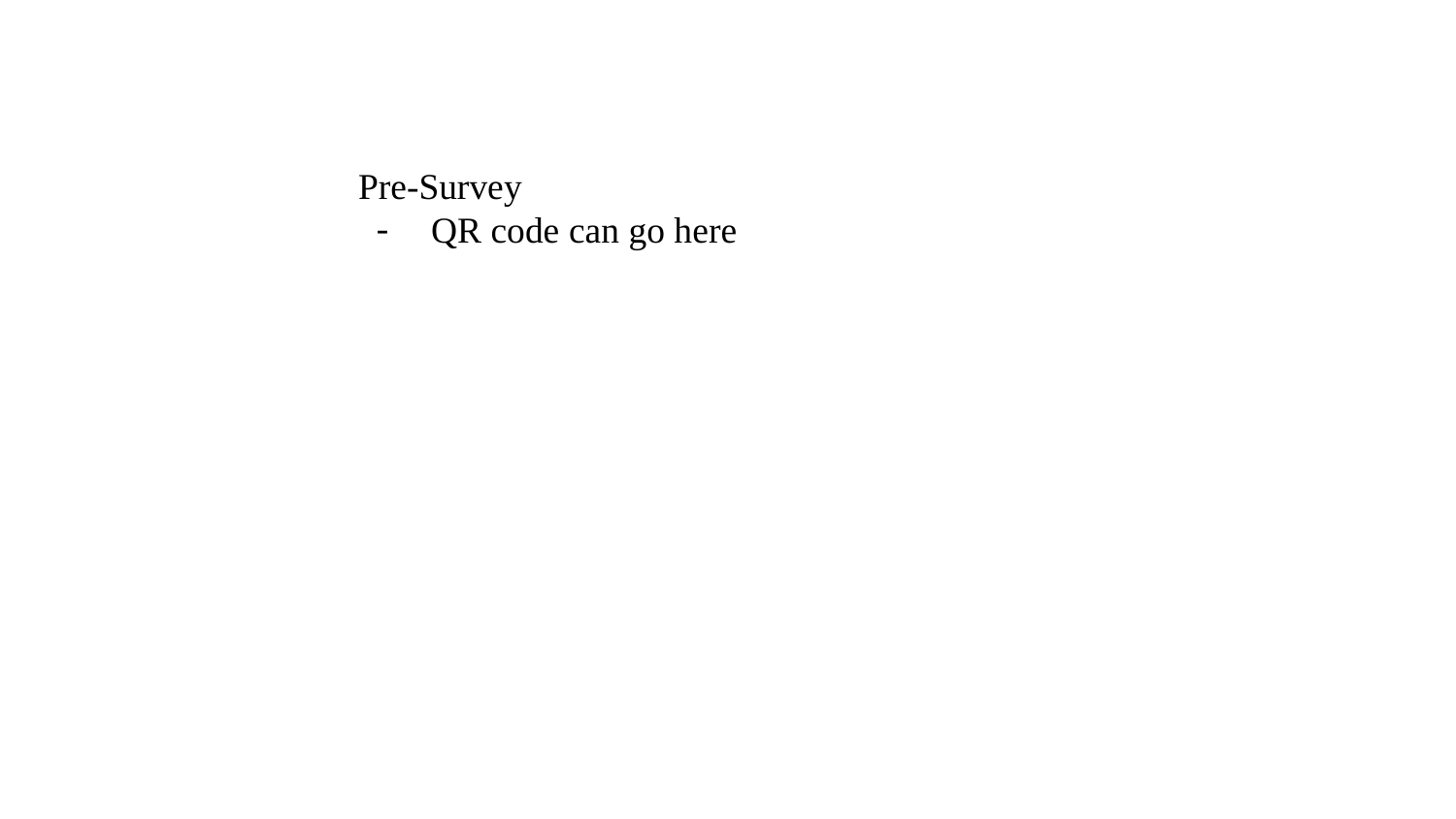

Pre-Survey
QR code can go here

## Slide 3
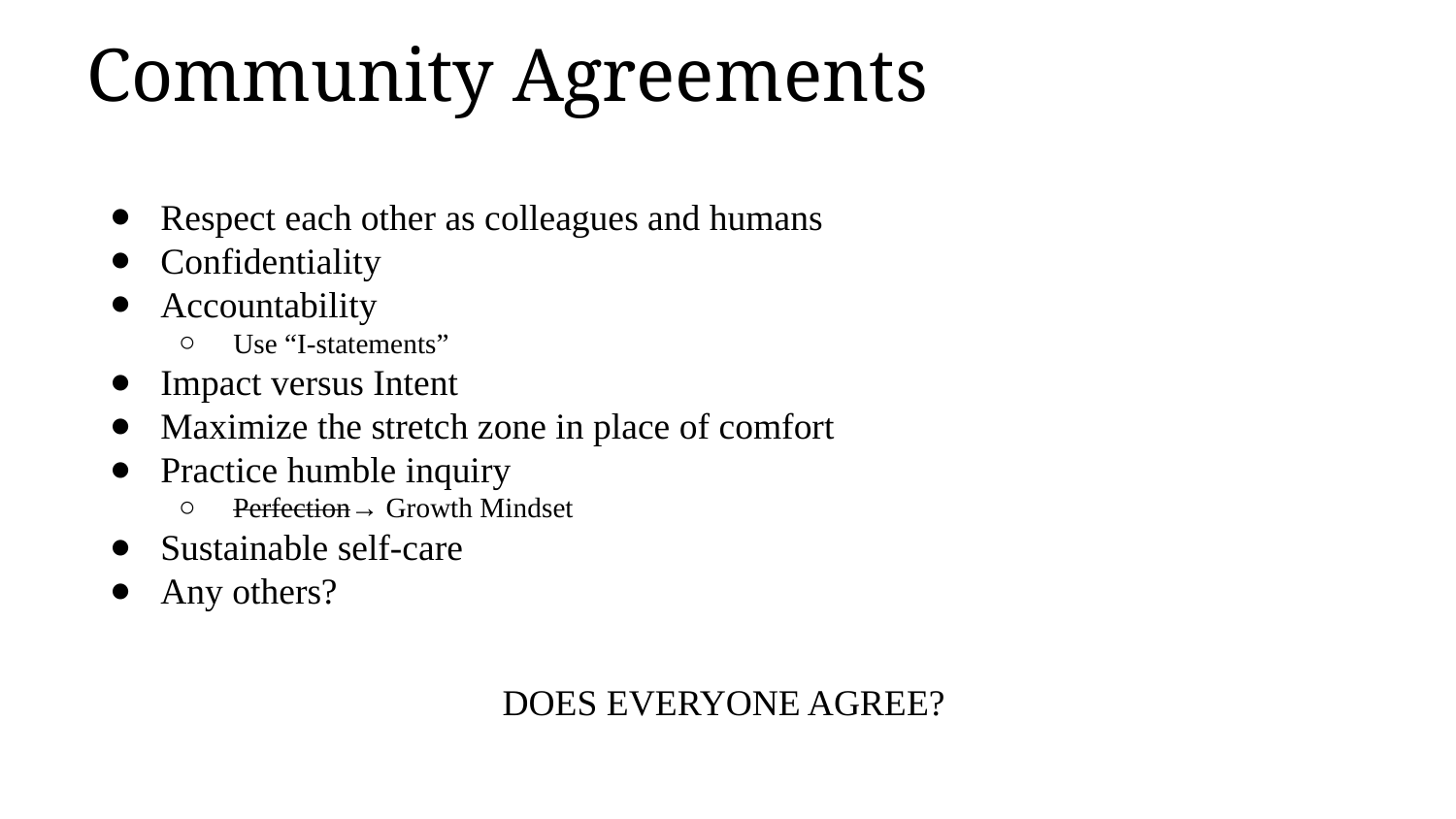

# Community Agreements
Respect each other as colleagues and humans
Confidentiality
Accountability
Use “I-statements”
Impact versus Intent
Maximize the stretch zone in place of comfort
Practice humble inquiry
Perfection→ Growth Mindset
Sustainable self-care
Any others?
DOES EVERYONE AGREE?

## Slide 4
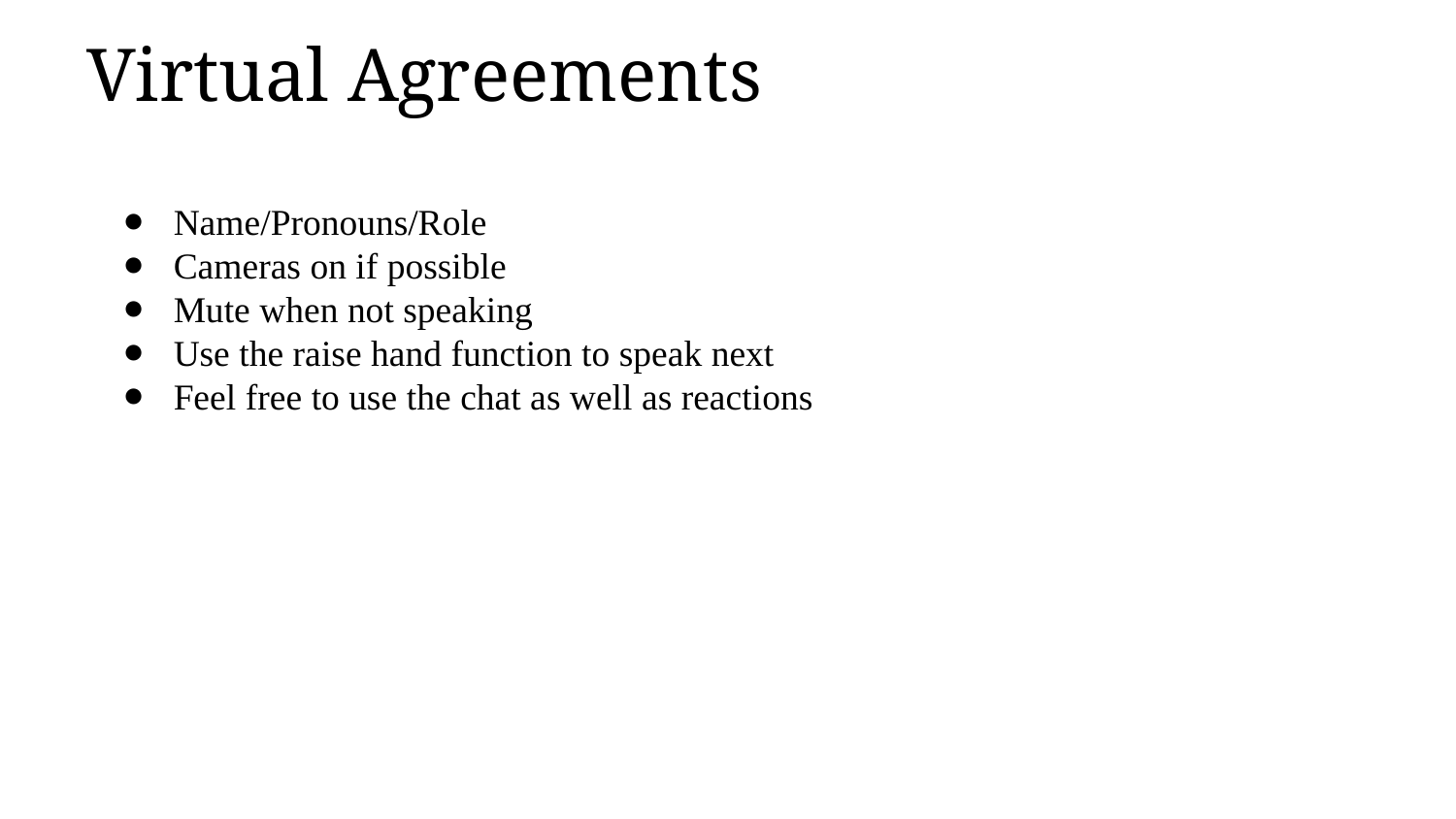

# Virtual Agreements
Name/Pronouns/Role
Cameras on if possible
Mute when not speaking
Use the raise hand function to speak next
Feel free to use the chat as well as reactions

## Slide 5
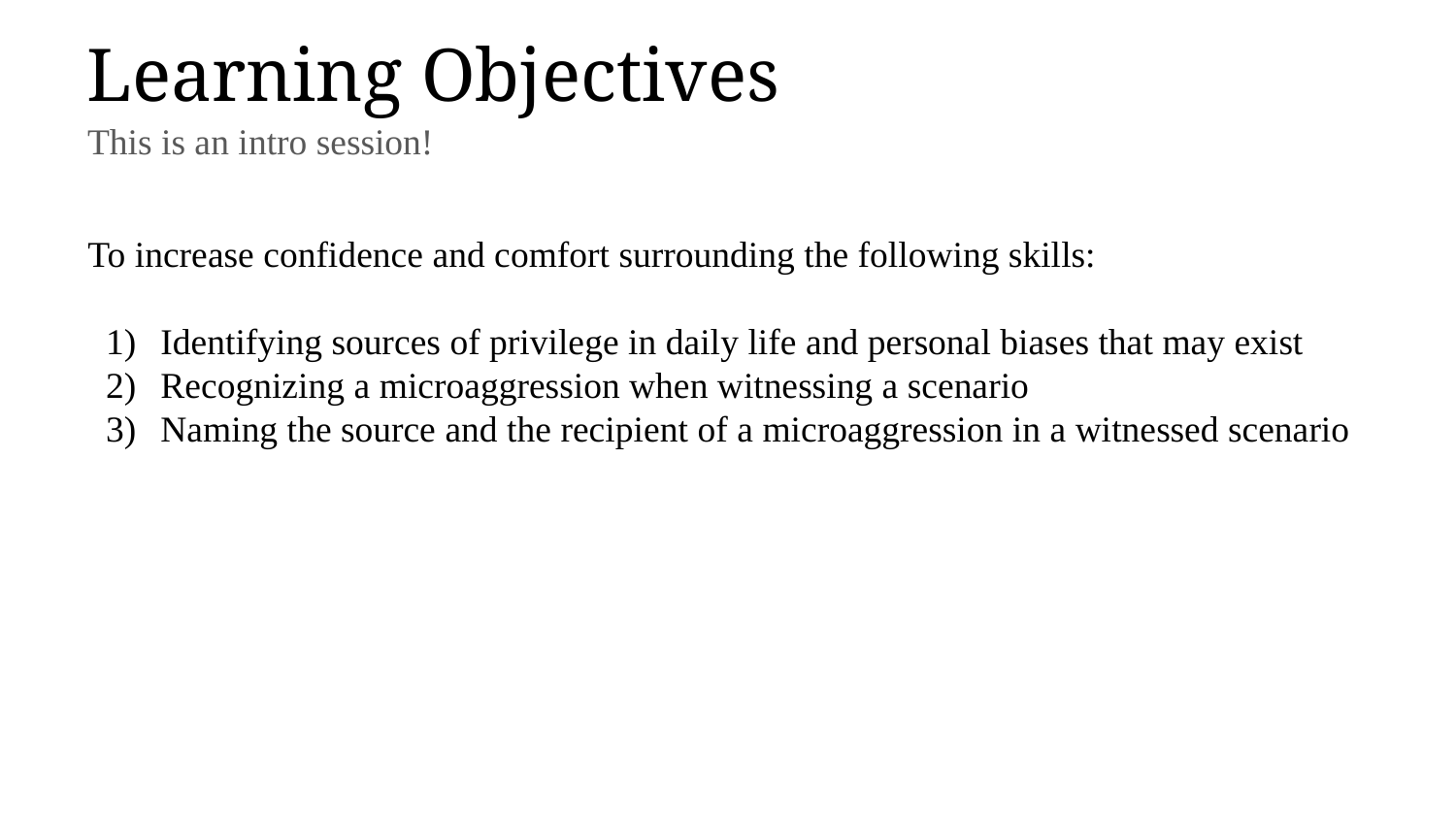

# Learning Objectives
This is an intro session!
To increase confidence and comfort surrounding the following skills:
Identifying sources of privilege in daily life and personal biases that may exist
Recognizing a microaggression when witnessing a scenario
Naming the source and the recipient of a microaggression in a witnessed scenario

## Slide 6
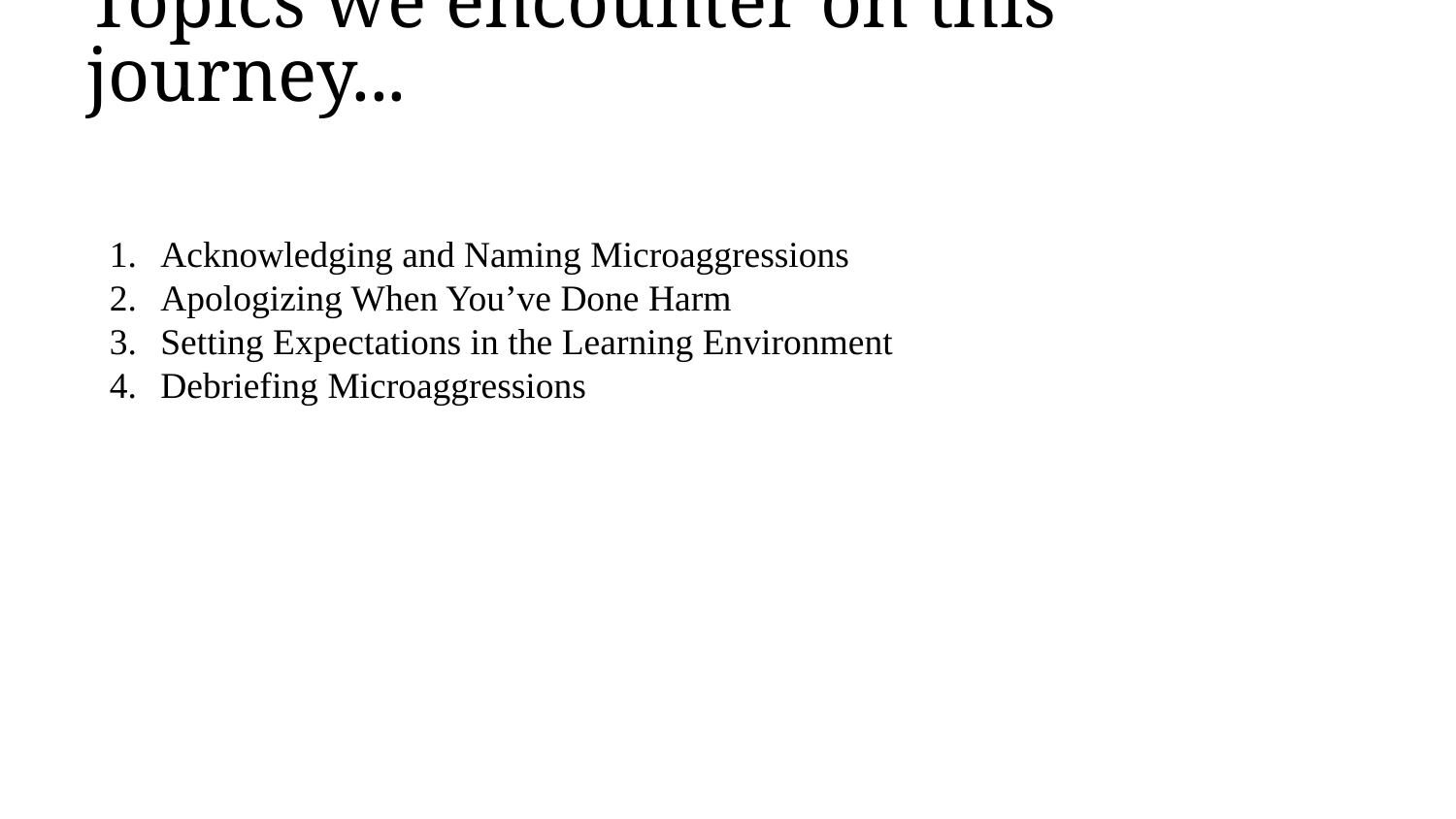

# Topics we encounter on this journey...
Acknowledging and Naming Microaggressions
Apologizing When You’ve Done Harm
Setting Expectations in the Learning Environment
Debriefing Microaggressions

## Slide 7
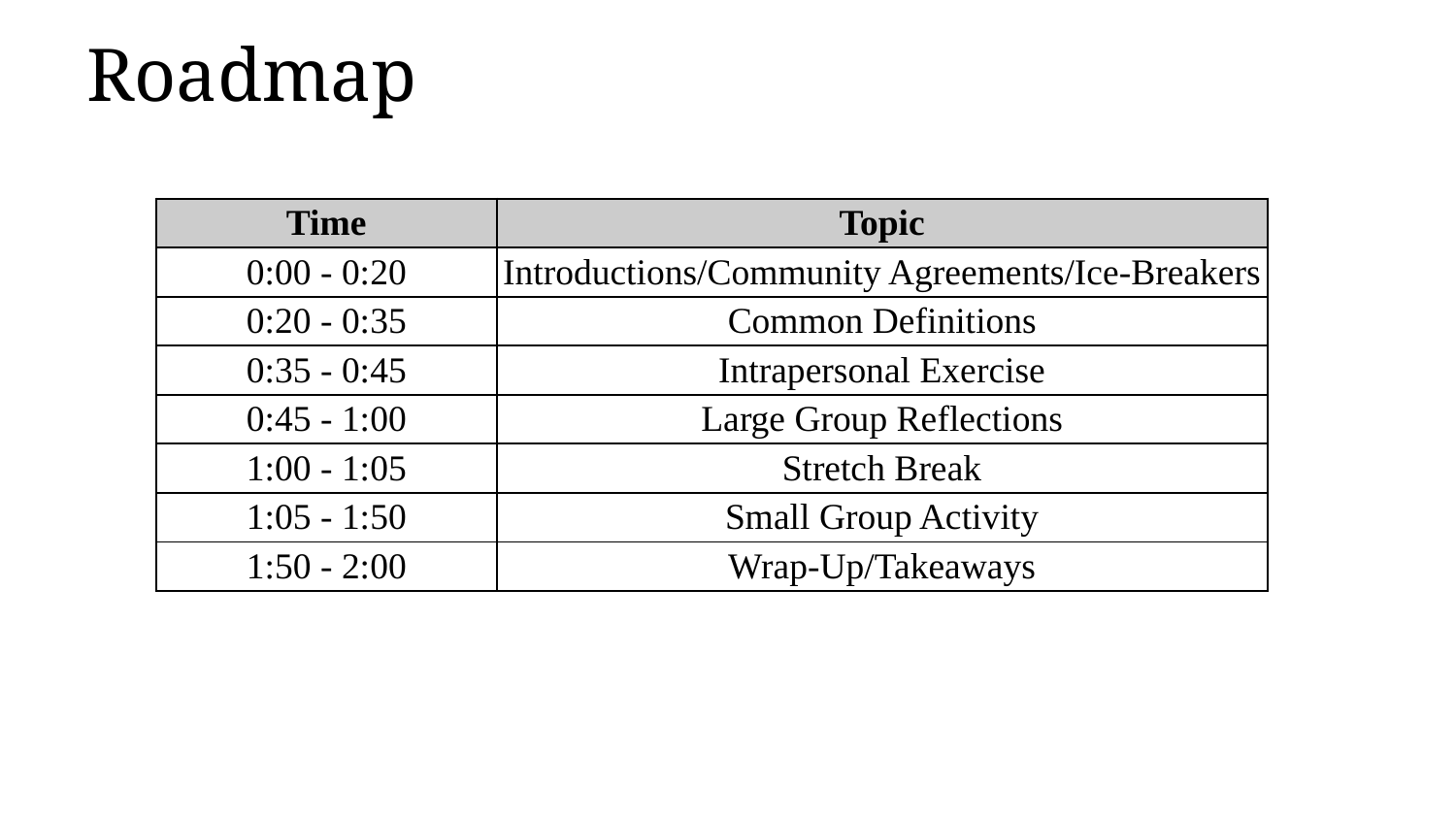

# Roadmap
| Time | Topic |
| --- | --- |
| 0:00 - 0:20 | Introductions/Community Agreements/Ice-Breakers |
| 0:20 - 0:35 | Common Definitions |
| 0:35 - 0:45 | Intrapersonal Exercise |
| 0:45 - 1:00 | Large Group Reflections |
| 1:00 - 1:05 | Stretch Break |
| 1:05 - 1:50 | Small Group Activity |
| 1:50 - 2:00 | Wrap-Up/Takeaways |

## Slide 8
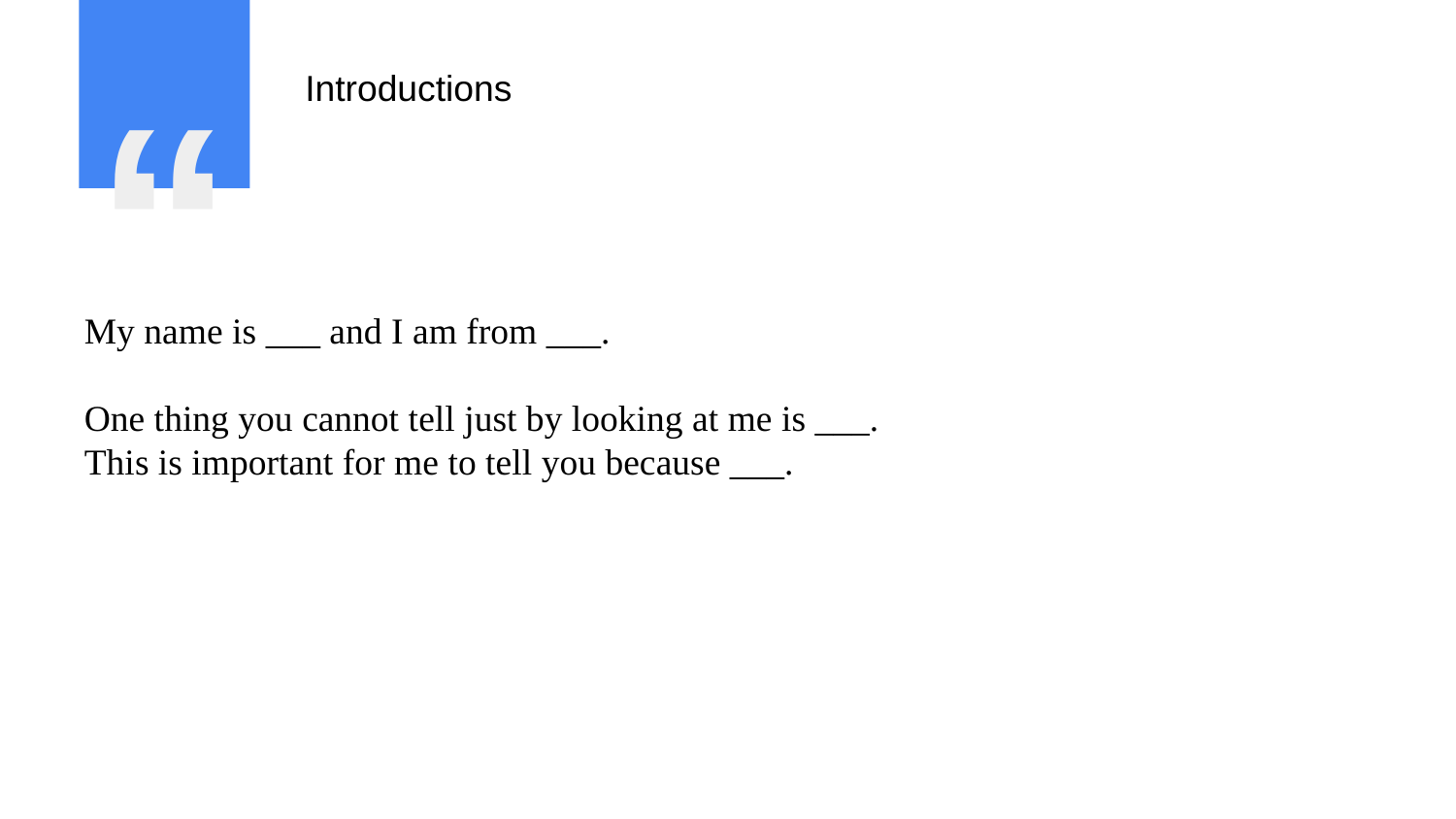

Introductions
My name is ___ and I am from ___.
One thing you cannot tell just by looking at me is ___.
This is important for me to tell you because ___.

## Slide 9
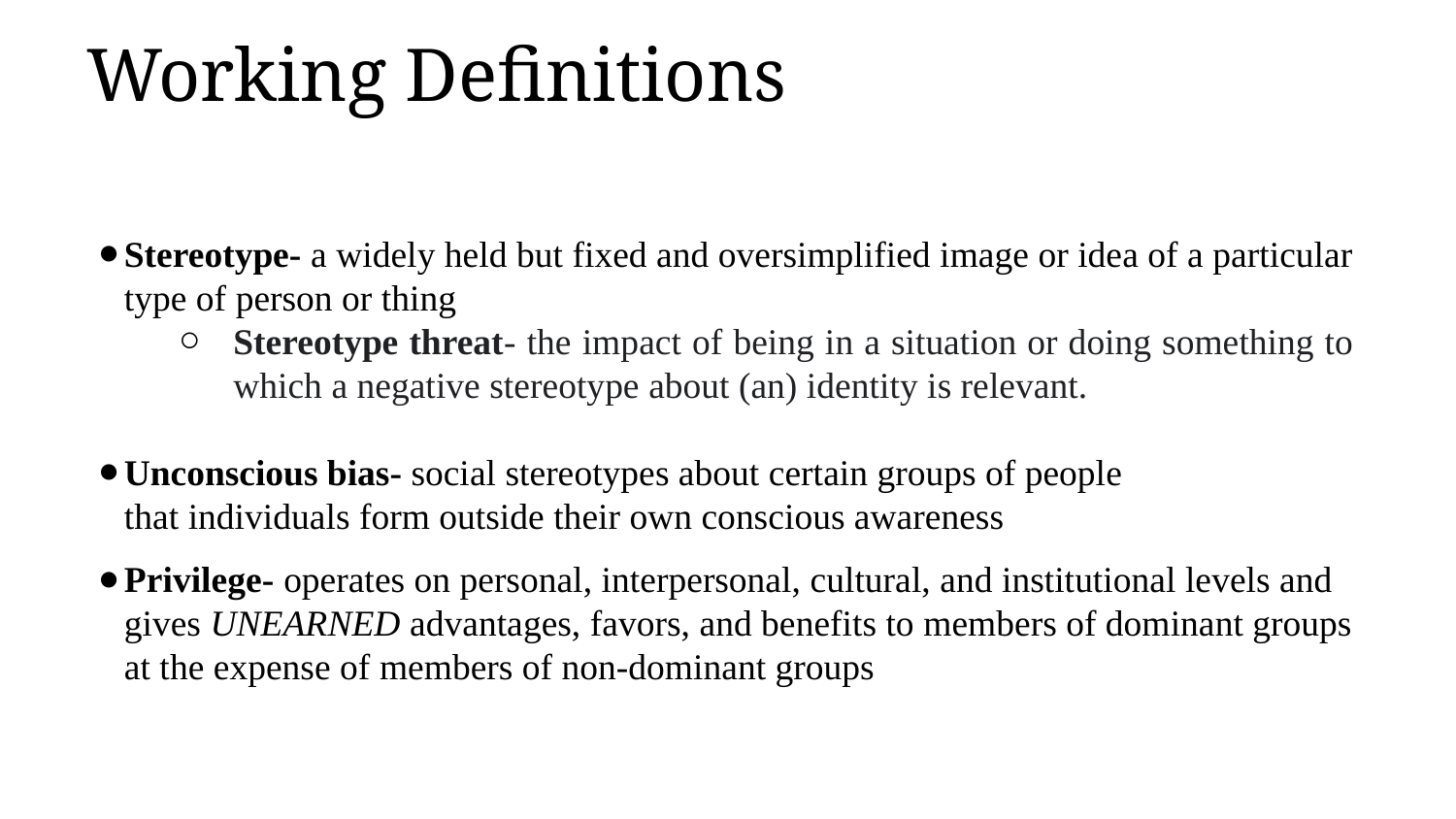

# Working Definitions
Stereotype- a widely held but fixed and oversimplified image or idea of a particular type of person or thing
Stereotype threat- the impact of being in a situation or doing something to which a negative stereotype about (an) identity is relevant.
Unconscious bias- social stereotypes about certain groups of people that individuals form outside their own conscious awareness​
Privilege- operates on personal, interpersonal, cultural, and institutional levels and gives UNEARNED advantages, favors, and benefits to members of dominant groups at the expense of members of non-dominant groups​

## Slide 10
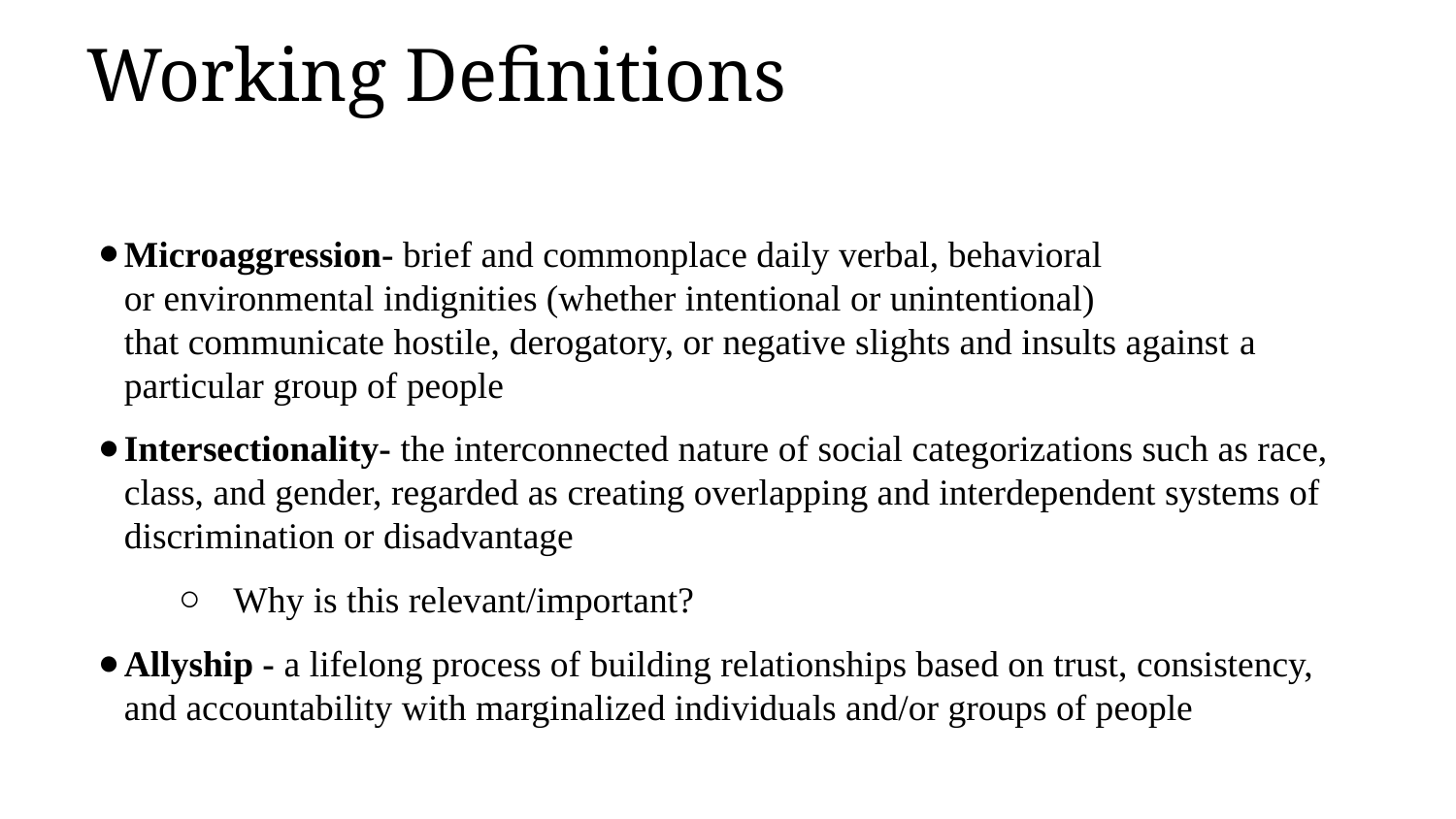

# Working Definitions
Microaggression- brief and commonplace daily verbal, behavioral or environmental indignities (whether intentional or unintentional) that communicate hostile, derogatory, or negative slights and insults against ​a particular group of people
Intersectionality- the interconnected nature of social categorizations such as race, class, and gender, regarded as creating overlapping and interdependent systems of discrimination or disadvantage​
Why is this relevant/important?
Allyship - a lifelong process of building relationships based on trust, consistency, and accountability with marginalized individuals and/or groups of people

## Slide 11
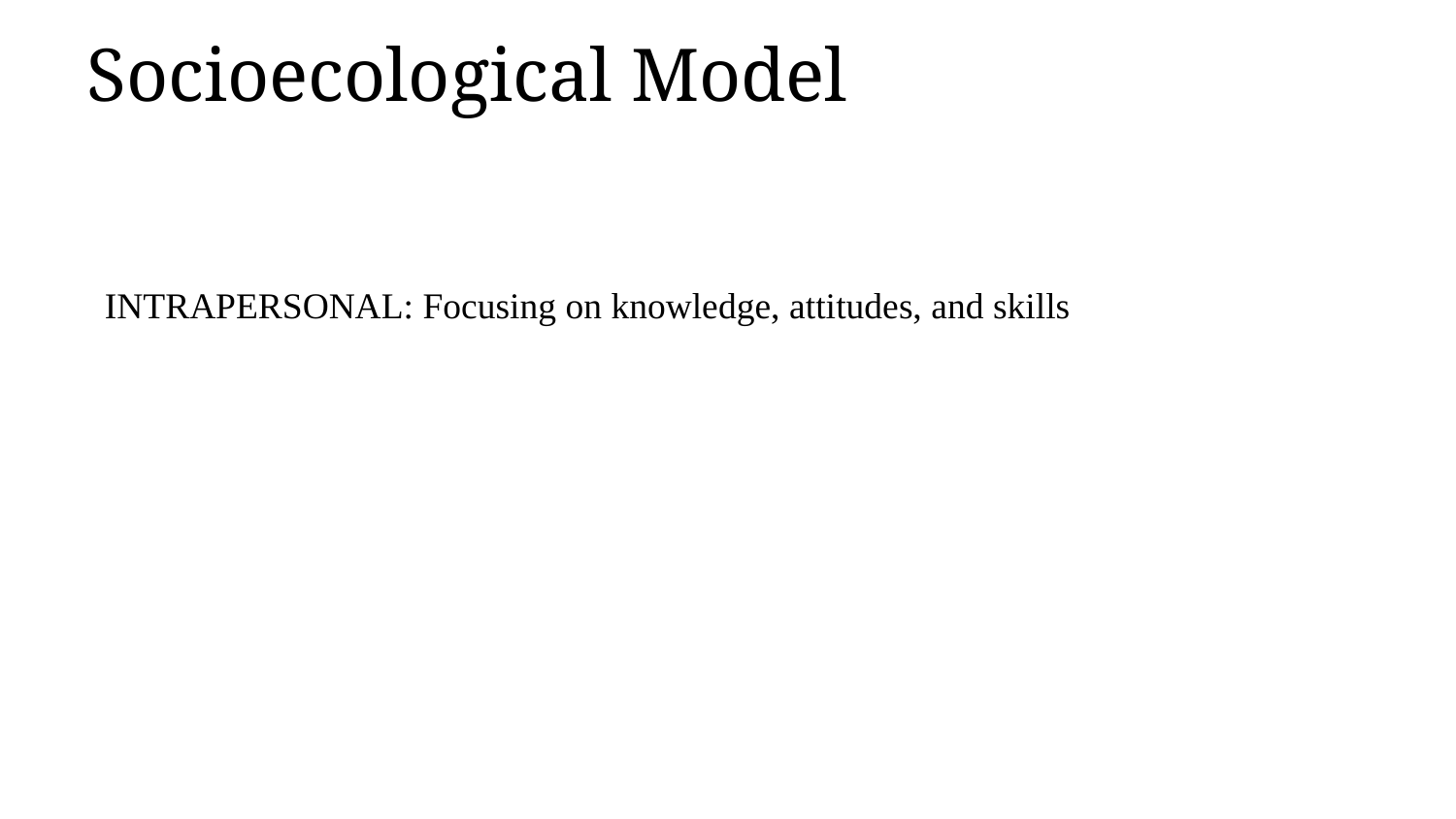

# Socioecological Model
INTRAPERSONAL: Focusing on knowledge, attitudes, and skills

## Slide 12
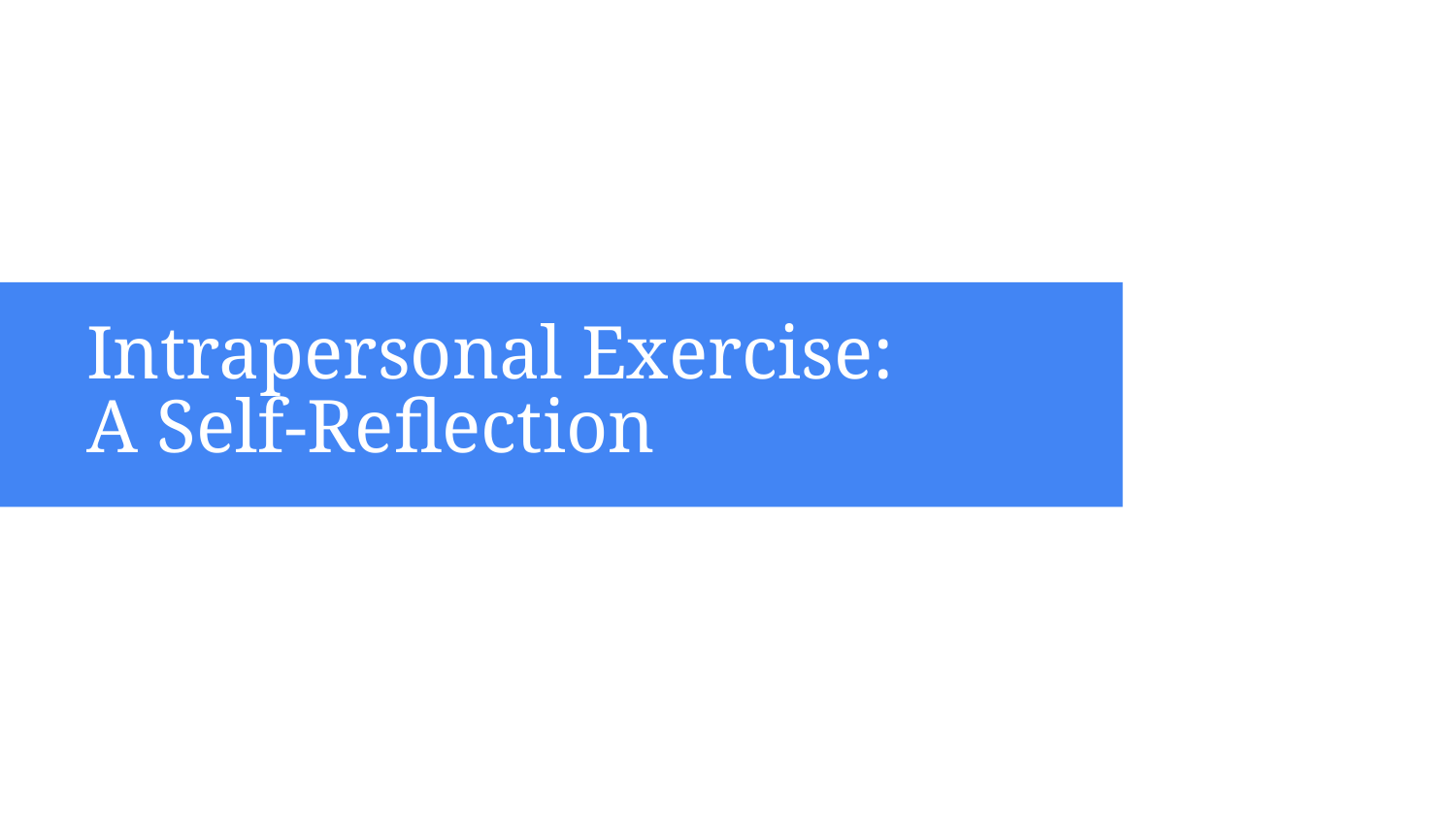

# Intrapersonal Exercise:
A Self-Reflection

## Slide 13
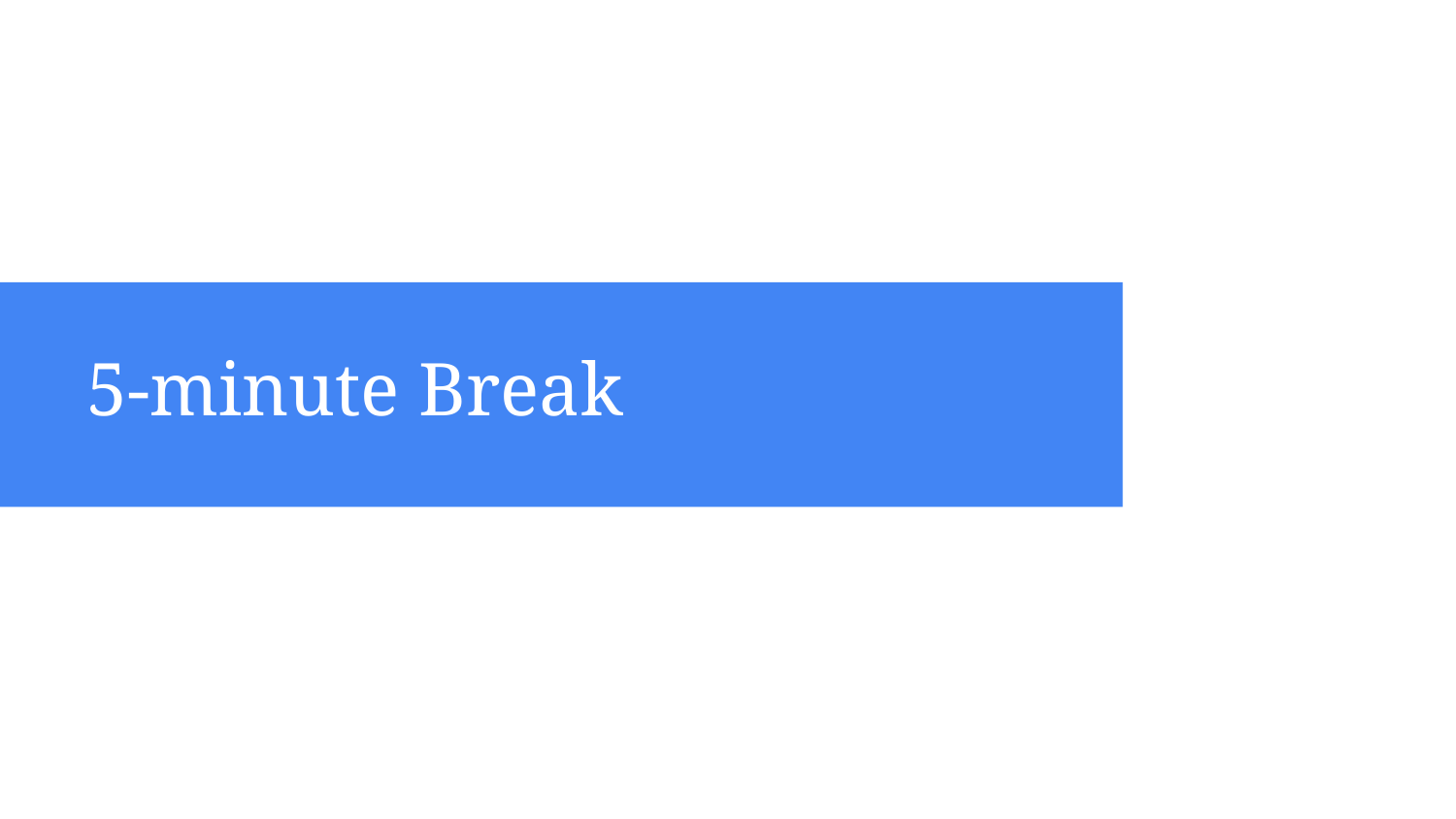

# 5-minute Break

## Slide 14
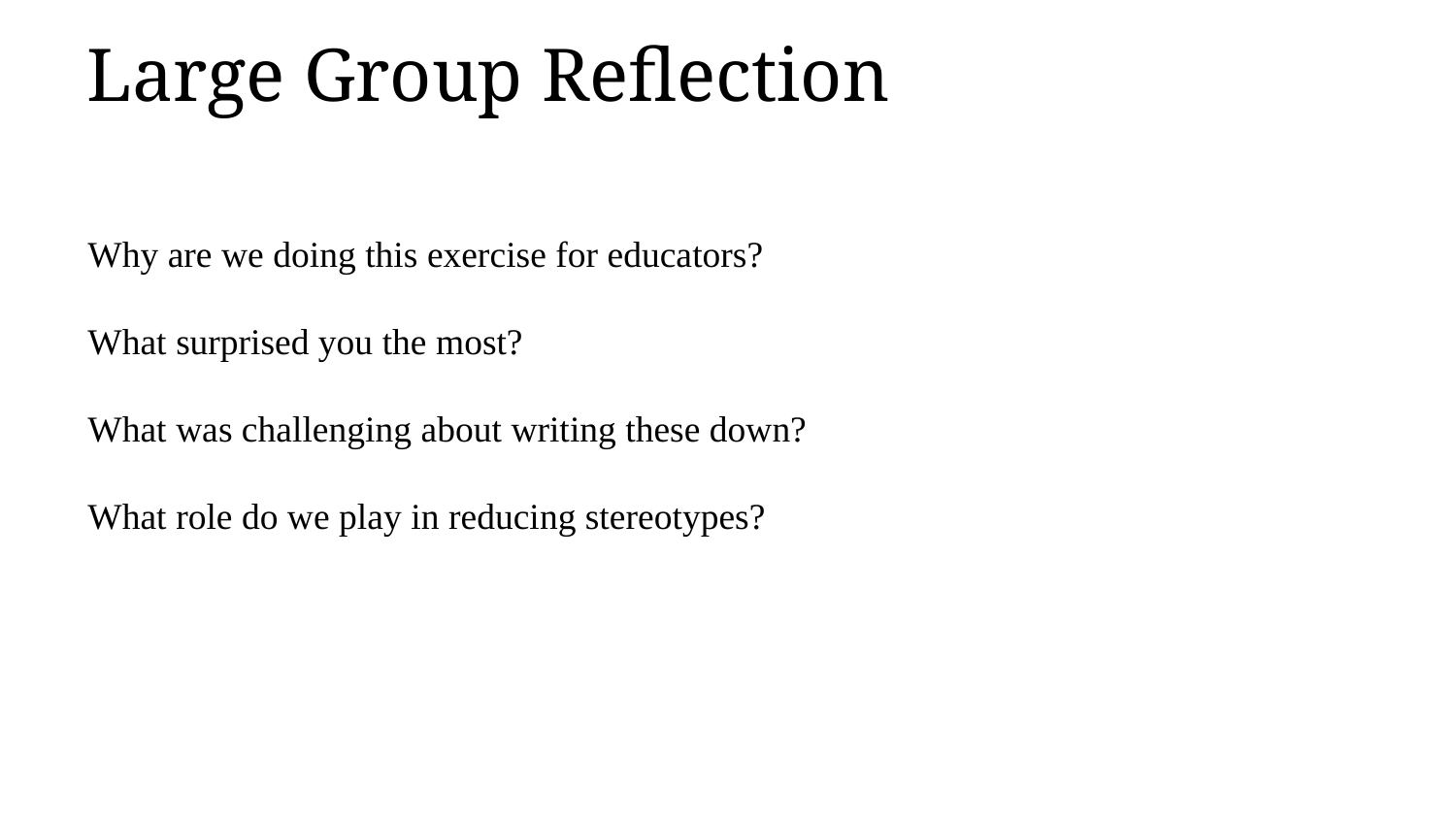

# Large Group Reflection
Why are we doing this exercise for educators?
What surprised you the most?
What was challenging about writing these down?
What role do we play in reducing stereotypes?

## Slide 15
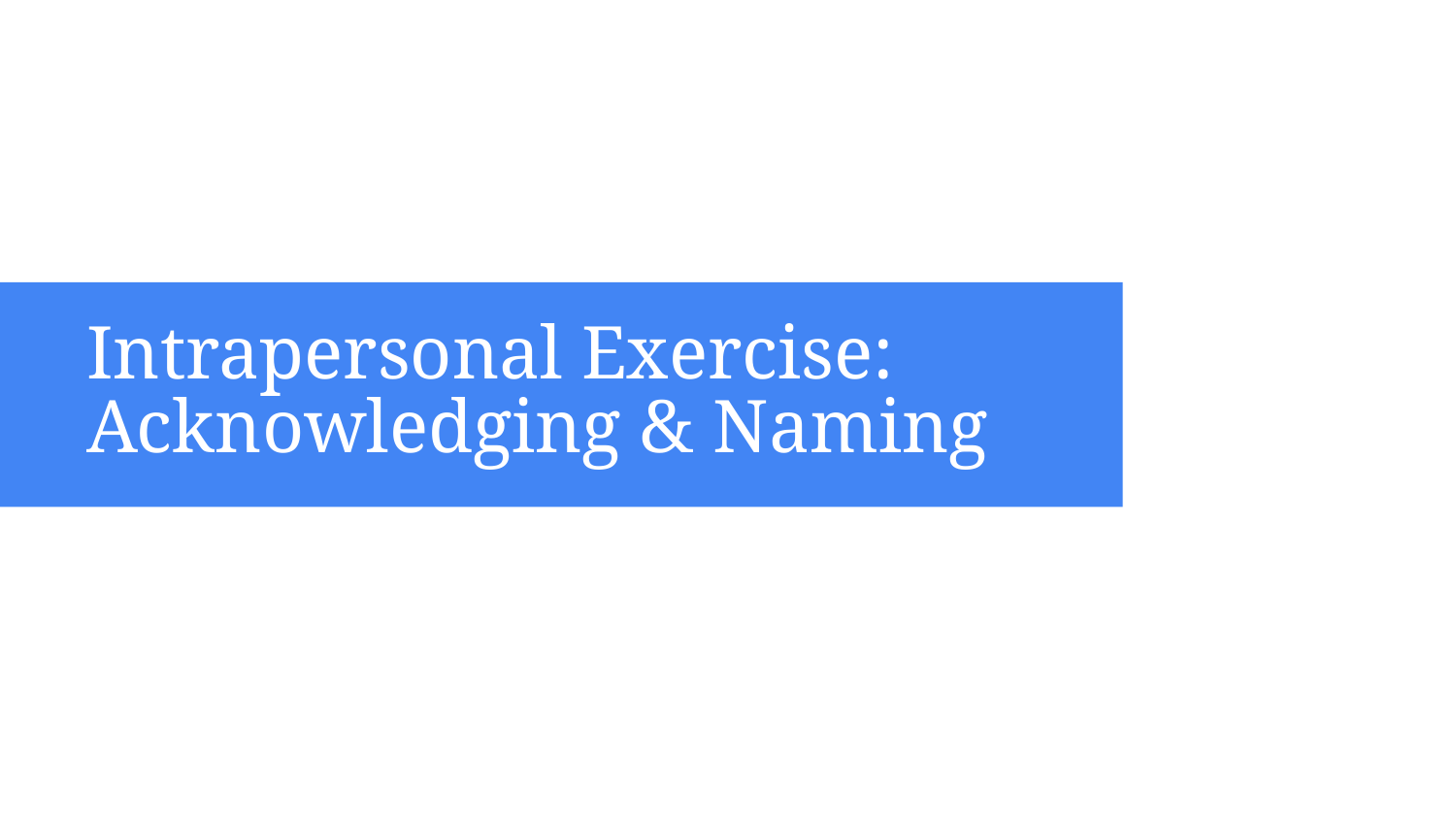

# Intrapersonal Exercise:
Acknowledging & Naming

## Slide 16
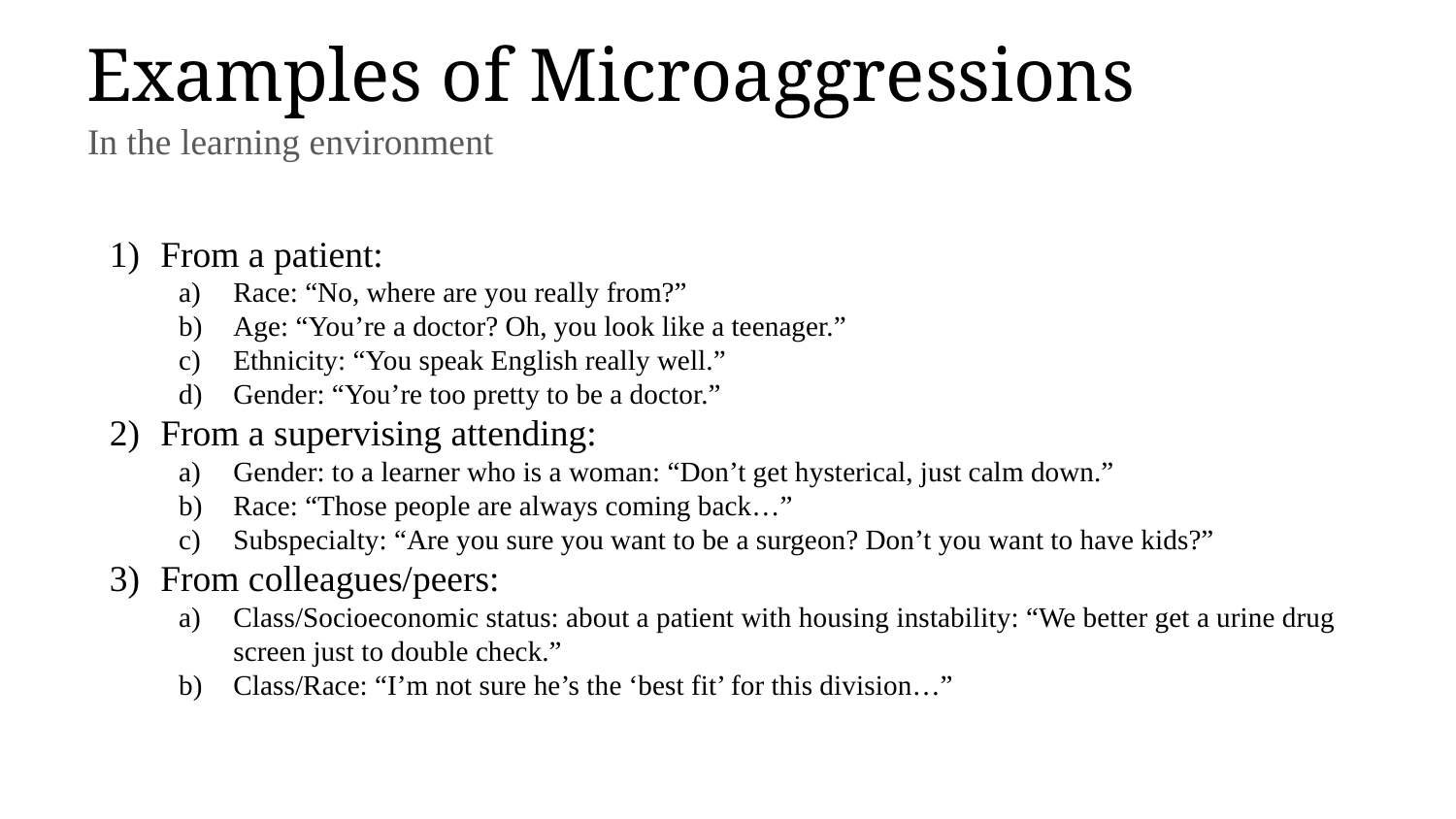

# Examples of Microaggressions
In the learning environment
From a patient:
Race: “No, where are you really from?”
Age: “You’re a doctor? Oh, you look like a teenager.”
Ethnicity: “You speak English really well.”
Gender: “You’re too pretty to be a doctor.”
From a supervising attending:
Gender: to a learner who is a woman: “Don’t get hysterical, just calm down.”
Race: “Those people are always coming back…”
Subspecialty: “Are you sure you want to be a surgeon? Don’t you want to have kids?”
From colleagues/peers:
Class/Socioeconomic status: about a patient with housing instability: “We better get a urine drug screen just to double check.”
Class/Race: “I’m not sure he’s the ‘best fit’ for this division…”

## Slide 17
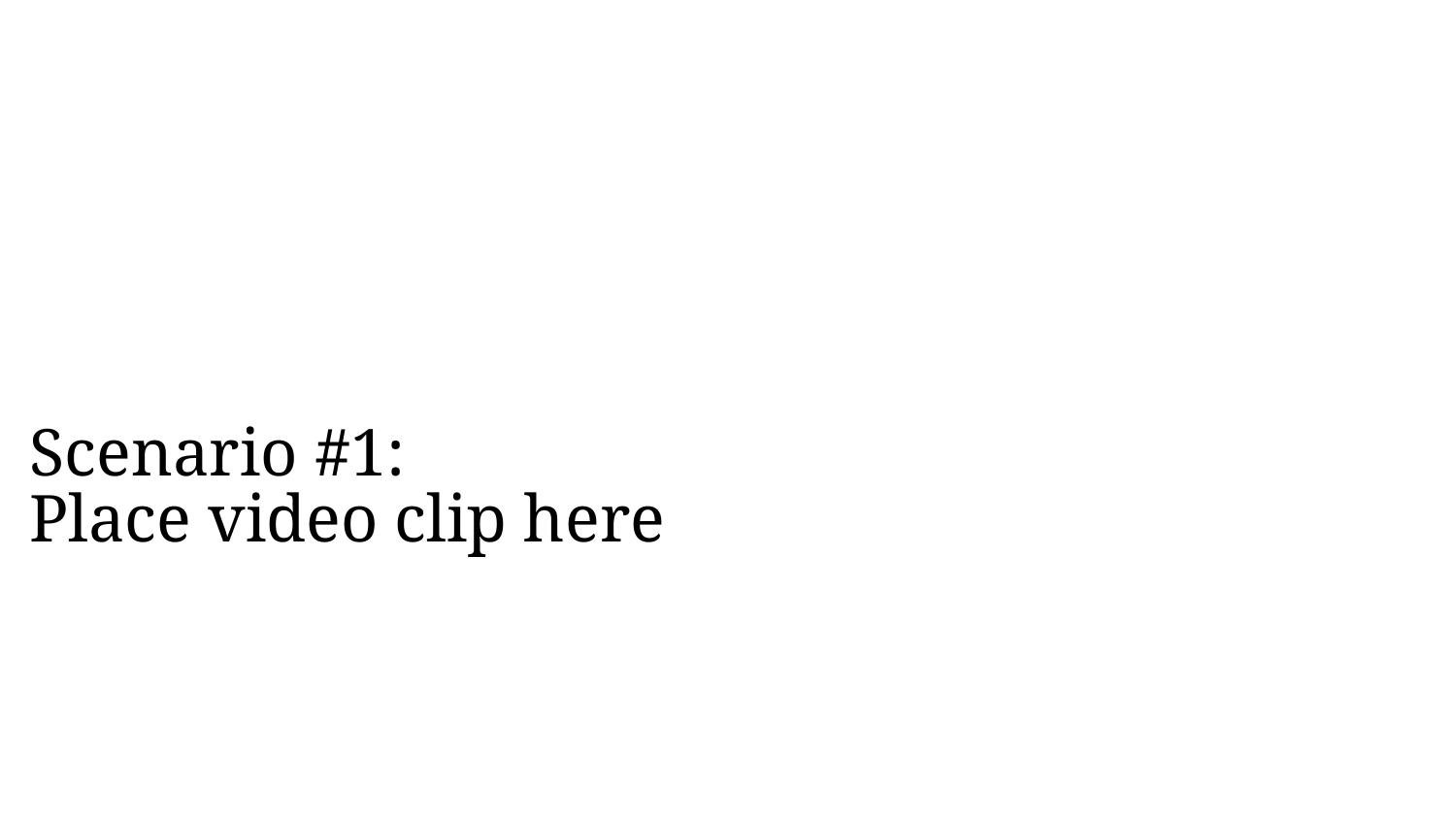

# Scenario #1:
Place video clip here

## Slide 18
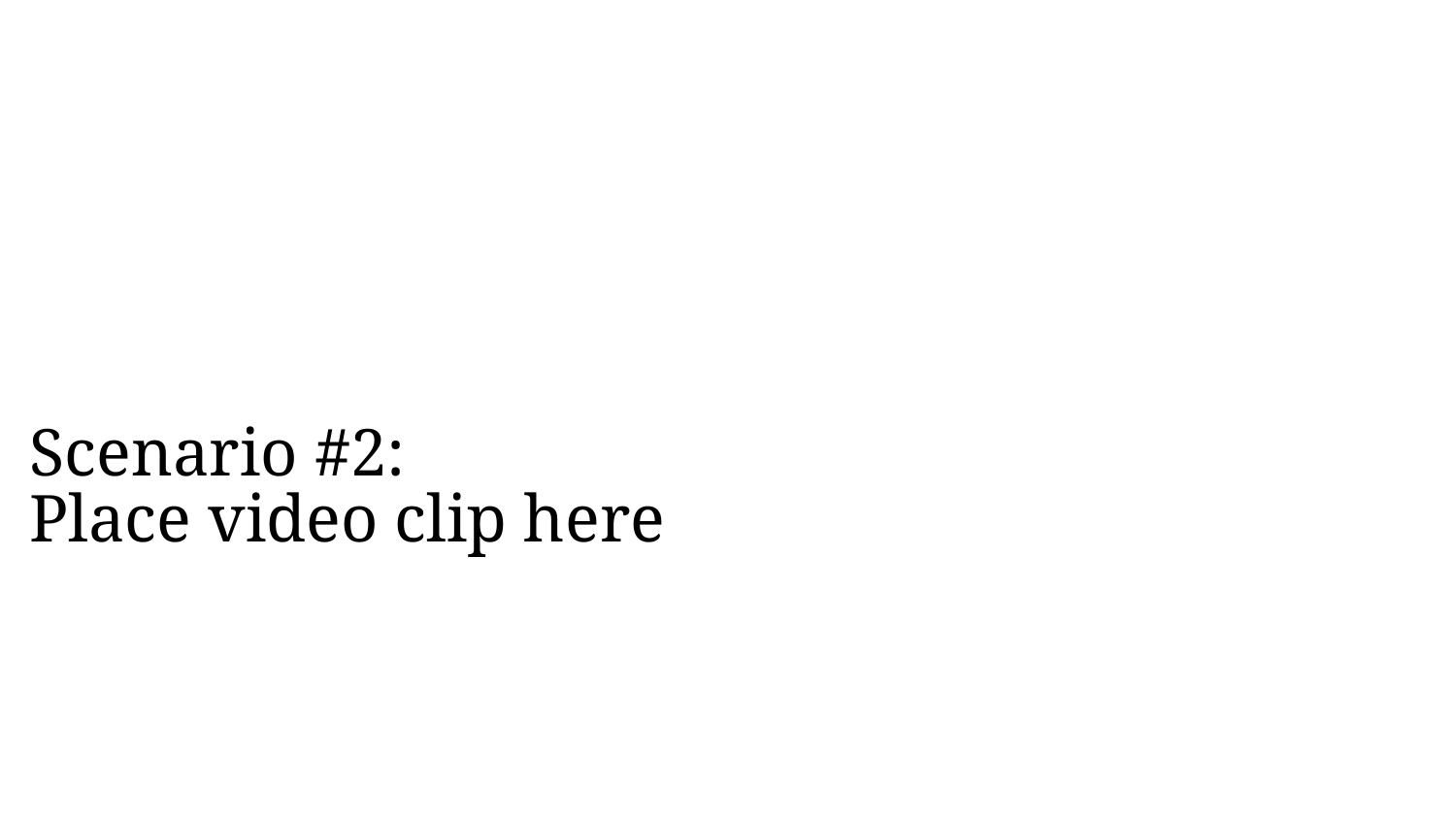

# Scenario #2:
Place video clip here

## Slide 19
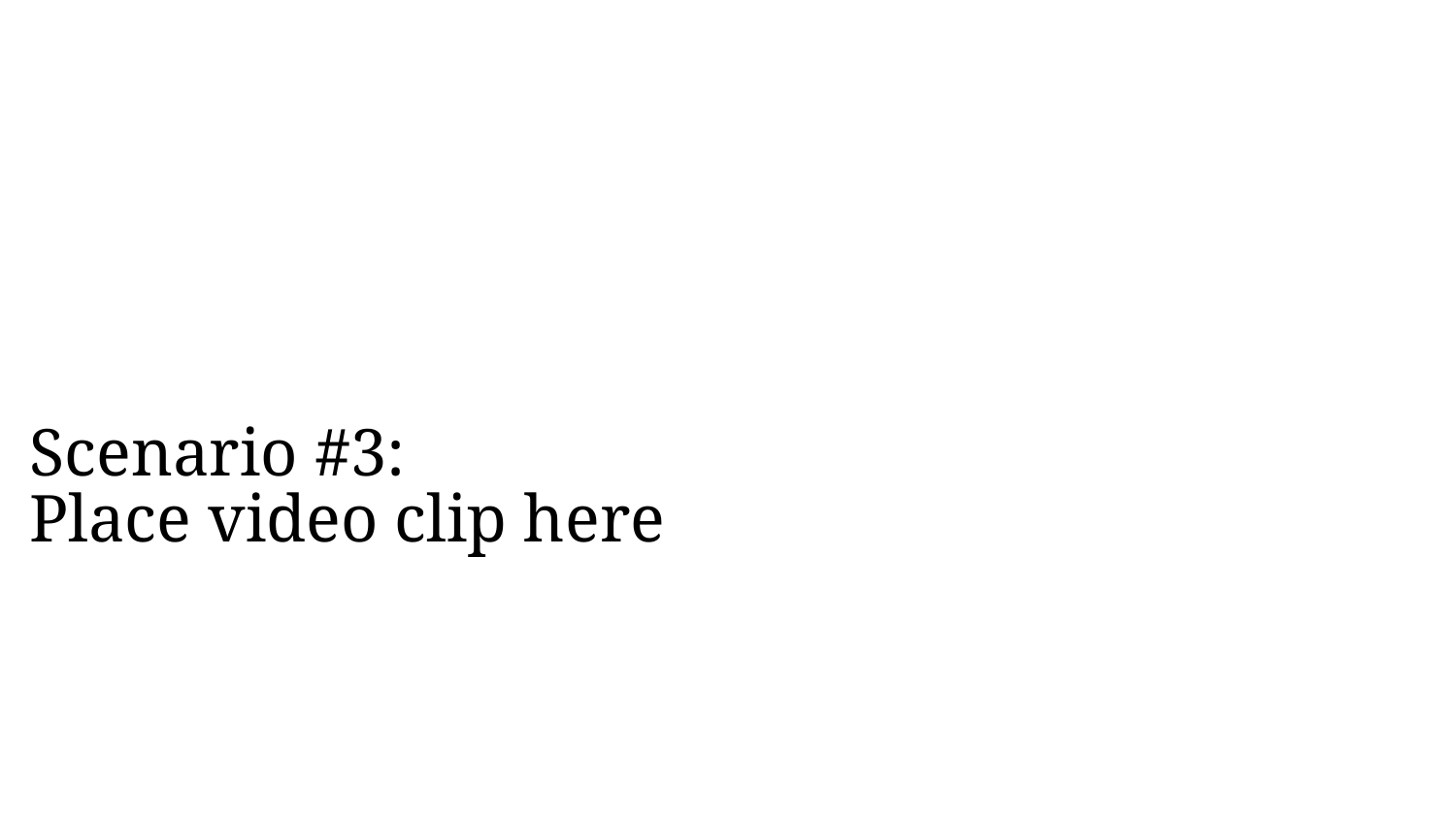

# Scenario #3:
Place video clip here

## Slide 20
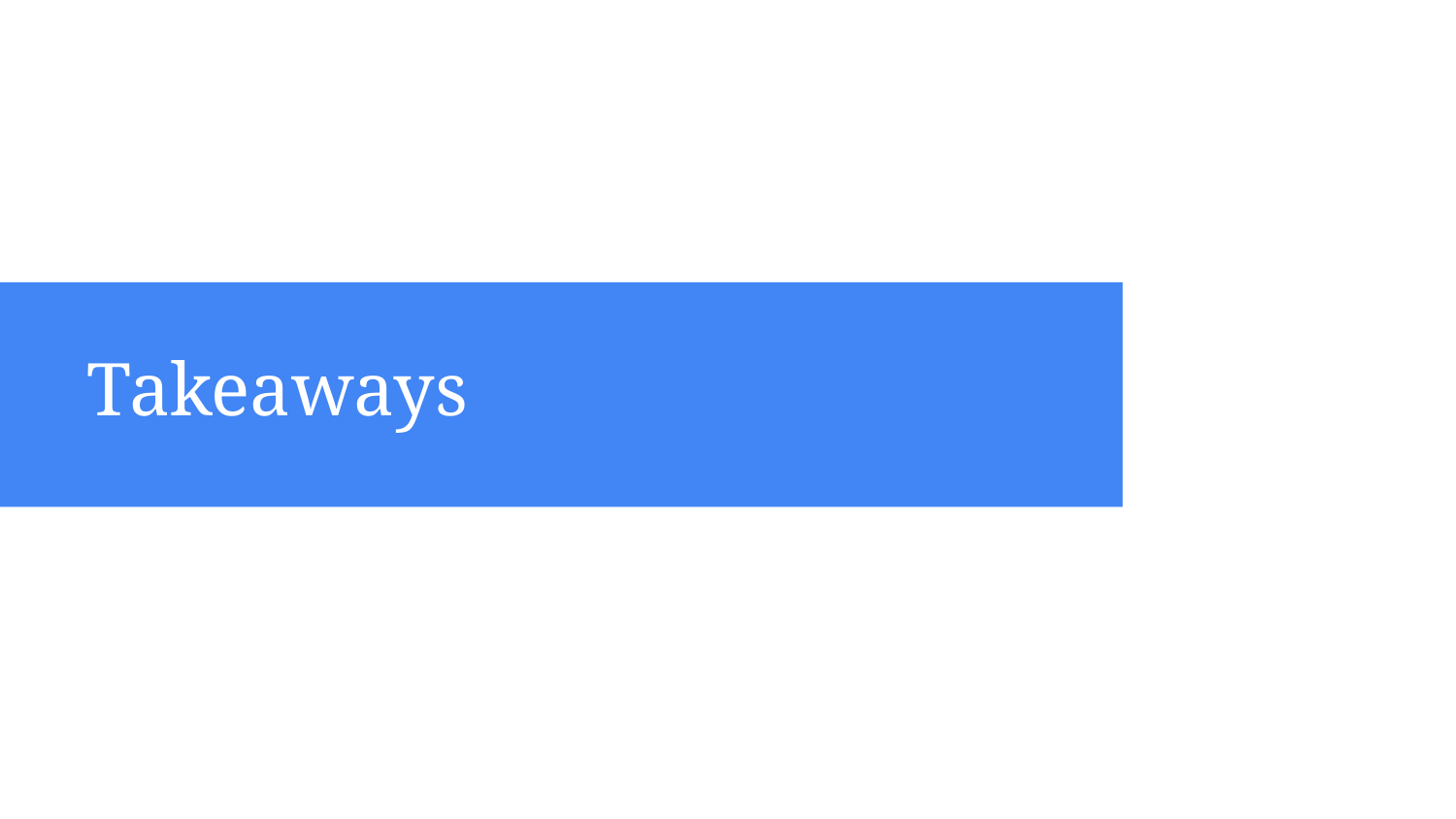

# Takeaways

## Slide 21
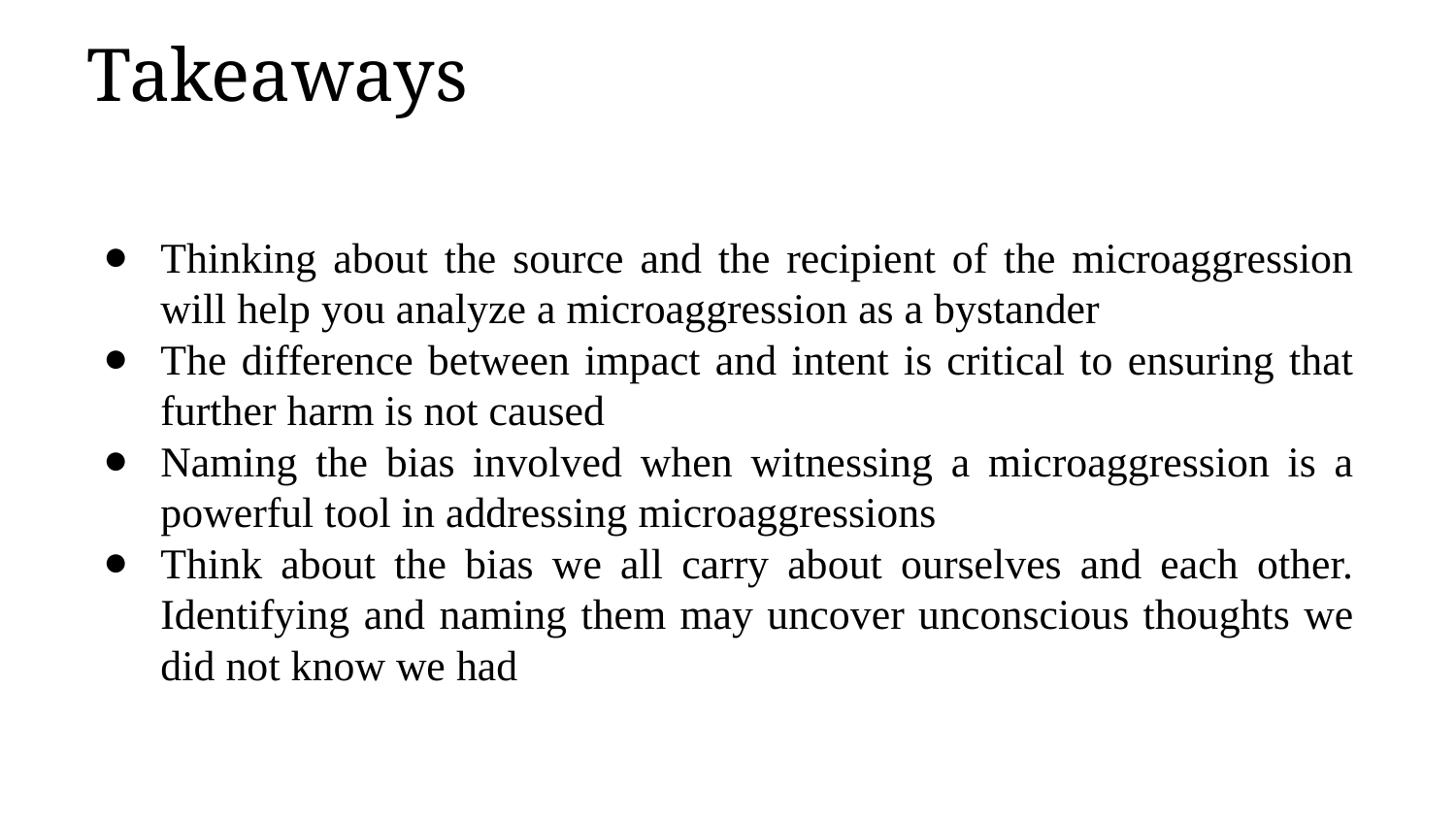

# Takeaways
Thinking about the source and the recipient of the microaggression will help you analyze a microaggression as a bystander
The difference between impact and intent is critical to ensuring that further harm is not caused
Naming the bias involved when witnessing a microaggression is a powerful tool in addressing microaggressions
Think about the bias we all carry about ourselves and each other. Identifying and naming them may uncover unconscious thoughts we did not know we had

## Slide 22
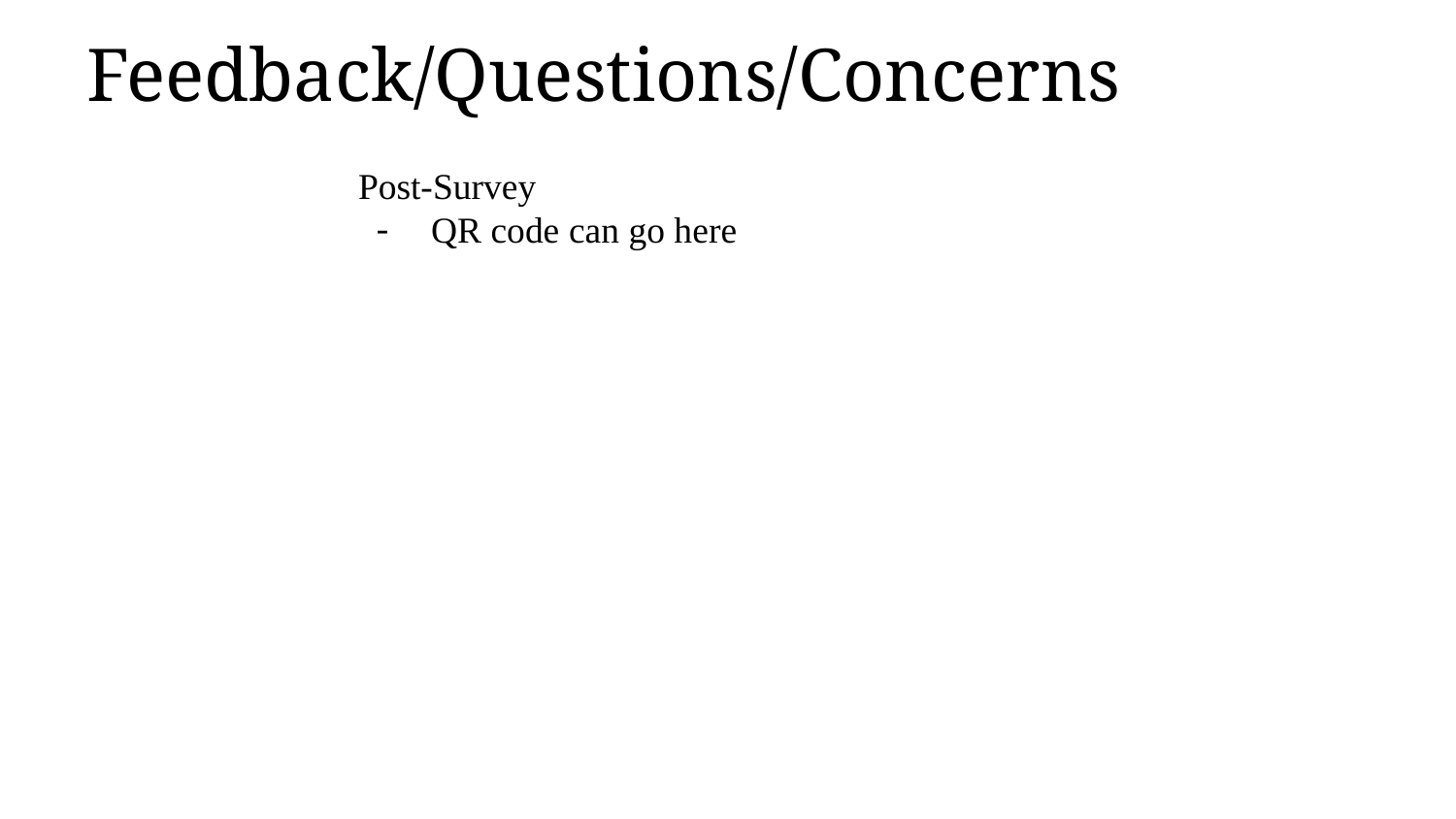

# Feedback/Questions/Concerns
Post-Survey
QR code can go here

## Slide 23
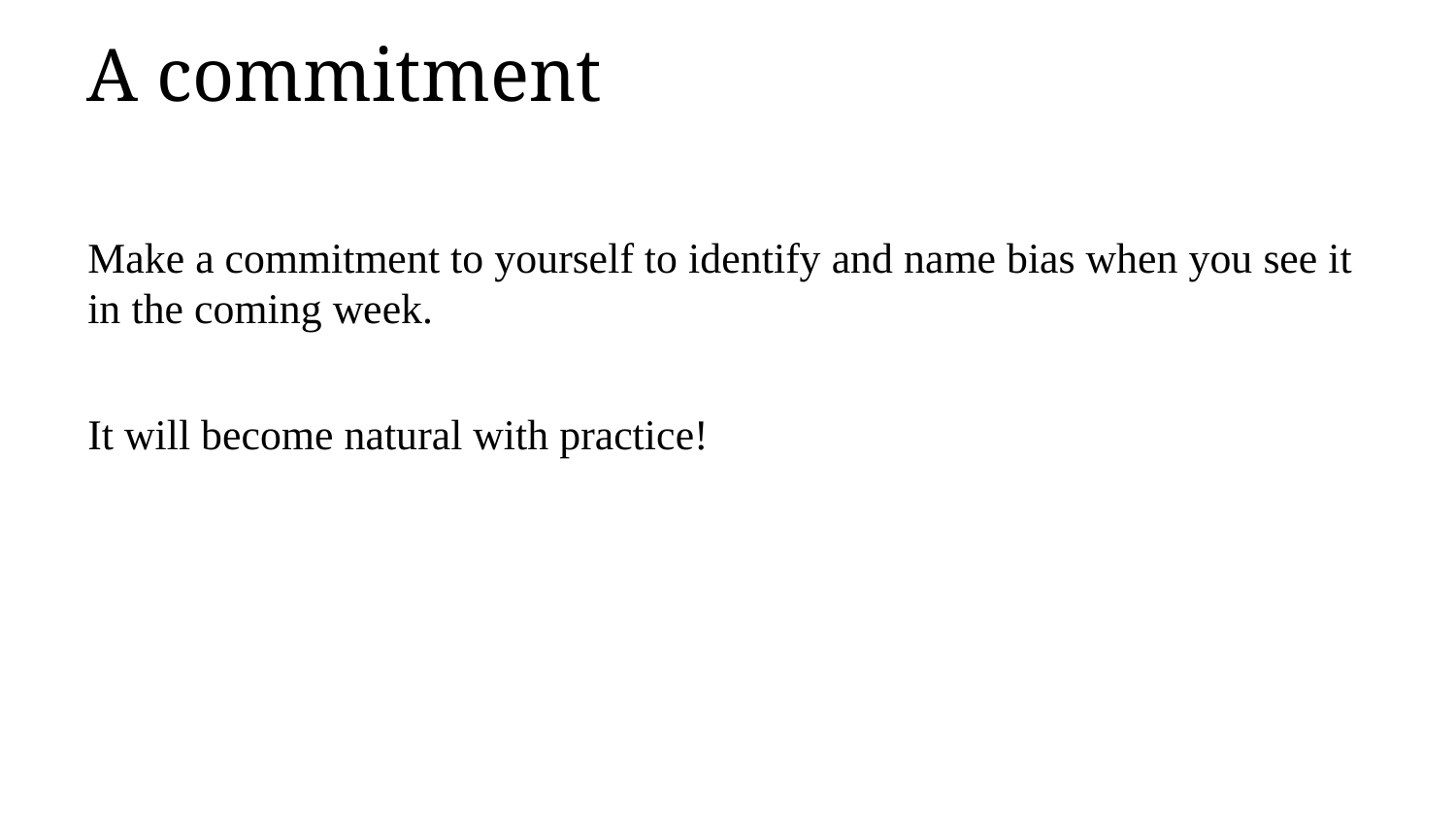

# A commitment
Make a commitment to yourself to identify and name bias when you see it in the coming week.
It will become natural with practice!

## Slide 24
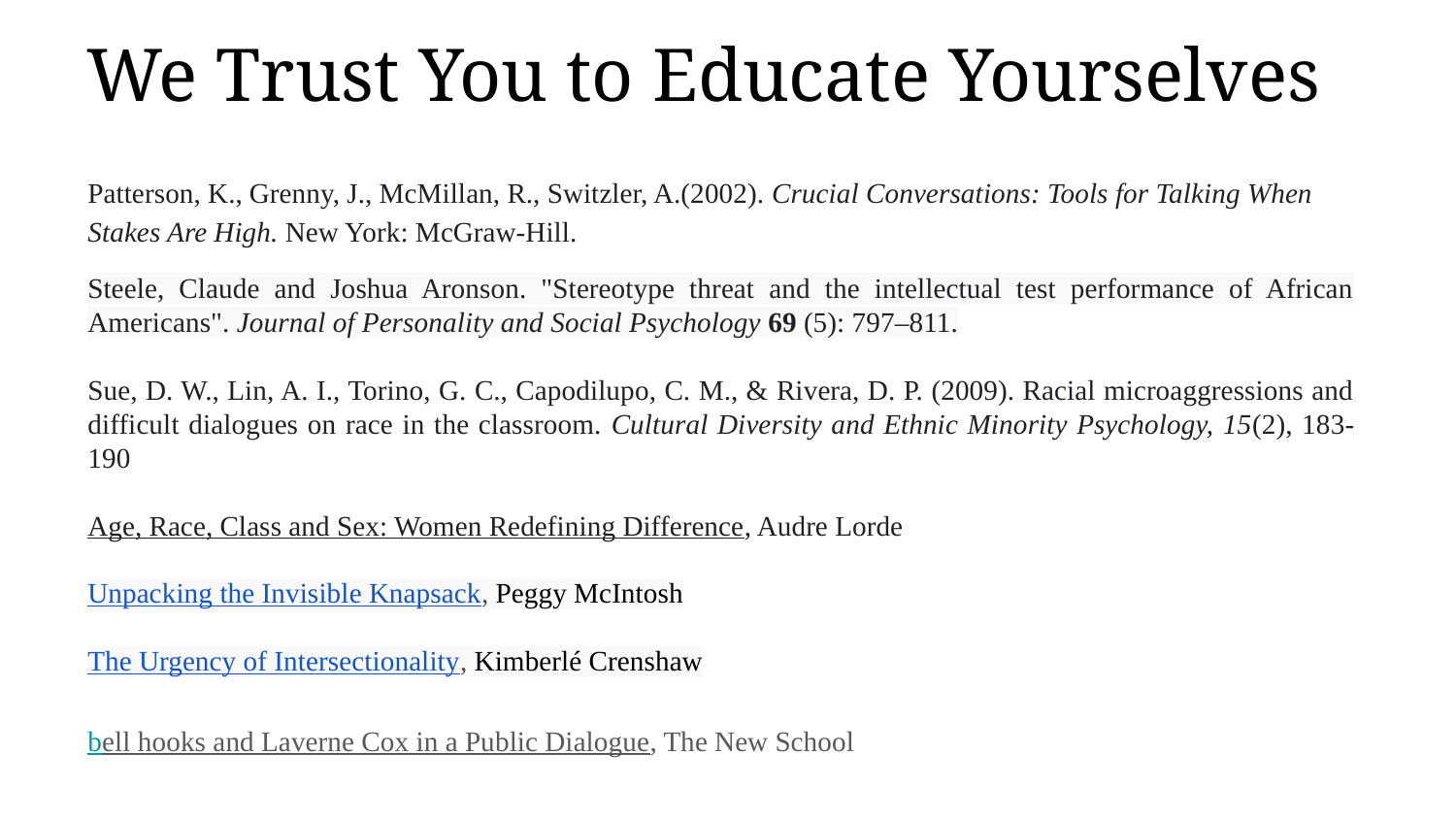

# We Trust You to Educate Yourselves
Patterson, K., Grenny, J., McMillan, R., Switzler, A.(2002). Crucial Conversations: Tools for Talking When Stakes Are High. New York: McGraw-Hill.
Steele, Claude and Joshua Aronson. "Stereotype threat and the intellectual test performance of African Americans". Journal of Personality and Social Psychology 69 (5): 797–811.
Sue, D. W., Lin, A. I., Torino, G. C., Capodilupo, C. M., & Rivera, D. P. (2009). Racial microaggressions and difficult dialogues on race in the classroom. Cultural Diversity and Ethnic Minority Psychology, 15(2), 183-190
Age, Race, Class and Sex: Women Redefining Difference, Audre Lorde
Unpacking the Invisible Knapsack, Peggy McIntosh
The Urgency of Intersectionality, Kimberlé Crenshaw
bell hooks and Laverne Cox in a Public Dialogue, The New School
