# Supplementary material for: Acknowledging and Addressing Microaggressions: A Virtual Experiential Learning Approach for Faculty
Source: MedEdPORTAL. 2024 Sep 4;20:11436. doi: 10.15766/mep_2374-8265.11436 (PMC11374130; doi:10.15766/mep_2374-8265.11436)
Supplement: Supplementary file 1 — Sample Flier.pptxWorkshop 1 - Slides.pptxWorkshop 1 - Facilitator GuideWorkshop 1 - Participant Handout.docxWorkshop 1 - Pre- and Postsurvey.docxWorkshop 2 - Slides.pptxWorkshop 2 - Facilitator Guide.docxWorkshop 2 - Participant Handout.docxWorkshop 2 - Pre- and Postsurvey.docxWorkshop 3 - Slides.pptxWorkshop 3 - Facilitator Guide.docxWorkshop 3 - Participant Handout.docxWorkshop 3 - Pre- and Postsurvey.docxWorkshop 4 - Slides.pptxWorkshop 4 - Facilitator Guide.docxWorkshop 4 - Participant Handout.docxWorkshop 4 - Pre- and Postsurvey.docx [file mep_2374-8265.11436-s001.zip › C. Workshop 1 - Facilitator Guide.docx]

**Faculty Microaggressions Curriculum**

**Acknowledging & Naming Microaggressions**

Workshop #1

Dates:

Time: 2 hours

Where:

Lead Facilitator:

This is the first of a four-part series developed for faculty leaders in Graduate Medical education surrounding the topic of microaggressions. In order to meet learners where they are, earlier workshops will focus on learning and expanding introductory skills surrounding microaggressions, and will lead gradually to more advanced skills such as apologizing to learners when harm has been experienced, setting expectations in the learning environment, and debriefing microaggressions experienced by learners.

Today, we will focus on identifying microaggressions in observed scenarios, determining who the source and recipient of the microaggressions are, and naming the biases involved in each scenario.

This workshop is particularly useful for those who hold multiple pockets of privilege. Learners may have started to learn about bias or perhaps have read a book about the subject, but have not yet had focused training on microaggressions. The learner may not personally encounter microaggressions often.

**Learning Objectives:**

To increase confidence and comfort surrounding the following skills:

1. Identifying sources of privilege in daily life and personal biases that may exist
2. Recognizing a microaggression when witnessing a scenario
3. Naming the source and the recipient of a microaggression in a witnessed scenario

**Workshop Agenda:**

| **Time** | **Topic** | **Participants** |
| --- | --- | --- |
| 0:00 - 0:20 | Introductions/Community Agreements/Ice-Breakers | Large Group-lead Facilitator |
| 0:20 - 0:35 | Common Definitions | Large Group- lead Facilitator |
| 0:35 - 0:45 | Intrapersonal Exercise | Individual Exercise |
| 0:45 - 1:00 | Large Group Reflections | Large Group |
| 1:00 - 1:05 | Stretch Break | -- |
| 1:05 - 1:50 | Small Group Activity | Small Groups- all Facilitators |
| 1:50 - 2:00 | Wrap-Up/Takeaways | Large Group |

**Introductions/Community Agreements/Ice Breakers (20 minutes)**

Lead facilitator for this workshop will begin the workshop with introduction of the workshop and proposed community agreements. Participants voluntarily agree to this set of operational and behavioral agreements to build trust in this learning space and engage in productive work together. Participants will be invited to add additional agreements for the working session.

Community Agreements

•Respect each other as colleagues and humans

•Confidentiality

- Stories stay within our task force meetings, and lessons may leave the room. Especially when we are talking about specific cases related to our trainees and other divisional members/leaders

•Accountability

- We hold each other accountable for our words, actions, and impact
- We hold each other accountable for adhering to our group agreements

•Use “I” statements

- Our opinions and stories are our own, and we will not make blanket statements about others

•Impact versus intent

- We will recognize the difference between IMPACT and INTENT
- We will hold ourselves and others accountable by acknowledging IMPACT of words and actions when we see/feel/hear it

•Maximize the STRETCH ZONE!

- When we do this work, we are maximally efficient in the STRETCH ZONE, a brave space where we can (and should!) be uncomfortable, yet also be productive and learn
- When we label people and shame others, it puts people in the PANIC ZONE, and decreases buy-in to continue our mission

•Ask questions and invite other perspectives continually (i.e. humble inquiry)

- Replace the idea of perfection with a growth mindset, a lifelong journey of learning, effort, and persistence

•Prioritize self-care

- We understand that there may be stories, descriptions of events, and content that may be triggering or re-traumatizing to experience
- In a community of practice, we encourage care for our own mental health, in whatever sustainable form in which that takes shape
- We aim to be present in support of each other during challenging moments

Virtual Agreements can help create guidelines for operating in a virtual learning environment as well.

•Name/Pronouns/Role

- We invite participants to re-title their virtual presence with their name, pronunciation of name, pronouns (if comfortable sharing), and role as faculty

•Cameras on if possible

- To simulate in-person interactions, we invite participants to have cameras on throughout the educational session
- We understand that this is not possible based on physical location, background distractions, and internet access/bandwidth

•Mute when not speaking

- To encourage active listening and one speaker talking at a time, we promote the use of the mute button when not speaking
- This also makes closed-captions for accessibility more feasible

•Use the raise hand function to speak next

- This helps facilitate one speaker at a time and participants not speaking over each other, preventing miscommunication

•Feel free to use the chat and reactions

- For those who prefer written expression to verbal expression, this is an opportunity to engage and share perspectives

** It is important to try to include the learners in establishing these agreements. One tactic is collaborating together on coming up with specific categories of norms for conversations. Examples include brainstorming together norms for topics such as how to respect others and yourself, accepting challenge and discomfort, and keeping an open mind. It is also important that each participant agrees to the set of community agreements before moving forward with the work. This can be done virtually through a reaction, a chat agreement, or gestures in the virtual room.

Tools for further ideas:

<https://radcliffe-harvard-edu-prod.s3.amazonaws.com/8b8bef3c-2b23-4771-9847-625fc015adc4/LeveragingNormsforChallengingConversationsFINAL-ua.pdf>

<https://guidetoteaching.newschool.org/community-agreements/>

Following community agreements, facilitators can share the learning objectives, topics of the longitudinal course, and the agenda for the workshop.

Then each participant will have an opportunity to introduce themselves and the answer to an ice breaker:

“My name is ___ and I am from ___. One thing you cannot tell just by looking at me is

___. This is important for me to tell you because ___.”

Lead facilitator will role model this first to develop a brave space for sharing, and pass it on to the next facilitator/participant until all people in the workshop have shared.

**Common Definitions (15 minutes)**

Lead facilitator will begin short didactics surrounding the main objectives for the workshop, working definitions for the workshop, and instructions for the intrapersonal exercise.

Working Definitions

- **Stereotype-** a widely held but fixed and oversimplified image or idea of a particular type of person or thing
  - **Stereotype threat**- being in a situation or doing something to which a negative stereotype about (an) identity is relevant. i.e. a woman feeling very worried to take a math and science test because of negative stereotypes associated with women in the STEM industry - women are not good at math...
- **Unconscious bias-** social stereotypes about certain groups of people that individuals form outside their own conscious awareness​
- **Privilege-** operates on personal, interpersonal, cultural, and institutional levels and gives advantages, favors, and benefits to members of dominant groups at the expense of members of non-dominant groups​.
  - Examples of groups that enjoy *UNEARNED* privilege: white people, men, able-bodied individuals, cis-gender individuals, wealthy individuals
- **Microaggression-** brief and commonplace daily verbal, behavioral or environmental indignities (whether intentional or unintentional) that communicate hostile, derogatory, or negative slights and insults against ​a particular group of people. It is important to note that the term micro refers to interactions between individuals, not the impact on the individuals, which can be immense and feel very “macro” to recipients of microaggressions. The term was coined by Dr. Chester Pierce, a Harvard psychiatrist who became the founding president of Black Psychiatrists of America, and we pay respect to this incredible physician who brought public attention to the everyday racism faced in America in the 1960s (Williams, 2019).
- **Intersectionality-** the interconnected nature of social categorizations such as race, class, and gender, regarded as creating overlapping and interdependent systems of discrimination or disadvantage​
- **Allyship -** a lifelong process of building relationships based on trust, consistency, and accountability with marginalized individuals and/or groups of people

**Intrapersonal Exercise (10 minutes)**

Next facilitator discusses the intrapersonal exercise. When teaching content related to diversity, equity, and inclusion (DEI), we commonly refer to the social ecological model (Golden et al, 2020) of health, understanding that health outcomes and health disparities are a result of intrapersonal attitudes and interpersonal relationships, along with the institutions, systems, culture, and policies that surround us. We start with an intrapersonal exercise to understand our own identities and some of the stereotypes that come along with it.

Stereotypes are common, and can be hurtful to people, making it difficult to celebrate identities. We will use some time to personally reflect on the parts of our identities that make us proud, and dispel the stereotypes that we may encounter along with them.

Fold a piece of paper in half lengthwise. On the left side, write “I am…” and write “But” on the middle line, followed by “I am not…” on the right side.

Take 10 minutes to write down at least five statements that fit in these columns.

If done early, reflect upon how this exercise made you feel when you were able to challenge stereotypes. Where did you learn these stereotypes? How did you choose which identities you placed on the piece of paper?

**Large Group Reflection Questions (15 minutes)**

Once the 10 minutes are up, the lead facilitator will re-gather the large group for reflection.

If participants are comfortable sharing, they will be invited to share 1-2 words that describe what this activity was like for them. What surprised them the most? What was challenging about writing these down?

Finally, pose to the large group, “What role do we play in reducing stereotypes?”

**Small Group Scenarios (45 minutes)**

As facilitators move to interpersonal exercises, we review some of the common microaggressions that may be witnessed in our learning environments. Facilitators can read these and ask participants if they have seen similar comments or if the comments resonate with experiences they have witnessed.

1. From a patient:
   1. “No, where are you really from?”
      1. Implies that the individual is not from this country or is foreign this land or culture
   2. “You’re a doctor? Oh, you look like a teenager.”
      1. Indicates that the individual may not be experienced or old enough for the responsibilities that are required for the profession
      2. Assumes age and performance based on looks alone
   3. “You speak English really well.”
      1. Relays the expectation that being from another country generally means incomprehensible or inferior English language skills
   4. “You’re too pretty to be a doctor.”
      1. These types of statements would not be made to males and speaks to the gender dominance historically in the medical field
2. From a supervising attending:
   1. To a learner who is a woman: “Don’t get hysterical, just calm down.”
      1. Assumes that women are more emotional and likely to create a scene
   2. “Those people are always coming back…”
      1. Refers to race or groups of people in a dismissive fashion
   3. “Are you sure you want to be a surgeon? Don’t you want to have kids?”
      1. Questions decision-making ability and often signifies sub-specialty disrespect in medicine
      2. Questions standards of family and care-taking ability
3. From colleagues/peers:
   1. About a patient with housing instability: “We better get a urine drug screen just to double check.”
      1. Assumes that somebody who does not have a stable home is automatically lying
      2. Stereotypes individuals who do not have housing as people who use drugs
      3. Blames individuals by implying that their behaviors created this circumstance
   2. “I’m not sure he’s the ‘best fit’ for this division…”
      1. Represents code for not meeting specific standards of elitism or class for a title
      2. Often also represents racism as the “best fit” has historically been white, male, and cisgendered

Next, small groups will be created to transition to thinking about how stereotypes and bias play out in scenarios that we may witness on a regular basis, inside or outside of the workplace. In small groups of 3-4 participants, learners will review three different video clips portraying microaggressions. The time allotted is about 45 minutes, leaving 15 minutes for discussion of each of the three scenarios.

We recommend finding three different video clips that represent three distinct types of microaggressions. These video clips can be found in movies, on the internet, or any other type of media forums. We recommend the clips be short; ie. 30-60 seconds long and have obvious microaggressions portrayed.

In our presentation, we utilized videos that demonstrate ageism, racism and sexism. Below are sample discussion questions that we utilized for each video viewed.

Scenario #1-

After watching the first video, facilitator should pause the video for discussion questions:

- Did you witness a microaggression?
- Who was the source of the microaggression?
- Who was the target of the microaggression?
- What is this about? What stereotypes or bias might be at play?
- What do you think was the impact of this microaggression?

*How does it feel when somebody tells you “don’t look like” what they expected?*

*How might it feel when that is tied to your professional identity? i.e. “You don’t*

*look like a doctor.”*

*There is no right answer to this, but some feelings that may come up include fear,*

*shame, worthlessness, insecurity, outcast, excluded, etc.*

*Every time we witness a microaggression as a bystander, think about the impact on the person experiencing it. Think about if you were experiencing it. What harm do you think this causes one time? How about after experiencing it many times?*

Scenario #2-

After watching the second video, facilitator should pause the video for discussion questions:

- Did you witness a microaggression?
- Who was the source of the microaggression?
- Who was the target of the microaggression?
- What is this about? What stereotypes or bias might be at play?
- How does it feel to witness this microaggression? What do you think was the impact of this microaggression? What do you think the intent was? Does the intent matter?

*Some may feel embarrassed to watch this scene. Some may feel uncomfortable or awkward. Some may feel nervous about what to say. These are normal feelings.*

*Thinking about this scenario, these comments can create a very clear sense of othering and exclusion to the person it's directed toward. These sorts of comments can make people feel unwelcome, unaccepted and uncomfortable.*

*Intent does not matter as much as the impact. When we separate intent and impact, we can better identify the harm.*

Scenario #3 -

After watching the third video, facilitator should pause the video for further discussion:

- Did you witness a microaggression?
- Who was the source of the microaggression?
- Who was the target of the microaggression?
- What is this about? What stereotypes or bias might be at play?
- How does it feel to witness this microaggression? What do you think was the impact of this microaggression? Would it be empowering for anyone to interrupt a microaggression?

*Sometimes it feels uncomfortable watching one person stand up to another person*

*because it is clear that there was harm done. Some people may feel embarrassed*

*that someone they may identify with is making this mistake. Many people may*

*resonate with the attempt to try to keep explaining away the inherent bias when it*

*comes out of our mouths.*

**Wrap-Up/Takeaways (10 minutes)**

Facilitators can take this time to ask learners to self-reflect on their takeaways and learning pearls from this workshop. Ask learners to say aloud one lesson they will take away, and one skill they further hope to learn in the future surrounding microaggressions, stereotypes, or bias.

Some points the facilitator may choose to highlight:

- Thinking about the source and the recipient of the microaggression will help you analyze a microaggression as a bystander
- The difference between impact and intent is critical to ensuring that further harm is not caused
- Naming the bias involved when witnessing a microaggression is a powerful tool in combating microaggressions
- Think about the bias we all carry about ourselves and each other. Naming them may uncover unconscious thoughts we did not know we had

In order to promote a growth mindset, we also acknowledge that learners can continue to learn on their own with the references provided at the end of the slide deck.

References for participants:

- PowerPoint slides
- Participant handout

References for this workshop:

1. Polk, W., & El-Amin, A. (2016). *Leveraging Norms for Challenging Conversations*. <https://radcliffe-harvard-edu-prod.s3.amazonaws.com/8b8bef3c-2b23-4771-9847-625fc015adc4/LeveragingNormsforChallengingConversationsFINAL-ua.pdf>
2. The New School. (2020). *Community Agreements*. Guide to teaching and learning. <https://guidetoteaching.newschool.org/community-agreements/>
3. Williams, M. T. (2019). Microaggressions: Clarification, evidence, and impact. *Perspectives on Psychological Science*, *15*(1), 3–26. <https://doi.org/10.1177/1745691619827499>
4. Golden, T. L., & Wendel, M. L. (2020). Public health’s next step in advancing equity: Re-evaluating epistemological assumptions to move social determinants from theory to practice. *Frontiers in Public Health*, *8*. https://doi.org/10.3389/fpubh.2020.00131
