# Supplementary material for: Acknowledging and Addressing Microaggressions: A Virtual Experiential Learning Approach for Faculty
Source: MedEdPORTAL. 2024 Sep 4;20:11436. doi: 10.15766/mep_2374-8265.11436 (PMC11374130; doi:10.15766/mep_2374-8265.11436)
Supplement: Supplementary file 1 — Sample Flier.pptxWorkshop 1 - Slides.pptxWorkshop 1 - Facilitator GuideWorkshop 1 - Participant Handout.docxWorkshop 1 - Pre- and Postsurvey.docxWorkshop 2 - Slides.pptxWorkshop 2 - Facilitator Guide.docxWorkshop 2 - Participant Handout.docxWorkshop 2 - Pre- and Postsurvey.docxWorkshop 3 - Slides.pptxWorkshop 3 - Facilitator Guide.docxWorkshop 3 - Participant Handout.docxWorkshop 3 - Pre- and Postsurvey.docxWorkshop 4 - Slides.pptxWorkshop 4 - Facilitator Guide.docxWorkshop 4 - Participant Handout.docxWorkshop 4 - Pre- and Postsurvey.docx [file mep_2374-8265.11436-s001.zip › D. Workshop 1 - Participant Handout.docx]

**Microaggressions Curriculum**

**Acknowledging & Naming Microaggressions**

**Skills Handout**

**Learning Objectives:**

To increase confidence and comfort surrounding the following skills:

1. Identifying sources of privilege in daily life and personal biases that may exist
2. Recognizing a microaggression when witnessing a scenario
3. Naming the source and the recipient of a microaggression in a witnessed scenario

Working Definitions

- **Stereotype-** a widely held but fixed and oversimplified image or idea of a particular type of person or thing
  - **Stereotype threat**- being in a situation or doing something to which a negative stereotype about (an) identity is relevant. i.e. a woman feeling very worried to take a math and science test because of negative stereotypes associated with women in the STEM industry - women are not good at math...
- **Unconscious bias-** social stereotypes about certain groups of people that individuals form outside their own conscious awareness
- **Privilege-** operates on personal, interpersonal, cultural, and institutional levels and gives advantages, favors, and benefits to members of dominant groups at the expense of members of non-dominant groups​.
  - Examples of groups that enjoy *UNEARNED* privilege: white people, men, able-bodied individuals, cis-gender individuals, wealthy individuals
- **Microaggression-** brief and commonplace daily verbal, behavioral or environmental indignities (whether intentional or unintentional) that communicate hostile, derogatory, or negative slights and insults against ​a particular group of people. It is important to note that the term micro refers to interactions between individuals, not the impact on the individuals, which can be immense and feel very “macro” to recipients of microaggressions. The term was coined by Dr. Chester Pierce, a Harvard psychiatrist who became the founding president of Black Psychiatrists of America, and we pay respect to this incredible physician who brought public attention to the everyday racism faced in America in the 1960s (Williams, 2019).
- **Intersectionality-** the interconnected nature of social categorizations such as race, class, and gender, regarded as creating overlapping and interdependent systems of discrimination or disadvantage​
- **Allyship -** a lifelong process of building relationships based on trust, consistency, and accountability with marginalized individuals and/or groups of people

**Intrapersonal Exercise**

Stereotypes are common, and can be hurtful to people, making it difficult to celebrate identities. We will use some time to personally reflect on the parts of our identities that make us proud, and dispel the stereotypes that we may encounter along with them.

Fold a piece of paper in half lengthwise. On the left side, write “I am…” and write “But” on the middle line, followed by “I am not…” on the right side.

Take 10 minutes to write down at least five statements that fit in these columns.

If done early, reflect upon how this exercise made you feel when you were able to challenge stereotypes. Where did you learn these stereotypes? How did you choose which identities you placed on the piece of paper?

“What role do we play in reducing stereotypes?”

**Small Group Scenarios**

We will transition to thinking about how stereotypes and bias play out in scenarios that we may witness on a regular basis, in or outside of the workplace.

Scenario #1-

After watching the first video,

- Did you witness a microaggression?
- Who was the source of the microaggression?
- Who was the target of the microaggression?
- What is this about? What stereotypes or bias might be at play?
- What do you think was the impact of this microaggression?

Scenario #2-

After watching the second video,

- Did you witness a microaggression?
- Who was the source of the microaggression?
- Who was the target of the microaggression?
- What is this about? What stereotypes or bias might be at play?
- How does it feel to witness this microaggression? What do you think was the impact of this microaggression? What do you think the intent was? Does the intent matter?

Scenario #3 -

After watching the third video,

- Did you witness a microaggression?
- Who was the source of the microaggression?
- Who was the target of the microaggression?
- What is this about? What stereotypes or bias might be at play?
- How does it feel to witness this microaggression? What do you think was the impact of this microaggression? Would it be empowering to interrupt the microaggression?

**Wrap-Up/Takeaways**

Some points to highlight from the workshop:

- Thinking about the source and the recipient of the microaggression will help you analyze a microaggression as a bystander
- The difference between impact and intent is critical to ensuring that further harm is not caused
- Naming the bias involved when witnessing a microaggression is a powerful tool in combating microaggressions
- Think about the bias we all carry about ourselves and each other. Naming them may uncover unconscious thoughts we did not know we had

**Resources**

Patterson, K., Grenny, J., McMillan, R., Switzler, A.(2002). *Crucial Conversations: Tools for Talking When Stakes Are High.* New York: McGraw-Hill.

Steele, Claude and Joshua Aronson. "Stereotype threat and the intellectual test performance of African Americans". *Journal of Personality and Social Psychology* **69** (5): 797–811.

Sue, D. W., Lin, A. I., Torino, G. C., Capodilupo, C. M., & Rivera, D. P. (2009). Racial microaggressions and difficult dialogues on race in the classroom. *Cultural Diversity and Ethnic Minority Psychology, 15*(2), 183-190

[Age, Race, Class and Sex: Women Redefining Difference](https://www.colorado.edu/odece/sites/default/files/attached-files/rba09-sb4converted_8.pdf), Audre Lorde

[Unpacking the Invisible Knapsack](https://www.youtube.com/watch?v=DRnoddGTMTY), Peggy McIntosh

[The Urgency of Intersectionality](https://www.ted.com/talks/kimberle_crenshaw_the_urgency_of_intersectionality?language=en), Kimberlé Crenshaw

[bell hooks and Laverne Cox in a Public Dialogue](https://www.youtube.com/watch?v=9oMmZIJijgY), The New School
