# Supplementary material for: Acknowledging and Addressing Microaggressions: A Virtual Experiential Learning Approach for Faculty
Source: MedEdPORTAL. 2024 Sep 4;20:11436. doi: 10.15766/mep_2374-8265.11436 (PMC11374130; doi:10.15766/mep_2374-8265.11436)
Supplement: Supplementary file 1 — Sample Flier.pptxWorkshop 1 - Slides.pptxWorkshop 1 - Facilitator GuideWorkshop 1 - Participant Handout.docxWorkshop 1 - Pre- and Postsurvey.docxWorkshop 2 - Slides.pptxWorkshop 2 - Facilitator Guide.docxWorkshop 2 - Participant Handout.docxWorkshop 2 - Pre- and Postsurvey.docxWorkshop 3 - Slides.pptxWorkshop 3 - Facilitator Guide.docxWorkshop 3 - Participant Handout.docxWorkshop 3 - Pre- and Postsurvey.docxWorkshop 4 - Slides.pptxWorkshop 4 - Facilitator Guide.docxWorkshop 4 - Participant Handout.docxWorkshop 4 - Pre- and Postsurvey.docx [file mep_2374-8265.11436-s001.zip › E. Workshop 1 - Pre- and Postsurvey.docx]

**Microaggressions Workshop #1: Acknowledging and Naming Microaggressions PRE-SURVEY**

**Do you consent to using your responses as part of the research surrounding this work? ?**

_____ **YES**, you may use my responses in the research study.

_____ **NO**, you may NOT use my responses in the research study.

**What is your participant ID?** _______________ **(only asked if individual participates in study)**

2-digit birth**DAY** + last 2 letters of birth **CITY** + first initial of each **PARENT** in alphabetical order (use X if unknown)

E.g., Participant ID for a person born on July **09** in Tope**ka** whose parents are **K**yle and **S**am is **09KAKS**

**Demographic Information**

1. **My GME affiliated role is in the department of ____________**
2. **What is your race/ethnicity? (check all that apply)**

_____ American Indian or Alaska Native _____ White

_____ Asian _____ Multi-race/Ethnicity

_____ Black or African American _____ Unknown/Prefer not to say

_____ LatinX _____ Other/self-describe: _______

_____ Native Hawaiian or Other Pacific Islander

1. **What gender do you identify with?**

_____ Female _____ Other (please specify): _______

_____ Male ____ Prefer not to sate

_____ Transgender female

_____ Transgender male

_____ Genderqueer/gender non-conforming

1. **Other identities that I hold related to my leadership/teaching role (free text):**
2. **I prefer to participate in these workshops:**
3. In person
4. Virtually
5. **To what extent do you agree with these statements? (check one per row)**

| **Recognizing** | **Strongly Disagree** | **Disagree** | **Neutral** | **Agree** | **Strongly Agree** |
| --- | --- | --- | --- | --- | --- |
| It is important for me to be able to recognize microaggressions in the learning environment. |  |  |  |  |  |
| I am confident in my ability to recognize microaggressions in the learning environment. |  |  |  |  |  |
| I can differentiate between *sources* and r*ecipients* of microaggressions. |  |  |  |  |  |
| **Naming** | **Strongly Disagree** | **Disagree** | **Neutral** | **Agree** | **Strongly Agree** |
| I am aware of my own personal biases that affect the learning climate. |  |  |  |  |  |
| It is important for me to be able to openly name bias when I see it in the learning environment. |  |  |  |  |  |
| I am confident in my ability to openly name bias when I see it in the learning environment |  |  |  |  |  |
| **Next Steps** | **Strongly Disagree** | **Disagree** | **Neutral** | **Agree** | **Strongly Agree** |
| I feel confident in my ability to open a discussion with learners surrounding stereotypes and bias after experiencing a microaggression. |  |  |  |  |  |

**Microaggression Workshop #1: Acknowledging and Naming Microaggressions POST-SURVEY**

1. **After participating in the microaggression workshop, to what extent do you agree with these statements? (check one per row)**

| **Recognizing** | **Strongly Disagree** | **Disagree** | **Neutral** | **Agree** | **Strongly Agree** |
| --- | --- | --- | --- | --- | --- |
| It is important for me to be able to recognize microaggressions in the learning environment. |  |  |  |  |  |
| I am confident in my ability to recognize microaggressions in the learning environment. |  |  |  |  |  |
| I can differentiate between *sources* and r*ecipients* of microaggressions. |  |  |  |  |  |
| **Naming** | **Strongly Disagree** | **Disagree** | **Neutral** | **Agree** | **Strongly Agree** |
| I am aware of my own personal biases that affect the learning climate. |  |  |  |  |  |
| It is important for me to be able to openly name bias when I see it in the learning environment. |  |  |  |  |  |
| I am confident in my ability to openly name bias when I see it in the learning environment |  |  |  |  |  |
| **Next Steps** | **Strongly Disagree** | **Disagree** | **Neutral** | **Agree** | **Strongly Agree** |
| I feel confident in my ability to open a discussion with learners surrounding stereotypes and bias after experiencing a microaggression. |  |  |  |  |  |

1. What was the **most useful** part of this workshop? Why?
2. What was the **least useful** part of this workshop? Why?
3. What would you **change** about this workshop? Why?
4. Please feel free to offer feedback to your facilitators. Did they create an inclusive learning environment? What did they do well? What could they do better?
5. Commit to one personal change you will make to create a more inclusive learning environment after this workshop:
