# Supplementary material for: Acknowledging and Addressing Microaggressions: A Virtual Experiential Learning Approach for Faculty
Source: MedEdPORTAL. 2024 Sep 4;20:11436. doi: 10.15766/mep_2374-8265.11436 (PMC11374130; doi:10.15766/mep_2374-8265.11436)
Supplement: Supplementary file 1 — Sample Flier.pptxWorkshop 1 - Slides.pptxWorkshop 1 - Facilitator GuideWorkshop 1 - Participant Handout.docxWorkshop 1 - Pre- and Postsurvey.docxWorkshop 2 - Slides.pptxWorkshop 2 - Facilitator Guide.docxWorkshop 2 - Participant Handout.docxWorkshop 2 - Pre- and Postsurvey.docxWorkshop 3 - Slides.pptxWorkshop 3 - Facilitator Guide.docxWorkshop 3 - Participant Handout.docxWorkshop 3 - Pre- and Postsurvey.docxWorkshop 4 - Slides.pptxWorkshop 4 - Facilitator Guide.docxWorkshop 4 - Participant Handout.docxWorkshop 4 - Pre- and Postsurvey.docx [file mep_2374-8265.11436-s001.zip › F. Workshop 2 - Slides.pptx]

## Slide 1
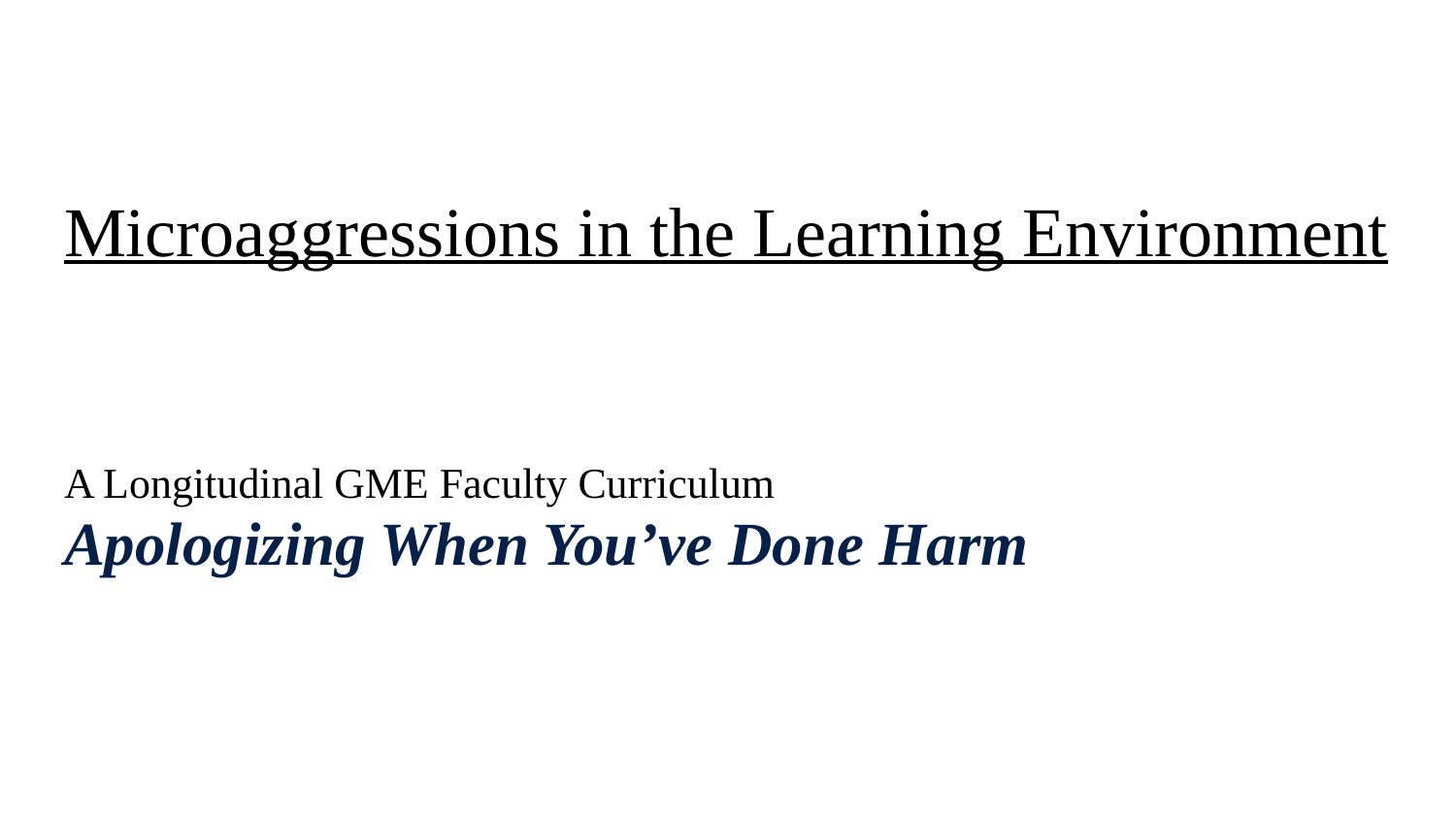

# Microaggressions in the Learning Environment
A Longitudinal GME Faculty Curriculum
Apologizing When You’ve Done Harm

## Slide 2
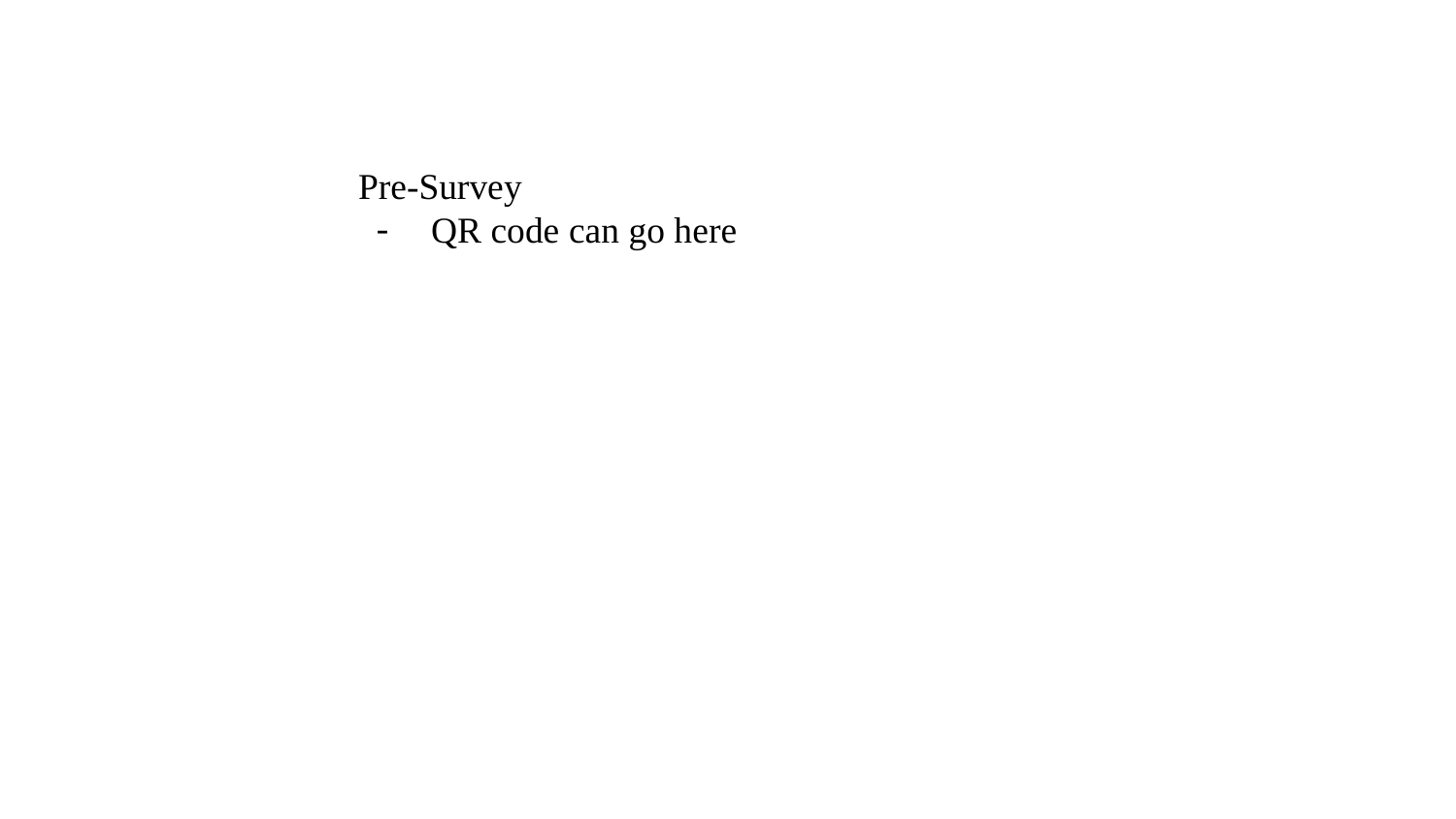

Pre-Survey
QR code can go here

## Slide 3
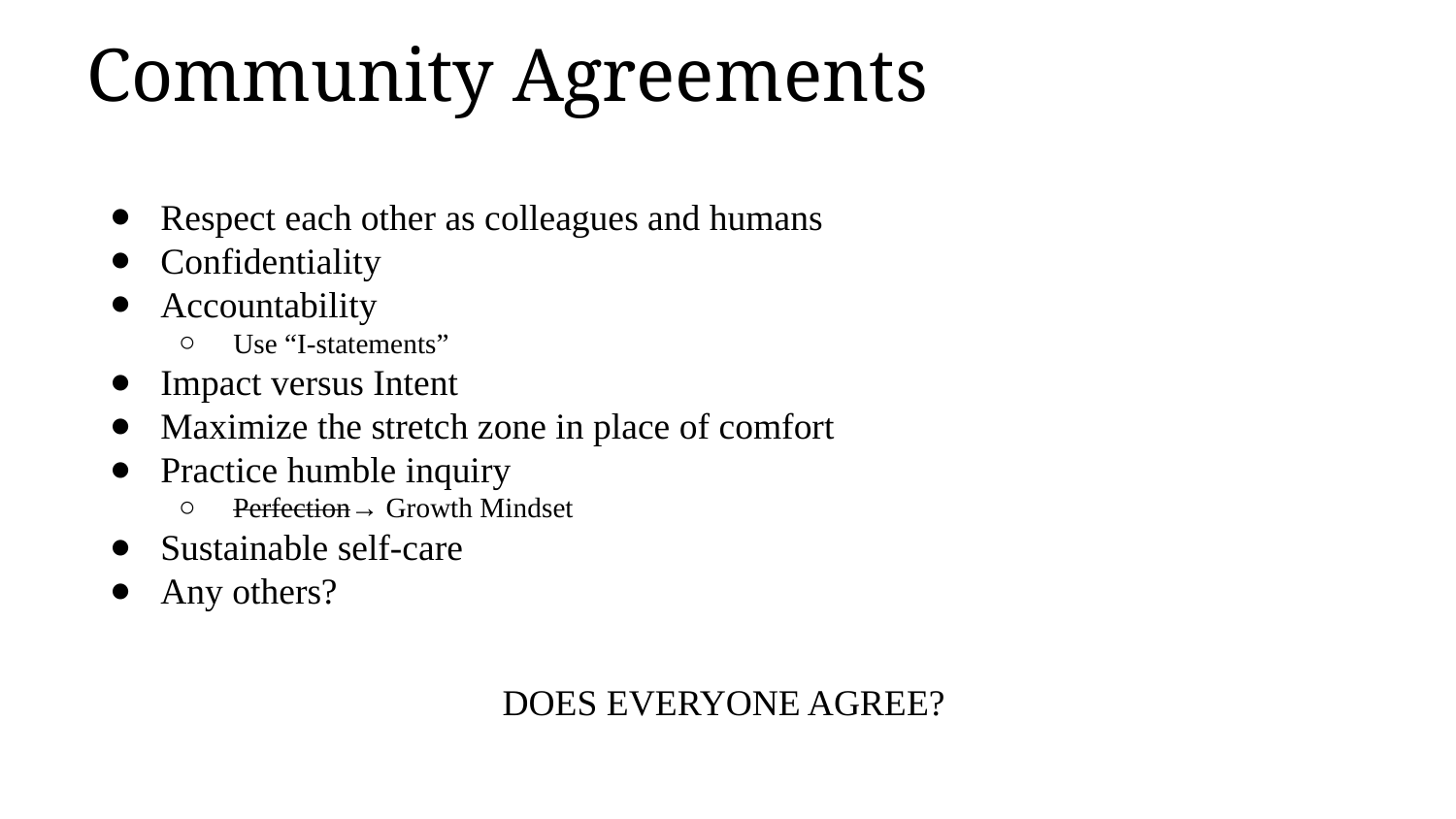

# Community Agreements
Respect each other as colleagues and humans
Confidentiality
Accountability
Use “I-statements”
Impact versus Intent
Maximize the stretch zone in place of comfort
Practice humble inquiry
Perfection→ Growth Mindset
Sustainable self-care
Any others?
DOES EVERYONE AGREE?

## Slide 4
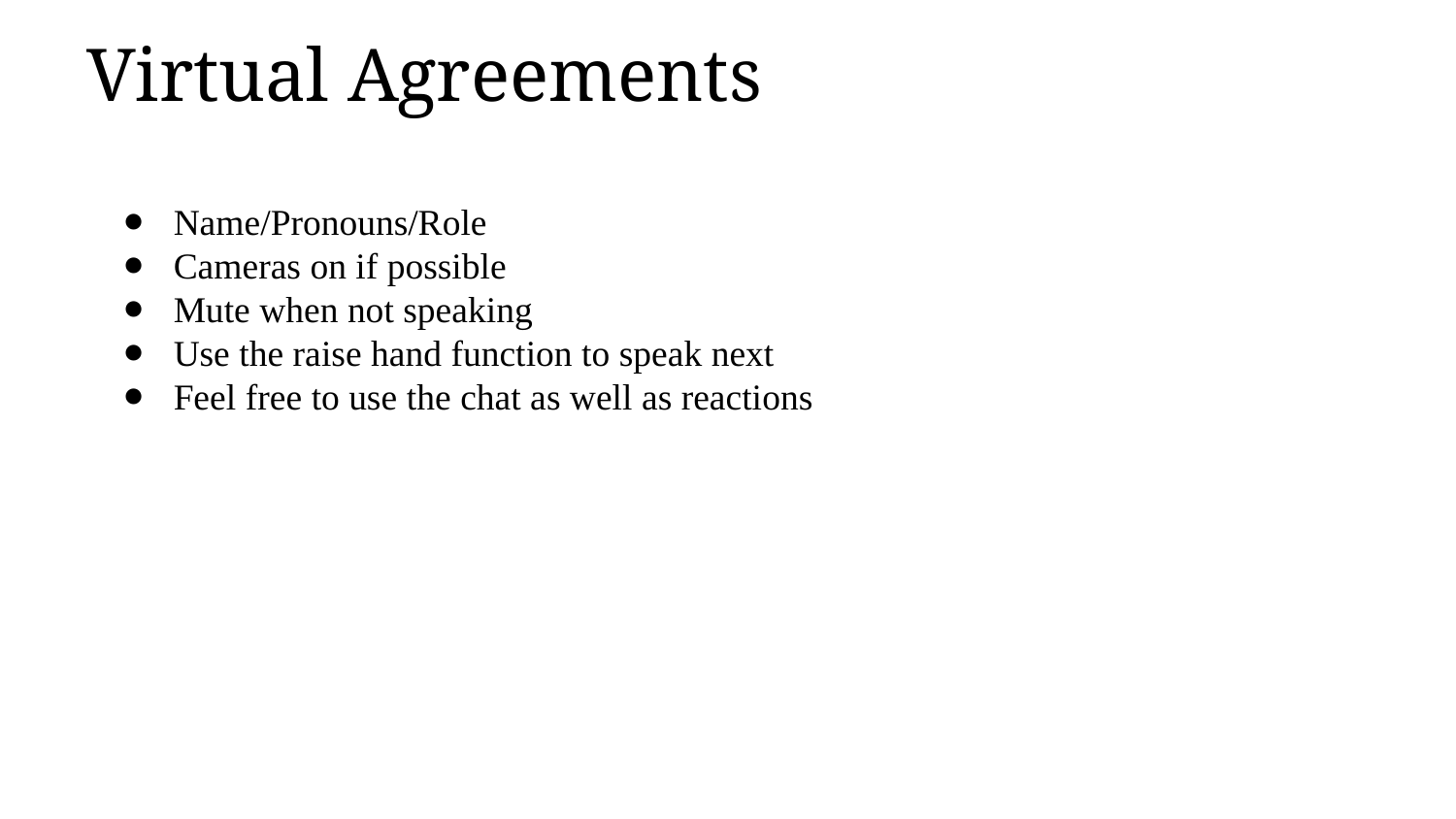

# Virtual Agreements
Name/Pronouns/Role
Cameras on if possible
Mute when not speaking
Use the raise hand function to speak next
Feel free to use the chat as well as reactions

## Slide 5
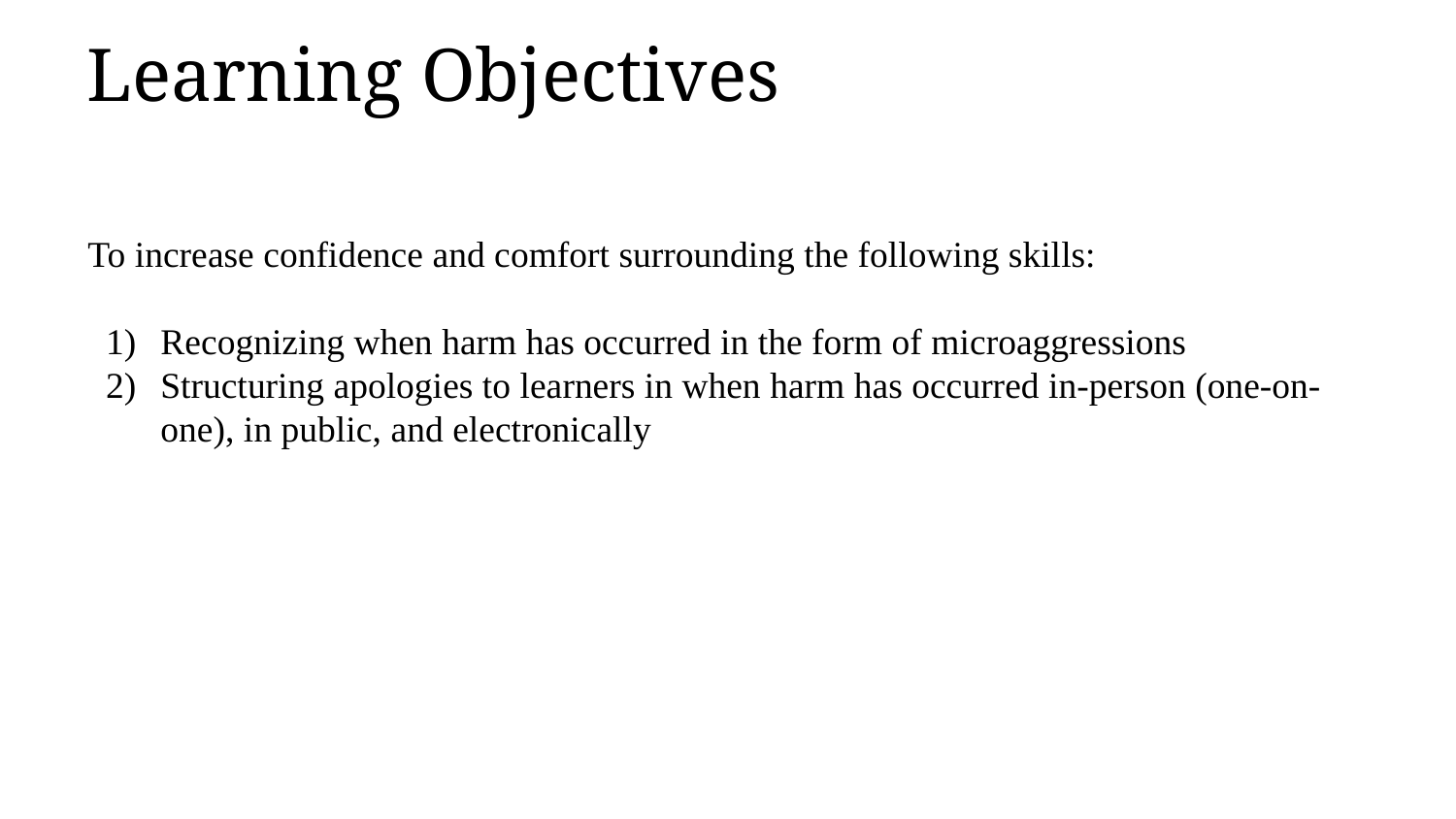

# Learning Objectives
To increase confidence and comfort surrounding the following skills:
Recognizing when harm has occurred in the form of microaggressions
Structuring apologies to learners in when harm has occurred in-person (one-on-one), in public, and electronically

## Slide 6
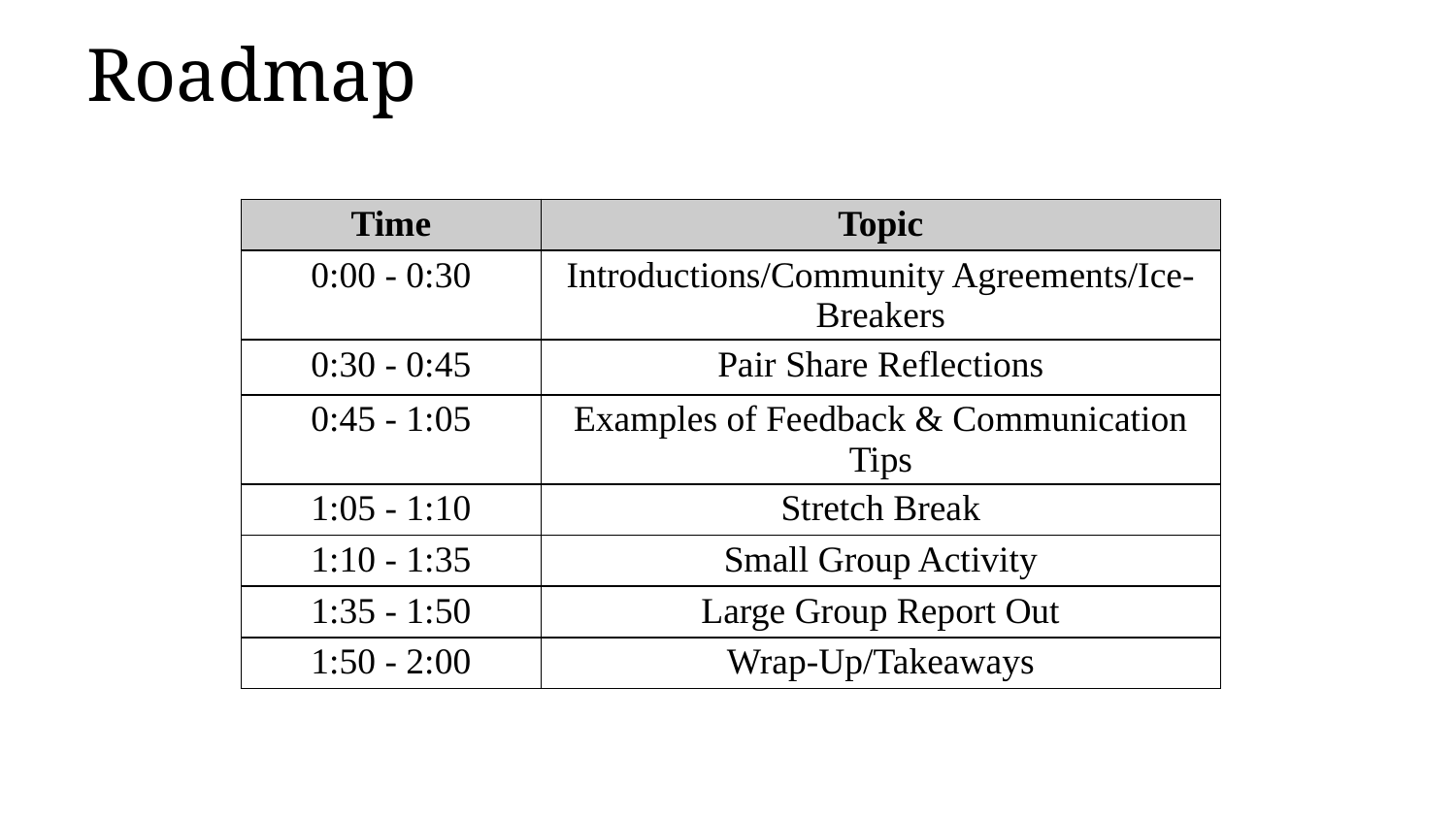

# Roadmap
| Time | Topic |
| --- | --- |
| 0:00 - 0:30 | Introductions/Community Agreements/Ice-Breakers |
| 0:30 - 0:45 | Pair Share Reflections |
| 0:45 - 1:05 | Examples of Feedback & Communication Tips |
| 1:05 - 1:10 | Stretch Break |
| 1:10 - 1:35 | Small Group Activity |
| 1:35 - 1:50 | Large Group Report Out |
| 1:50 - 2:00 | Wrap-Up/Takeaways |

## Slide 7
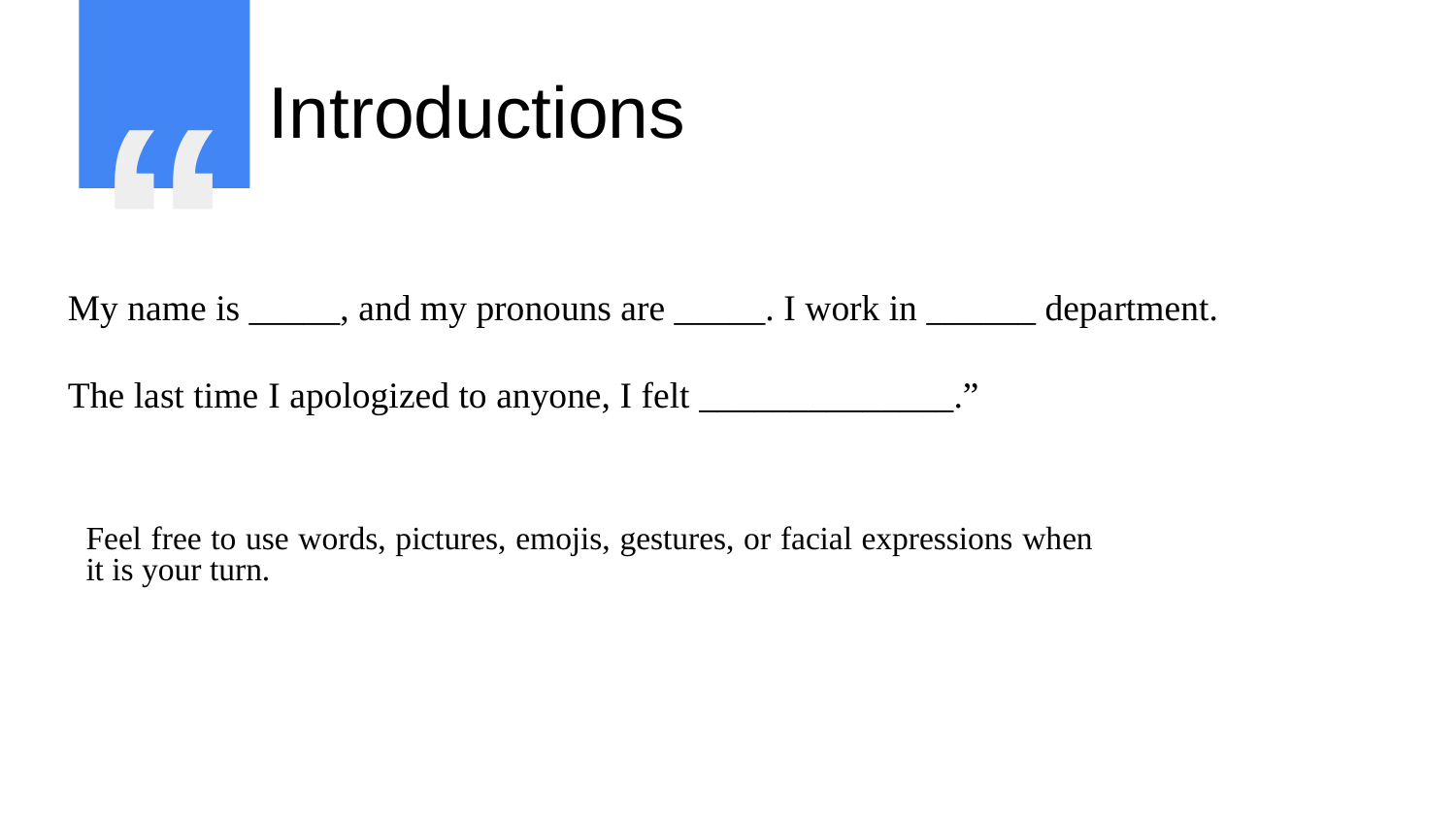

Introductions
My name is _____, and my pronouns are _____. I work in ______ department.
The last time I apologized to anyone, I felt ______________.”
Feel free to use words, pictures, emojis, gestures, or facial expressions when it is your turn.

## Slide 8
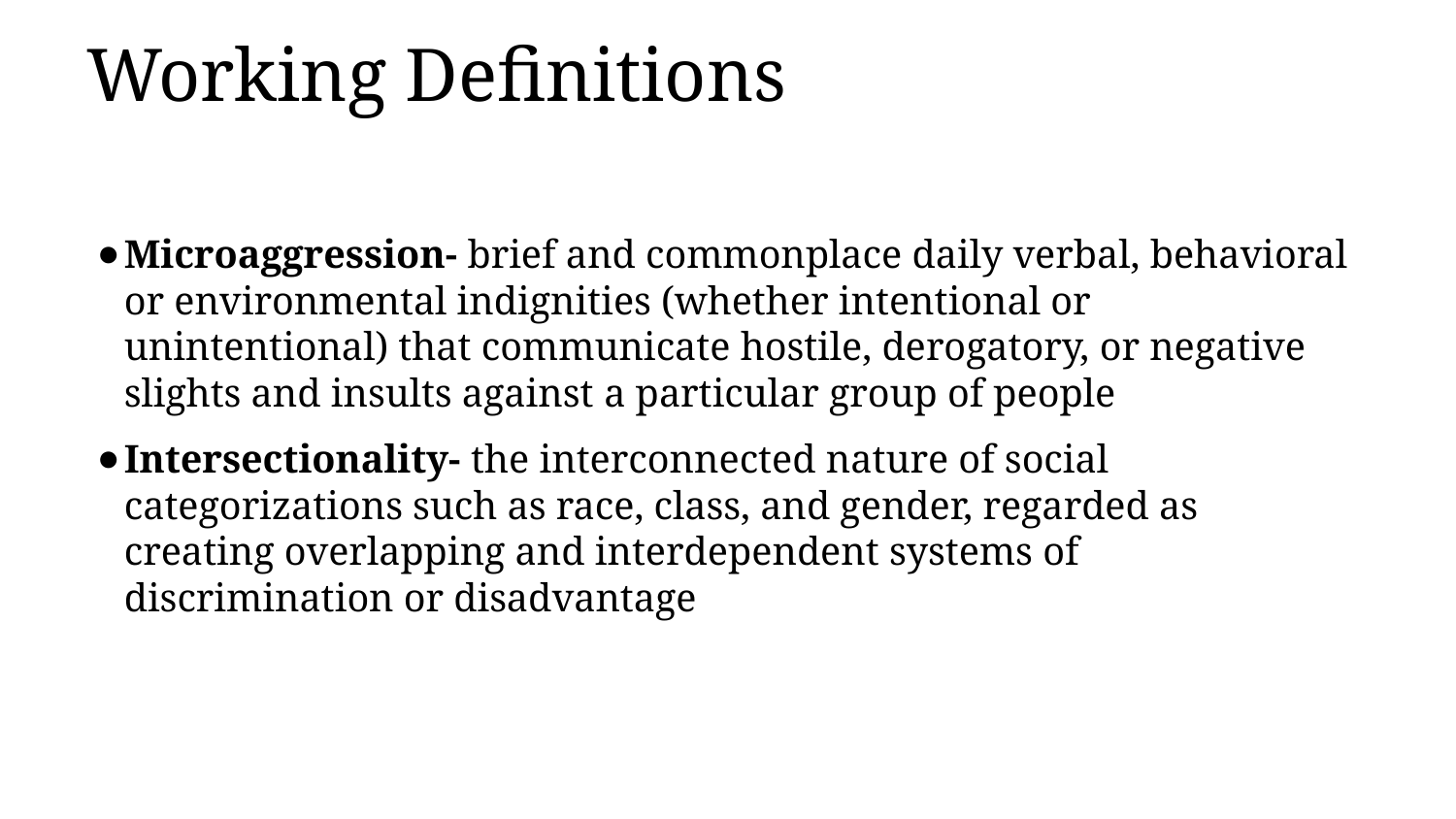

# Working Definitions
Microaggression- brief and commonplace daily verbal, behavioral or environmental indignities (whether intentional or unintentional) that communicate hostile, derogatory, or negative slights and insults against ​a particular group of people
Intersectionality- the interconnected nature of social categorizations such as race, class, and gender, regarded as creating overlapping and interdependent systems of discrimination or disadvantage​

## Slide 9
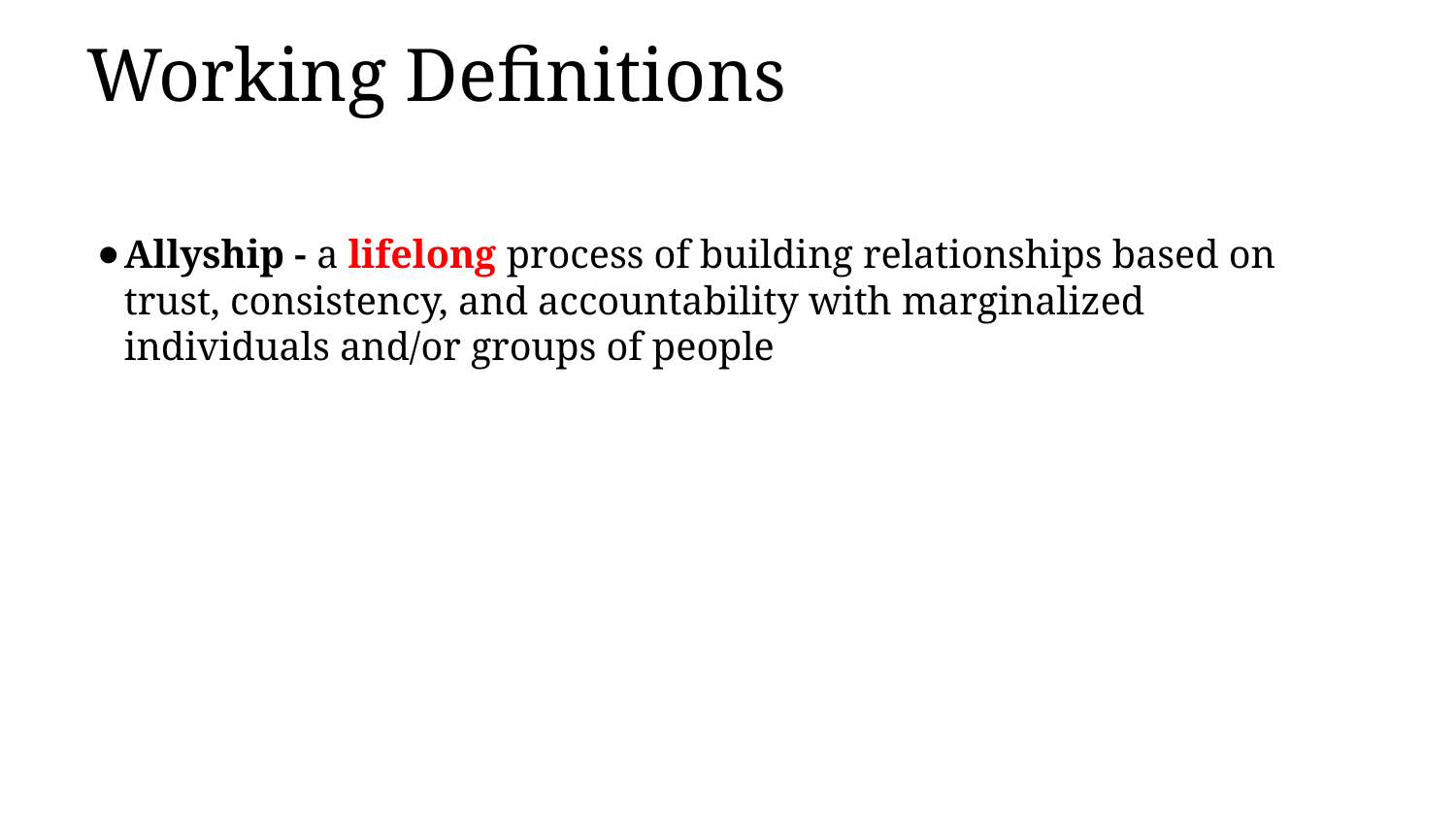

# Working Definitions
Allyship - a lifelong process of building relationships based on trust, consistency, and accountability with marginalized individuals and/or groups of people

## Slide 10
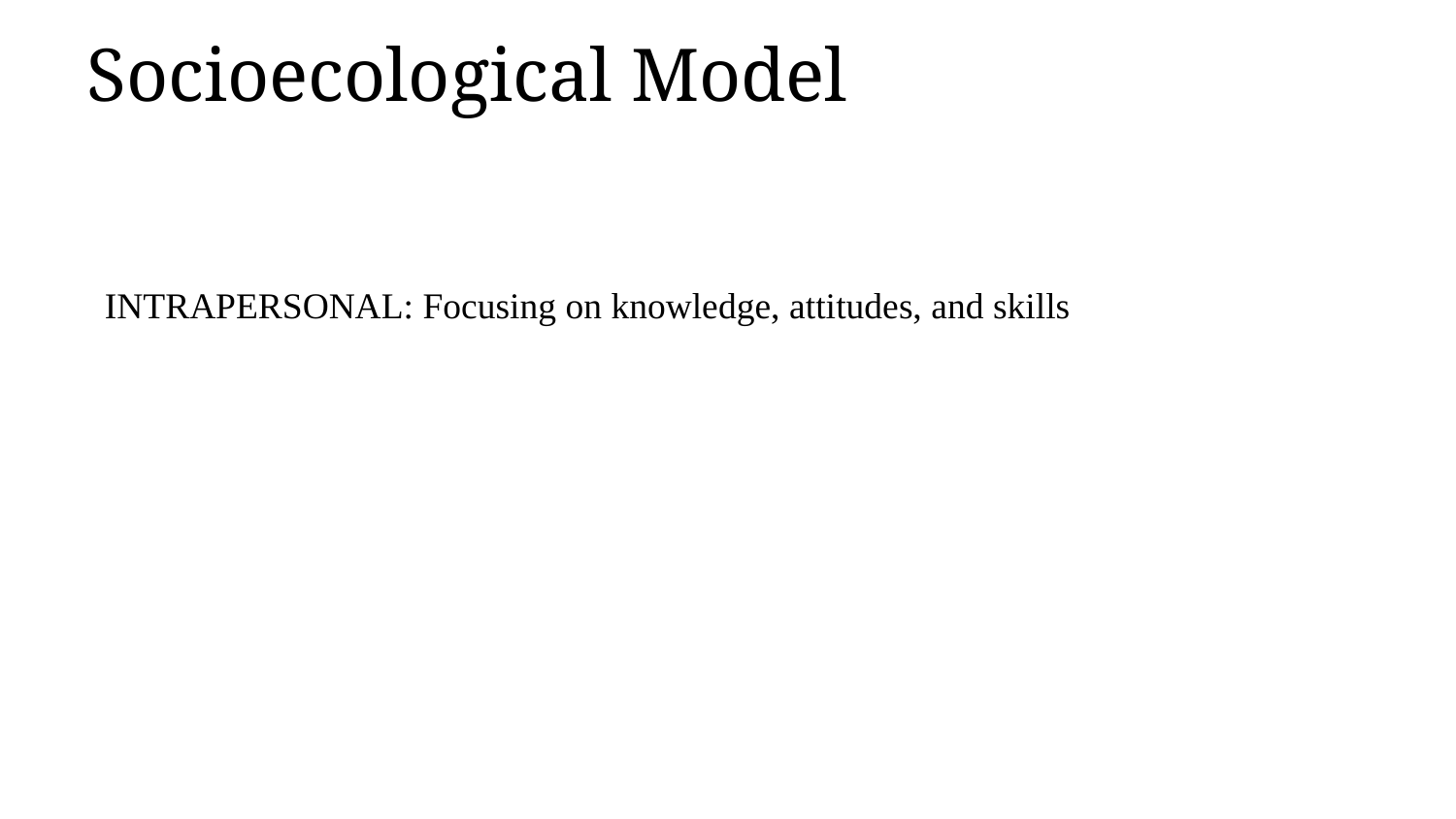

# Socioecological Model
INTRAPERSONAL: Focusing on knowledge, attitudes, and skills

## Slide 11
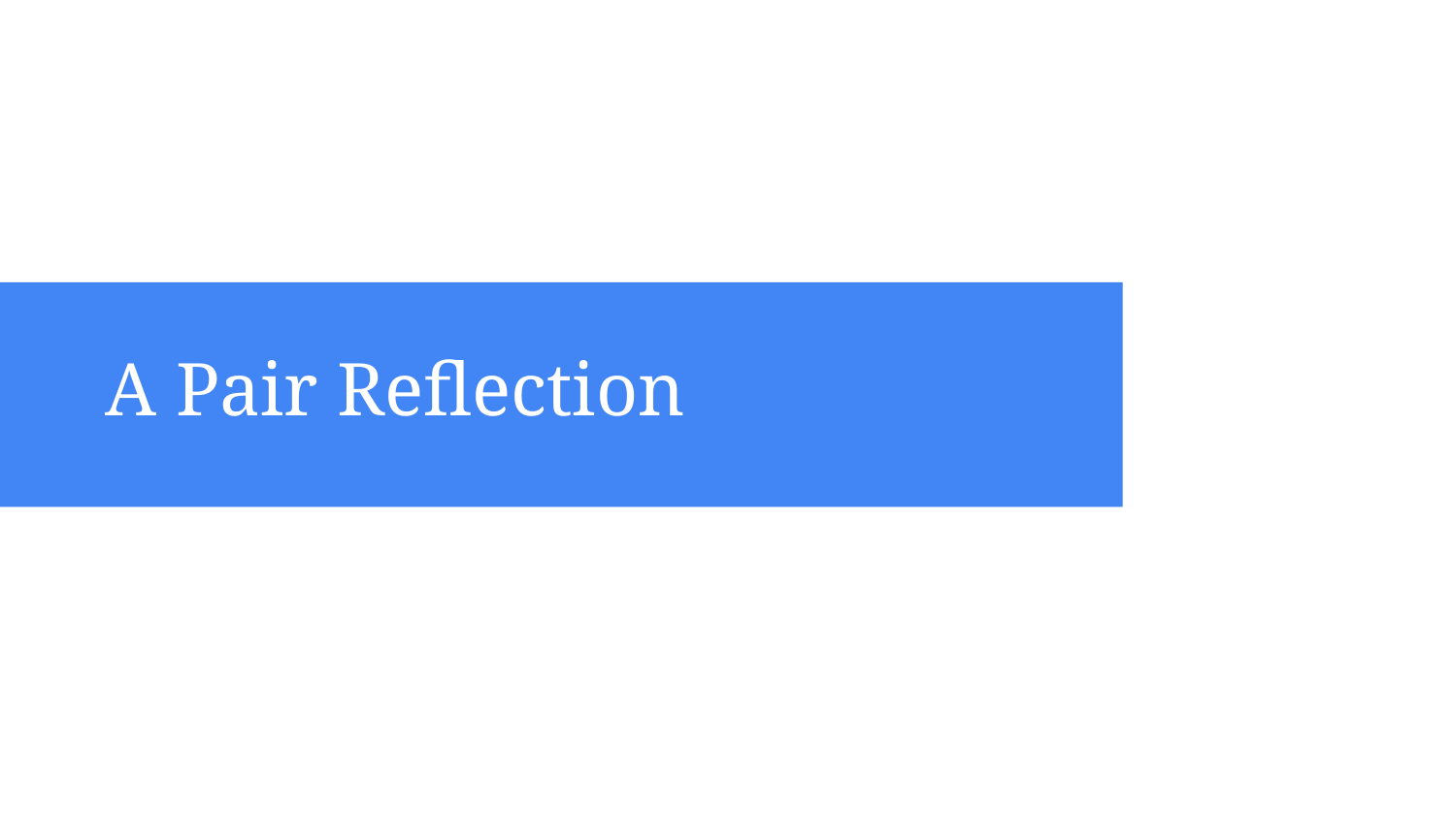

# A Pair Reflection

## Slide 12
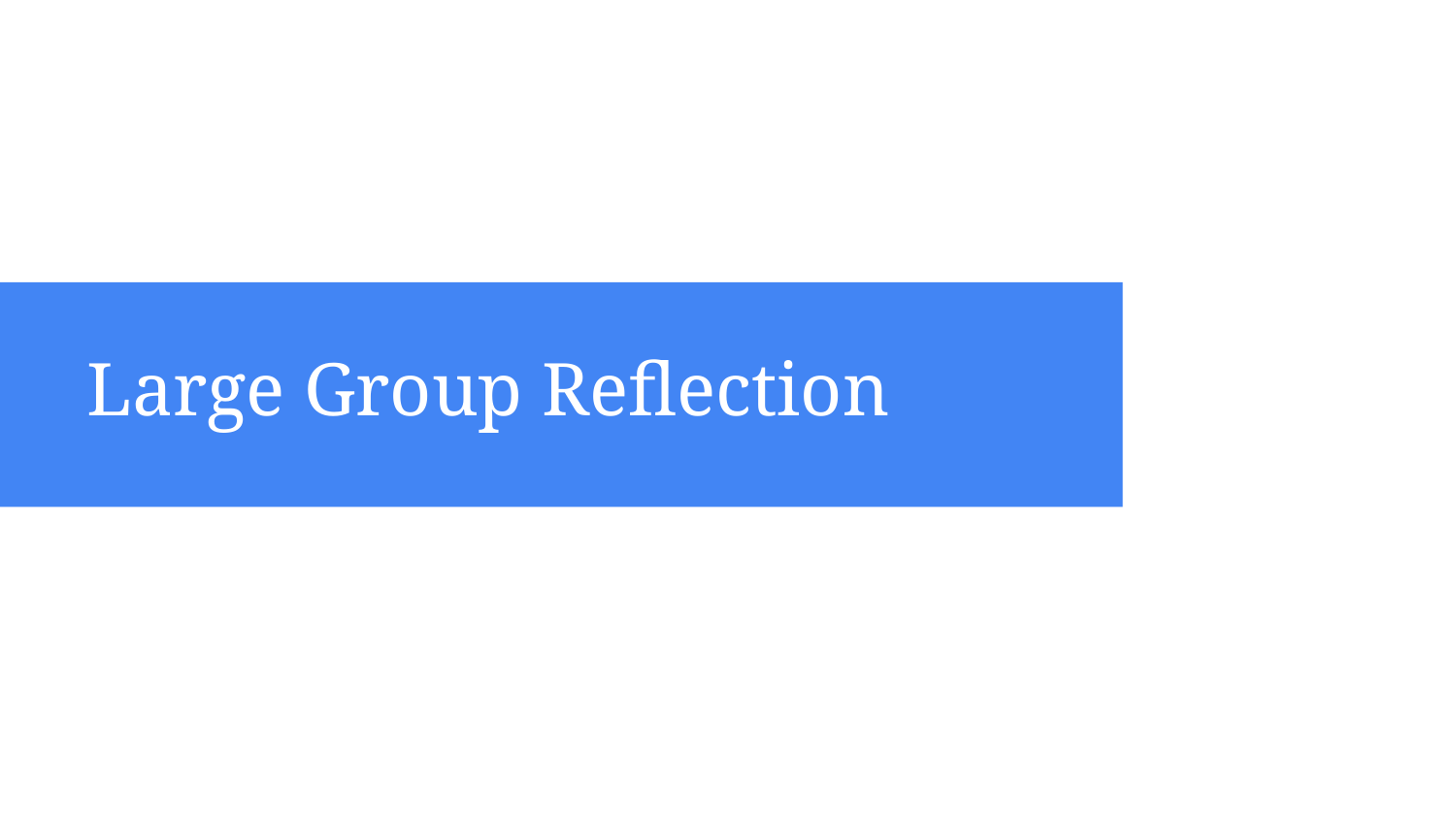

# Large Group Reflection

## Slide 13
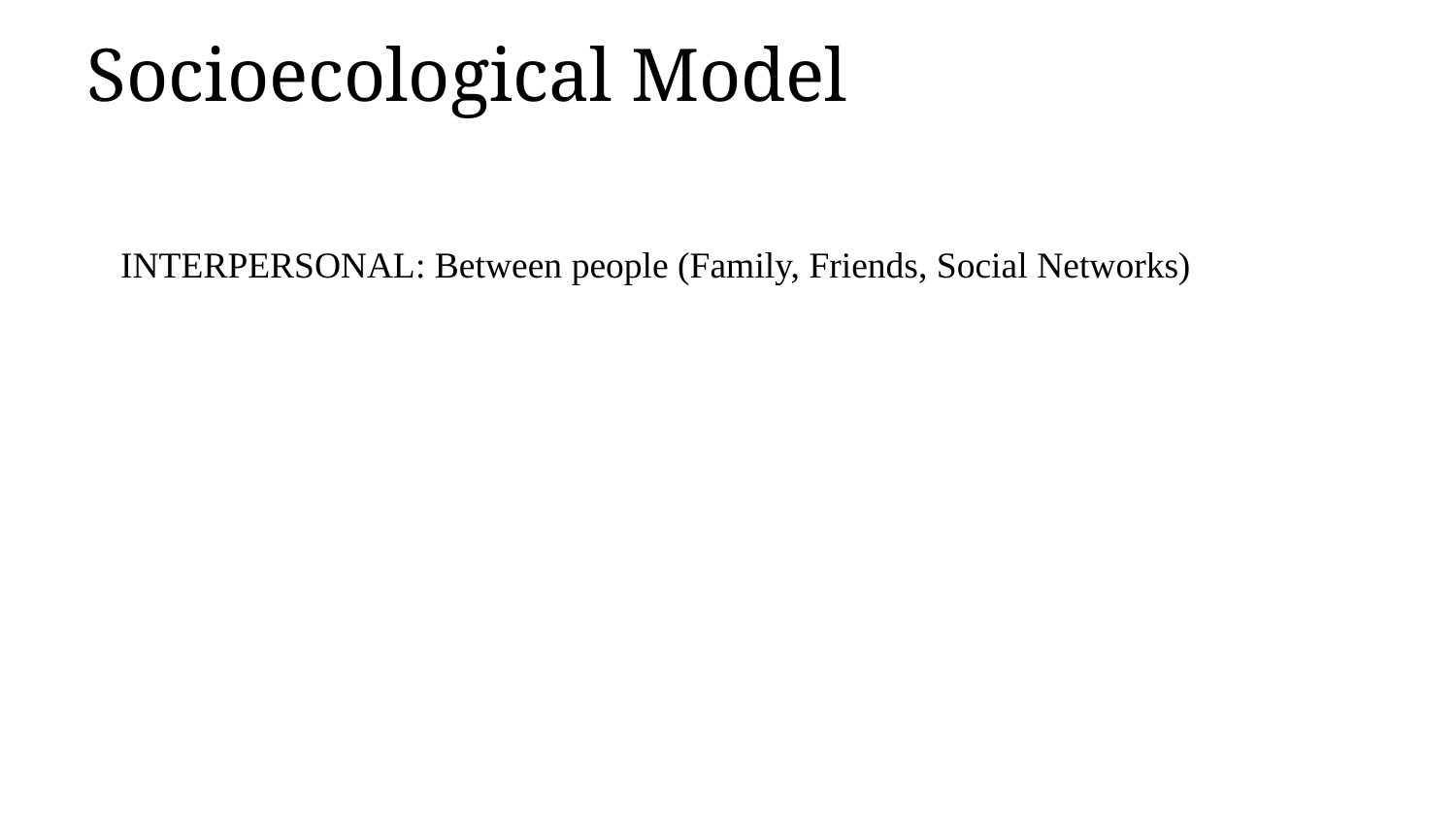

# Socioecological Model
INTERPERSONAL: Between people (Family, Friends, Social Networks)

## Slide 14
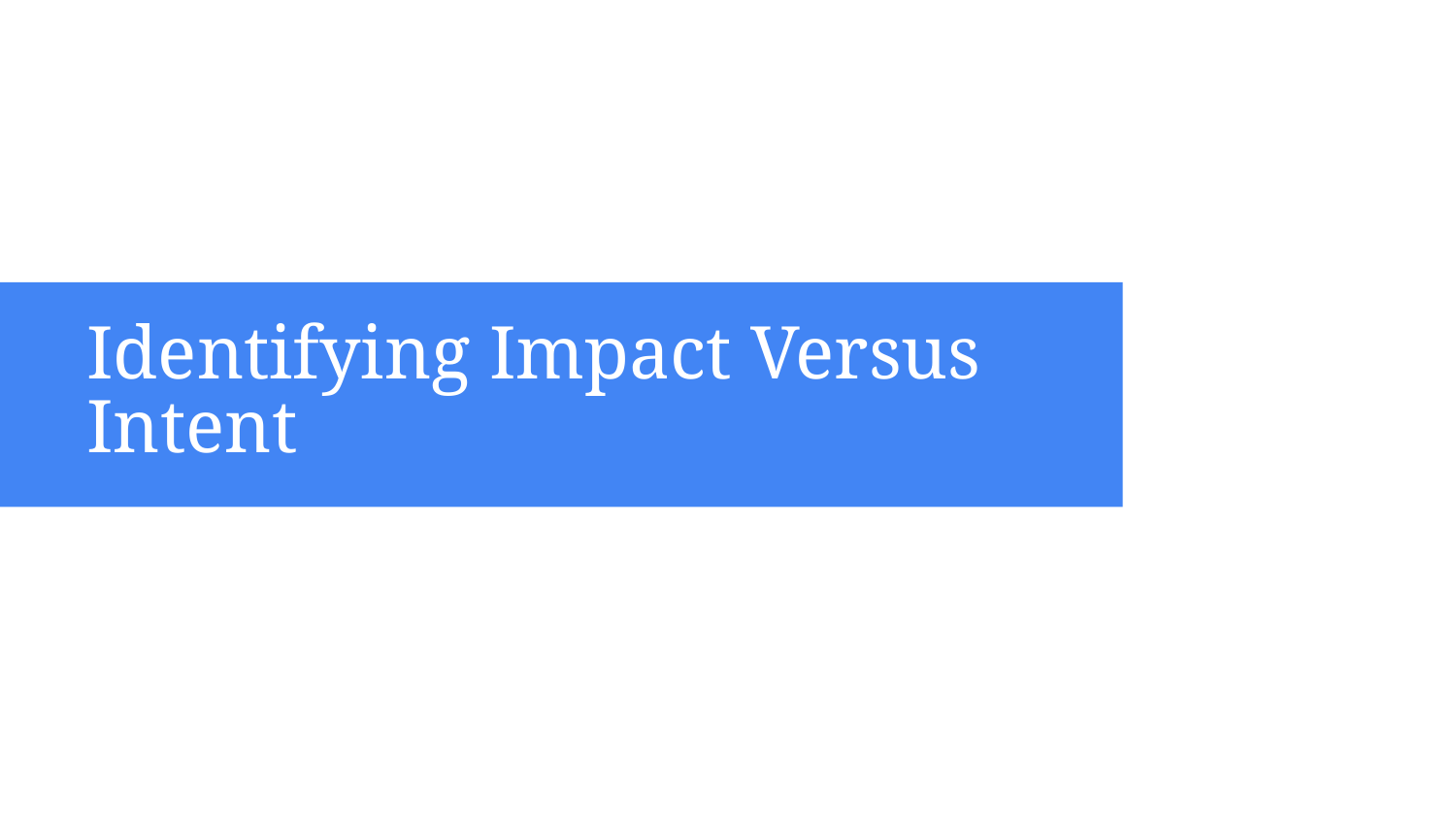

# Identifying Impact Versus Intent

## Slide 15
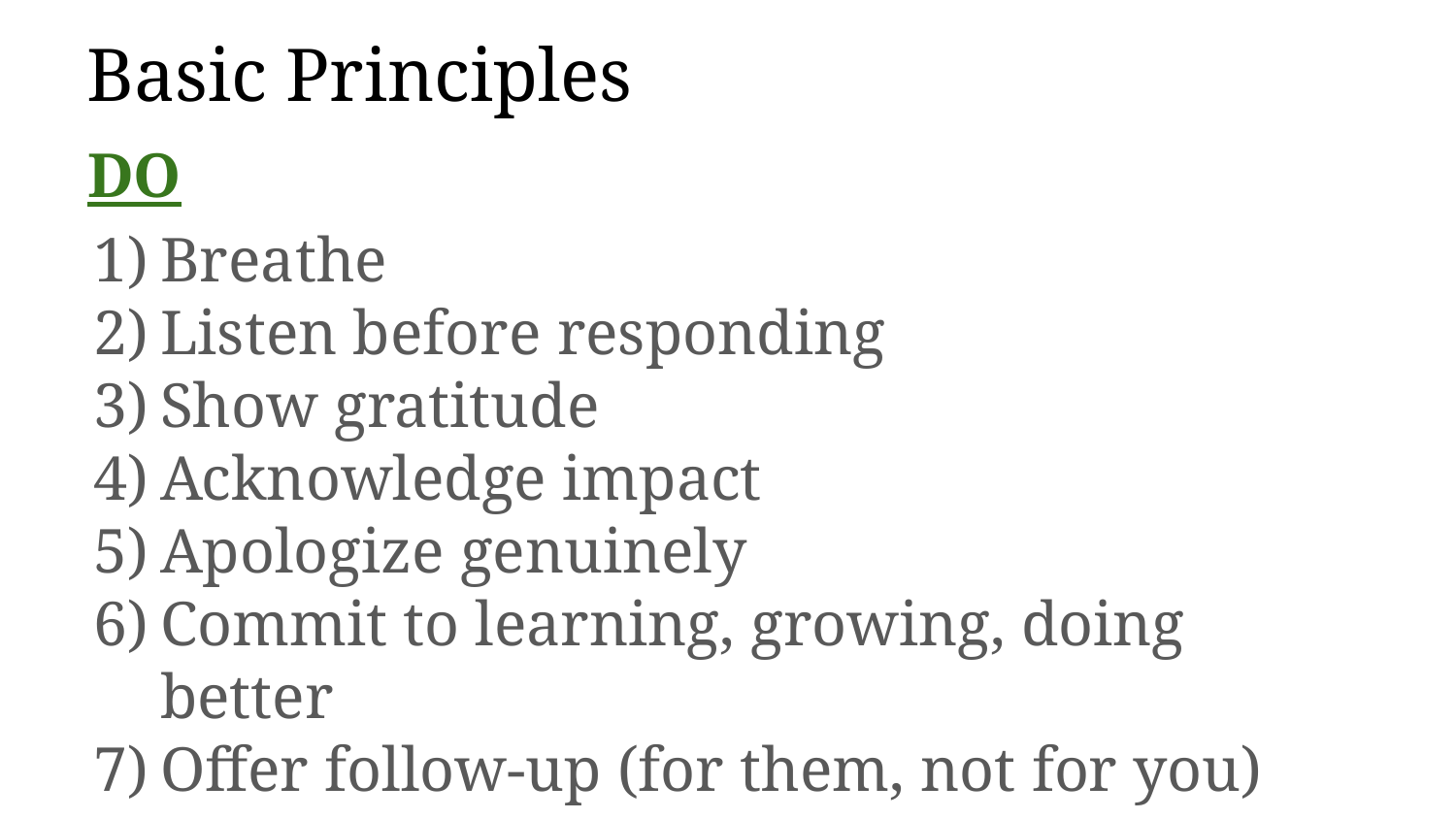

# Basic Principles
DO
Breathe
Listen before responding
Show gratitude
Acknowledge impact
Apologize genuinely
Commit to learning, growing, doing better
Offer follow-up (for them, not for you)

## Slide 16
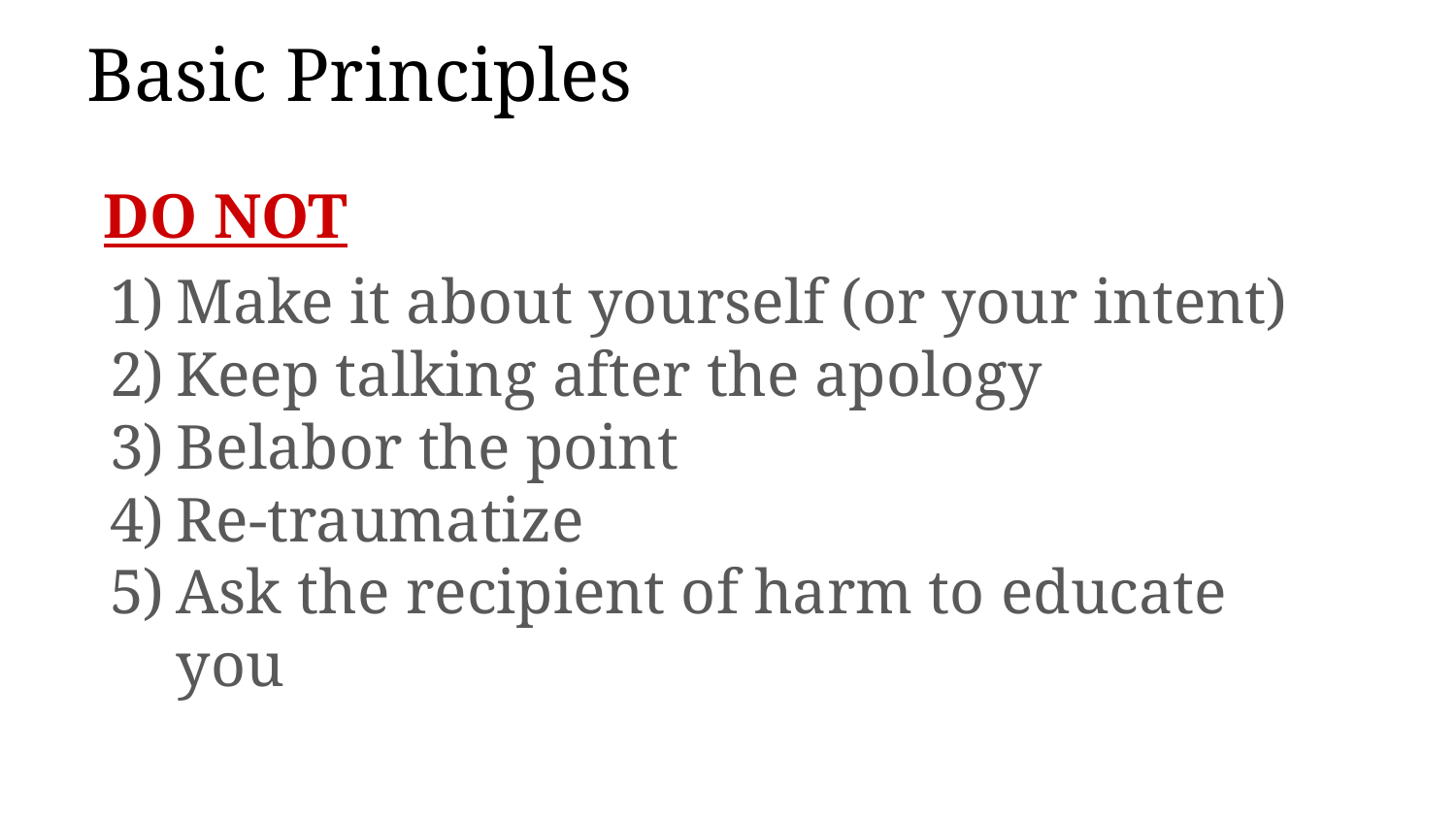

# Basic Principles
DO NOT
Make it about yourself (or your intent)
Keep talking after the apology
Belabor the point
Re-traumatize
Ask the recipient of harm to educate you

## Slide 17
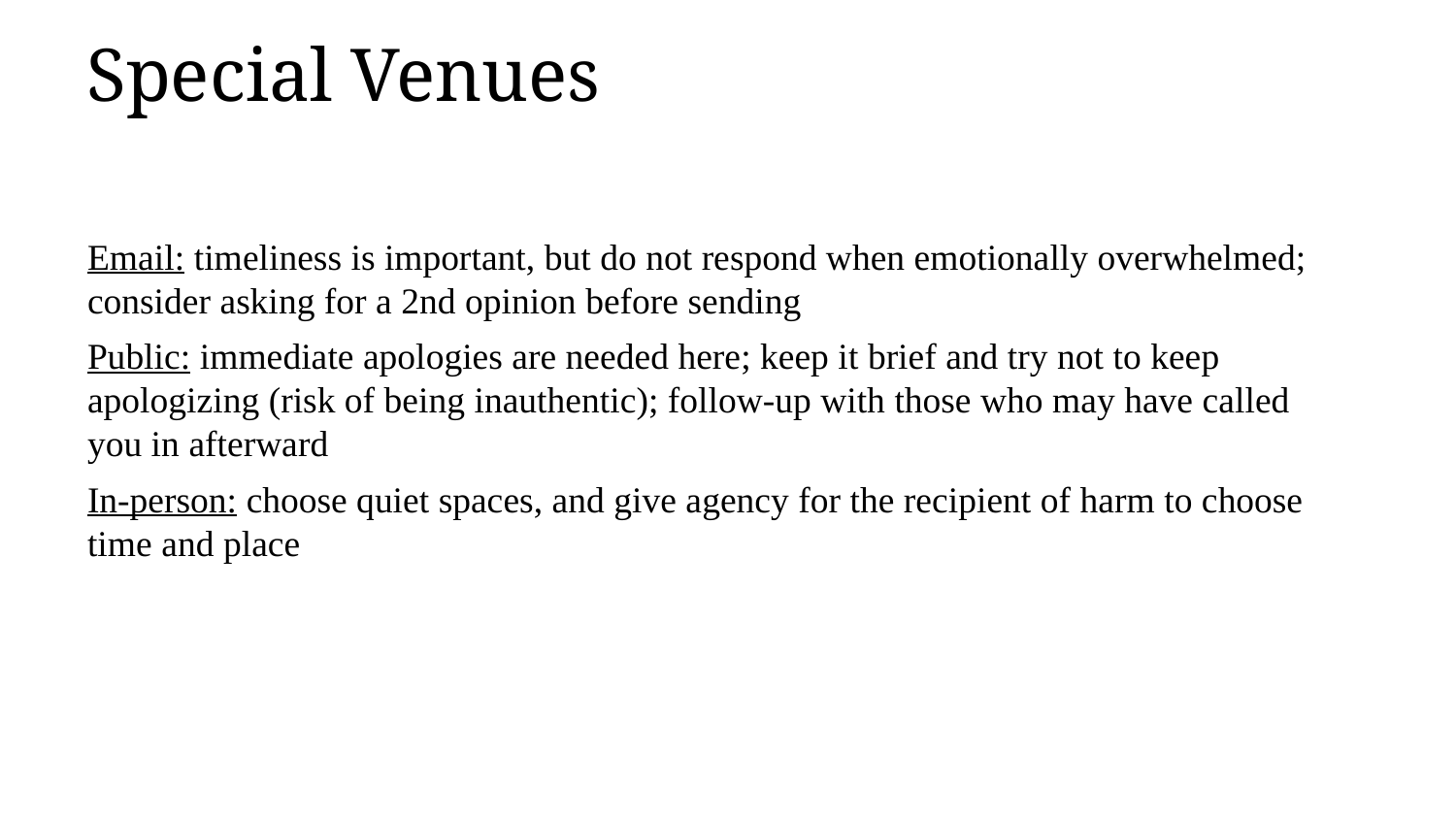

# Special Venues
Email: timeliness is important, but do not respond when emotionally overwhelmed; consider asking for a 2nd opinion before sending
Public: immediate apologies are needed here; keep it brief and try not to keep apologizing (risk of being inauthentic); follow-up with those who may have called you in afterward
In-person: choose quiet spaces, and give agency for the recipient of harm to choose time and place

## Slide 18
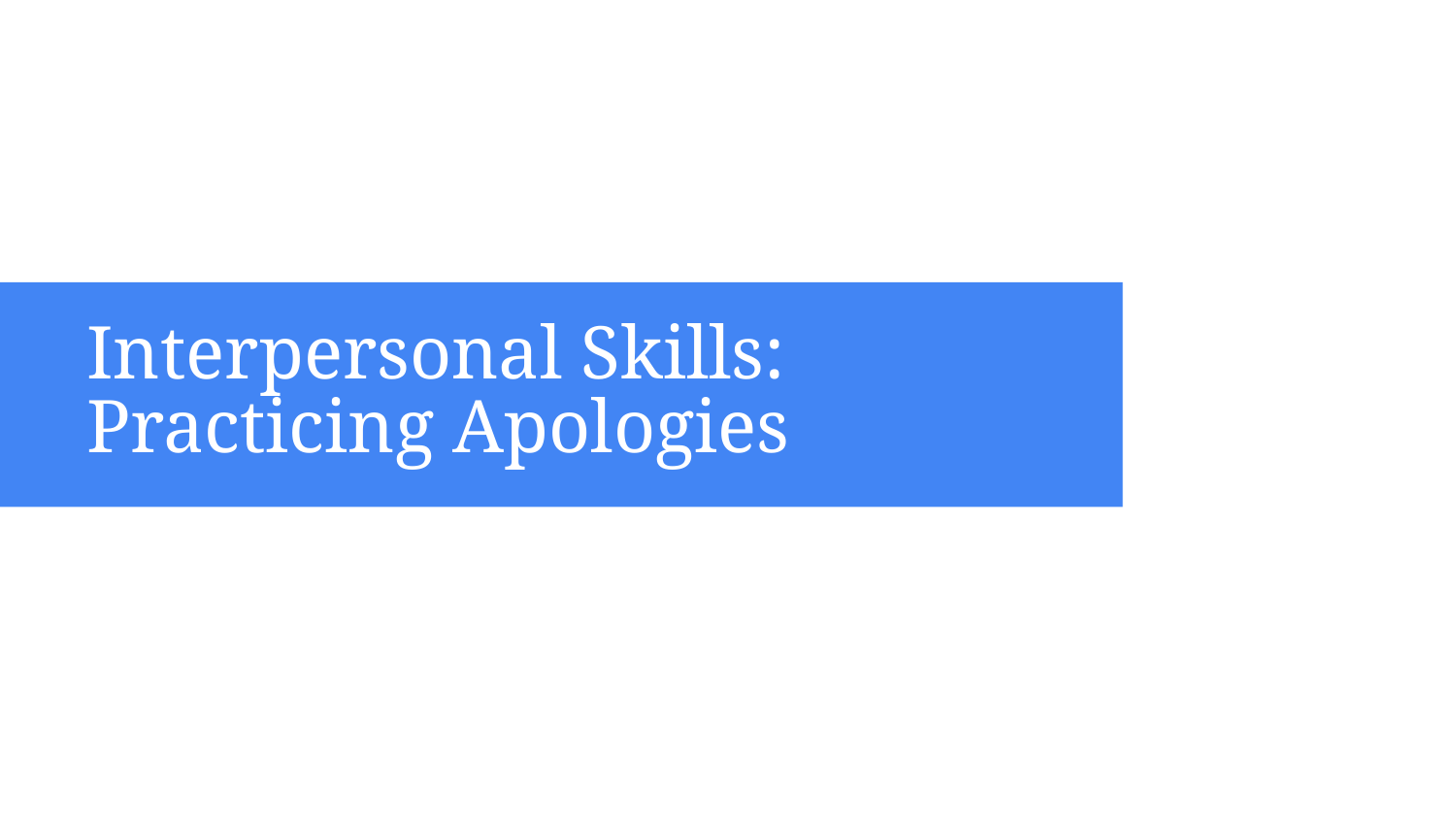

# Interpersonal Skills:
Practicing Apologies

## Slide 19
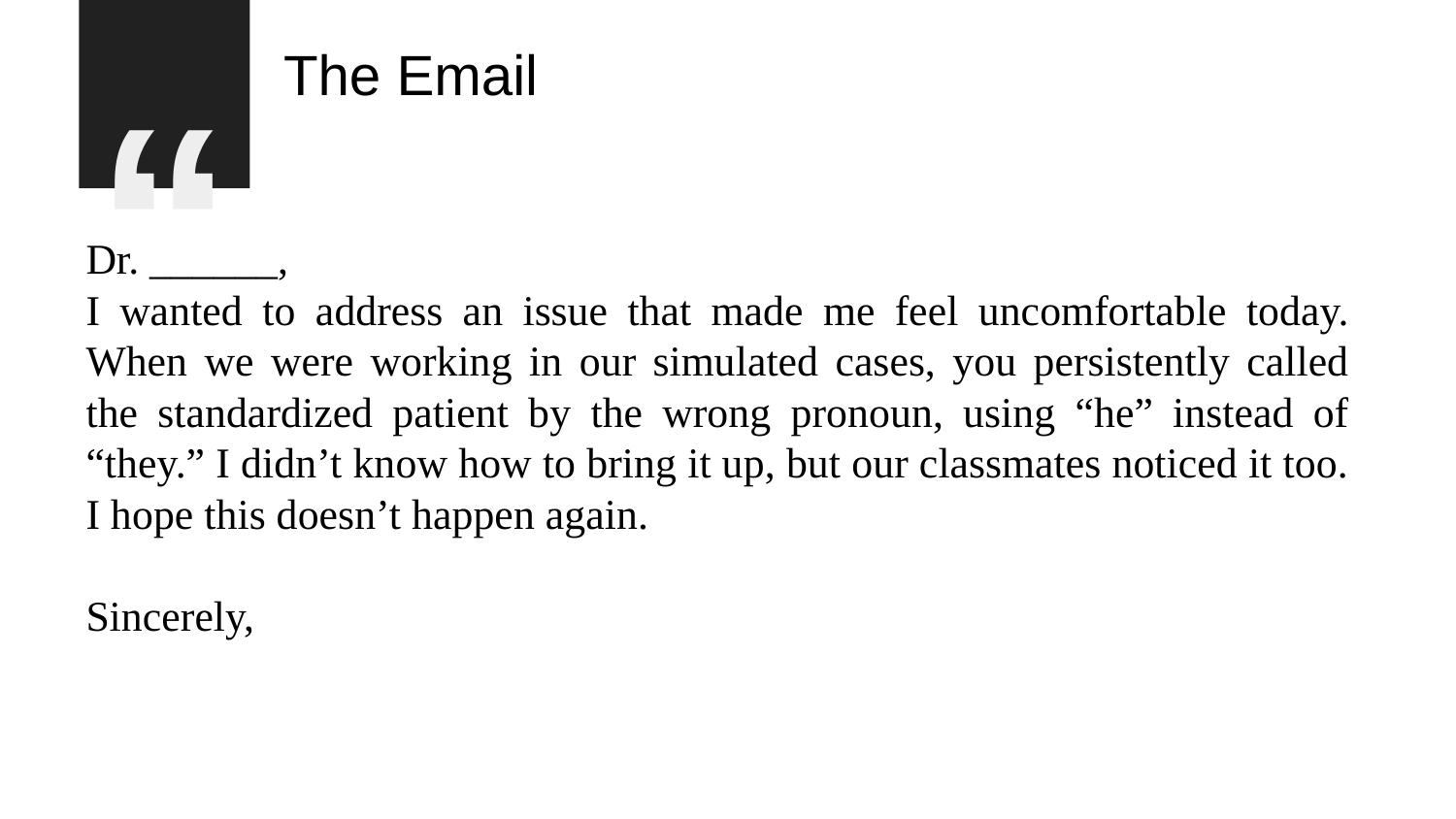

The Email
Dr. ______,
I wanted to address an issue that made me feel uncomfortable today. When we were working in our simulated cases, you persistently called the standardized patient by the wrong pronoun, using “he” instead of “they.” I didn’t know how to bring it up, but our classmates noticed it too. I hope this doesn’t happen again.
Sincerely,

## Slide 20
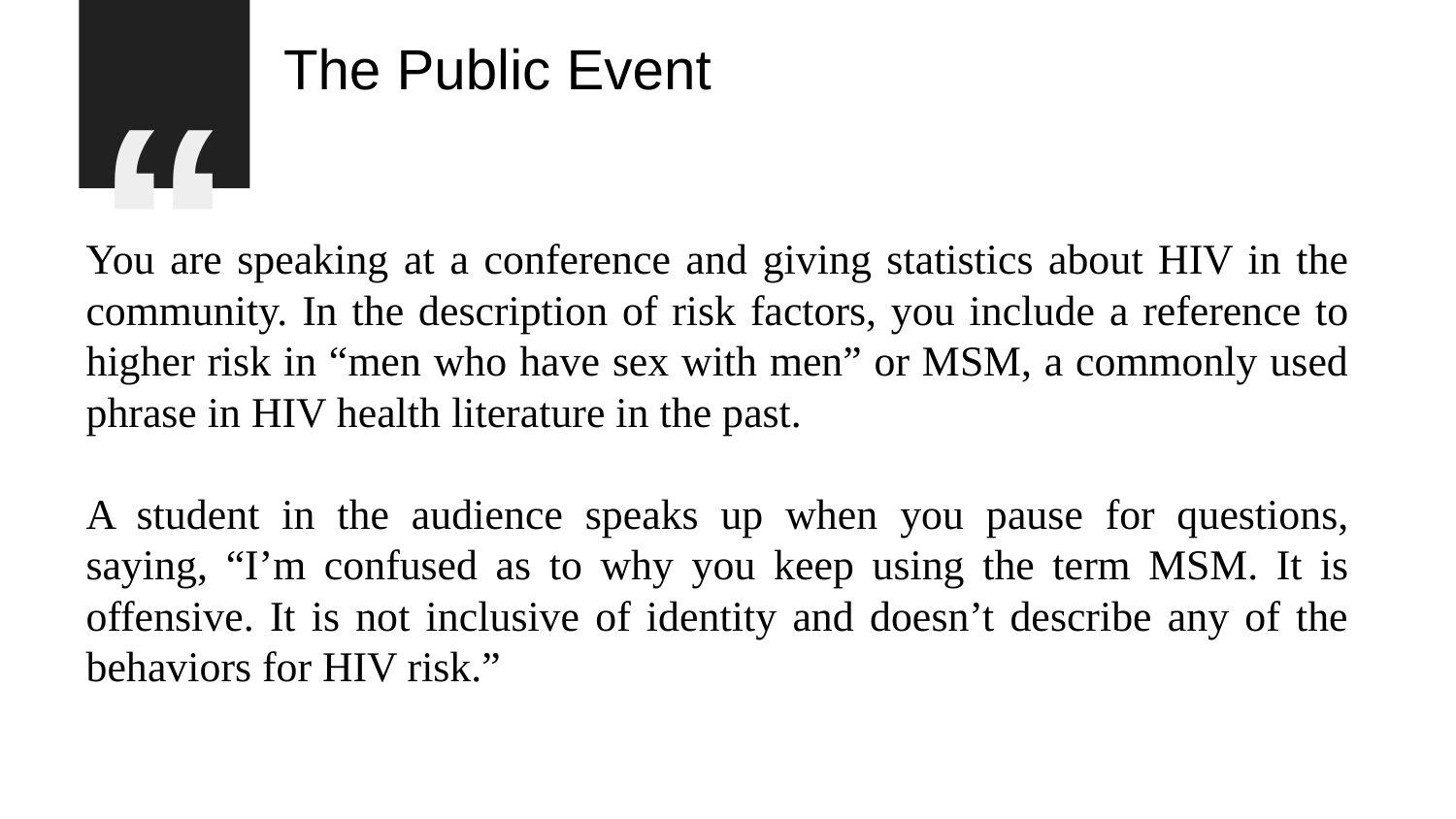

The Public Event
You are speaking at a conference and giving statistics about HIV in the community. In the description of risk factors, you include a reference to higher risk in “men who have sex with men” or MSM, a commonly used phrase in HIV health literature in the past.
A student in the audience speaks up when you pause for questions, saying, “I’m confused as to why you keep using the term MSM. It is offensive. It is not inclusive of identity and doesn’t describe any of the behaviors for HIV risk.”

## Slide 21
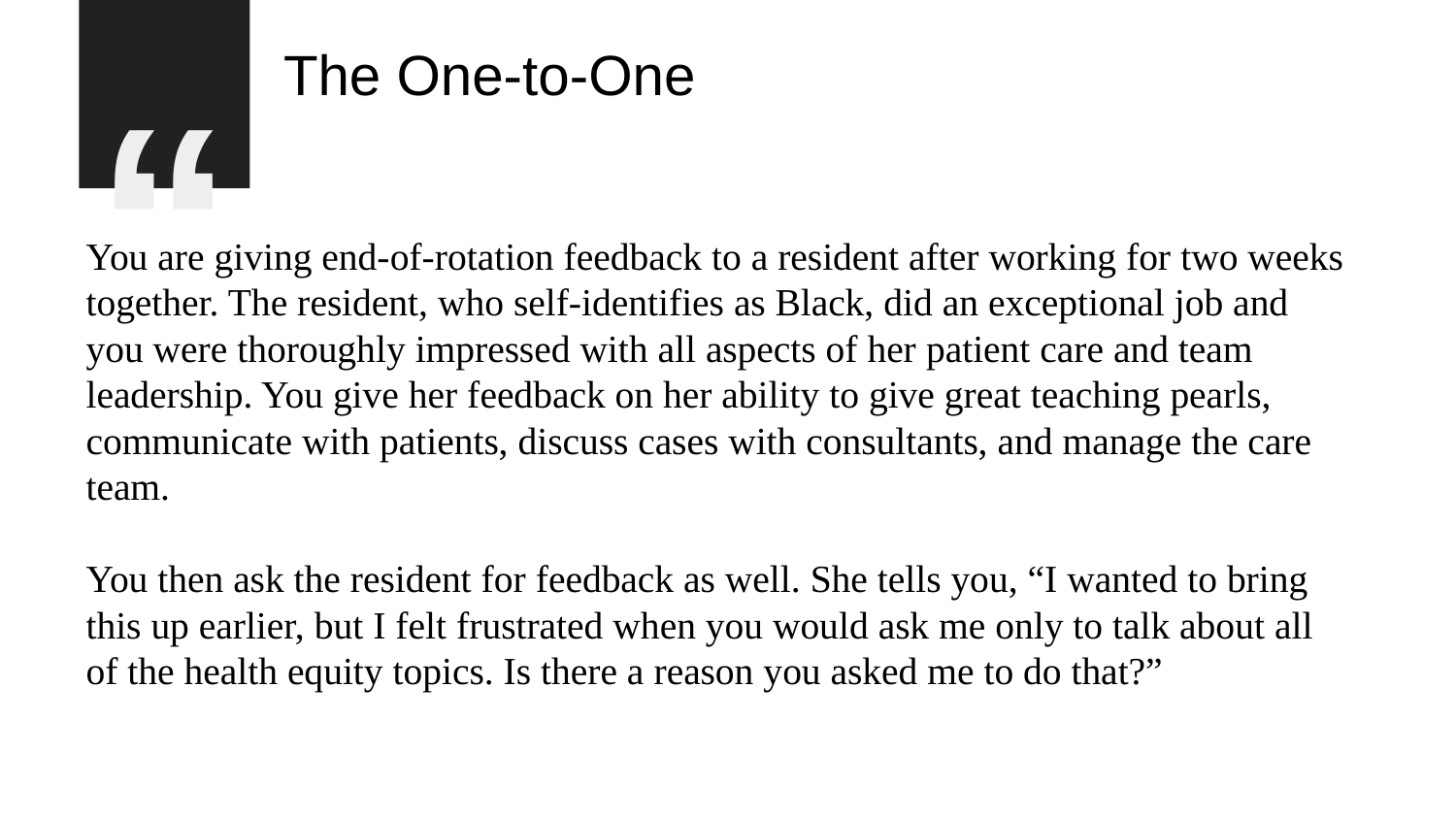

The One-to-One
You are giving end-of-rotation feedback to a resident after working for two weeks together. The resident, who self-identifies as Black, did an exceptional job and you were thoroughly impressed with all aspects of her patient care and team leadership. You give her feedback on her ability to give great teaching pearls, communicate with patients, discuss cases with consultants, and manage the care team.
You then ask the resident for feedback as well. She tells you, “I wanted to bring this up earlier, but I felt frustrated when you would ask me only to talk about all of the health equity topics. Is there a reason you asked me to do that?”

## Slide 22
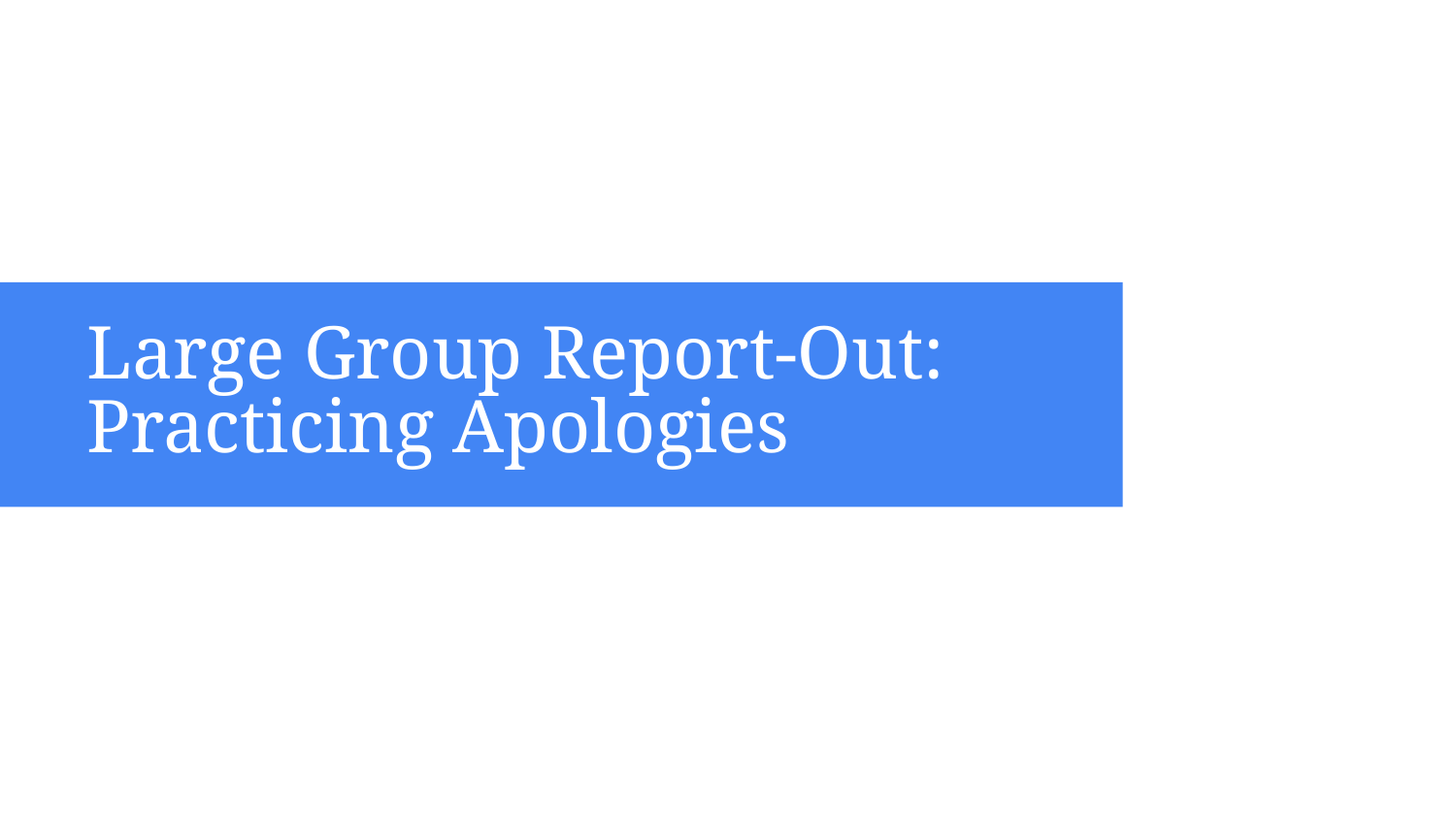

# Large Group Report-Out:
Practicing Apologies

## Slide 23
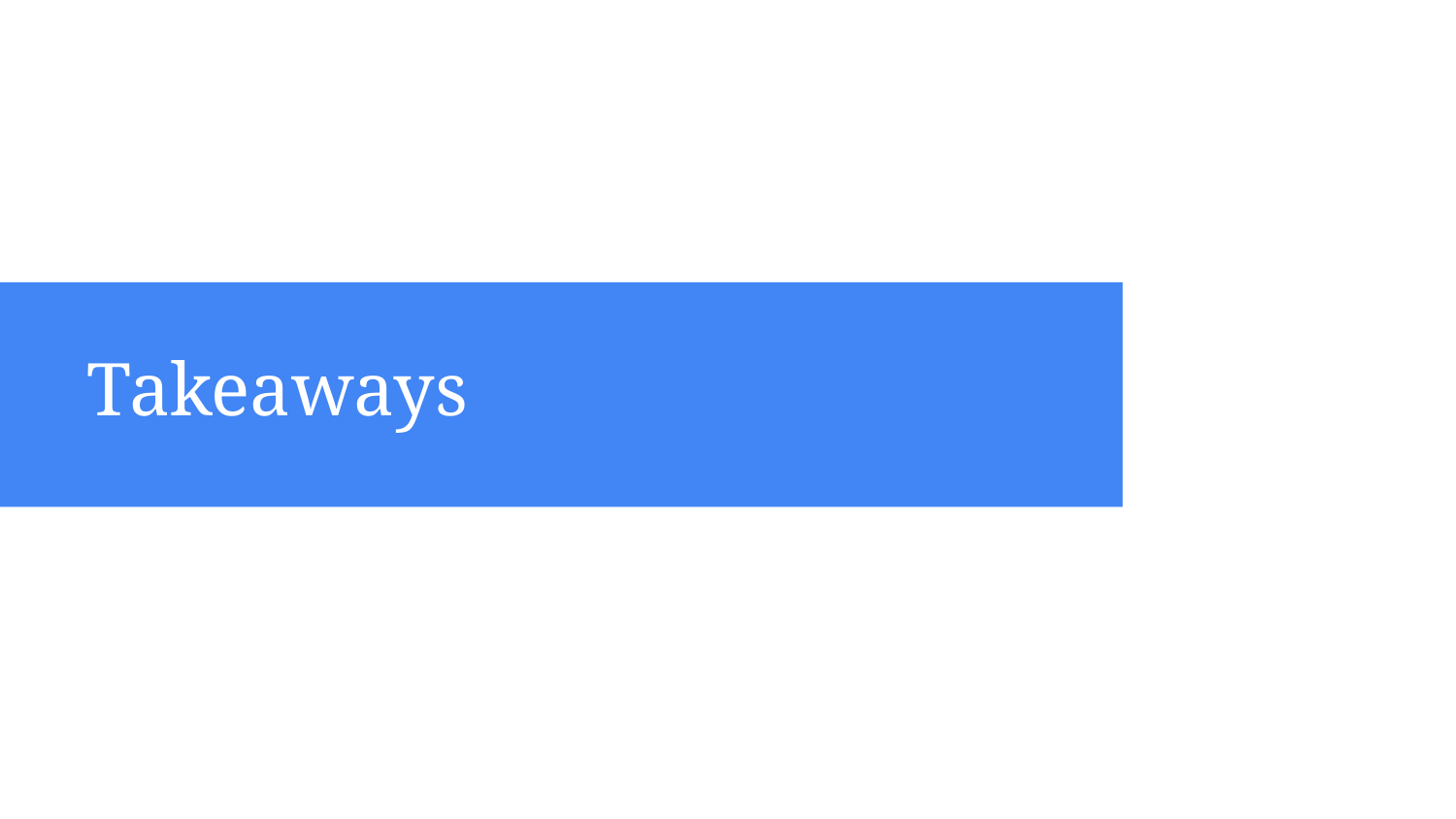

# Takeaways

## Slide 24
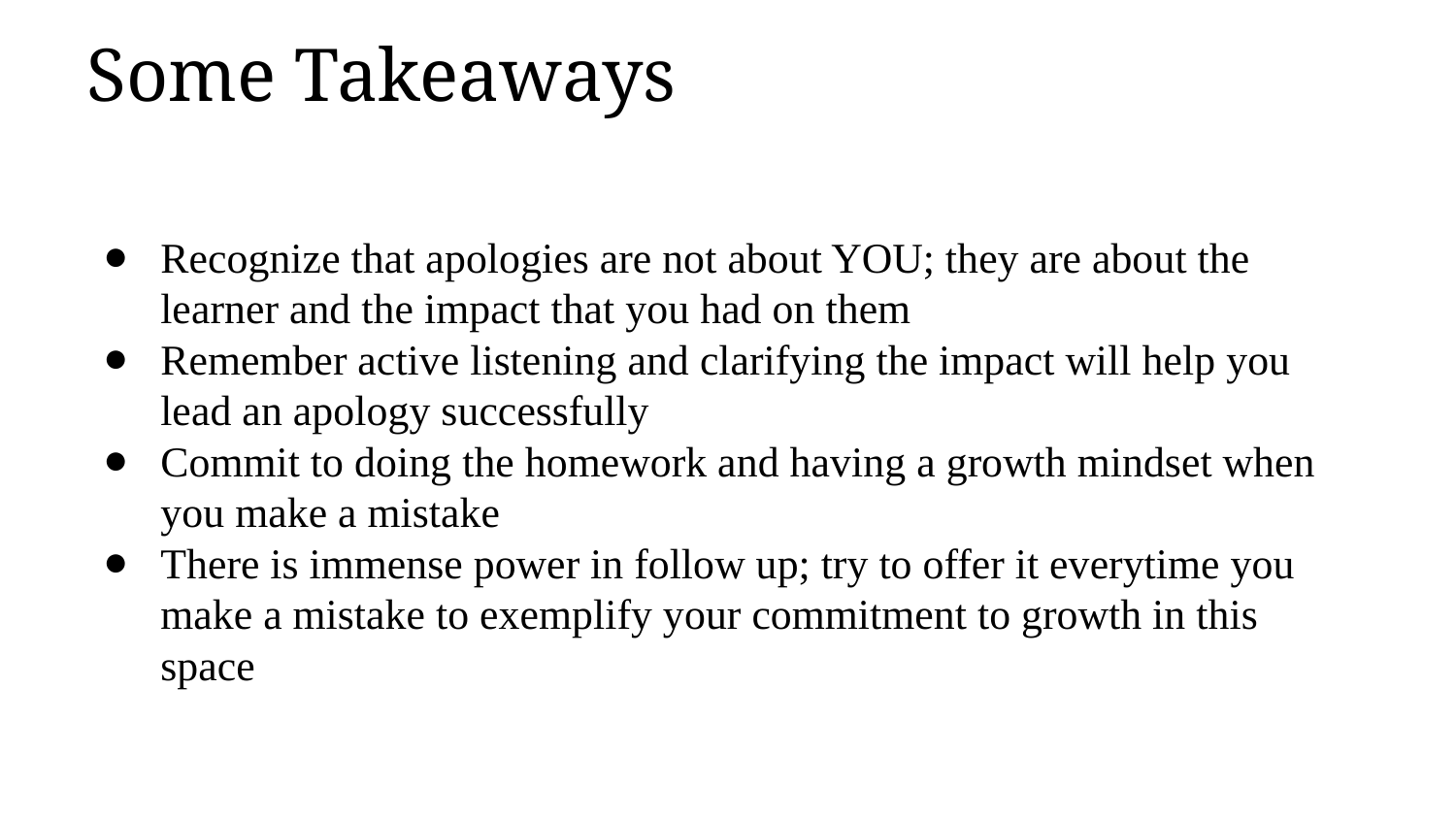

# Some Takeaways
Recognize that apologies are not about YOU; they are about the learner and the impact that you had on them
Remember active listening and clarifying the impact will help you lead an apology successfully
Commit to doing the homework and having a growth mindset when you make a mistake
There is immense power in follow up; try to offer it everytime you make a mistake to exemplify your commitment to growth in this space

## Slide 25
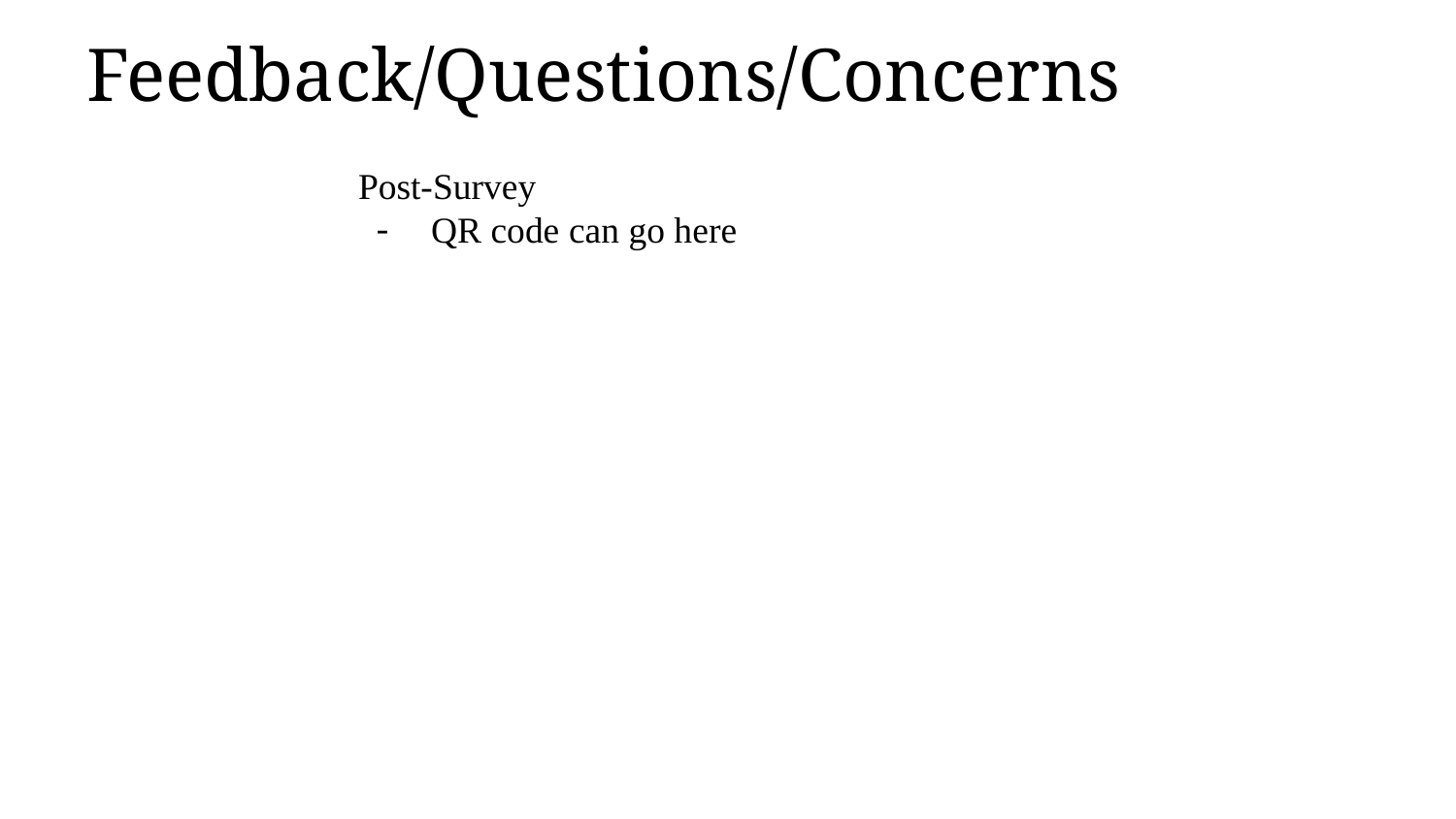

# Feedback/Questions/Concerns
Post-Survey
QR code can go here
