# Supplementary material for: Acknowledging and Addressing Microaggressions: A Virtual Experiential Learning Approach for Faculty
Source: MedEdPORTAL. 2024 Sep 4;20:11436. doi: 10.15766/mep_2374-8265.11436 (PMC11374130; doi:10.15766/mep_2374-8265.11436)
Supplement: Supplementary file 1 — Sample Flier.pptxWorkshop 1 - Slides.pptxWorkshop 1 - Facilitator GuideWorkshop 1 - Participant Handout.docxWorkshop 1 - Pre- and Postsurvey.docxWorkshop 2 - Slides.pptxWorkshop 2 - Facilitator Guide.docxWorkshop 2 - Participant Handout.docxWorkshop 2 - Pre- and Postsurvey.docxWorkshop 3 - Slides.pptxWorkshop 3 - Facilitator Guide.docxWorkshop 3 - Participant Handout.docxWorkshop 3 - Pre- and Postsurvey.docxWorkshop 4 - Slides.pptxWorkshop 4 - Facilitator Guide.docxWorkshop 4 - Participant Handout.docxWorkshop 4 - Pre- and Postsurvey.docx [file mep_2374-8265.11436-s001.zip › G. Workshop 2 - Facilitator Guide.docx]

**Faculty Microaggressions Curriculum**

**Apologizing When You’ve Done Harm**

Workshop #2

Dates:

Time: 2 hours

Where:

Lead Facilitator:

This is the second of a four-part series developed for faculty leaders in Graduate Medical education surrounding the topic of microaggressions. In order to meet learners where they are, earlier workshops will focus on learning and expanding introductory skills surrounding microaggressions, and will lead gradually to more advanced skills such as apologizing to learners when harm has been experienced, setting expectations in the learning environment, and debriefing microaggressions experienced by learners.

Today, we will focus on skills in effective apologies when one is the source of microaggression. Everyone is vulnerable to committing a microaggression. Leaders in the learning environment must own and be accountable for committing microaggressions, and have the skills to effectively communicate an apology to learners. This workshop discusses the impact of our own microaggressions on learners, and how to repair relationships once harm has been done.

**Learning Objectives:**

To increase confidence and comfort surrounding the following skills:

1. Recognizing when harm has occurred in the form of microaggressions
2. Structuring apologies to learners in when harm has occurred in-person (one-on-one), in public, and electronically

**Workshop Agenda:**

| **Time** | **Topic** | **Participants** |
| --- | --- | --- |
| 0:00 - 0:30 | Introductions/Community Agreements/Ice-Breakers | Large Group-Lead facilitator |
| 0:30 - 0:45 | Pair Share Reflections | Pair Work |
| 0:45 - 1:05 | Examples of Feedback & Communication Tips | Large Group- Lead facilitator |
| 1:05 - 1:10 | Stretch Break | -- |
| 1:10 - 1:35 | Small Group Activity | Small Group Work, all facilitators |
| 1:35 - 1:50 | Large Group Report Out | Large Group |
| 1:50 - 2:00 | Wrap-Up/Takeaways | Large Group |

**Introductions/Community Agreements/Ice Breakers (30 minutes)**

Lead facilitator for this workshop will begin the workshop with introduction of the workshop and proposed community agreements. Participants voluntarily agree to this set of operational and behavioral agreements to build trust in this learning space and engage in productive work together. Participants will be invited to add additional agreements for the working session.

Community Agreements

•Respect each other as colleagues and humans

•Confidentiality

- Stories stay within our task force meetings, and lessons may leave the room. Especially when we are talking about specific cases related to our trainees and other divisional members/leaders

•Accountability

- We hold each other accountable for our words, actions, and impact
- We hold each other accountable for adhering to our group agreements

•Use “I” statements

- Our opinions and stories are our own, and we will not make blanket statements about others

•Impact versus intent

- We will recognize the difference between IMPACT and INTENT
- We will hold ourselves and others accountable by acknowledging IMPACT of words and actions when we see/feel/hear it

•Maximize the STRETCH ZONE!

- When we do this work, we are maximally efficient in the STRETCH ZONE, a brave space where we can (and should!) be uncomfortable, yet also be productive and learn
- When we label people and shame others, it puts people in the PANIC ZONE, and decreases buy-in to continue our mission

•Ask questions and invite other perspectives continually (i.e. humble inquiry)

- Replace the idea of perfection with a growth mindset, a lifelong journey of learning, effort, and persistence

•Prioritize self-care

- We understand that there may be stories, descriptions of events, and content that may be triggering or re-traumatizing to experience
- In a community of practice, we encourage care for our own mental health, in whatever sustainable form in which that takes shape
- We aim to be present in support of each other during challenging moments

Virtual Agreements can help create guidelines for operating in a virtual learning environment as well.

•Name/Pronouns/Role

- We invite participants to re-title their virtual presence with their name, pronunciation of name, pronouns (if comfortable sharing), and role as faculty

•Cameras on if possible

- To simulate in-person interactions, we invite participants to have cameras on throughout the educational workshop
- We understand that this is not possible based on physical location, background distractions, and internet access/bandwidth

•Mute when not speaking

- To encourage active listening and one speaker talking at a time, we promote the use of the mute button when not speaking
- This also makes closed-captions for accessibility more feasible

•Use the raise hand function to speak next

- This helps facilitate one speaker at a time and participants not speaking over each other, preventing miscommunication

•Feel free to use the chat and reactions

- For those who prefer written expression to verbal expression, this is an opportunity to engage and share perspectives

** It is important to try to include the learners in establishing these agreements. One tactic is collaborating together on coming up with specific categories of norms for conversations. Examples include brainstorming together norms for topics such as how to respect others and yourself, accepting challenge and discomfort, and keeping an open mind. It is also important that each participant agrees to the set of community agreements before moving forward with the work. This can be done virtually through a reaction, a chat agreement, or gestures in the virtual room.

Tools for further ideas:

<https://radcliffe-harvard-edu-prod.s3.amazonaws.com/8b8bef3c-2b23-4771-9847-625fc015adc4/LeveragingNormsforChallengingConversationsFINAL-ua.pdf>

<https://guidetoteaching.newschool.org/community-agreements/>

Facilitators can share the learning objectives and the agenda for the workshop.

Following community agreements, each participant will have an opportunity to introduce themselves and the answer to an ice breaker:

“The last time I apologized to anyone, I felt ______________.” Feel free to use words, pictures, emojis, gestures, or facial expressions when it is your turn.

Lead facilitator will model this first to develop a brave space, and pass it on to the next facilitator/participant until all people in the workshop have shared.

A brief review of working definitions is then presented (these have all been presented in the first workshop).

Working Definitions

- **Microaggression-** brief and commonplace daily verbal, behavioral or environmental indignities (whether intentional or unintentional) that communicate hostile, derogatory, or negative slights and insults against ​a particular group of people. It is important to note that the term micro refers to interactions between individuals, not the impact on the individuals, which can be immense and feel very “macro” to recipients of microaggressions. The term was coined by Dr. Chester Pierce, a Harvard psychiatrist who became the founding president of Black Psychiatrists of America, and we pay respect to this incredible physician who brought public attention to the everyday racism faced in America in the 1960s (Williams, 2019).
- **Intersectionality-** the interconnected nature of social categorizations such as race, class, and gender, regarded as creating overlapping and interdependent systems of discrimination or disadvantage​
- **Allyship -** a lifelong process of building relationships based on trust, consistency, and accountability with marginalized individuals and/or groups of people

**Intrapersonal Exercise**

Next facilitator discusses the intrapersonal exercise. When teaching content related to diversity, equity, and inclusion (DEI), we commonly refer to the social ecological model (Golden et al, 2020) of health, understanding that health outcomes and health disparities are a result of intrapersonal attitudes and interpersonal relationships, along with the institutions, systems, culture, and policies that surround us.

**Pair Share Reflections & Pre-Work (15 mins)**

You do not have to write this down, but please spend some time thinking about this prompt.

*Think of a time when you received feedback that you had caused harm:*

*In what form was that feedback given to you?*

*What were your first thoughts and feelings as you received that feedback?*

*How did you address it?*

*What do you wish you had done differently?*

They will now spend some time sharing their answers to the reflection questions together in pairs. During the pair-share, they will begin compiling a list of DOs and DON’Ts that they have learned through their experiences.

**Feedback & Communication Tips (20 mins)**

Facilitator will ask questions to the larger group after this activity.

*How did it feel to think about these experiences?*

*What did you gain from this activity of creating DOs and DON'Ts based on your experience?*

Facilitator will then transition to some tips for communicating an apology. As a group, we will review different means by which feedback, specifically about equity and inclusivity, might be delivered. Often, it is not in-person. Some ways that feedback can be delivered include:

- By email
- By educator evaluation
- By comment during a public speaking event
- Through text message
- In person
- Through a private zoom chat
- By phone call
- Through the grapevine (from a third party)
- Via social media

Sometimes, feedback modalities can be difficult, because many do not offer a chance to reply directly in real-time. This can seem frustrating, but can also be an opportunity for you to reflect before approaching an apology. In-the-moment apologies take pause and practice to be successful. We will lay out strategies for apologizing in three different venues.

Before we talk about each of these venues, we want to emphasize the importance of understanding the difference between intent and impact. Microaggressions are often an unintended expression, however, the impact has lasting effects. We want to decentralize ourselves and our intent in this workshop. We want to apologize for the impact when we make mistakes. And we want to model humility and our commitment to lifelong learning.

Skills

**In person/One-on-one**

***DO- Breathe***

- Pause to breathe and internally reflect upon your own feelings before responding verbally to any sort of

feedback. What are you feeling? Disappointed? Sad? Angry? Embarrassed? Scared? Nervous? Frazzled? Defensive?

- You need to find your own emotional self before engaging in an apology. Understand where your thoughts

and feelings are, process them, and then move on to the topic of importance-- the learner-- which is the

person upon whom this conversation is centered.

***DO- Listen before responding***

- Active listening requires you to listen to the feedback in order to process it. ACTIVE LISTENING IS THE ONLY WAY YOU WILL BE ABLE TO IDENTIFY THE IMPACT OF YOUR BEHAVIOR VERSUS YOUR INTENT.
- Minimize all of your external (phone, computer, other people) and internal distractions. Validate what the other person is saying with gestures and words (Mmm...hmmm, I see, I hear you).
- Avoid interrupting their voice; wait until they are finished talking to have your turn.
- Make sure you summarize, paraphrase, restate impact, and ask clarifying questions without re-playing the scene.

***DO- Apologize genuinely***

- The apology must be genuine and must, again, be accountable to your own actions.

“I apologize deeply for saying something so offensive.”

“I am so sorry for the way that landed.”

“I am sorry for that impact that I had when I…”

***DO- Follow up***

- Offer to follow up with that person. If they want to have another conversation with you, make yourself available. If not, offer them your contact information.
- Remember that the follow up is not for you or to ask the learner to educate you on your mistakes. It is to

follow up on how they are feeling and whether or not there will be difficulty in building trust in your

relationship in the future.

***DO NOT- Make it about you (or your intent)***

- This is about the impact you had on somebody else and the possible harm you inflicted on another being. Whether or not you meant it does not matter. Keep it about the impact on the learner, not about what you wanted to message or hoped to come across.
- Additionally, the learner should not educate you on the problem or the historical context, this is up to you to read about and discover on your own

***DO NOT- Keep talking after the apology***

- DO NOT keep talking about your guilt or stating that you aren’t racist or biased or defending yourself. In general, replace your defensiveness with genuine curiosity and empathy

“I am committed to doing better…”

“I want you to feel included and heard on this team…”

- The more you keep talking, the more you risk re-traumatizing or triggering the recipient of harm.

**In public**

***DO- Show gratitude***

- Getting feedback in a public space can often feel embarrassing. Remembering again that this is not about you is important for getting out of the embarrassment state.
- Instead of focusing on your own feelings, approach feedback with gratitude. It must have also been difficult for an audience member to deliver that feedback in front of a group.

“Thank you for voicing this feedback.”

“I really appreciate you bringing this to my attention.”

***DO- Be brief***

- The entire presentation or talk does not now have to center on this mistake. In order to address it, and redirect back to the topic, be brief.

“I apologize for using that word. I will not be using it in the future.”

“I am sorry for the impact of my words. I will not be referring to ___ anymore.”

***DO- Commit to learning, growing, and doing Better***

- Go home and do the homework. Figure out why what you did had a negative or harmful impact. Use literature to help you understand how to do better.

***DO- Follow up personally***

- Apologizing directly to a person publicly may be perceived as performative. Instead, consider speaking to the person who gave the feedback afterward, or corresponding over email, to see if they would like to discuss more.

***DO NOT- Belabor the point***

- There is no need to continuously refer to the mistake or continuously apologize or state your intent. If there is more to say, leave room at the end of your presentation to discuss with those who may be interested.

***DO NOT- Make it about you (or your intent)***

- This is about the impact you had on somebody else and the possible harm you inflicted on another being.
- Whether or not you meant it does not matter. Keep it about the impact on the learner, not about what you wanted to message or hoped to come across.

**Electronically**

***DO- Show gratitude***

- Similar to public speaking, it is important to express gratitude to the learner for bringing up this feedback. If they didn’t bring this up, then you wouldn’t know you had a negative impact, and you wouldn’t have an opportunity for change.

***DO- State the impact***

- For clarity, state the impact in your email explicitly, the way that you understand it. This ensures that the learner got their message across and that you are perceiving your impact correctly.

***DO- State your commitment***

- As above, go home and do your homework. And explicitly state that you are committed to doing better in the future.

“I am committed to doing better.”

***DO- Offer follow up options***

- Offer to meet in person to follow up on any lingering thoughts or concerns from the learner.

***DO- Ask for a second opinion before pressing “Send”***

- It is never a bad idea to get a second opinion before sending out a message on the internet.

***DO NOT- Make it about you (or your intent)***

- This is about the impact you had on somebody else and the possible harm you inflicted

on another being.

- Whether or not you meant it does not matter. Keep it about the impact on the learner, not about what you wanted to message or hoped to come across.

***DO NOT- Respond when emotionally overwhelmed***

- Emails are considerably more difficult because now you are committing yourself to your words that can be forwarded, screen-shot, and placed on the internet for all to view.
- DO NOT respond when you are emotionally charged. Take some time to, again, reflect on your feelings and process them-- and then turn your attention to the learner.

**Small Group Synthesis (25 minutes)**

Participants will be divided into three groups (each group has a venue– email, in public, in-person) to develop an apology for three different case scenarios using the skills learned from the didactic workshop:

Email

You receive an email from a resident after a workday:

Dr. ______,

I wanted to address an issue that made me feel uncomfortable today. When we were working in our simulated cases, you persistently called the standardized patient by the wrong pronoun, using “he” instead of “they.” I didn’t know how to bring it up, but our classmates noticed it too. I hope this doesn’t happen again.

Sincerely,

As a group, draft a template email to respond to this learner and email it out to your group members to save as a reminder for the future. Each group member can personalize the template to their style.

Public Event

You are speaking at a conference and giving statistics about HIV in the community. In the description of risk factors, you include a reference to higher risk in “men who have sex with men” or MSM, a commonly used phrase in HIV health literature in the past.

A student in the audience speaks up when you pause for questions, saying, “I’m confused as to why you keep using the term MSM. It is offensive. It is not inclusive of identity and doesn’t describe any of the behaviors for HIV risk.”

As a group, determine the best verbal response for your apology in a public forum and email it to the rest of the group. Before the end of group work time, each person should practice saying aloud their personal version of that script.

**Note: If people do not understand the historical context (the risk factor here is unprotected anal receptive intercourse, not the previously blaming terminology that shames the behaviors and identities of men who have sex with men) and the identification of the microaggression here, ask them to take a few minutes to pause and read about this, coming together to determine why this statement is problematic. The group can then brainstorm about how they would develop this apology and what words they would use. This is an example of modeling commitment to educating oneself.

In-Person

You are giving end-of-rotation feedback to a resident after working for two weeks together. The resident, who identifies as Black, did an exceptional job and you were thoroughly impressed with all aspects of her patient care and team leadership. You give her feedback on her ability to give great teaching pearls, communicate with patients, discuss cases with consultants, and manage the care team. You then ask the resident for feedback as well. She tells you, “I wanted to bring this up earlier, but I felt frustrated when you would ask me only to talk about all of the health equity topics. Is there a reason you asked me to do that?”

As a group, determine the best response for this feedback in-the-moment. You may consider writing it/typing it and emailing it out to the rest of your small group for reference later. Each person in the group should practice saying aloud their personal version of the apology.

**Large Group Report Out (15 minutes)**

Each group will have an opportunity to share their scenario and some key phrases in their apology, along with questions and concerns that came up while doing the small group work.

If there is additional time, consider asking:

What are some new DOs and DON’Ts you came up with as you practiced your apologies in each of these scenarios?

What was uncomfortable or challenging?

Do you see any events or experiences where these would be helpful?

How do you see yourself using these in the future?

**WRAP-UP and TAKEAWAYS (10 minutes)**

Lead facilitator will transition to wrap up from the workshop with four key points:

- Recognize that apologies are not about YOU; they are about the learner and the impact that you had on them
- Remember active listening and clarifying the impact will help you lead an apology successfully
- Commit to doing the homework and having a growth mindset when you make a mistake
- There is immense power in followup; try to offer it everytime you make a mistake to exemplify your commitment to growth in this space

Learners will have an opportunity to add to their workshop google jamboard with their own takeaways as facilitator will review summary slide.

References for home:

- PowerPoint slides
- Participant handout
- Small group work examples shared with each other

References for this workshop:

1. Polk, W., & El-Amin, A. (2016). *Leveraging Norms for Challenging Conversations*. <https://radcliffe-harvard-edu-prod.s3.amazonaws.com/8b8bef3c-2b23-4771-9847-625fc015adc4/LeveragingNormsforChallengingConversationsFINAL-ua.pdf>
2. The New School. (2020). *Community Agreements*. Guide to teaching and learning. <https://guidetoteaching.newschool.org/community-agreements/>
3. Williams, M. T. (2019). Microaggressions: Clarification, evidence, and impact. *Perspectives on Psychological Science*, *15*(1), 3–26. <https://doi.org/10.1177/1745691619827499>
4. Golden, T. L., & Wendel, M. L. (2020). Public health’s next step in advancing equity: Re-evaluating epistemological assumptions to move social determinants from theory to practice. *Frontiers in Public Health*, *8*. https://doi.org/10.3389/fpubh.2020.00131
