# Supplementary material for: Acknowledging and Addressing Microaggressions: A Virtual Experiential Learning Approach for Faculty
Source: MedEdPORTAL. 2024 Sep 4;20:11436. doi: 10.15766/mep_2374-8265.11436 (PMC11374130; doi:10.15766/mep_2374-8265.11436)
Supplement: Supplementary file 1 — Sample Flier.pptxWorkshop 1 - Slides.pptxWorkshop 1 - Facilitator GuideWorkshop 1 - Participant Handout.docxWorkshop 1 - Pre- and Postsurvey.docxWorkshop 2 - Slides.pptxWorkshop 2 - Facilitator Guide.docxWorkshop 2 - Participant Handout.docxWorkshop 2 - Pre- and Postsurvey.docxWorkshop 3 - Slides.pptxWorkshop 3 - Facilitator Guide.docxWorkshop 3 - Participant Handout.docxWorkshop 3 - Pre- and Postsurvey.docxWorkshop 4 - Slides.pptxWorkshop 4 - Facilitator Guide.docxWorkshop 4 - Participant Handout.docxWorkshop 4 - Pre- and Postsurvey.docx [file mep_2374-8265.11436-s001.zip › H. Workshop 2 - Participant Handout.docx]

**Microaggressions Curriculum**

**Apologizing When You’ve Done Harm**

**Skills Handout**

**Learning Objectives:**

To increase confidence and comfort surrounding the following skills:

1. Recognizing when harm has occurred in the form of microaggressions
2. Structuring apologies to learners in when harm has occurred in-person (one-on-one), in public, and electronically

**Self-Reflection for Participants:**

Self-reflection. You do not have to write this down, but please spend some time thinking about this prompt.

*In what form was that feedback given to you?*

*What were your first thoughts and feelings as you received that feedback?*

*How did you address it?*

*What were you proud of in your reaction?*

*What do you wish you had done differently?*

On a separate sheet of paper, start a list of DOs and DON’Ts of apologizing based on these experiences. This is an informal list to brainstorm practices related to apologies.

**Feedback & Communication Tips**

Some ways that feedback can be delivered include:

- By email
- By educator evaluation
- By comment during a public speaking event
- Through text message
- In person
- Through a private zoom chat
- By phone call
- Through the grapevine (from a third party)
- Via social media

Sometimes, feedback modalities can be difficult, because many do not offer a chance to reply directly in real-time. This can seem frustrating, but can also be an opportunity for you to reflect before approaching an apology. In-the-moment apologies take pause and practice to be successful.

Before we talk about each of these venues, we want to emphasize the importance of understanding the difference between intent and impact. Microaggressions are often an unintended expression, however, the impact has lasting effects. We want to decentralize ourselves and our intent in this workshop. We want to apologize for the impact when we make mistakes. And we want to model humility and our commitment to lifelong learning.

**In person/One-on-one**

***DO- Breathe***

- Pause to breathe and internally reflect upon your own feelings before responding verbally to any sort of

feedback. What are you feeling? Disappointed? Sad? Angry? Embarrassed? Scared? Nervous? Frazzled? Defensive?

- You need to find your own emotional self before engaging in an apology. Understand where your thoughts

and feelings are, process them, and then move on to the topic of importance-- the learner-- which is the

person upon whom this conversation is centered.

***DO- Listen before responding***

- Active listening requires you to listen to the feedback in order to process it. ACTIVE LISTENING IS THE ONLY WAY YOU WILL BE ABLE TO IDENTIFY THE IMPACT OF YOUR BEHAVIOR VERSUS YOUR INTENT.
- Minimize all of your external (phone, computer, other people) and internal distractions. Validate what the other person is saying with gestures and words (Mmm...hmmm, I see, I hear you).
- Avoid interrupting their voice; wait until they are finished talking to have your turn.
- Make sure you summarize, paraphrase, restate impact, and ask clarifying questions without re-playing the scene.

***DO- Apologize genuinely***

- The apology must be genuine and must, again, be accountable to your own actions.

“I apologize deeply for saying something so offensive.”

“I am so sorry for the way that landed.”

“I am sorry for that impact that I had when I…”

***DO- Follow up***

- Offer to follow up with that person. If they want to have another conversation with you, make yourself available. If not, offer them your contact information.
- Remember that the follow up is not for you or to ask the learner to educate you on your mistakes. It is to

followup on how they are feeling and whether or not there will be difficulty in building trust in your

relationship in the future.

***DO NOT- Make it about you (or your intent)***

- This is about the impact you had on somebody else and the possible harm you inflicted on another being. Whether or not you meant it does not matter. Keep it about the impact on the learner, not about what you wanted to message or hoped to come across.
- Additionally, the learner should not educate you on the problem or the historical context, this is up to you to read about and discover on your own

***DO NOT- Keep talking after the apology***

- DO NOT keep talking about your guilt or stating that you aren’t racist or biased or defending yourself. In general, replace your defensiveness with genuine curiosity and empathy

“I am committed to doing better…”

“I want you to feel included and heard on this team…”

- The more you keep talking, the more you risk re-traumatizing or triggering the recipient of harm.

---------------------------------------------------------------------------------------------------------------------

**CASE SCENARIO (IN-PERSON):**

You are giving end-of-rotation feedback to a resident after working for two weeks together. The resident, who identifies as Black, did an exceptional job and you were thoroughly impressed with all aspects of her patient care and team leadership. You give her feedback on her ability to give great teaching pearls, communicate with patients, discuss cases with consultants, and manage the care team. You then ask the resident for feedback as well.

She tells you, “I wanted to bring this up earlier, but I felt frustrated when you would ask me only to talk about all of the health equity topics. Is there a reason you asked me to do that?”

As a group, determine the best response for this feedback in-the-moment. You may consider writing it/typing it and emailing it out to the rest of your small group for reference later. Each person in the group should practice saying aloud their personal version of the apology.

**In public**

***DO- Show gratitude***

- Getting feedback in a public space can often feel embarrassing. Remembering again that this is not about you is important for getting out of the embarrassment state.
- Instead of focusing on your own feelings, approach feedback with gratitude. It must have also been difficult for an audience member to deliver that feedback in front of a group.

“Thank you for voicing this feedback.”

“I really appreciate you bringing this to my attention.”

***DO- Be brief***

- The entire presentation or talk does not now have to center on this mistake. In order to address it, and redirect back to the topic, be brief.

“I apologize for using that word. I will not be using it in the future.”

“I am sorry for the impact of my words. I will not be referring to ___ anymore.”

***DO- Commit to learning, growing, and doing Better***

- Go home and do the homework. Figure out why what you did had a negative or harmful impact. Use literature to help you understand how to do better.

***DO- Follow up personally***

- Apologizing directly to a person publicly may be perceived as performative. Instead, consider speaking to the person who gave the feedback afterward, or corresponding over email, to see if they would like to discuss more.

***DO NOT- Belabor the point***

- There is no need to continuously refer to the mistake or continuously apologize or state your intent. If there is more to say, leave room at the end of your presentation to discuss with those who may be interested.

***DO NOT- Make it about you (or your intent)***

- This is about the impact you had on somebody else and the possible harm you inflicted on another being.
- Whether or not you meant it does not matter. Keep it about the impact on the learner, not about what you wanted to message or hoped to come across.

---------------------------------------------------------------------------------------------------------------------

**CASE SCENARIO (PUBLIC EVENT):**

You are speaking at a conference and giving statistics about HIV in the community. In the description of risk factors, you include a reference to higher risk in “men who have sex with men” or MSM, a commonly used phrase in HIV health literature in the past.

A student in the audience speaks up when you pause for questions, saying, “I’m confused as to why you keep using the term MSM. It is offensive. It is not inclusive of identity and doesn’t describe any of the behaviors for HIV risk.”

As a group, determine the best verbal response for your apology in a public forum and email it to the rest of the group. Before the end of group work time, each person should practice saying aloud their personal version of that script.

Note: If you do not understand the historical context and the identification of the microaggression here, please take a few minutes to pause and read about this, coming together in your small group to determine why this statement is problematic. The group can then brainstorm about how they would develop this apology and what words they would use. This is an example of modeling commitment to educating oneself.

**Electronically**

***DO- Show gratitude***

- Similar to public speaking, it is important to express gratitude to the learner for bringing up this feedback. If they didn’t bring this up, then you wouldn’t know you had a negative impact, and you wouldn’t have an opportunity for change.

***DO- State the impact***

- For clarity, state the impact in your email explicitly, the way that you understand it. This ensures that the learner got their message across and that you are perceiving your impact correctly.

***DO- State your commitment***

- As above, go home and do your homework. And explicitly state that you are committed to doing better in the future.

“I am committed to doing better.”

***DO- Offer follow up options***

- Offer to meet in person to follow up on any lingering thoughts or concerns from the learner.

***DO- Ask for a second opinion before pressing “Send”***

- It is never a bad idea to get a second opinion before sending out a message on the internet.

***DO NOT- Make it about you (or your intent)***

- This is about the impact you had on somebody else and the possible harm you inflicted

on another being.

- Whether or not you meant it does not matter. Keep it about the impact on the learner, not about what you wanted to message or hoped to come across.

***DO NOT- Respond when emotionally overwhelmed***

- Emails are considerably more difficult because now you are committing yourself to your words that can be forwarded, screen-shot, and placed on the internet for all to view.
- DO NOT respond when you are emotionally charged. Take some time to, again, reflect on your feelings and process them-- and then turn your attention to the learner.

---------------------------------------------------------------------------------------------------------------------

**CASE SCENARIO (EMAIL):**

You receive an email from a resident after a workday:

Dr. ______,

I wanted to address an issue that made me feel uncomfortable today. When we were working in our simulated cases, you persistently called the standardized patient by the wrong pronoun, using “he” instead of “they.” I didn’t know how to bring it up, but our classmates noticed it too. I hope this doesn’t happen again.

Sincerely,

As a group, draft a template email to respond to this learner and email it out to your group members to save as a reminder for the future. Each group member can personalize the template to their style.
