# Supplementary material for: Acknowledging and Addressing Microaggressions: A Virtual Experiential Learning Approach for Faculty
Source: MedEdPORTAL. 2024 Sep 4;20:11436. doi: 10.15766/mep_2374-8265.11436 (PMC11374130; doi:10.15766/mep_2374-8265.11436)
Supplement: Supplementary file 1 — Sample Flier.pptxWorkshop 1 - Slides.pptxWorkshop 1 - Facilitator GuideWorkshop 1 - Participant Handout.docxWorkshop 1 - Pre- and Postsurvey.docxWorkshop 2 - Slides.pptxWorkshop 2 - Facilitator Guide.docxWorkshop 2 - Participant Handout.docxWorkshop 2 - Pre- and Postsurvey.docxWorkshop 3 - Slides.pptxWorkshop 3 - Facilitator Guide.docxWorkshop 3 - Participant Handout.docxWorkshop 3 - Pre- and Postsurvey.docxWorkshop 4 - Slides.pptxWorkshop 4 - Facilitator Guide.docxWorkshop 4 - Participant Handout.docxWorkshop 4 - Pre- and Postsurvey.docx [file mep_2374-8265.11436-s001.zip › I. Workshop 2 - Pre- and Postsurvey.docx]

**Microaggressions Workshop #2: Apologizing When You’ve Done Harm Skills PRE-SURVEY**

**Do you consent to using your responses as part of the research surrounding this work? ?**

_____ **YES**, you may use my responses in the research study.

_____ **NO**, you may NOT use my responses in the research study.

**What is your participant ID?** _______________ **(only asked if individual participates in study)**

2-digit birth**DAY** + last 2 letters of birth **CITY** + first initial of each **PARENT** in alphabetical order (use X if unknown)

E.g., Participant ID for a person born on July **09** in Tope**ka** whose parents are **K**yle and **S**am is **09KAKS**

**Demographic Information**

1. **My GME affiliated role is in the department of ____________**
2. **What is your race/ethnicity? (check all that apply)**

_____ American Indian or Alaska Native _____ White

_____ Asian _____ Multi-race/Ethnicity

_____ Black or African American _____ Unknown/Prefer not to say

_____ LatinX _____ Other/self-describe: _______

_____ Native Hawaiian or Other Pacific Islander

1. **What gender do you identify with?**

_____ Female _____ Other (please specify): _______

_____ Male ____ Prefer not to sate

_____ Transgender female

_____ Transgender male

_____ Genderqueer/gender non-conforming

1. **Other identities that I hold related to my leadership/teaching role (free text):**
2. **Previous workshops I have participated in include: (Drop down boxes)**
3. **Acknowledging and Naming Microaggressions: Virtual 8/30**
4. **Acknowledging and Naming Microaggressions: In Person 9/20**
5. **This is my first workshop**

**6. I prefer to participate in these workshops:**

1. **In person**
2. **Virtually**

**To what extent do you agree with these statements? (check one per row)**

| **Recognizing Learner’s Harm** | **Strongly Disagree** | **Disagree** | **Neutral** | **Agree** | **Strongly Agree** |
| --- | --- | --- | --- | --- | --- |
| It is important to recognize when a learner has been harmed by a microaggression in the learning environment. |  |  |  |  |  |
| I am confident in my ability to recognize when a learner has been harmed by a microaggression in the clinical learning environment. |  |  |  |  |  |
| **Effective Communication with apologies** | **Strongly Disagree** | **Disagree** | **Neutral** | **Agree** | **Strongly Agree** |
| I am comfortable apologizing to a trainee who has been hurt by a microaggression *in person (one on one).* |  |  |  |  |  |
| I am comfortable apologizing to a trainee who has been hurt by a microaggression *in public.* |  |  |  |  |  |
| I am comfortable apologizing to a trainee who has been hurt by a microaggression *electronically (ie email).* |  |  |  |  |  |

**My biggest barriers to communicating apologies to trainees when they have experienced harm:**

**Microaggression Workshop #2: Apologizing When You’ve Done Harm Skills POST-SURVEY**

**To what extent do you agree with these statements? (check one per row)**

| **Recognizing Learner’s Harm** | **Strongly Disagree** | **Disagree** | **Neutral** | **Agree** | **Strongly Agree** |
| --- | --- | --- | --- | --- | --- |
| It is important to recognize when a learner has been harmed by a microaggression in the learning environment. |  |  |  |  |  |
| I am confident in my ability to recognize when a learner has been harmed by a microaggression in the clinical learning environment. |  |  |  |  |  |
| **Effective Communication with apologies** | **Strongly Disagree** | **Disagree** | **Neutral** | **Agree** | **Strongly Agree** |
| I am comfortable apologizing to a trainee who has been hurt by a microaggression *one-on-one.* |  |  |  |  |  |
| I am comfortable apologizing to a trainee who has been hurt by a microaggression *in public.* |  |  |  |  |  |
| I am comfortable apologizing to a trainee who has been hurt by a microaggression *electronically (ie email).* |  |  |  |  |  |

1. What was the **most useful** part of this workshop? Why?
2. What was the **least useful** part of this workshop? Why?
3. What would you **change** about this workshop? Why?
4. Please feel free to offer feedback to your facilitators. Did they create an inclusive learning environment? What did they do well? What could they do better?
5. Commit to one personal change you will make to create a more inclusive learning environment after this workshop:
