# Supplementary material for: Acknowledging and Addressing Microaggressions: A Virtual Experiential Learning Approach for Faculty
Source: MedEdPORTAL. 2024 Sep 4;20:11436. doi: 10.15766/mep_2374-8265.11436 (PMC11374130; doi:10.15766/mep_2374-8265.11436)
Supplement: Supplementary file 1 — Sample Flier.pptxWorkshop 1 - Slides.pptxWorkshop 1 - Facilitator GuideWorkshop 1 - Participant Handout.docxWorkshop 1 - Pre- and Postsurvey.docxWorkshop 2 - Slides.pptxWorkshop 2 - Facilitator Guide.docxWorkshop 2 - Participant Handout.docxWorkshop 2 - Pre- and Postsurvey.docxWorkshop 3 - Slides.pptxWorkshop 3 - Facilitator Guide.docxWorkshop 3 - Participant Handout.docxWorkshop 3 - Pre- and Postsurvey.docxWorkshop 4 - Slides.pptxWorkshop 4 - Facilitator Guide.docxWorkshop 4 - Participant Handout.docxWorkshop 4 - Pre- and Postsurvey.docx [file mep_2374-8265.11436-s001.zip › J. Workshop 3 - Slides.pptx]

## Slide 1
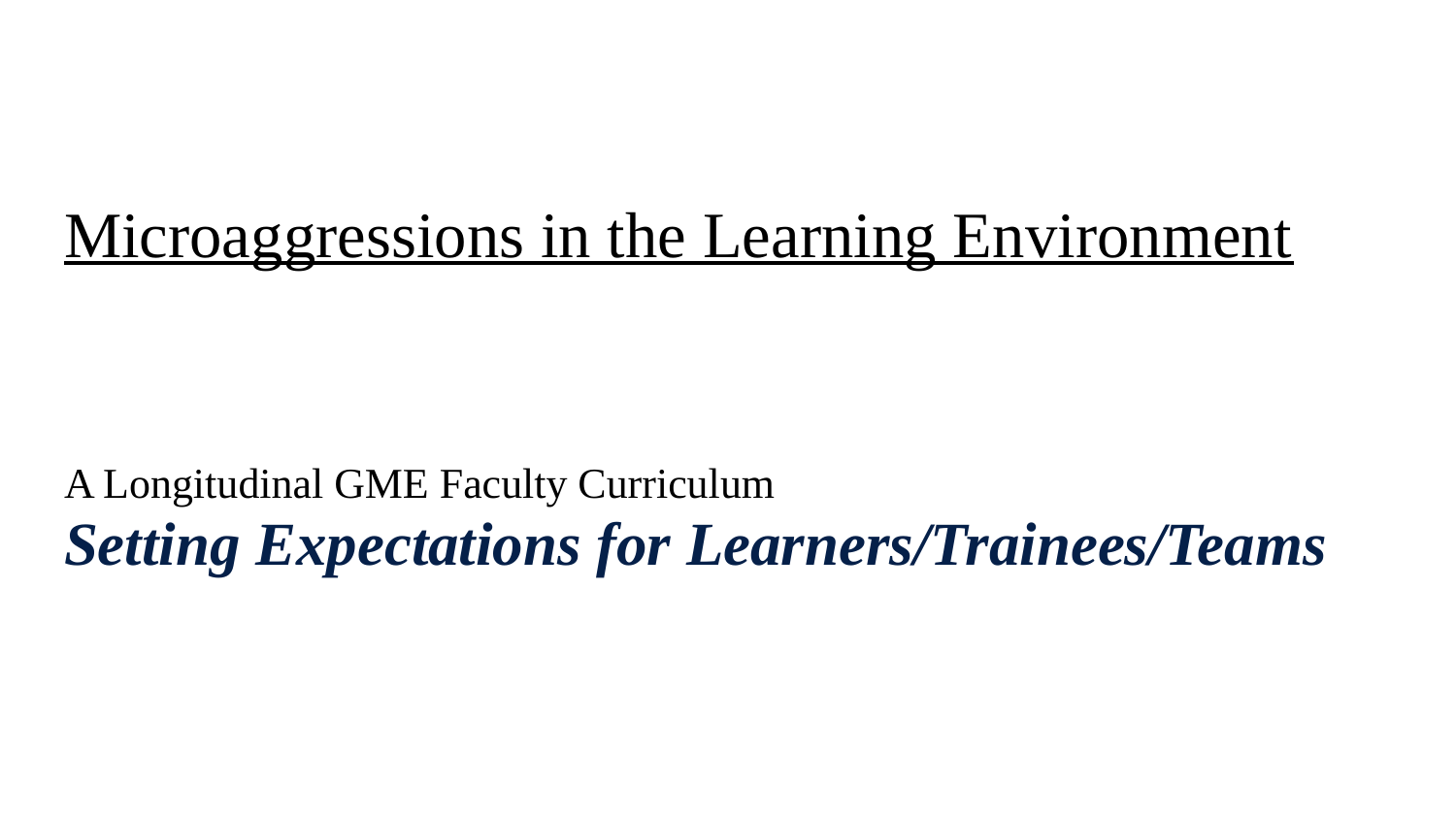

Microaggressions in the Learning Environment
A Longitudinal GME Faculty Curriculum
Setting Expectations for Learners/Trainees/Teams

## Slide 2
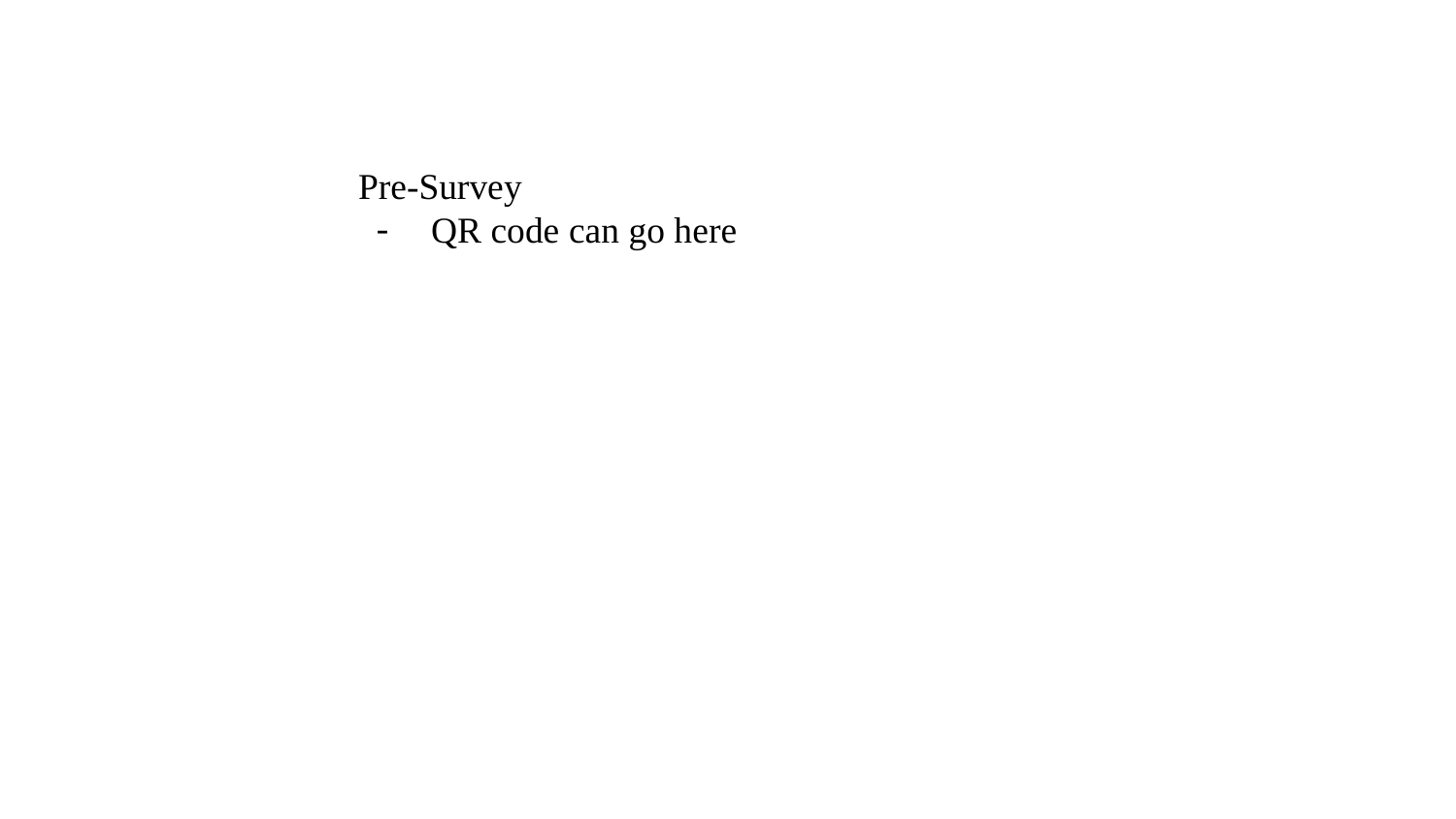

Pre-Survey
QR code can go here

## Slide 3
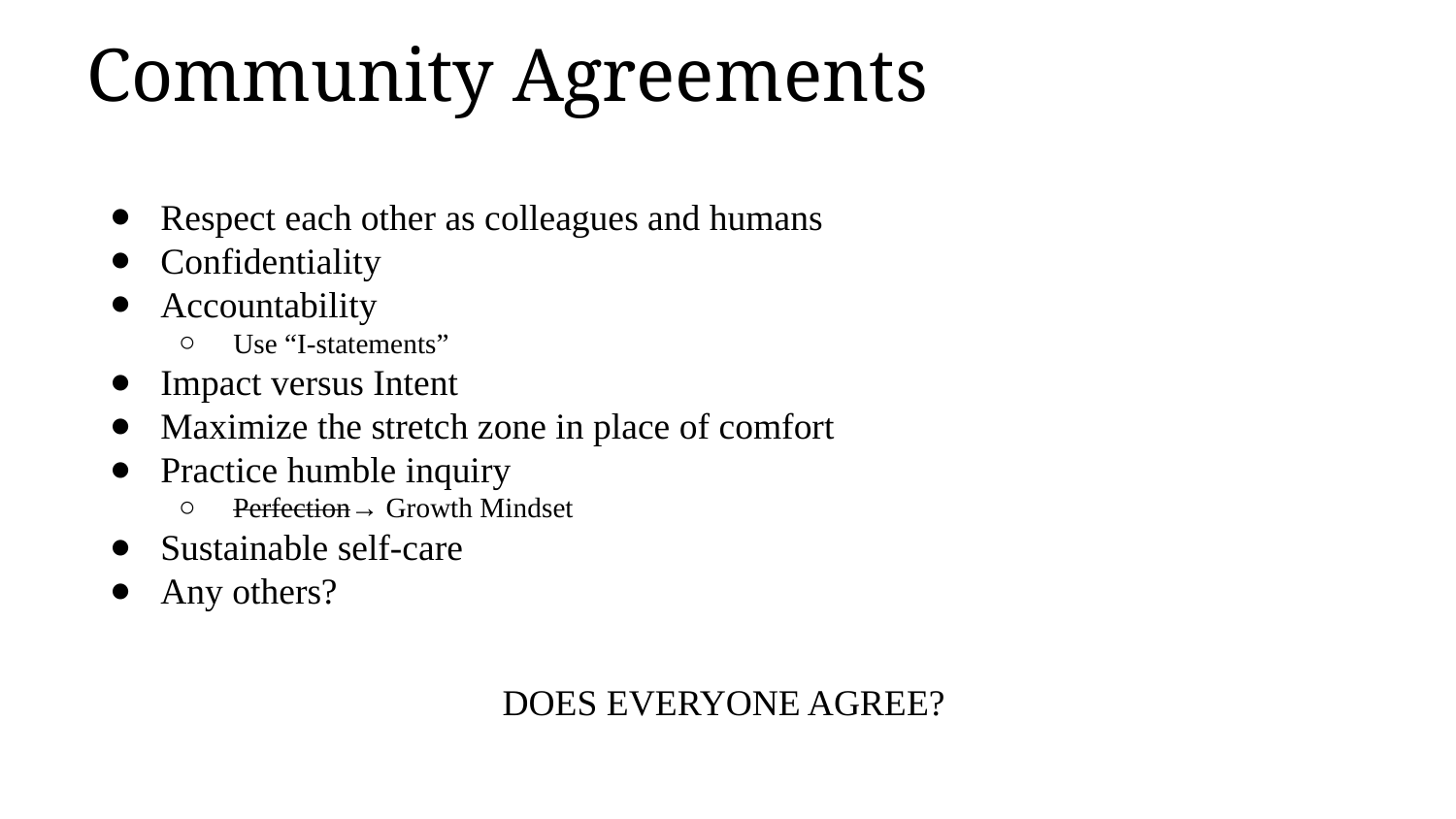

# Community Agreements
Respect each other as colleagues and humans
Confidentiality
Accountability
Use “I-statements”
Impact versus Intent
Maximize the stretch zone in place of comfort
Practice humble inquiry
Perfection→ Growth Mindset
Sustainable self-care
Any others?
DOES EVERYONE AGREE?

## Slide 4
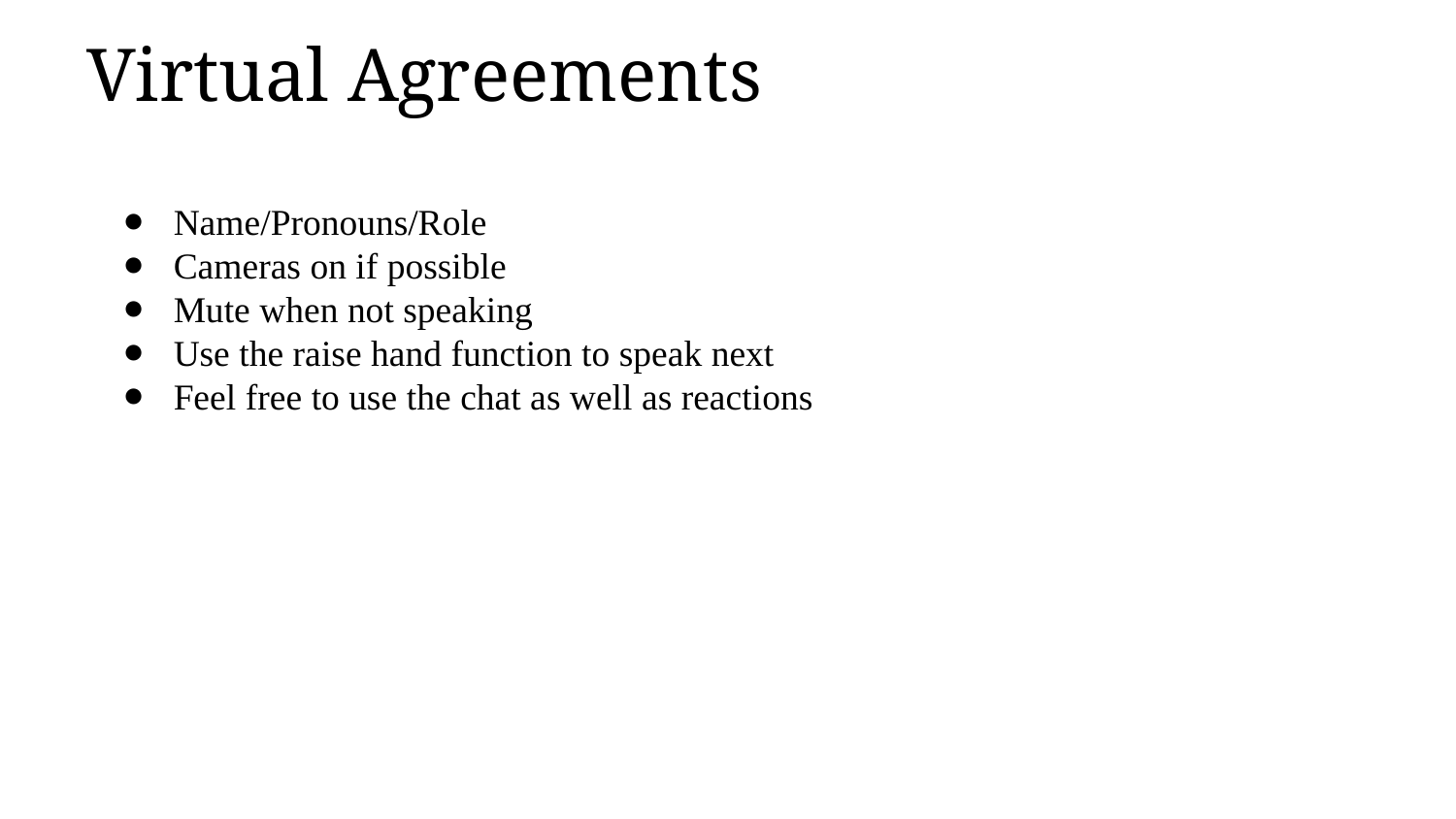

# Virtual Agreements
Name/Pronouns/Role
Cameras on if possible
Mute when not speaking
Use the raise hand function to speak next
Feel free to use the chat as well as reactions

## Slide 5
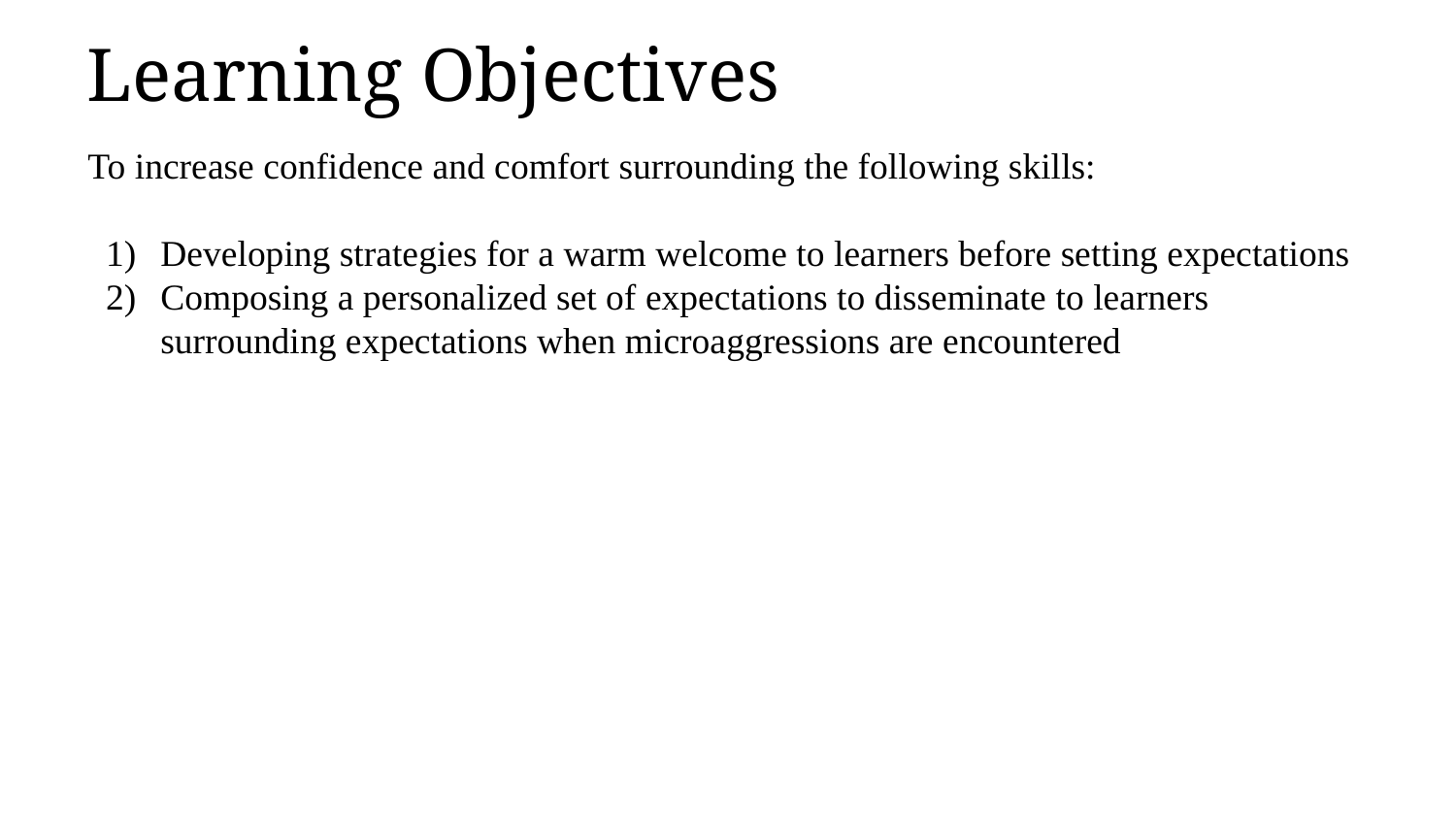

# Learning Objectives
To increase confidence and comfort surrounding the following skills:
Developing strategies for a warm welcome to learners before setting expectations
Composing a personalized set of expectations to disseminate to learners surrounding expectations when microaggressions are encountered

## Slide 6
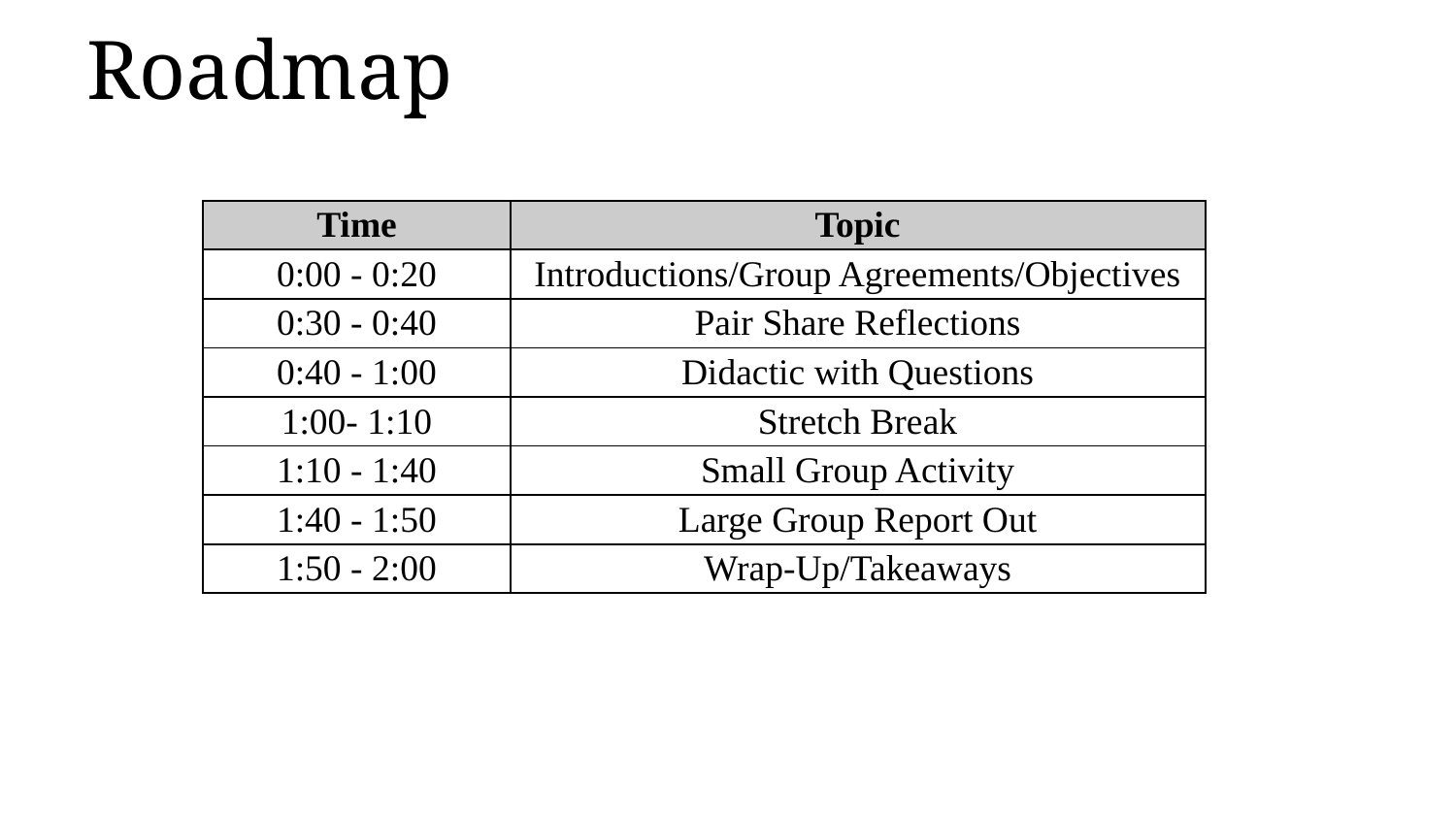

# Roadmap
| Time | Topic |
| --- | --- |
| 0:00 - 0:20 | Introductions/Group Agreements/Objectives |
| 0:30 - 0:40 | Pair Share Reflections |
| 0:40 - 1:00 | Didactic with Questions |
| 1:00- 1:10 | Stretch Break |
| 1:10 - 1:40 | Small Group Activity |
| 1:40 - 1:50 | Large Group Report Out |
| 1:50 - 2:00 | Wrap-Up/Takeaways |

## Slide 7
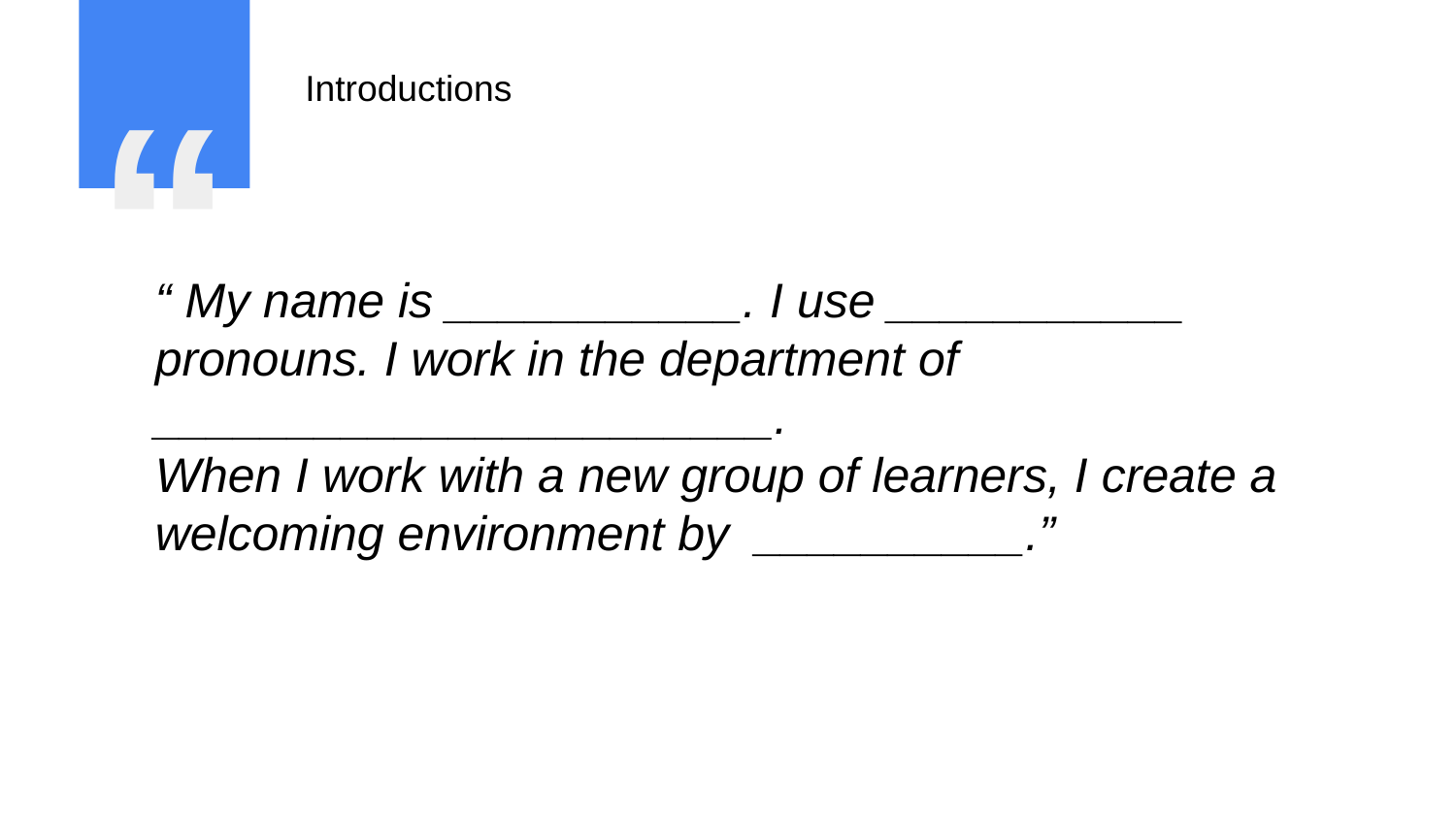

Introductions
“ My name is ___________. I use ___________ pronouns. I work in the department of _______________________.
When I work with a new group of learners, I create a welcoming environment by __________.”

## Slide 8
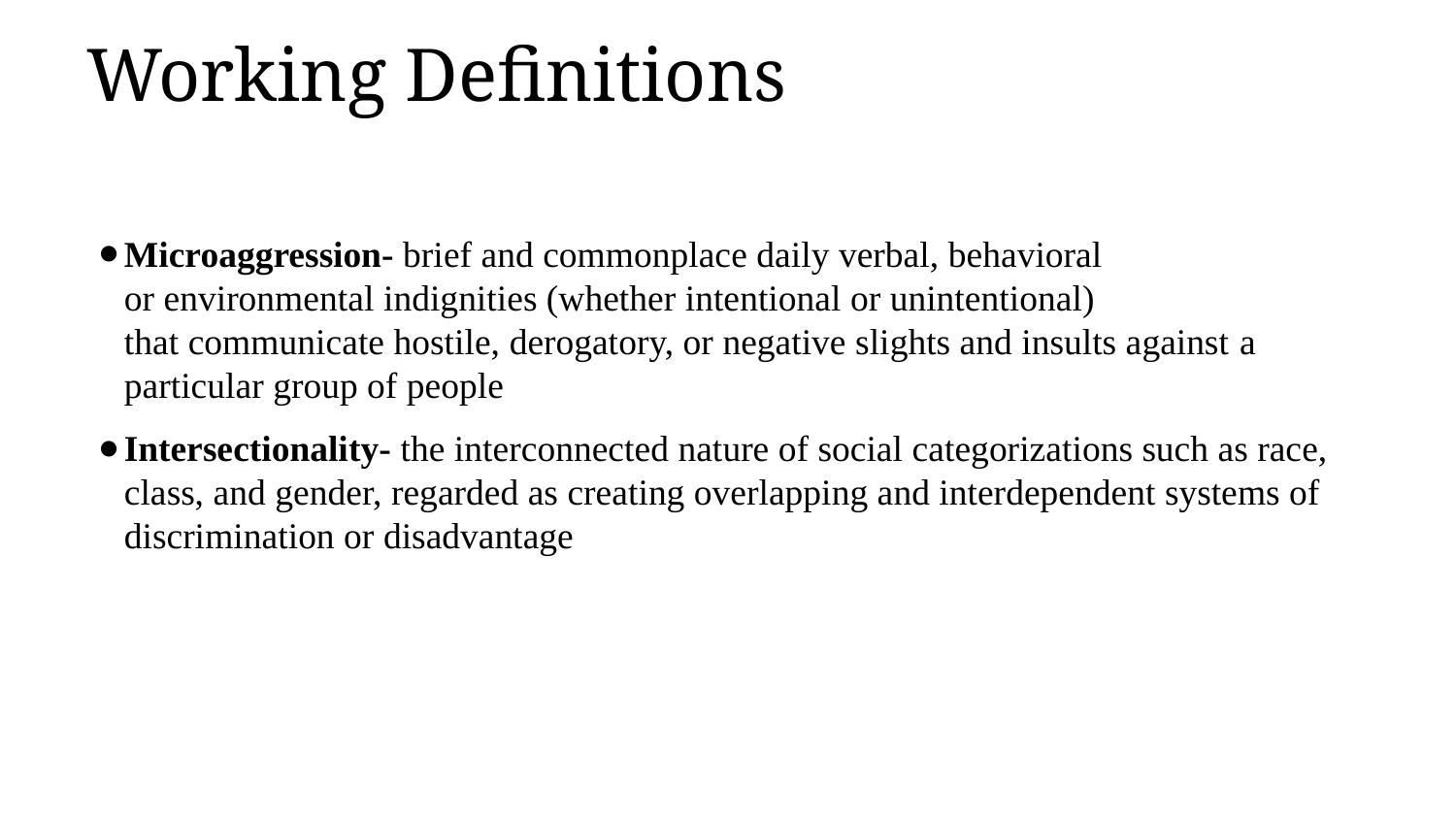

# Working Definitions
Microaggression- brief and commonplace daily verbal, behavioral or environmental indignities (whether intentional or unintentional) that communicate hostile, derogatory, or negative slights and insults against ​a particular group of people
Intersectionality- the interconnected nature of social categorizations such as race, class, and gender, regarded as creating overlapping and interdependent systems of discrimination or disadvantage​

## Slide 9
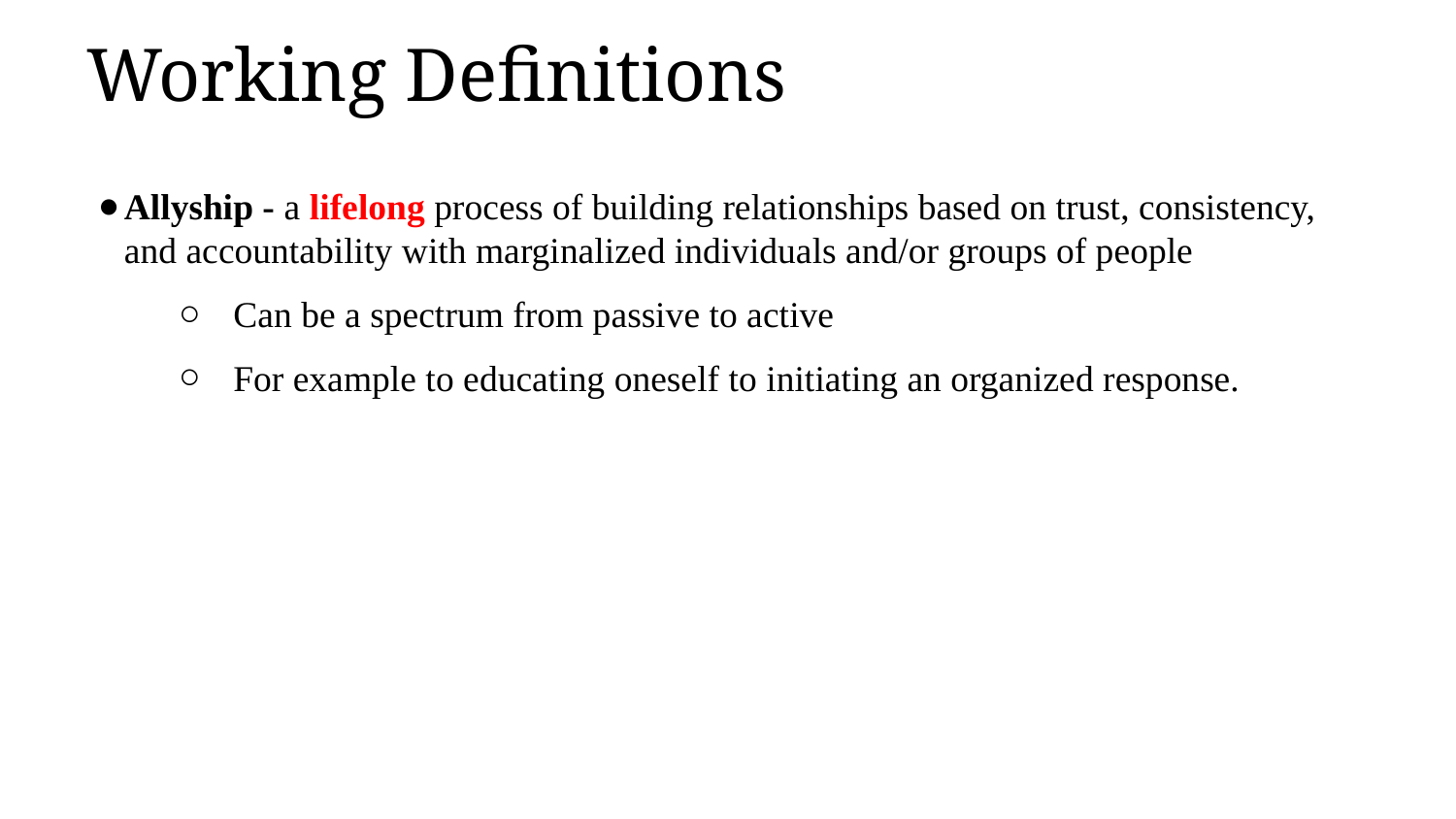

# Working Definitions
Allyship - a lifelong process of building relationships based on trust, consistency, and accountability with marginalized individuals and/or groups of people
Can be a spectrum from passive to active
For example to educating oneself to initiating an organized response.

## Slide 10
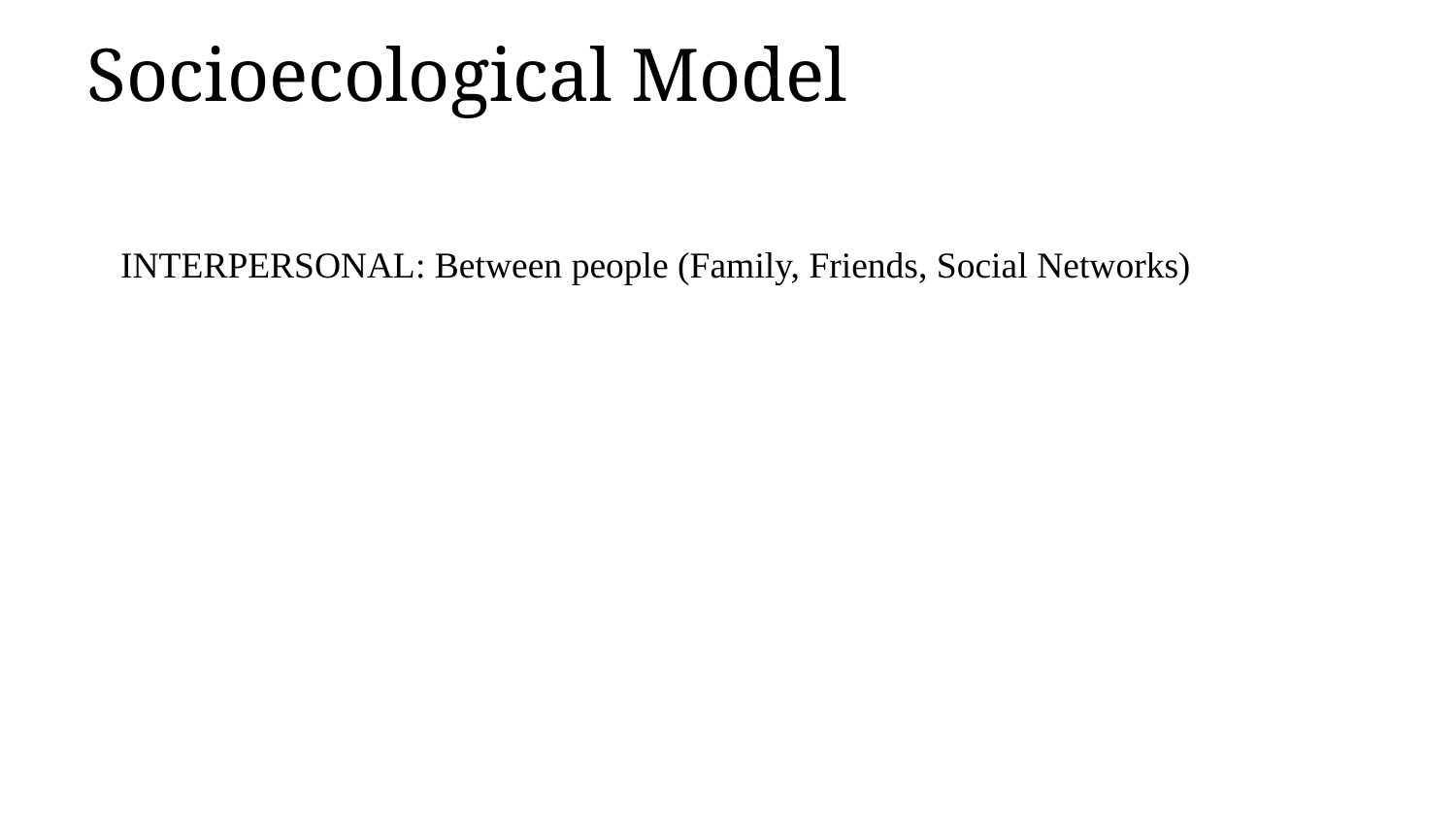

# Socioecological Model
INTERPERSONAL: Between people (Family, Friends, Social Networks)

## Slide 11
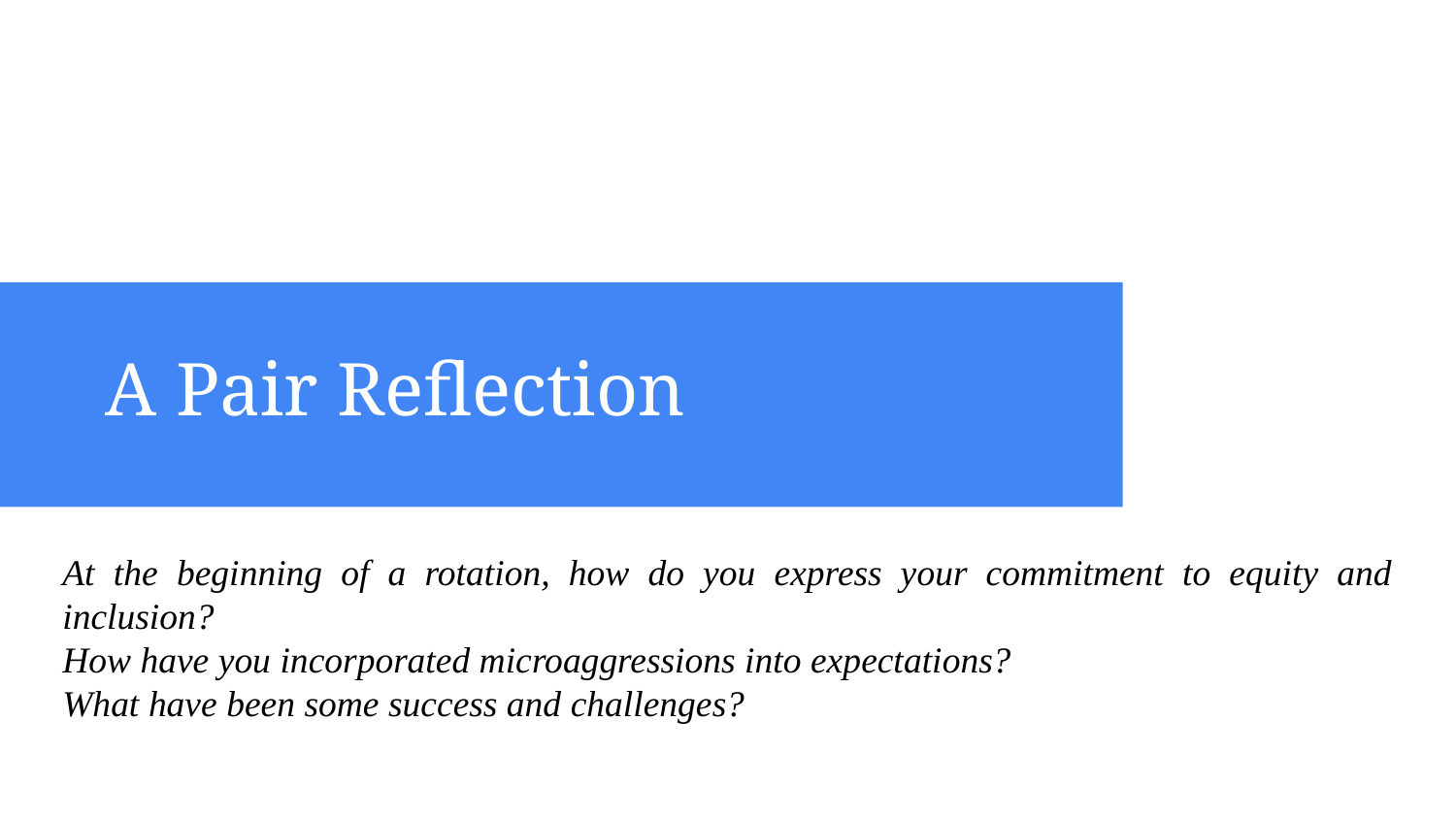

# A Pair Reflection
At the beginning of a rotation, how do you express your commitment to equity and inclusion?
How have you incorporated microaggressions into expectations?
What have been some success and challenges?

## Slide 12
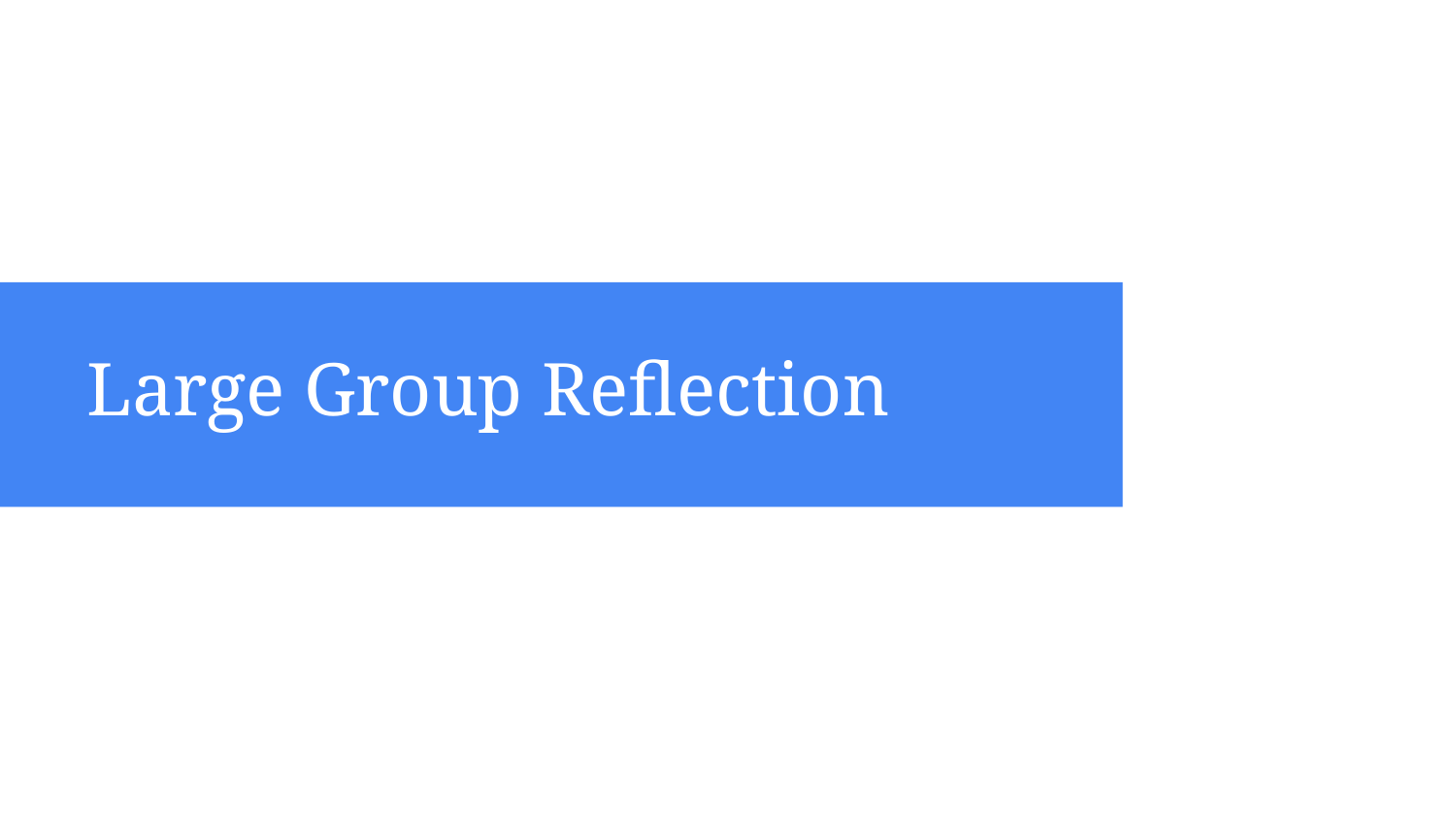

# Large Group Reflection

## Slide 13
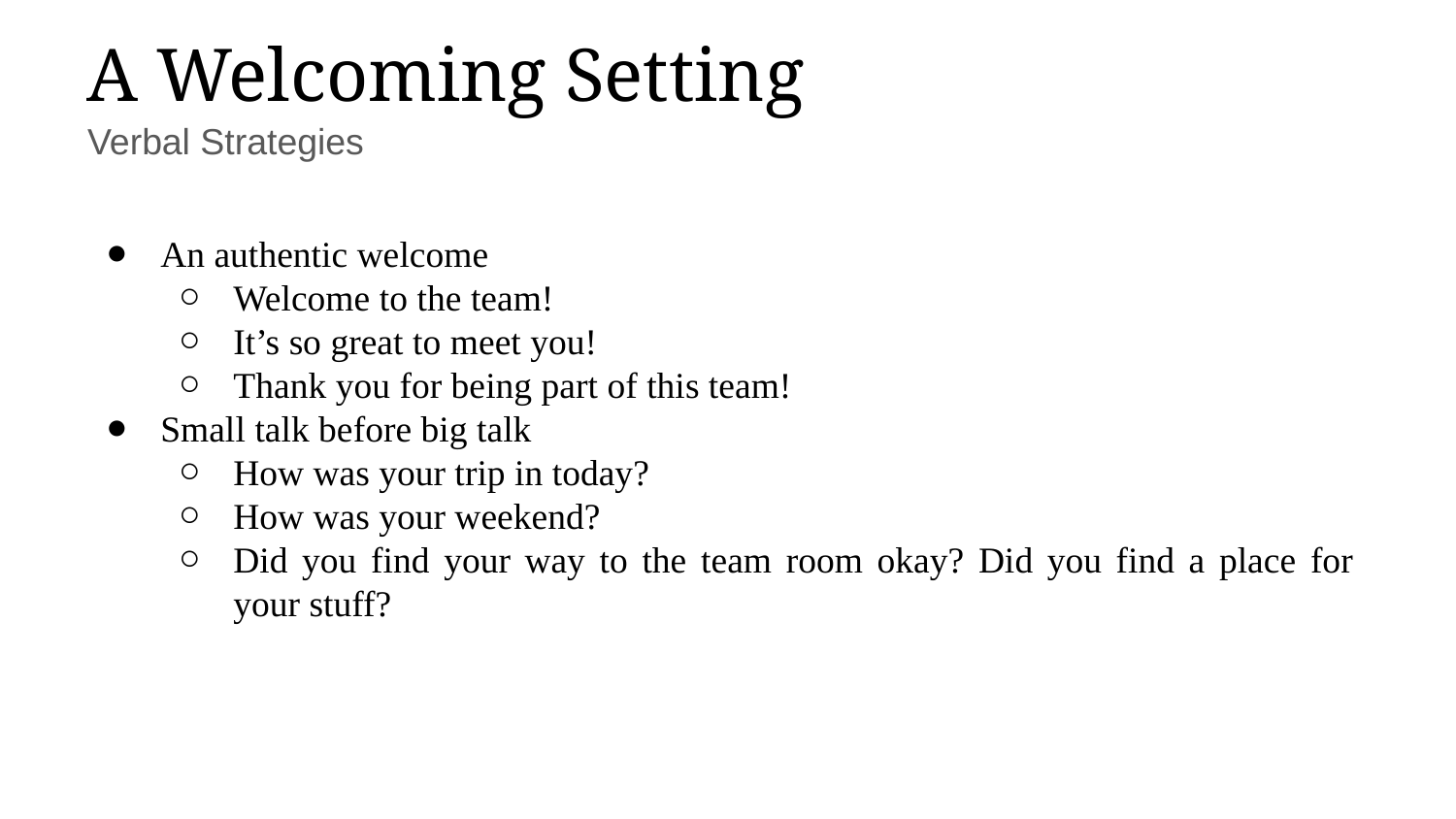

# A Welcoming Setting
Verbal Strategies
An authentic welcome
Welcome to the team!
It’s so great to meet you!
Thank you for being part of this team!
Small talk before big talk
How was your trip in today?
How was your weekend?
Did you find your way to the team room okay? Did you find a place for your stuff?

## Slide 14
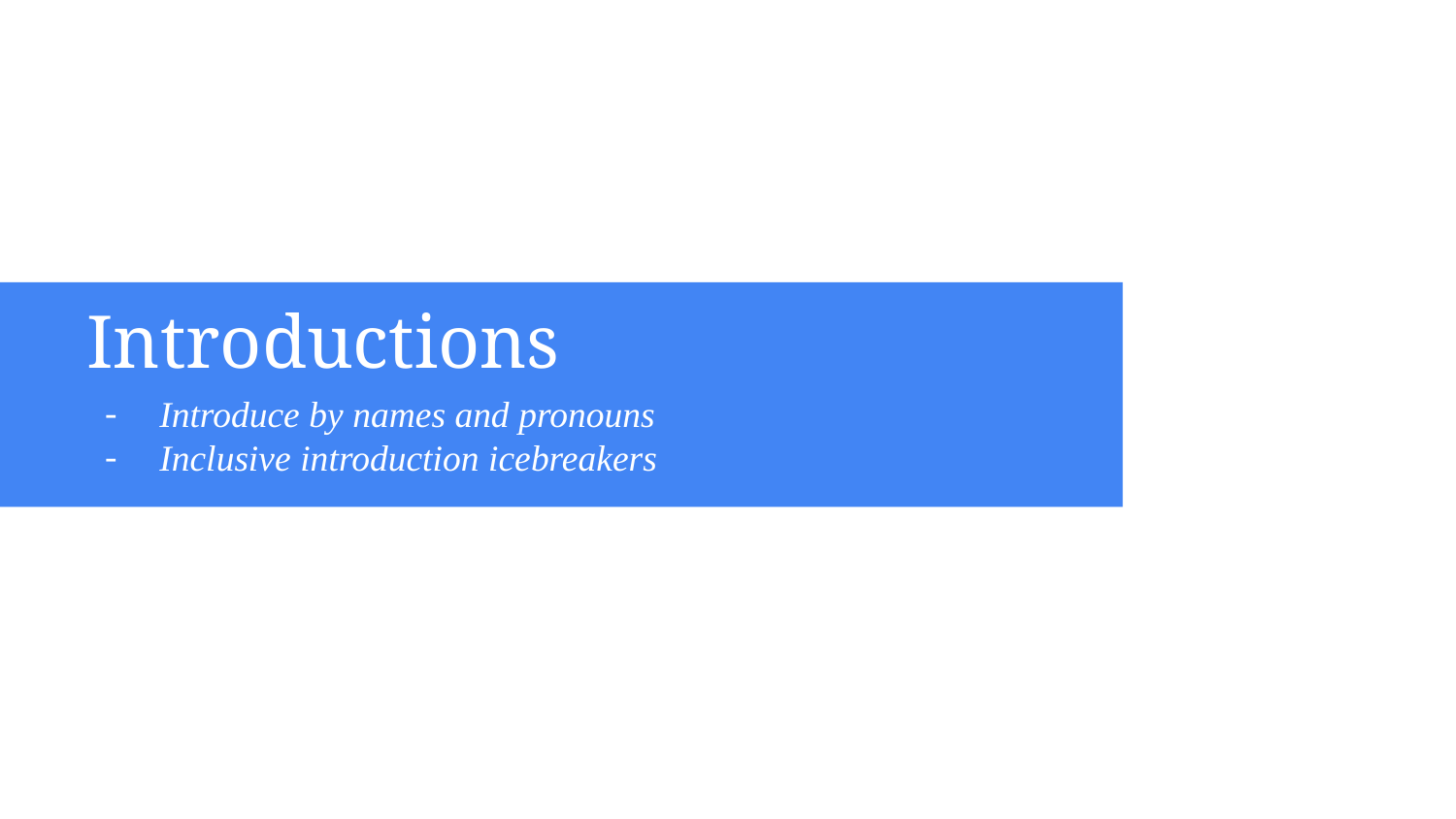

# Introductions
Introduce by names and pronouns
Inclusive introduction icebreakers

## Slide 15
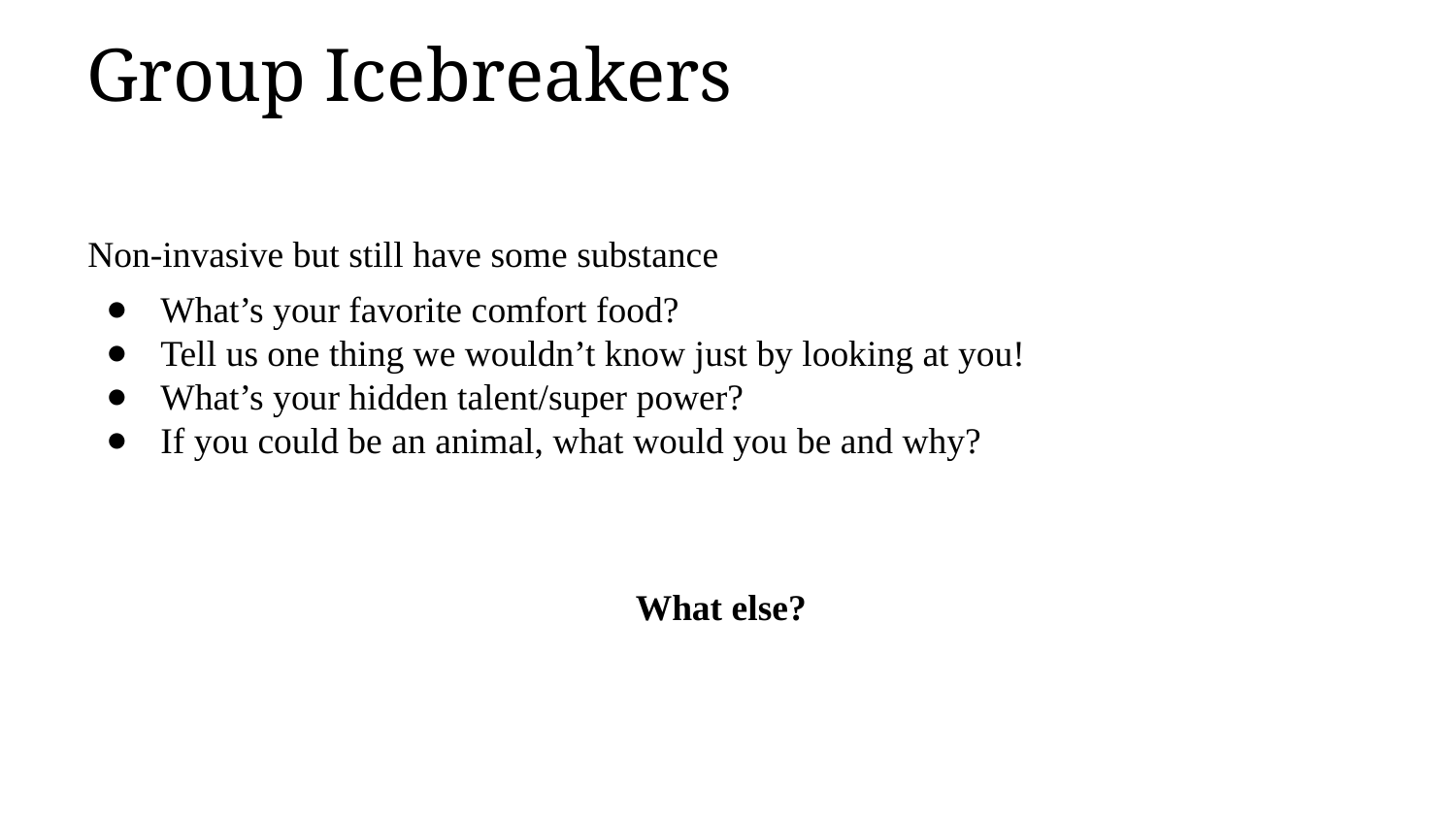

# Group Icebreakers
Non-invasive but still have some substance
What’s your favorite comfort food?
Tell us one thing we wouldn’t know just by looking at you!
What’s your hidden talent/super power?
If you could be an animal, what would you be and why?
What else?

## Slide 16
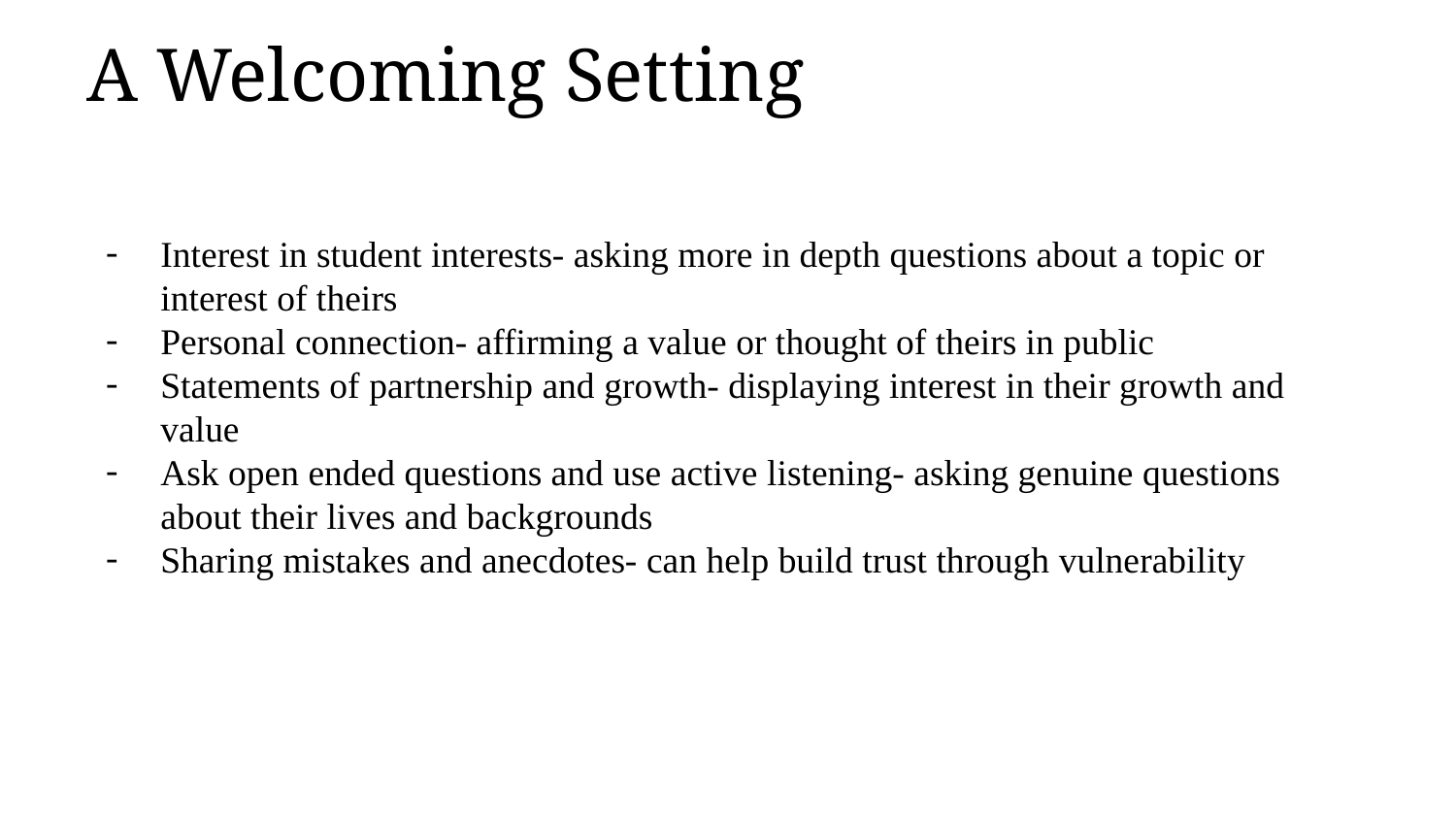

# A Welcoming Setting
Interest in student interests- asking more in depth questions about a topic or interest of theirs
Personal connection- affirming a value or thought of theirs in public
Statements of partnership and growth- displaying interest in their growth and value
Ask open ended questions and use active listening- asking genuine questions about their lives and backgrounds
Sharing mistakes and anecdotes- can help build trust through vulnerability

## Slide 17
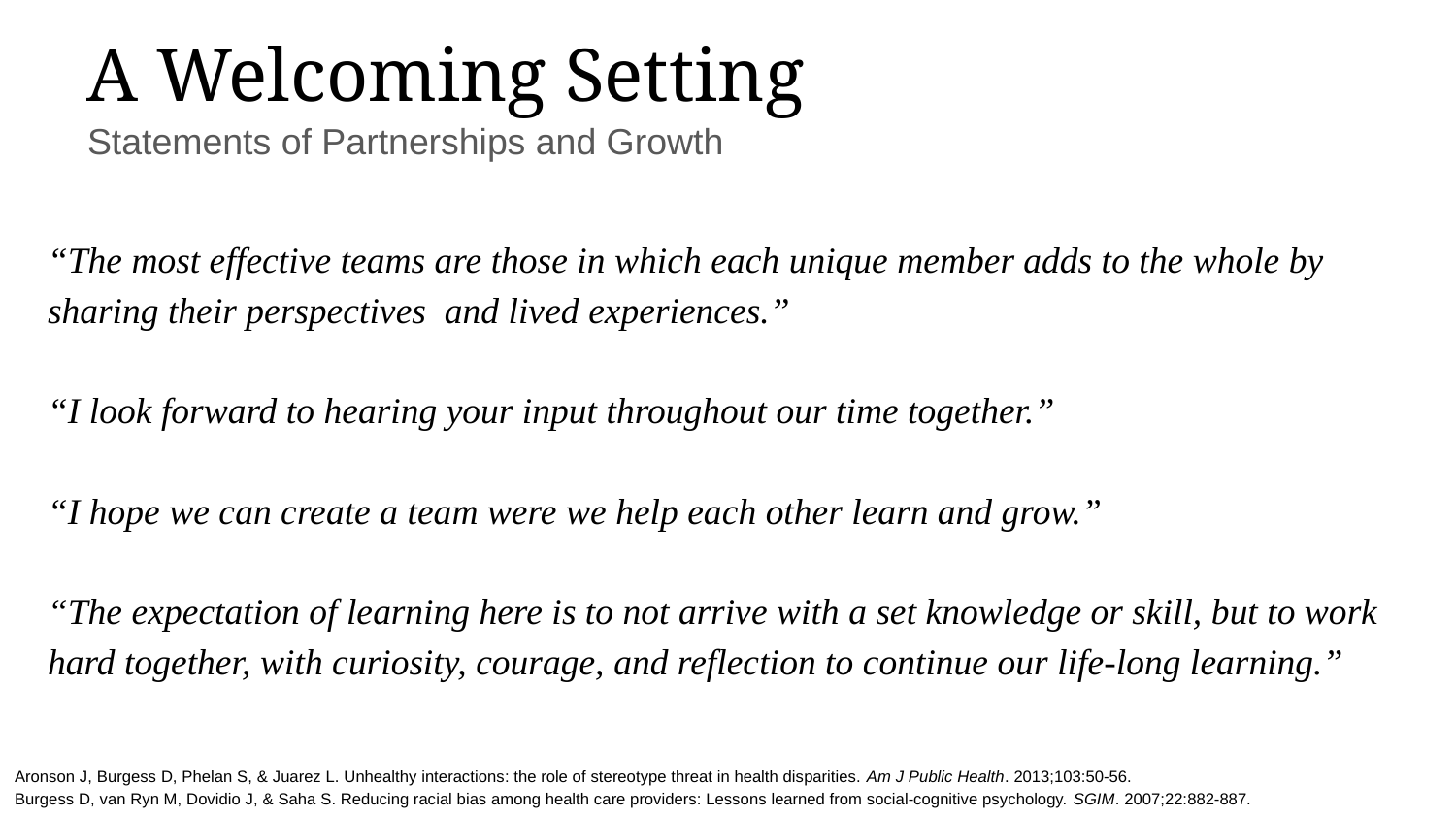

# A Welcoming Setting
Statements of Partnerships and Growth
“The most effective teams are those in which each unique member adds to the whole by sharing their perspectives and lived experiences.”
“I look forward to hearing your input throughout our time together.”
“I hope we can create a team were we help each other learn and grow.”
“The expectation of learning here is to not arrive with a set knowledge or skill, but to work hard together, with curiosity, courage, and reflection to continue our life-long learning.”
Aronson J, Burgess D, Phelan S, & Juarez L. Unhealthy interactions: the role of stereotype threat in health disparities. Am J Public Health. 2013;103:50-56.
Burgess D, van Ryn M, Dovidio J, & Saha S. Reducing racial bias among health care providers: Lessons learned from social-cognitive psychology. SGIM. 2007;22:882-887.

## Slide 18
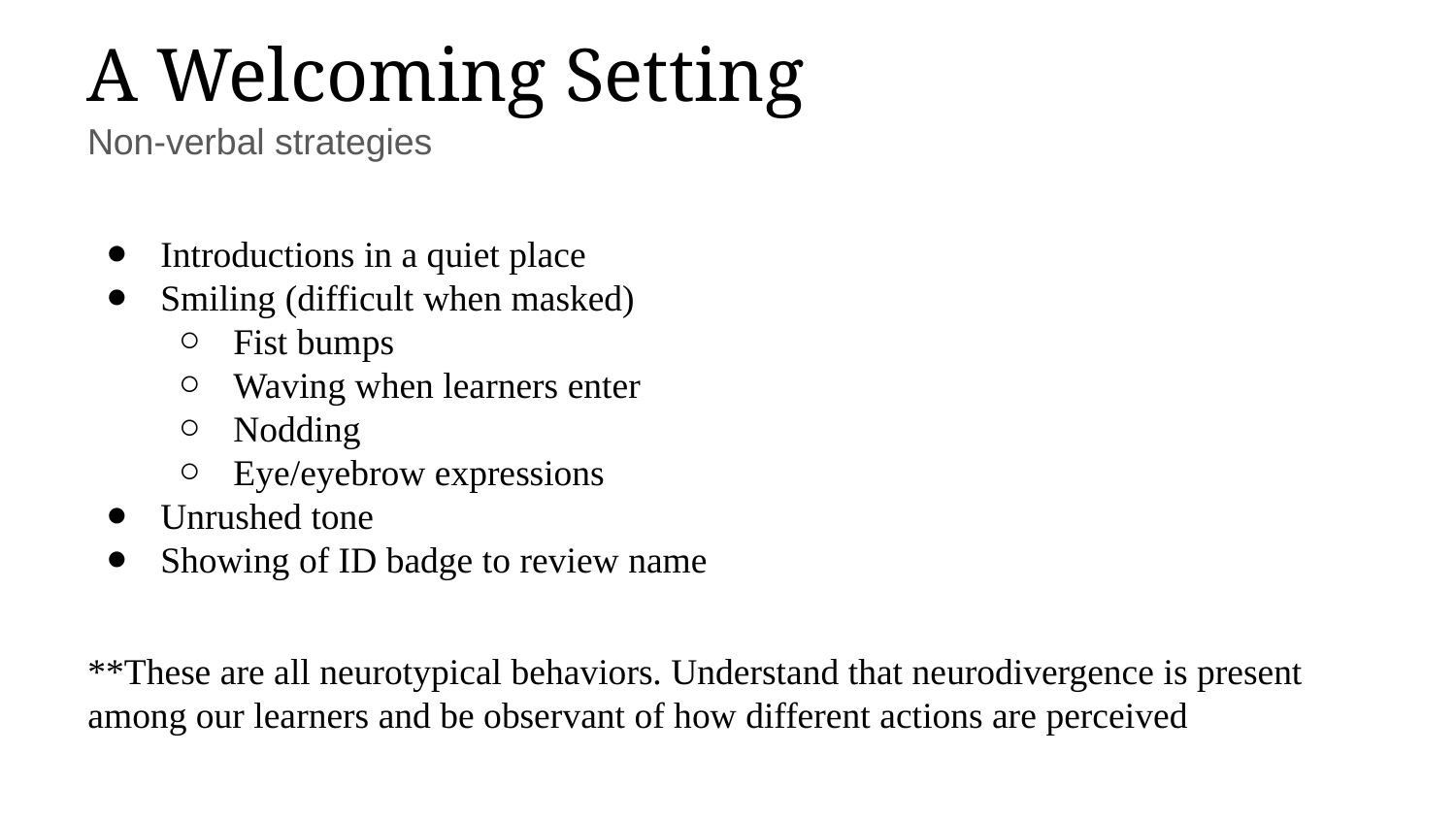

# A Welcoming Setting
Non-verbal strategies
Introductions in a quiet place
Smiling (difficult when masked)
Fist bumps
Waving when learners enter
Nodding
Eye/eyebrow expressions
Unrushed tone
Showing of ID badge to review name
**These are all neurotypical behaviors. Understand that neurodivergence is present among our learners and be observant of how different actions are perceived

## Slide 19
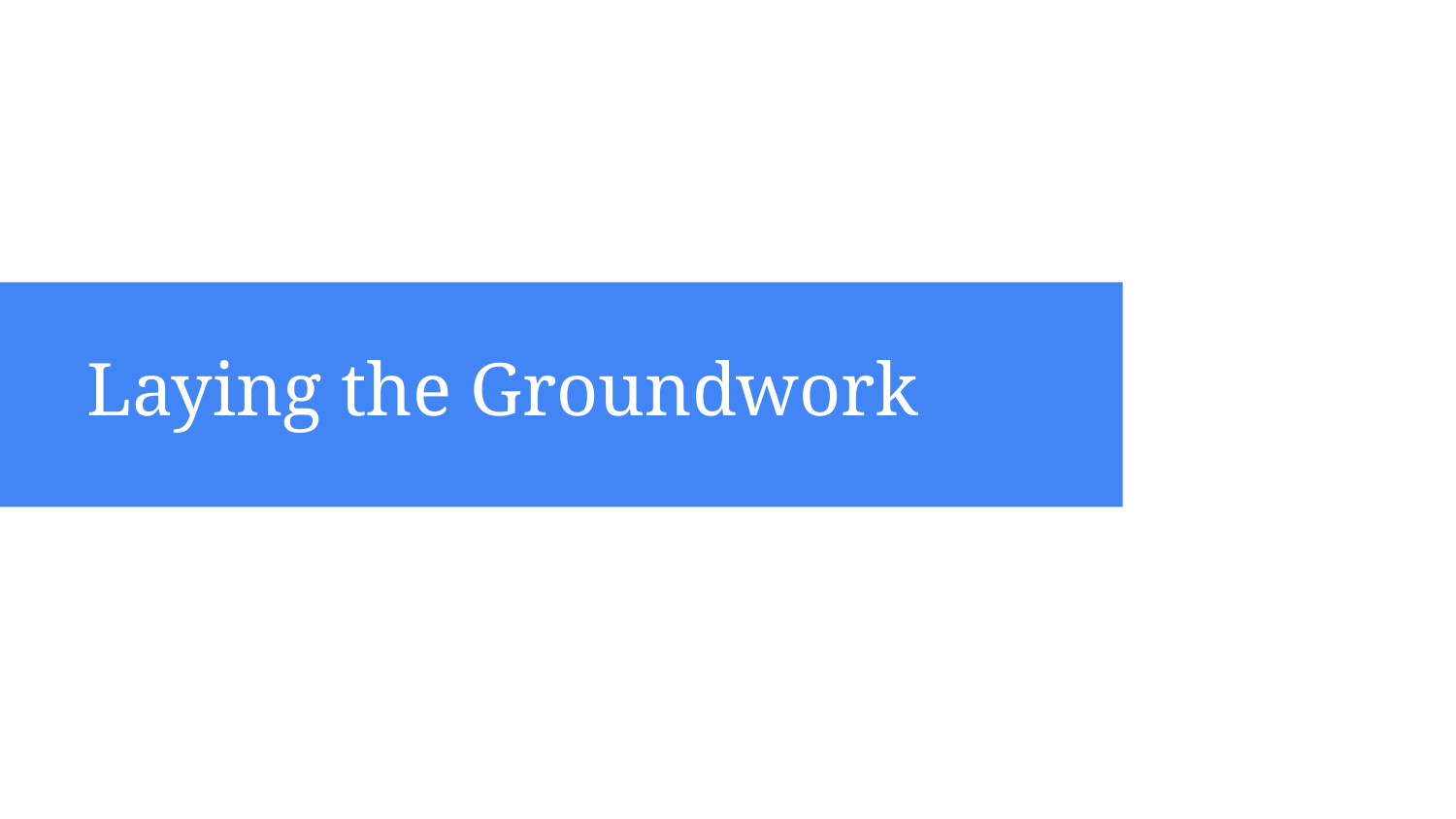

# Laying the Groundwork

## Slide 20
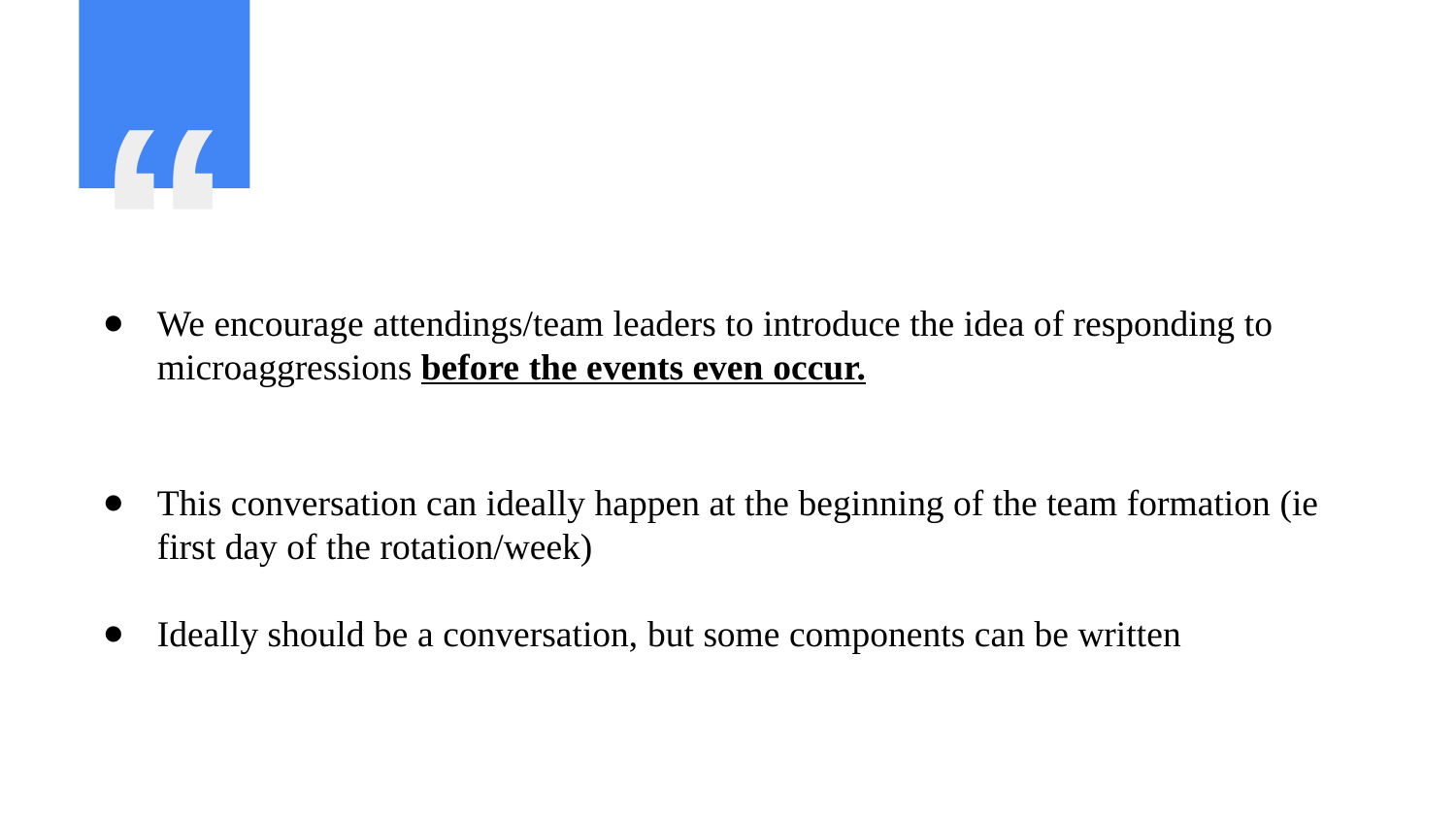

We encourage attendings/team leaders to introduce the idea of responding to microaggressions before the events even occur.
This conversation can ideally happen at the beginning of the team formation (ie first day of the rotation/week)
Ideally should be a conversation, but some components can be written

## Slide 21
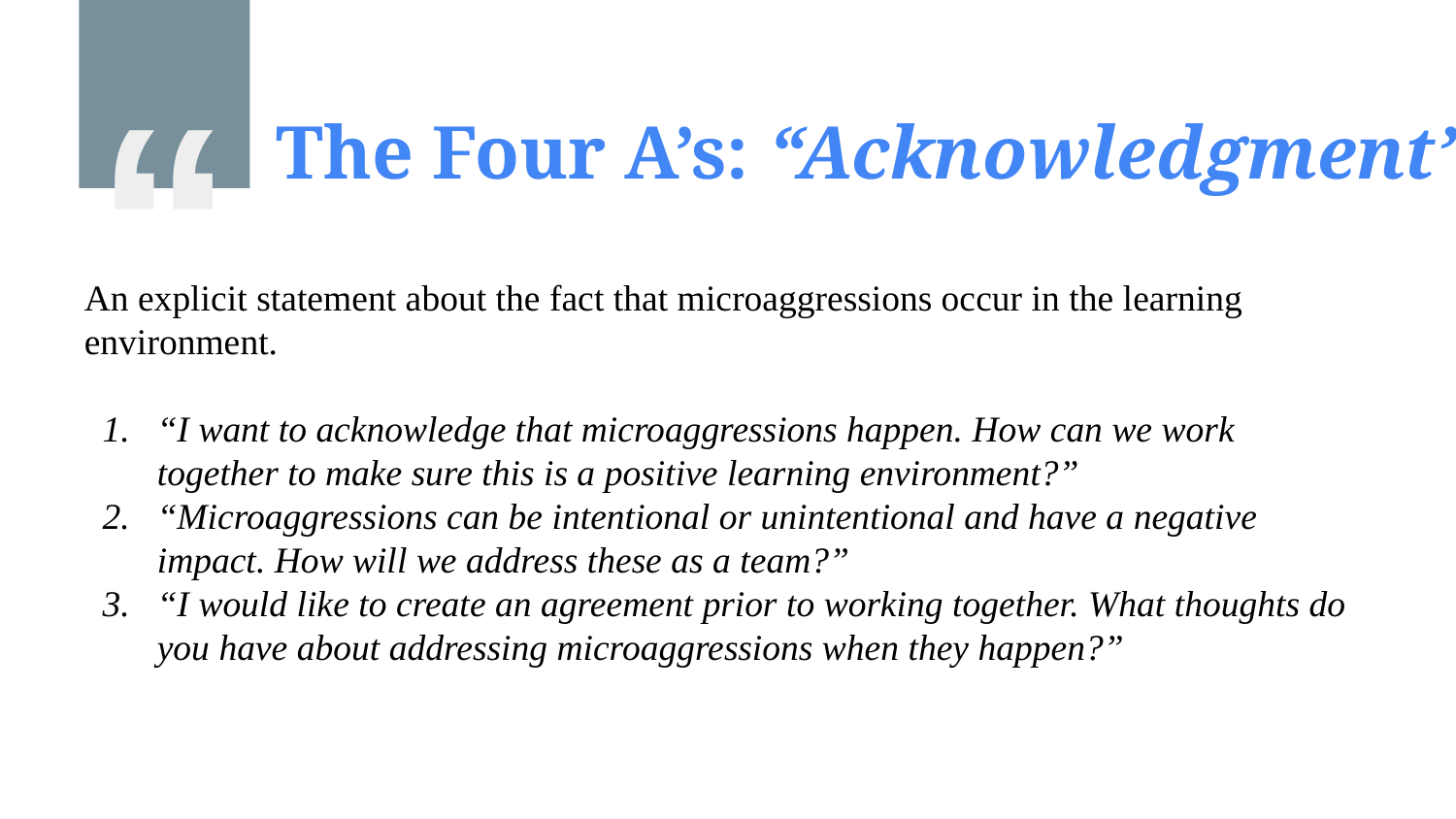

The Four A’s: “Acknowledgment”
An explicit statement about the fact that microaggressions occur in the learning environment.
“I want to acknowledge that microaggressions happen. How can we work together to make sure this is a positive learning environment?”
“Microaggressions can be intentional or unintentional and have a negative impact. How will we address these as a team?”
“I would like to create an agreement prior to working together. What thoughts do you have about addressing microaggressions when they happen?”

## Slide 22
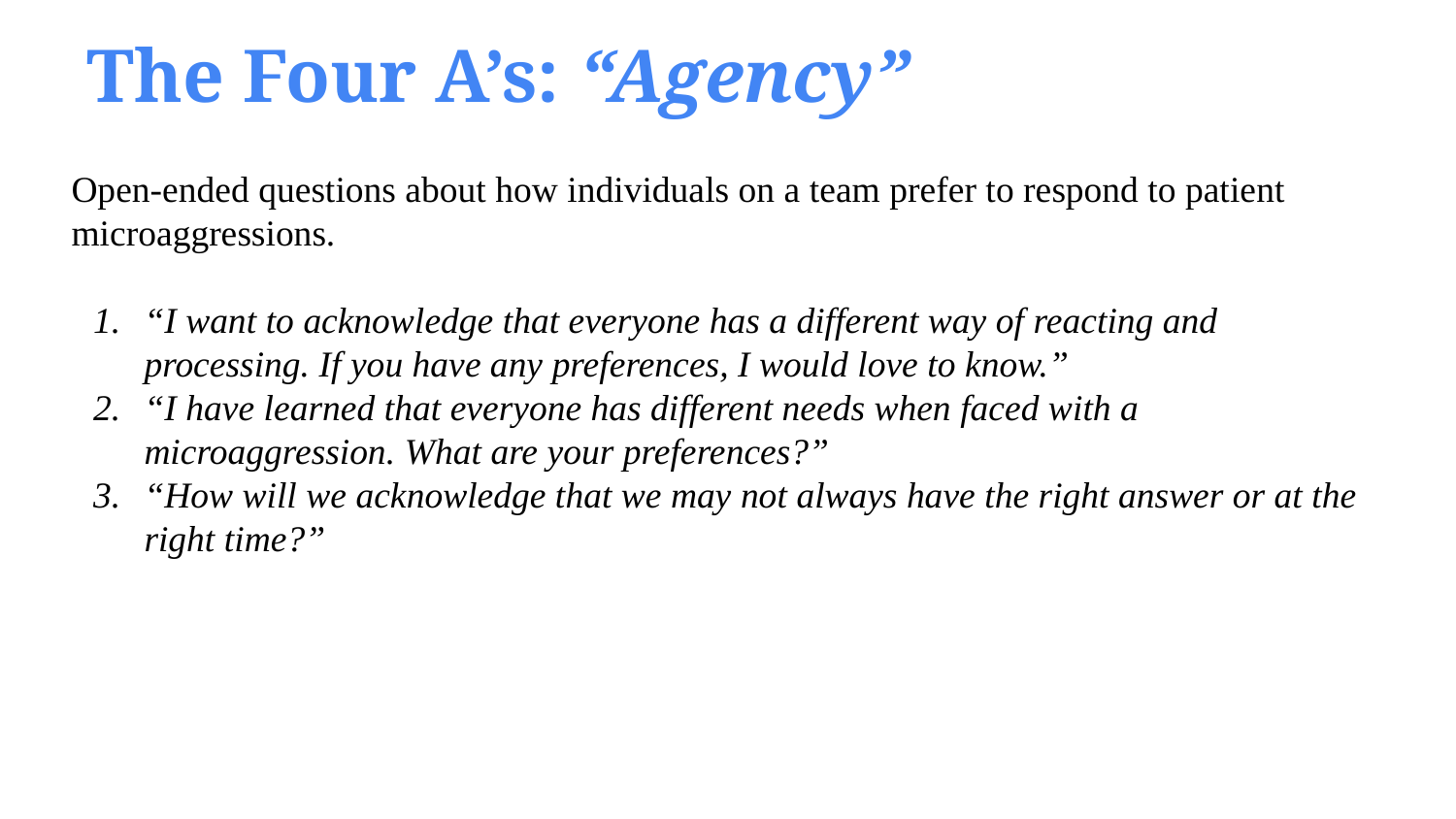

# The Four A’s: “Agency”
Open-ended questions about how individuals on a team prefer to respond to patient microaggressions.
“I want to acknowledge that everyone has a different way of reacting and processing. If you have any preferences, I would love to know.”
“I have learned that everyone has different needs when faced with a microaggression. What are your preferences?”
“How will we acknowledge that we may not always have the right answer or at the
right time?”

## Slide 23
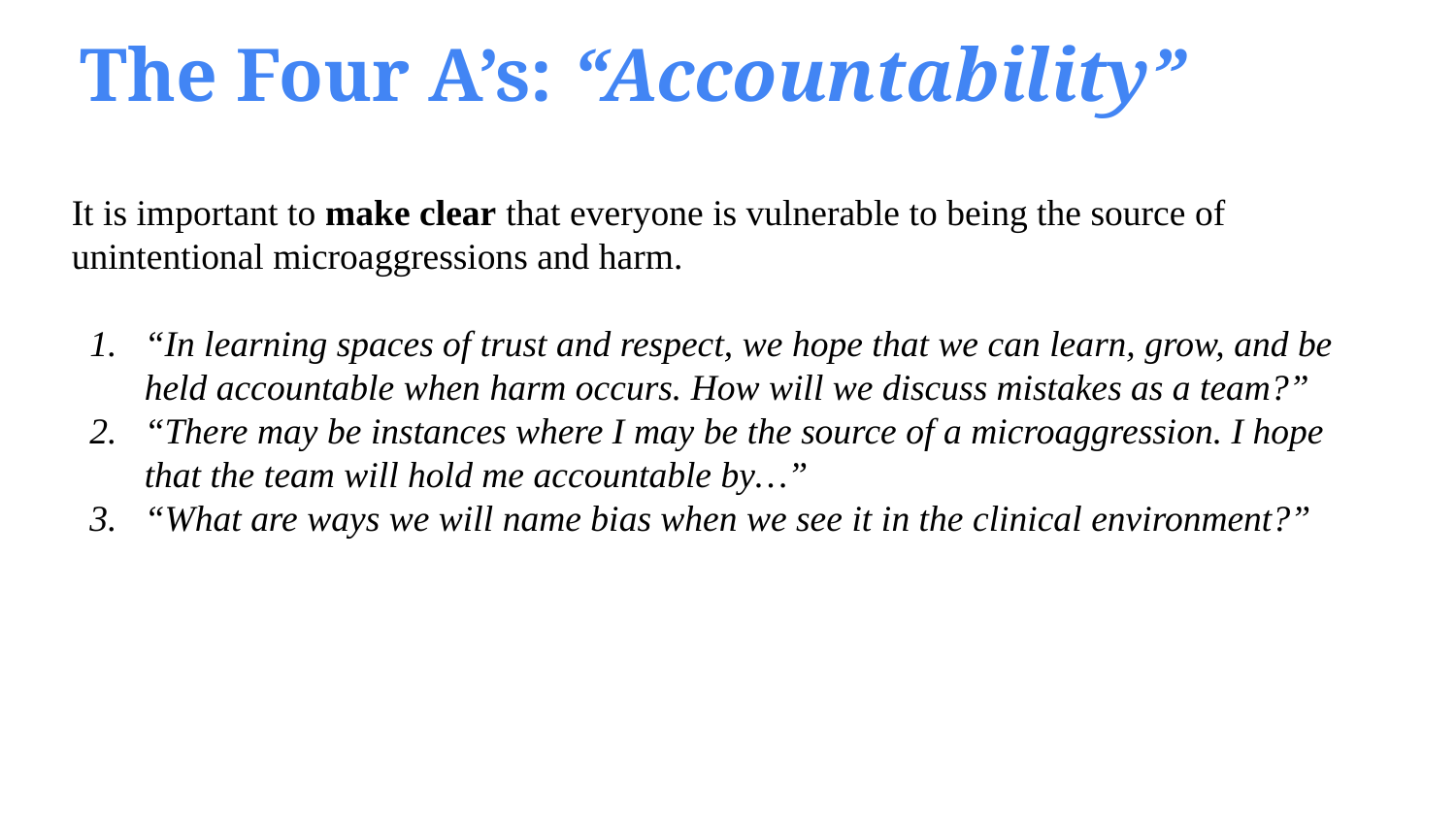

# The Four A’s: “Accountability”
It is important to make clear that everyone is vulnerable to being the source of unintentional microaggressions and harm.
“In learning spaces of trust and respect, we hope that we can learn, grow, and be held accountable when harm occurs. How will we discuss mistakes as a team?”
“There may be instances where I may be the source of a microaggression. I hope that the team will hold me accountable by…”
“What are ways we will name bias when we see it in the clinical environment?”

## Slide 24
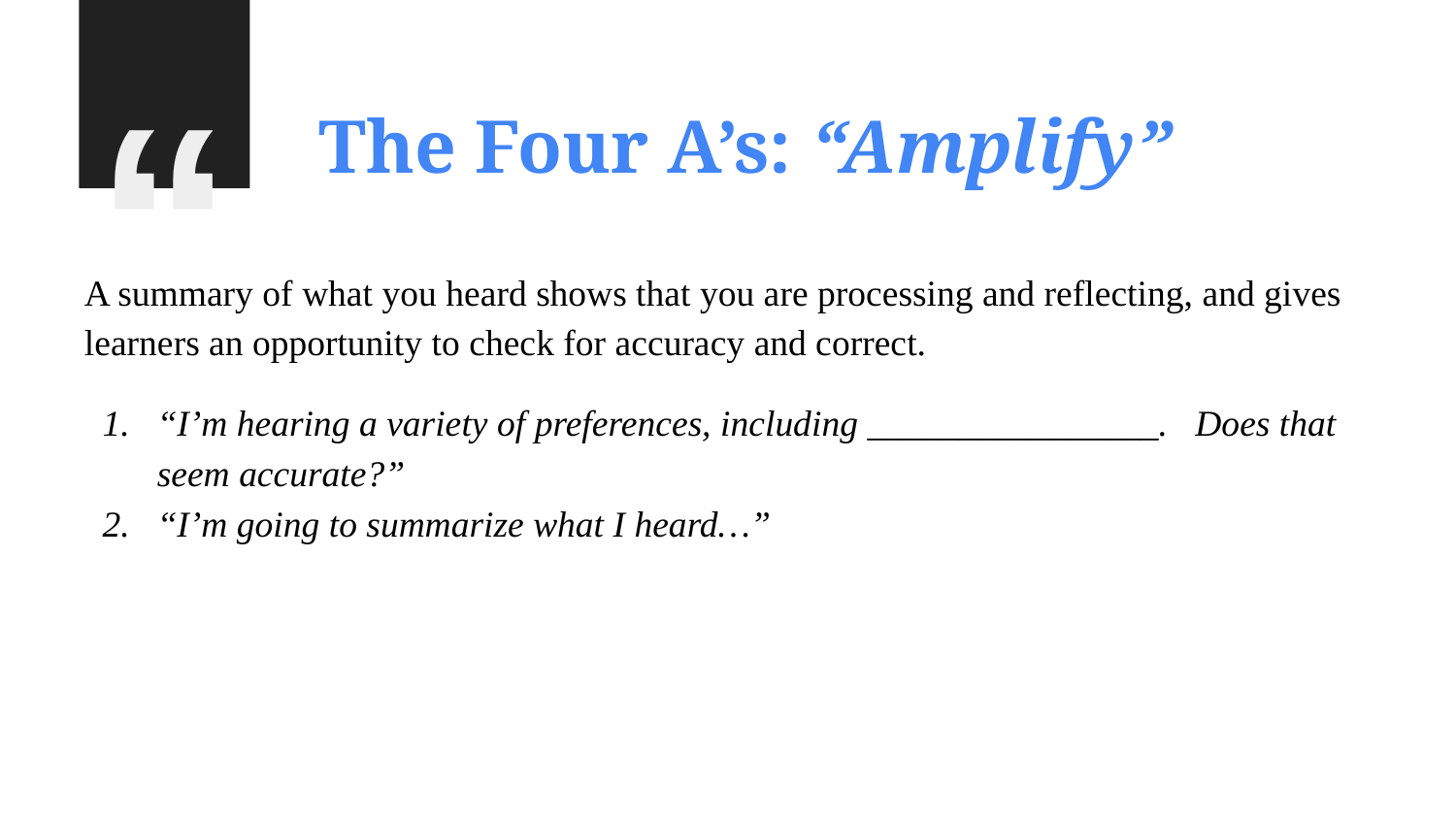

The Four A’s: “Amplify”
A summary of what you heard shows that you are processing and reflecting, and gives learners an opportunity to check for accuracy and correct.
“I’m hearing a variety of preferences, including ________________. Does that seem accurate?”
“I’m going to summarize what I heard…”

## Slide 25
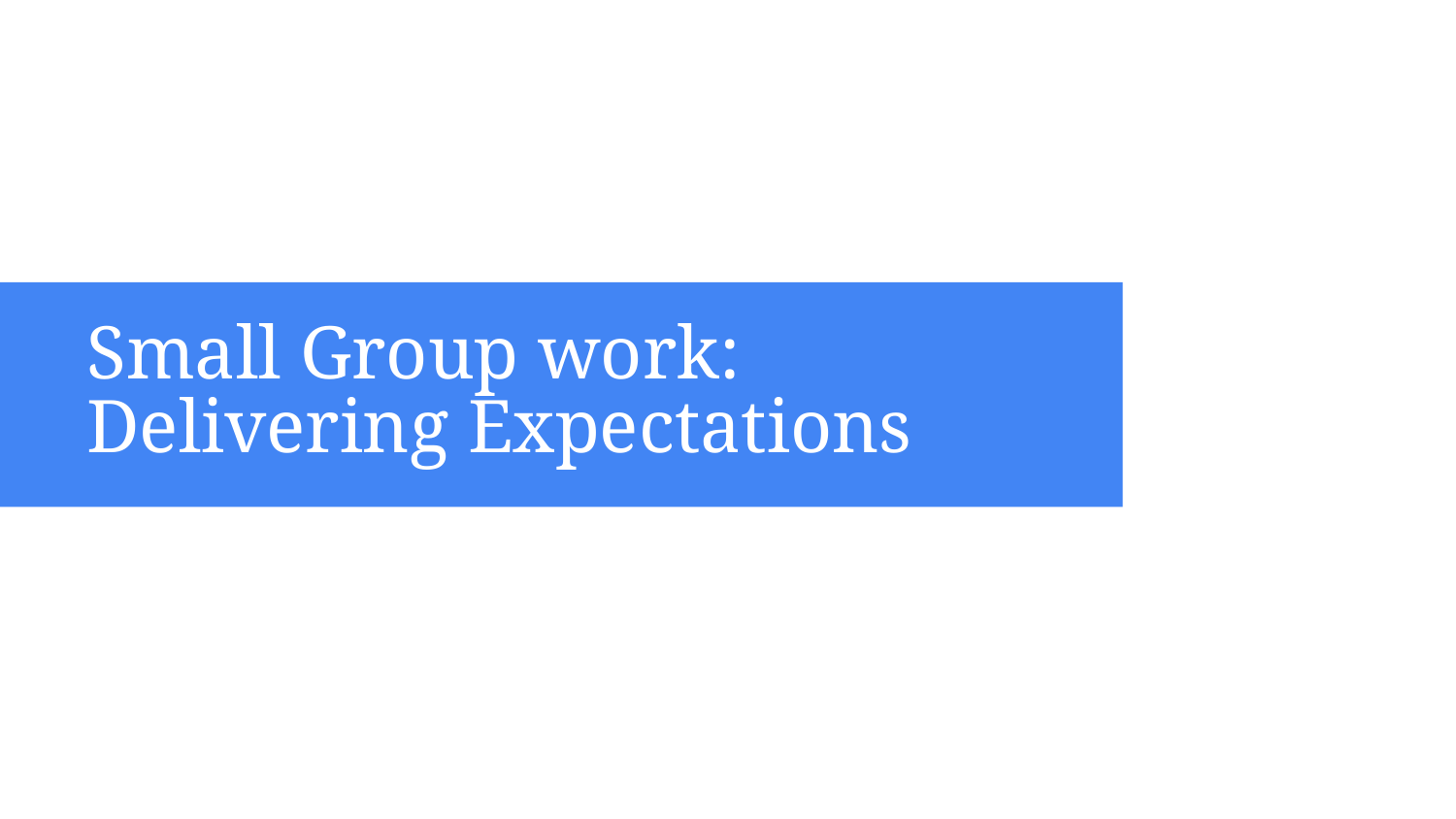

# Small Group work:
Delivering Expectations

## Slide 26
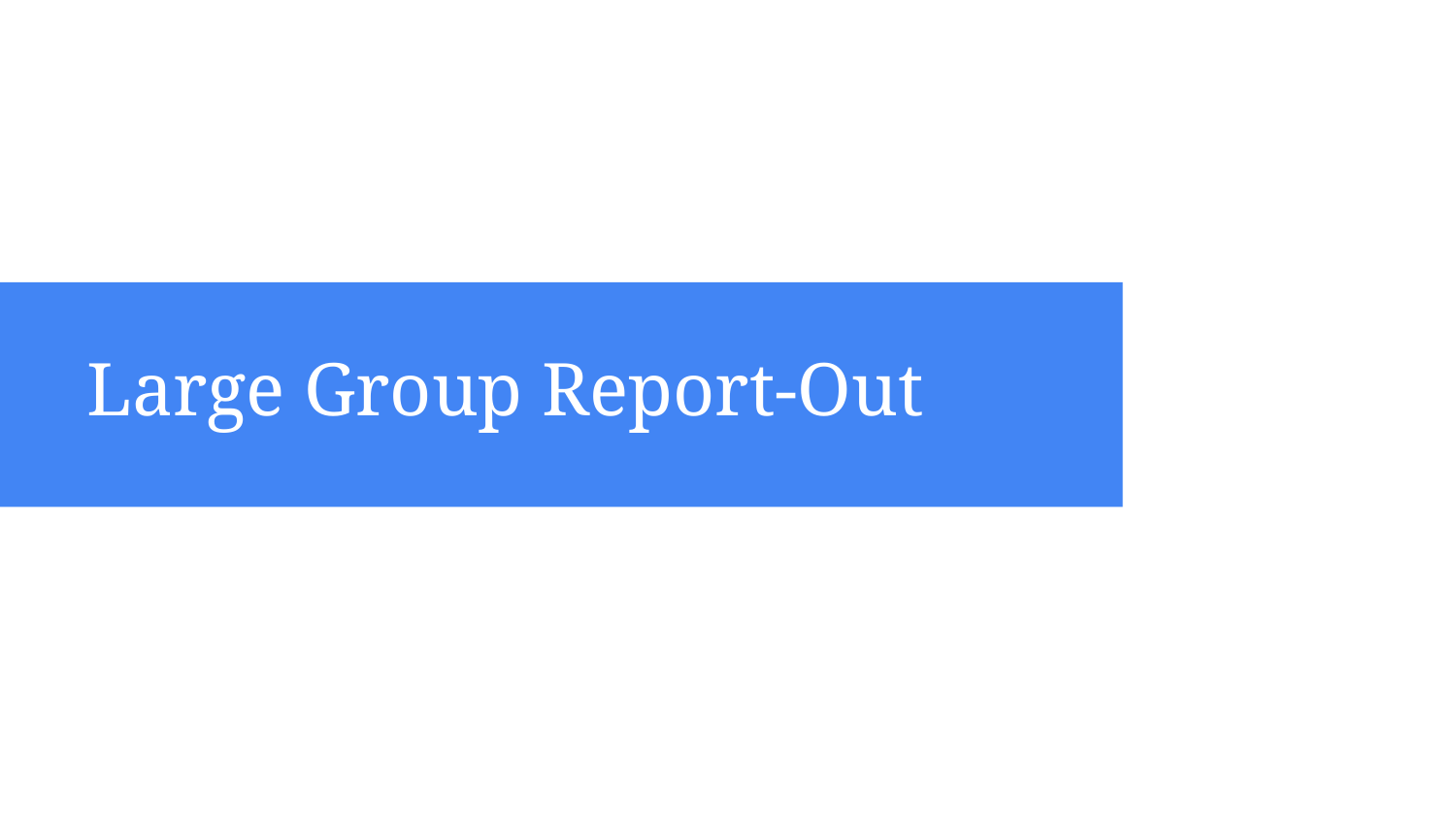

# Large Group Report-Out

## Slide 27
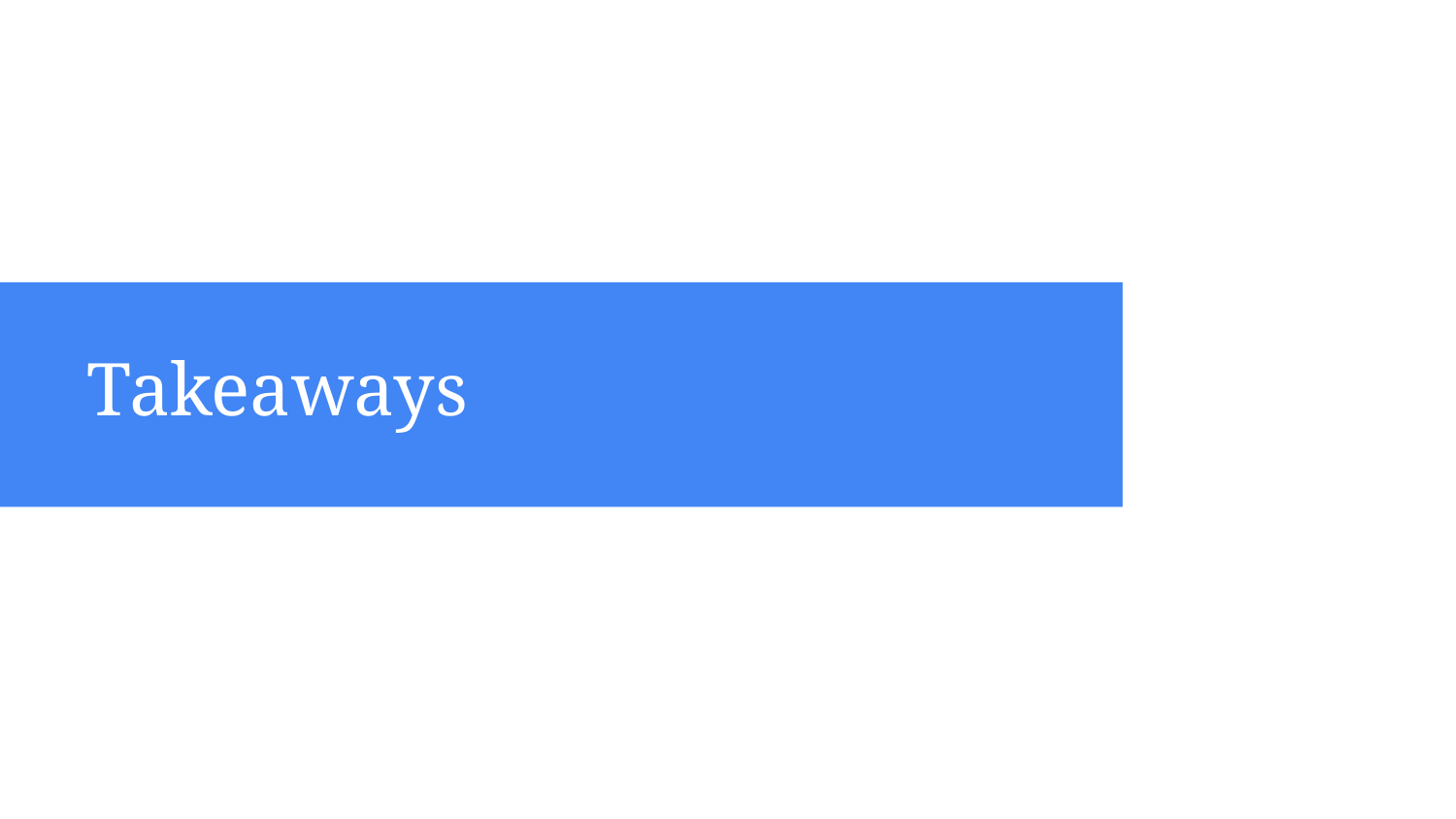

# Takeaways

## Slide 28
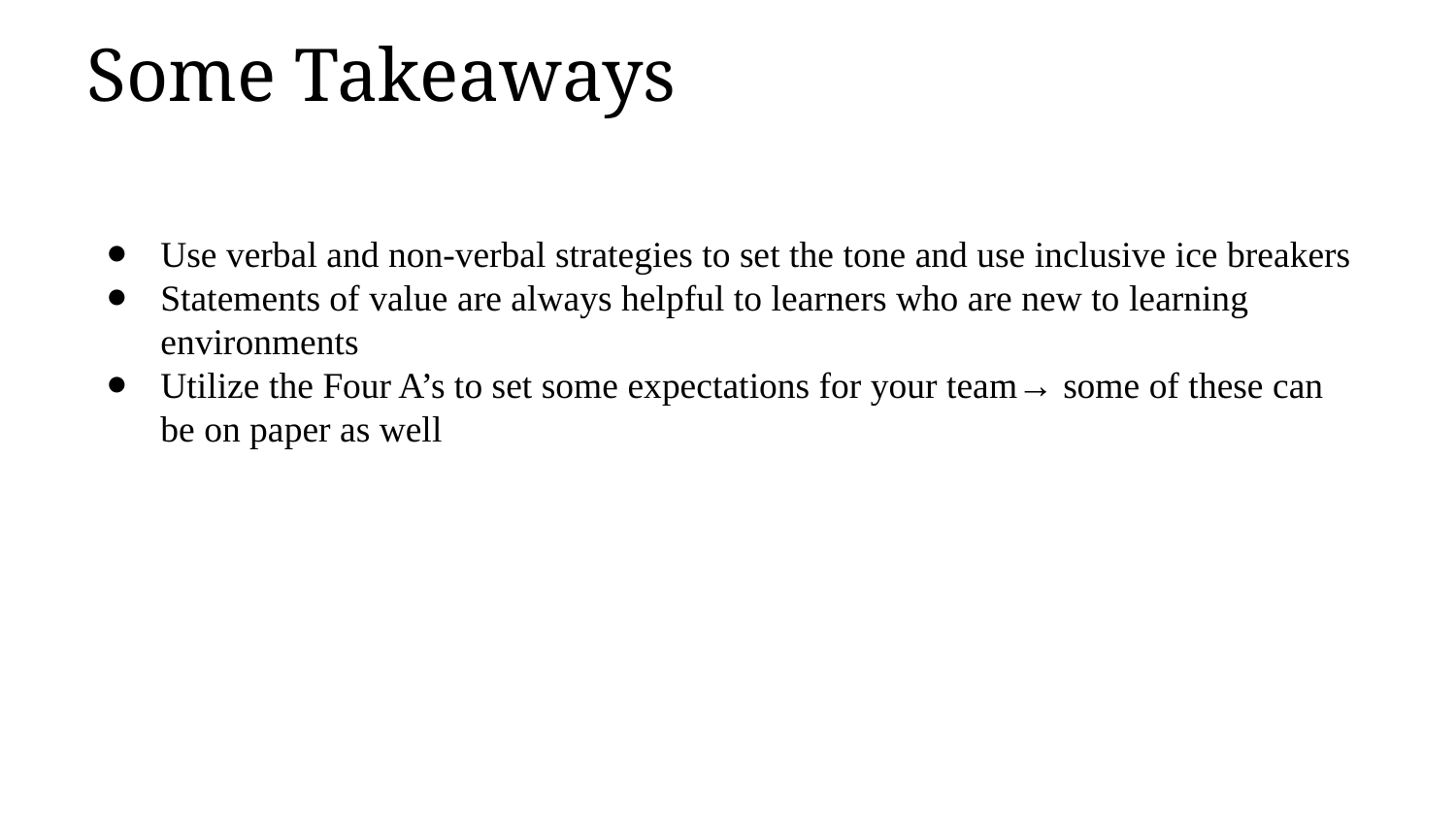

# Some Takeaways
Use verbal and non-verbal strategies to set the tone and use inclusive ice breakers
Statements of value are always helpful to learners who are new to learning environments
Utilize the Four A’s to set some expectations for your team→ some of these can be on paper as well

## Slide 29
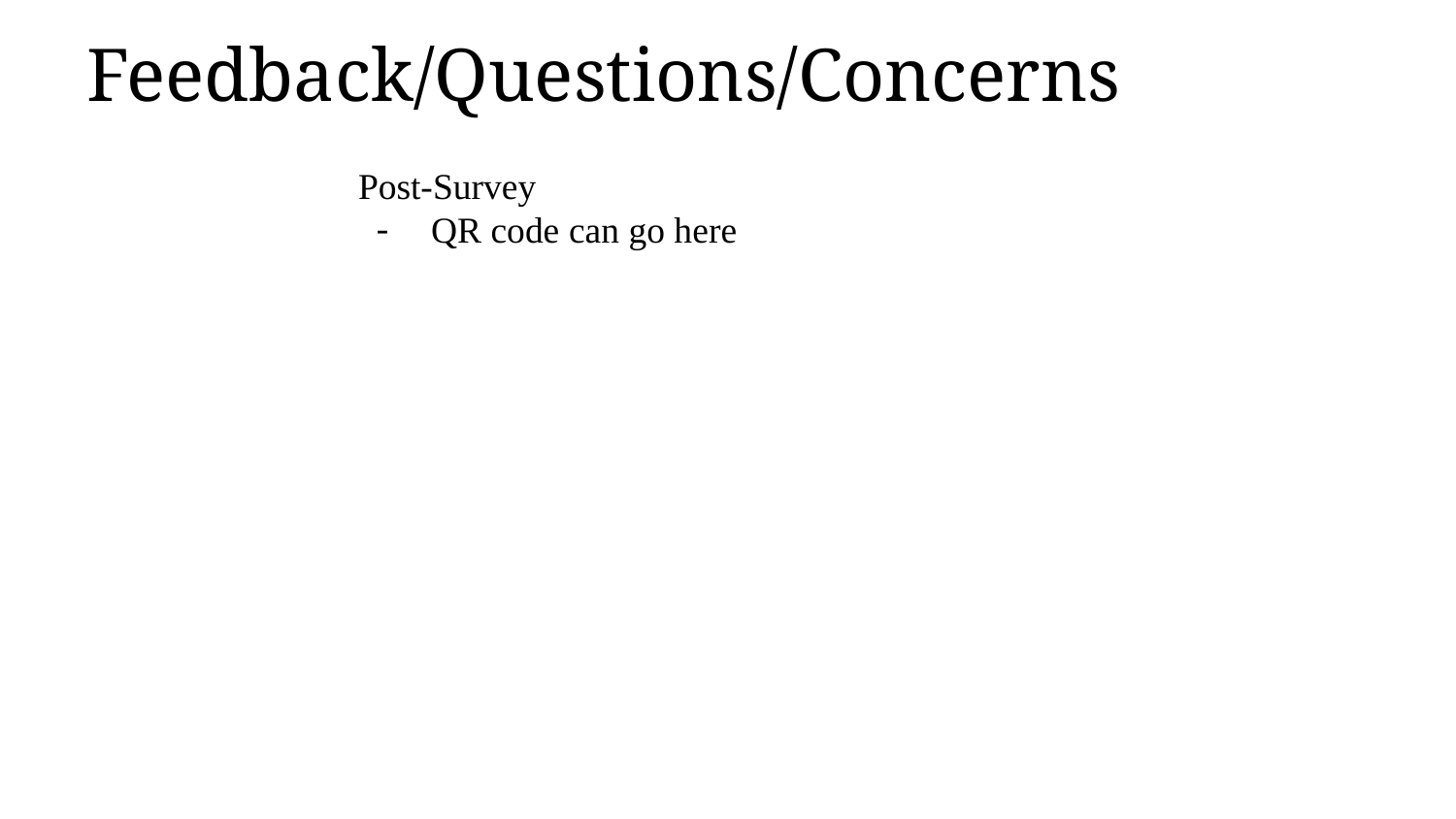

# Feedback/Questions/Concerns
Post-Survey
QR code can go here
