# Supplementary material for: Acknowledging and Addressing Microaggressions: A Virtual Experiential Learning Approach for Faculty
Source: MedEdPORTAL. 2024 Sep 4;20:11436. doi: 10.15766/mep_2374-8265.11436 (PMC11374130; doi:10.15766/mep_2374-8265.11436)
Supplement: Supplementary file 1 — Sample Flier.pptxWorkshop 1 - Slides.pptxWorkshop 1 - Facilitator GuideWorkshop 1 - Participant Handout.docxWorkshop 1 - Pre- and Postsurvey.docxWorkshop 2 - Slides.pptxWorkshop 2 - Facilitator Guide.docxWorkshop 2 - Participant Handout.docxWorkshop 2 - Pre- and Postsurvey.docxWorkshop 3 - Slides.pptxWorkshop 3 - Facilitator Guide.docxWorkshop 3 - Participant Handout.docxWorkshop 3 - Pre- and Postsurvey.docxWorkshop 4 - Slides.pptxWorkshop 4 - Facilitator Guide.docxWorkshop 4 - Participant Handout.docxWorkshop 4 - Pre- and Postsurvey.docx [file mep_2374-8265.11436-s001.zip › K. Workshop 3 - Facilitator Guide.docx]

**Faculty Microaggressions Curriculum**

**Setting Expectations for Learners/Trainees/Teams**

Workshop #3

Dates:

Time: 2 hours

Where:

Lead Facilitator::

This is the third of a four-part series developed for faculty leaders in Graduate Medical education surrounding the topic of microaggressions. In order to meet learners where they are, earlier workshops will focus on learning and expanding introductory skills surrounding microaggressions, and will lead gradually to more advanced skills such as apologizing to learners when harm has been experienced, setting expectations in the learning environment, and debriefing microaggressions experienced by learners.

Today, we will focus on setting expectations for the learners at the beginning of a rotation or learning encounters surrounding microaggressions. We will help develop a verbal and written script to utilize at the beginning of your time with the team.

This workshop is particularly useful for those who work frequently with trainees or medical students.

**Learning Objectives:**

To increase confidence and comfort surrounding the following skills:

1. Developing strategies for a warm welcome to learners before setting expectations
2. Composing a personalized set of expectations to disseminate to learners surrounding expectations when microaggressions are encountered

**Workshop Agenda:**

| **Time** | **Topic** | **Participants** |
| --- | --- | --- |
| 0:00 - 0:20 | Introductions/Group Agreements/Objectives | Large Group-Lead facilitator |
| 0:30 - 0:40 | Pair Share Reflections | Breakout rooms in pairs |
| 0:40 - 1:00 | Didactic with Questions | Large Group |
| 1:00- 1:10 | Stretch Break |  |
| 1:10 - 1:40 | Small Group Activity | Small Group Work, all facilitators |
| 1:40 - 1:50 | Large Group Report Out | Large Group- Lead facilitator |
| 1:50 - 2:00 | Wrap-Up/Takeaways | Large Group |

Lead facilitator for this workshop will begin the workshop with introduction of the workshop and community agreements. Participants will be invited to add additional agreements for the working session.

**Introductions/Community Agreements/Ice Breakers (20 minutes)**

Lead facilitator for this workshop will begin the session with introduction of the workshop and proposed community agreements. Participants voluntarily agree to this set of operational and behavioral agreements to build trust in this learning space and engage in productive work together. Participants will be invited to add additional agreements for the working session.

Community Agreements

•Respect each other as colleagues and humans

•Confidentiality

- Stories stay within our task force meetings, and lessons may leave the room. Especially when we are talking about specific cases related to our trainees and other divisional members/leaders

•Accountability

- We hold each other accountable for our words, actions, and impact
- We hold each other accountable for adhering to our group agreements

•Use “I” statements

- Our opinions and stories are our own, and we will not make blanket statements about others

•Impact versus intent

- We will recognize the difference between IMPACT and INTENT
- We will hold ourselves and others accountable by acknowledging IMPACT of words and actions when we see/feel/hear it

•Maximize the STRETCH ZONE!

- When we do this work, we are maximally efficient in the STRETCH ZONE, a brave space where we can (and should!) be uncomfortable, yet also be productive and learn
- When we label people and shame others, it puts people in the PANIC ZONE, and decreases buy-in to continue our mission

•Ask questions and invite other perspectives continually (i.e. humble inquiry)

- Replace the idea of perfection with a growth mindset, a lifelong journey of learning, effort, and persistence

•Prioritize self-care

- We understand that there may be stories, descriptions of events, and content that may be triggering or re-traumatizing to experience
- In a community of practice, we encourage care for our own mental health, in whatever sustainable form in which that takes shape
- We aim to be present in support of each other during challenging moments

Virtual Agreements can help create guidelines for operating in a virtual learning environment as well.

•Name/Pronouns/Role

- We invite participants to re-title their virtual presence with their name, pronunciation of name, pronouns (if comfortable sharing), and role as faculty

•Cameras on if possible

- To simulate in-person interactions, we invite participants to have cameras on throughout the educational workshop
- We understand that this is not possible based on physical location, background distractions, and internet access/bandwidth

•Mute when not speaking

- To encourage active listening and one speaker talking at a time, we promote the use of the mute button when not speaking
- This also makes closed-captions for accessibility more feasible

•Use the raise hand function to speak next

- This helps facilitate one speaker at a time and participants not speaking over each other, preventing miscommunication

•Feel free to use the chat and reactions

- For those who prefer written expression to verbal expression, this is an opportunity to engage and share perspectives

** It is important to try to include the learners in establishing these agreements. One tactic is collaborating together on coming up with specific categories of norms for conversations. Examples include brainstorming together norms for topics such as how to respect others and yourself, accepting challenge and discomfort, and keeping an open mind. It is also important that each participant agrees to the set of community agreements before moving forward with the work. This can be done virtually through a reaction, a chat agreement, or gestures in the virtual room.

Tools for further ideas:

<https://radcliffe-harvard-edu-prod.s3.amazonaws.com/8b8bef3c-2b23-4771-9847-625fc015adc4/LeveragingNormsforChallengingConversationsFINAL-ua.pdf>

<https://guidetoteaching.newschool.org/community-agreements/>

Following community agreements, each participant will have an opportunity to introduce themselves and the answer to an ice breaker:

*“ My name is ___________. I use ___________ pronouns. I work in the department of _______________________.*

*When I work with a new group of learners, I create a welcoming environment by __________.”*

A brief review of working definitions is then presented (these have all been presented in the first workshop).

Working Definitions

- **Microaggression-** brief and commonplace daily verbal, behavioral or environmental indignities (whether intentional or unintentional) that communicate hostile, derogatory, or negative slights and insults against ​a particular group of people. It is important to note that the term micro refers to interactions between individuals, not the impact on the individuals, which can be immense and feel very “macro” to recipients of microaggressions. The term was coined by Dr. Chester Pierce, a Harvard psychiatrist who became the founding president of Black Psychiatrists of America, and we pay respect to this incredible physician who brought public attention to the everyday racism faced in America in the 1960s (Williams, 2019).
- **Intersectionality-** the interconnected nature of social categorizations such as race, class, and gender, regarded as creating overlapping and interdependent systems of discrimination or disadvantage​
- **Allyship -** a lifelong process of building relationships based on trust, consistency, and accountability with marginalized individuals and/or groups of people

When teaching content related to diversity, equity, and inclusion (DEI), we commonly refer to the social ecological

model (Golden et al, 2020) of health, understanding that health outcomes and health disparities are a result of intrapersonal attitudes and interpersonal relationships, along with the institutions, systems, culture, and policies that surround us. Today we will focus on interpersonal relationships between faculty and learners.

**Pair Reflection Activity (10 minutes)**

Start with separating the group into pairs for a reflection surrounding relationships with learners.

.

*At the beginning of a rotation, how do you express your commitment to equity and inclusion?*

*How have you incorporated microaggressions into expectations?*

*What have been some successes and challenges?*

Large Group:

In the large group, facilitators will ask questions to participants about best practices. Then, they can offer other options we can use to make a student feel welcome in a new learning space.

- Personal connection→ a warm greeting or statement about their added value in the patients care or on the care team
- Interest in student interests→ learning about the student and their interests or lives
- Explicit roles→ their role as a learners and yours as a supporter, teacher, and supervisor
- Stories about mistakes in the clinical and learning environment

**Didactic with Questions: (20 minutes)**

**A Welcoming Setting**

**How can we set up a warm welcome:**

1. *An authentic welcome*

- Welcome to the team!
- It’s so great to meet you!
- Thank you for being part of this team!

1. *Small talk before big talk*

- How was your trip in today?
  - - How was your weekend?
    - Did you find your way to the team room okay? Did you find a place for your stuff?

**Introductions:**

- Introduce by names and pronouns
- Inclusive introduction icebreakers ( ex of inclusive v. exclusive)

**Examples:**

- 1. What’s your favorite comfort food?
  2. Tell us one thing we wouldn’t know just by looking at you!
  3. What’s your hidden talent/super power?
  4. If you could be an animal, what would you be and why?
  5. What is one goal you’d like to accomplish in your lifetime?
  6. Who is your hero and why?
  7. If you could visit any place in the world right now, where would you go and why?
  8. If a movie were made about your life, who would you want to play you?
  9. What’s your favorite way to relax or re-energize yourself?
  10. What color describes your mood right now?

Example of exclusionary ice breakers:

What was your last vacation (knowing that learners may not all be able to afford vacations and time away)?

What was your favorite gift you have received (some learners may not have received any gifts in their lifetime)?

Behaviors that help establish connection

- Interest in student interests- asking more in depth questions about a topic or interest of theirs
- Personal connection- affirming a value or thought of theirs in public
- Statements of partnership and growth- displaying interest in their growth and value
- Ask open ended questions and use active listening- asking genuine questions about their lives and backgrounds
- Sharing mistakes and anecdotes- can help build trust through vulnerability
- Facial cues, gestures, tone of voice. Note that neurodivergence plays a part in how these actions are perceived, so it’s important to be aware of responses and understand if these non-verbals require adjustment

**Delivering Expectations:**

We encourage attendings/team leaders to introduce the idea of responding to microaggressions **before the events even** **occur.**

This conversation can ideally happen at the beginning of the team formation (ie first day of the rotation/week)

1. **Setting the stage: [ACKNOWLEDGEMENT]**

The conversation can begin about problematic behavior in an open- ended manner.

The key is **acknowledgment.**

*I wish microaggressions didn’t occur in the learning environment. When they do occur, it can be harmful and uncomfortable. I am committed to supporting your well-being. I’d like to make sure that we all set some expectations for when we witness, receive or are the source of microaggressions.*

1. “I want to acknowledge that microaggressions happen. How can we work together to make sure this is a positive learning environment?”
2. “Microaggressions can be intentional or unintentional and have a negative impact. How will we address these as a team?”
3. “I would like to create an agreement prior to working together. What thoughts do you have about addressing microaggressions when they happen?”

*Does anyone else use a certain phrase, or have heard something they like?*

1. **Invite Learner’s Input [AGENCY]:**

Open-ended questions about how individuals on a team prefer to respond to patient microaggressions.

- - Some trainees prefer to address microaggressions themselves in the moment
  - Some learners may want to debrief only, and not intervene in the midst of a microaggression
  - Some learners may not want a response at all from you, and find support in other ways
  - Others prefer a response from more senior members of the team, especially surrounding patients or other members of the clinical environment

1. “I have learned that everyone has different needs when faced with a microaggression. What are your preferences?”
2. “I want to acknowledge that everyone has a different way of reacting and processing. If you have any preferences, I would love to know.”
3. “How will we acknowledge that we may not always have the right answer or at the right time?”

- Value of a debrief to discuss in depth
- Other resources that are available (through the school, healthcare system, etc.) to learners

1. **About yourself [ACCOUNTABILITY]:**

It is important to make clear that everyone is vulnerable to being the source of unintentional microaggressions and harm. In learning spaces of trust and respect, we hope that we can learn, grow, and be held accountable when harm occurs.

1. “We all have blind spots and will say things that could have an unintentional impact. I would like you to tell me if I say something that has a negative impact.”
2. “How else can we keep ourselves accountable when mistakes are made?”
3. “In learning spaces of trust and respect, we hope that we can learn, grow, and be held accountable when harm occurs. How will we discuss mistakes as a team?”
4. “There may be instances where I may be the source of a microaggression. I hope that the team will hold me accountable by…”
5. “What are ways we will name bias when we see it in the clinical environment?”

Ex. Last workshop on apologizing (in public, in person, email)

1. **Summary/Conclusion [AMPLIFY]:**

A summary of what you heard shows that you are processing and reflecting, and gives learners an opportunity to check for accuracy and correct..

1. “I’m hearing a variety of preferences, including ________________. Does that seem accurate?”
2. “I’m going to summarize what I heard…”

**Small Group Activity (30 minutes)**

In small groups of 3-4 participants, we will take some time to write out a script and then practice delivering expectations surrounding microaggressions in the learning environment.

**Large Group Reflection (10 minutes)**

What did you learn from doing this activity?

Where do you think you might implement this?

What do you foresee as still being challenges?

**Wrap-Up/Takeaways (10 minutes)**

- Use verbal and non-verbal strategies to set the tone and use inclusive ice breakers
- Statements of value are always helpful to learners who are new to learning environments
- Utilize the Four A’s to set some expectations for your team→ some of these can be on paper as well

References for this workshop:

1. Polk, W., & El-Amin, A. (2016). *Leveraging Norms for Challenging Conversations*. <https://radcliffe-harvard-edu-prod.s3.amazonaws.com/8b8bef3c-2b23-4771-9847-625fc015adc4/LeveragingNormsforChallengingConversationsFINAL-ua.pdf>
2. The New School. (2020). *Community Agreements*. Guide to teaching and learning. <https://guidetoteaching.newschool.org/community-agreements/>
3. Williams, M. T. (2019). Microaggressions: Clarification, evidence, and impact. *Perspectives on Psychological Science*, *15*(1), 3–26. <https://doi.org/10.1177/1745691619827499>
4. Golden, T. L., & Wendel, M. L. (2020). Public health’s next step in advancing equity: Re-evaluating epistemological assumptions to move social determinants from theory to practice. *Frontiers in Public Health*, *8*. <https://doi.org/10.3389/fpubh.2020.00131>
5. Aronson J, Burgess D, Phelan S, & Juarez L. Unhealthy interactions: the role of stereotype threat in health disparities. *Am J Public Health*. 2013;103:50-56.
6. Burgess D, van Ryn M, Dovidio J, & Saha S. Reducing racial bias among health care providers: Lessons learned from social-cognitive psychology. *SGIM*. 2007;22:882-887.
