# Supplementary material for: Acknowledging and Addressing Microaggressions: A Virtual Experiential Learning Approach for Faculty
Source: MedEdPORTAL. 2024 Sep 4;20:11436. doi: 10.15766/mep_2374-8265.11436 (PMC11374130; doi:10.15766/mep_2374-8265.11436)
Supplement: Supplementary file 1 — Sample Flier.pptxWorkshop 1 - Slides.pptxWorkshop 1 - Facilitator GuideWorkshop 1 - Participant Handout.docxWorkshop 1 - Pre- and Postsurvey.docxWorkshop 2 - Slides.pptxWorkshop 2 - Facilitator Guide.docxWorkshop 2 - Participant Handout.docxWorkshop 2 - Pre- and Postsurvey.docxWorkshop 3 - Slides.pptxWorkshop 3 - Facilitator Guide.docxWorkshop 3 - Participant Handout.docxWorkshop 3 - Pre- and Postsurvey.docxWorkshop 4 - Slides.pptxWorkshop 4 - Facilitator Guide.docxWorkshop 4 - Participant Handout.docxWorkshop 4 - Pre- and Postsurvey.docx [file mep_2374-8265.11436-s001.zip › L. Workshop 3 - Participant Handout.docx]

**Faculty Microaggressions Curriculum**

**Setting Expectations for Learners/Trainees/Teams**

**Skills Handout**

**Learning Objectives:**

To increase confidence and comfort surrounding the following skills:

1. Developing strategies for a warm welcome to learners before setting expectations
2. Composing a personalized set of expectations to disseminate to learners surrounding expectations when microaggressions are encountered

**Pair Reflection for Participants:**

*At the beginning of a rotation, how do you express your commitment to equity and inclusion?*

*How have you incorporated microaggressions into expectations?*

*What have been some successes and challenges?*

**Ways to open team discussions:**

**1. A welcoming setting:**

1. *An authentic welcome*

- Welcome to the team!
- It’s so great to meet you!
- Thank you for being part of this team!

1. *Small talk before big talk*

- How was your trip in today?
  - - How was your weekend?
    - Did you find your way to the team room okay? Did you find a place for your stuff?

1. *Interest in student interests→ learning about the student and their interests or lives*

- Tell me more about…
- I heard that you like… what was that experience like?
- That sounds so exciting that you… how has that been?

1. *Value added→ a statement about their added value in the patients care or on the care team*

- You are going to be an invaluable member of our team in the next few weeks…
- I appreciate you joining me for the week and taking care of our patients…
- I am excited for the opportunity to learn and grow with you…

**2. Introduction:**

1. *Introduce names and pronouns*
2. *Inclusive ice breakers*
   1. What’s your favorite comfort food?
   2. Tell us one thing we wouldn’t know just by looking at you!
   3. What’s your hidden talent/super power?
   4. If you could be an animal, what would you be and why?
   5. What is one goal you’d like to accomplish in your lifetime?
   6. Who is your hero and why?
   7. If you could visit any place in the world right now, where would you go and why?
   8. If a movie were made about your life, who would you want to play you?
   9. What’s your favorite way to relax or re-energize yourself?
   10. What color describes your mood right now?
   11. What is your definition of home?

**3. Behaviors that help establish connection**

- Interest in student interests- asking more in depth questions about a topic or interest of theirs
- Personal connection- affirming a value or thought of theirs in public
- Statements of partnership and growth- displaying interest in their growth and value
- Ask open ended questions and use active listening- asking genuine questions about their lives and backgrounds
- Sharing mistakes and anecdotes- can help build trust through vulnerability
- Non-verbal cues: Facial cues, gestures, tone of voice. Note that neurodivergence plays a part in how these actions are perceived, so it’s important to be aware of responses and understand if these non-verbals require adjustment

We encourage attendings/team leaders to introduce the idea of responding to microaggressions **before the events even** **occur.** Ideally should be a conversation and happen at the beginning of your time together.

***The Four A’s* for setting up a team expectations:**

**1. Setting the stage: [ACKNOWLEDGEMENT]**

The conversation can begin about problematic behavior in an open- ended manner. The key is **acknowledgment.**

“I wish microaggressions didn’t occur in the learning environment. When they do occur, it can be harmful and uncomfortable. I am committed to supporting your well-being. I’d like to make sure that we all set some expectations for when we witness, receive or are the source of microaggressions.”

1. “I want to acknowledge that microaggressions happen. How can we work together to make sure this is a positive learning environment?”
2. “Microaggressions can be intentional or unintentional and have a negative impact. How will we address these as a team?”
3. “I would like to create an agreement prior to working together. What thoughts do you have about addressing microaggressions when they happen?”

**2. Invite Learner’s Input [AGENCY]:**

Open-ended questions about how individuals on a team prefer to respond to patient microaggressions.

- - Some trainees prefer to address microaggressions themselves in the moment
  - Some learners may want to debrief only, and not intervene in the midst of a microaggression
  - Some learners may not want a response at all from you, and find support in other ways
  - Others prefer a response from more senior members of the team, especially surrounding patients or other members of the clinical environment

1. “I have learned that everyone has different needs when faced with a microaggression. What are your preferences?”
2. “I want to acknowledge that everyone has a different way of reacting and processing. If you have any preferences, I would love to know.”
3. “How will we acknowledge that we may not always have the right answer or at the right time?”

- Value of a debrief to discuss in depth
- Other resources that are available (through the school, healthcare system, etc.) to learners

**3. About yourself [ACCOUNTABILITY]:**

It is important to make clear that everyone is vulnerable to being the source of unintentional microaggressions and harm. In learning spaces of trust and respect, we hope that we can learn, grow, and be held accountable when harm occurs.

1. “We all have blind spots and will say things that could have an unintentional impact. I would like you to tell me if I say something that has a negative impact.”
2. “How else can we keep ourselves accountable when mistakes are made?”
3. “In learning spaces of trust and respect, we hope that we can learn, grow, and be held accountable when harm occurs. How will we discuss mistakes as a team?”
4. “There may be instances where I may be the source of a microaggression. I hope that the team will hold me accountable by…”
5. “What are ways we will name bias when we see it in the clinical environment?”

**4. Summary/Conclusion [AMPLIFY]:**

A summary of what you heard shows that you are processing and reflecting, and gives learners an opportunity to check for accuracy and correct..

1. “I’m hearing a variety of preferences, including ________________. Does that seem accurate?”
2. “I’m going to summarize what I heard…”

Practice space for your own written/verbal expectations:
