# Supplementary material for: Acknowledging and Addressing Microaggressions: A Virtual Experiential Learning Approach for Faculty
Source: MedEdPORTAL. 2024 Sep 4;20:11436. doi: 10.15766/mep_2374-8265.11436 (PMC11374130; doi:10.15766/mep_2374-8265.11436)
Supplement: Supplementary file 1 — Sample Flier.pptxWorkshop 1 - Slides.pptxWorkshop 1 - Facilitator GuideWorkshop 1 - Participant Handout.docxWorkshop 1 - Pre- and Postsurvey.docxWorkshop 2 - Slides.pptxWorkshop 2 - Facilitator Guide.docxWorkshop 2 - Participant Handout.docxWorkshop 2 - Pre- and Postsurvey.docxWorkshop 3 - Slides.pptxWorkshop 3 - Facilitator Guide.docxWorkshop 3 - Participant Handout.docxWorkshop 3 - Pre- and Postsurvey.docxWorkshop 4 - Slides.pptxWorkshop 4 - Facilitator Guide.docxWorkshop 4 - Participant Handout.docxWorkshop 4 - Pre- and Postsurvey.docx [file mep_2374-8265.11436-s001.zip › M. Workshop 3 - Pre- and Postsurvey.docx]

**Microaggressions Workshop #3: *Setting Expectations for Learners/Trainees/Teams* PRE-SURVEY**

**Do you consent to using your responses as part of the research surrounding this work? ?**

_____ **YES**, you may use my responses in the research study.

_____ **NO**, you may NOT use my responses in the research study.

**What is your participant ID?** _______________ **(only asked if individual participates in study)**

2-digit birth**DAY** + last 2 letters of birth **CITY** + first initial of each **PARENT** in alphabetical order (use X if unknown)

E.g., Participant ID for a person born on July **09** in Tope**ka** whose parents are **K**yle and **S**am is **09KAKS**

**Demographic Information**

1. **My GME affiliated role is in the department of ____________**
2. **What is your race/ethnicity? (check all that apply)**

_____ American Indian or Alaska Native _____ White

_____ Asian _____ Multi-race/Ethnicity

_____ Black or African American _____ Unknown/Prefer not to say

_____ LatinX _____ Other/self-describe: _______

_____ Native Hawaiian or Other Pacific Islander

1. **What gender do you identify with?**

_____ Female _____ Other (please specify): _______

_____ Male ____ Prefer not to state

_____ Transgender female

_____ Transgender male

_____ Genderqueer/gender non-conforming

1. **Other identities that I hold related to my leadership/teaching role (free text):**
2. **Previous workshops I have participated in include: (Drop down boxes)**
3. **Acknowledging and Naming Microaggressions: Virtual 8/30**
4. **Acknowledging and Naming Microaggressions: Virtual 9/20**
5. **Apologizing when you have done harm: Virtual 10/11**
6. **Apologizing when you have done harm: Virtual 11/10**
7. **This is my first workshop**

**6. I prefer to participate in these workshops:**

1. **In person**
2. **Virtually**

**To what extent do you agree with these statements? (check one per row)**

| **Setting Expectation** | **Strongly Disagree** | **Disagree** | **Neutral** | **Agree** | **Strongly Agree** |
| --- | --- | --- | --- | --- | --- |
| It is important to incorporate expectations surrounding microaggressions in the learning environment with new teams/trainees. |  |  |  |  |  |
| It is important for learners to feel welcome upon entering a new learning environment |  |  |  |  |  |
| It is important to create an inclusive environment for all learners |  |  |  |  |  |
| **Effective communication with expectations** | **Strongly Disagree** | **Disagree** | **Neutral** | **Agree** | **Strongly Agree** |
| I am comfortable creating my own set of expectations surrounding microaggressions |  |  |  |  |  |
| I am confident in my ability to deliver expectations surrounding microaggressions in the learning environment with new learners. |  |  |  |  |  |
| I am confident in my ability to create a welcoming environment when meeting new learners. |  |  |  |  |  |
| I am confident in my ability to create an inclusive environment when meeting new learners. |  |  |  |  |  |

**My biggest barriers to setting expectations surrounding microaggressions in the learning environment with new teams or trainees include:**

**Microaggressions Workshop #3: *Setting Expectations for Learners/Trainees/Teams* POST-SURVEY**

**To what extent do you agree with these statements? (check one per row)**

| **Setting Expectations** | **Strongly Disagree** | **Disagree** | **Neutral** | **Agree** | **Strongly Agree** |
| --- | --- | --- | --- | --- | --- |
| It is important to incorporate expectations surrounding microaggressions in the learning environment with new teams/trainees. |  |  |  |  |  |
| It is important for learners to feel welcome upon entering a new learning environment |  |  |  |  |  |
| It is important to create an inclusive environment for all learners |  |  |  |  |  |
| **Effective communication with expectations** | **Strongly Disagree** | **Disagree** | **Neutral** | **Agree** | **Strongly Agree** |
| I am comfortable creating my own set of expectations surrounding microaggressions |  |  |  |  |  |
| I am confident in my ability to deliver expectations surrounding microaggressions in the learning environment with new learners. |  |  |  |  |  |
| I am confident in my ability to create a welcoming environment when meeting new learners. |  |  |  |  |  |
| I am confident in my ability to create an inclusive environment when meeting new learners. |  |  |  |  |  |

1. What was the **most useful** part of this workshop? Why?
2. What was the **least useful** part of this workshop? Why?
3. What would you **change** about this workshop? Why?
4. Please feel free to offer feedback to your facilitators. Did they create an inclusive learning environment? What did they do well? What could they do better?
5. Commit to one personal change you will make to create a more inclusive learning environment after this workshop:
