# Supplementary material for: Acknowledging and Addressing Microaggressions: A Virtual Experiential Learning Approach for Faculty
Source: MedEdPORTAL. 2024 Sep 4;20:11436. doi: 10.15766/mep_2374-8265.11436 (PMC11374130; doi:10.15766/mep_2374-8265.11436)
Supplement: Supplementary file 1 — Sample Flier.pptxWorkshop 1 - Slides.pptxWorkshop 1 - Facilitator GuideWorkshop 1 - Participant Handout.docxWorkshop 1 - Pre- and Postsurvey.docxWorkshop 2 - Slides.pptxWorkshop 2 - Facilitator Guide.docxWorkshop 2 - Participant Handout.docxWorkshop 2 - Pre- and Postsurvey.docxWorkshop 3 - Slides.pptxWorkshop 3 - Facilitator Guide.docxWorkshop 3 - Participant Handout.docxWorkshop 3 - Pre- and Postsurvey.docxWorkshop 4 - Slides.pptxWorkshop 4 - Facilitator Guide.docxWorkshop 4 - Participant Handout.docxWorkshop 4 - Pre- and Postsurvey.docx [file mep_2374-8265.11436-s001.zip › N. Workshop 4 - Slides.pptx]

## Slide 1
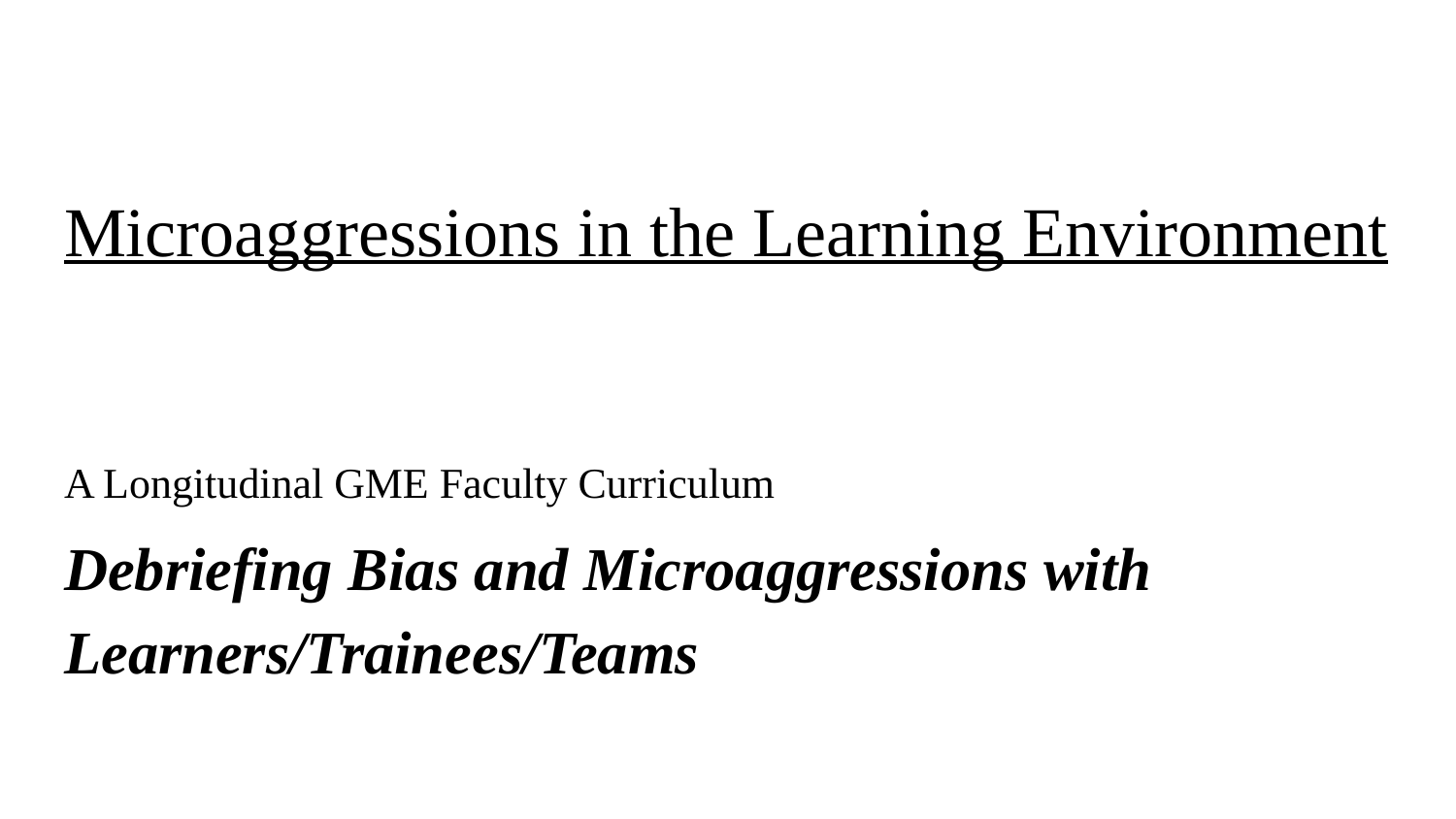

# Microaggressions in the Learning Environment
A Longitudinal GME Faculty Curriculum
Debriefing Bias and Microaggressions with Learners/Trainees/Teams

## Slide 2
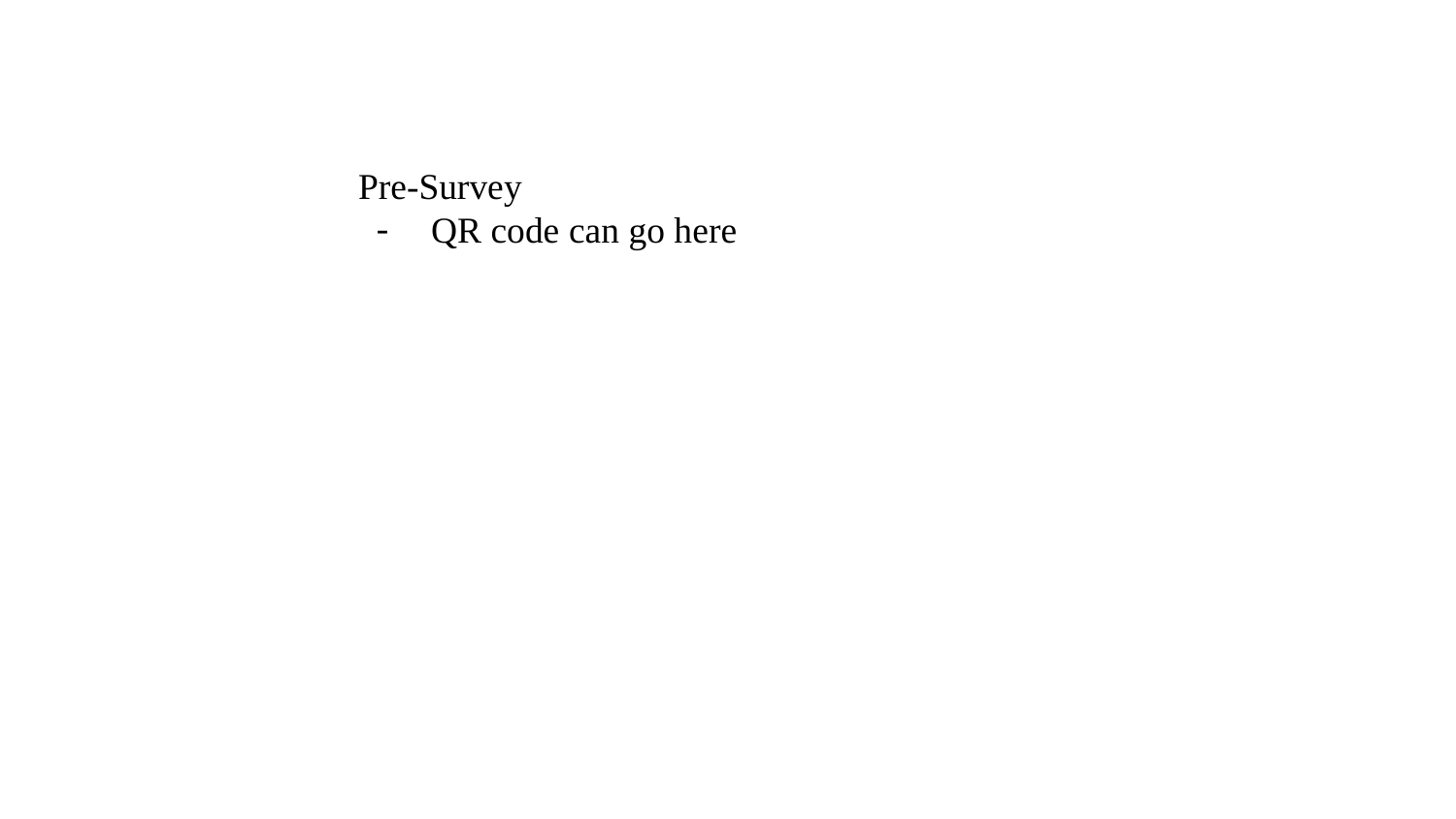

Pre-Survey
QR code can go here

## Slide 3
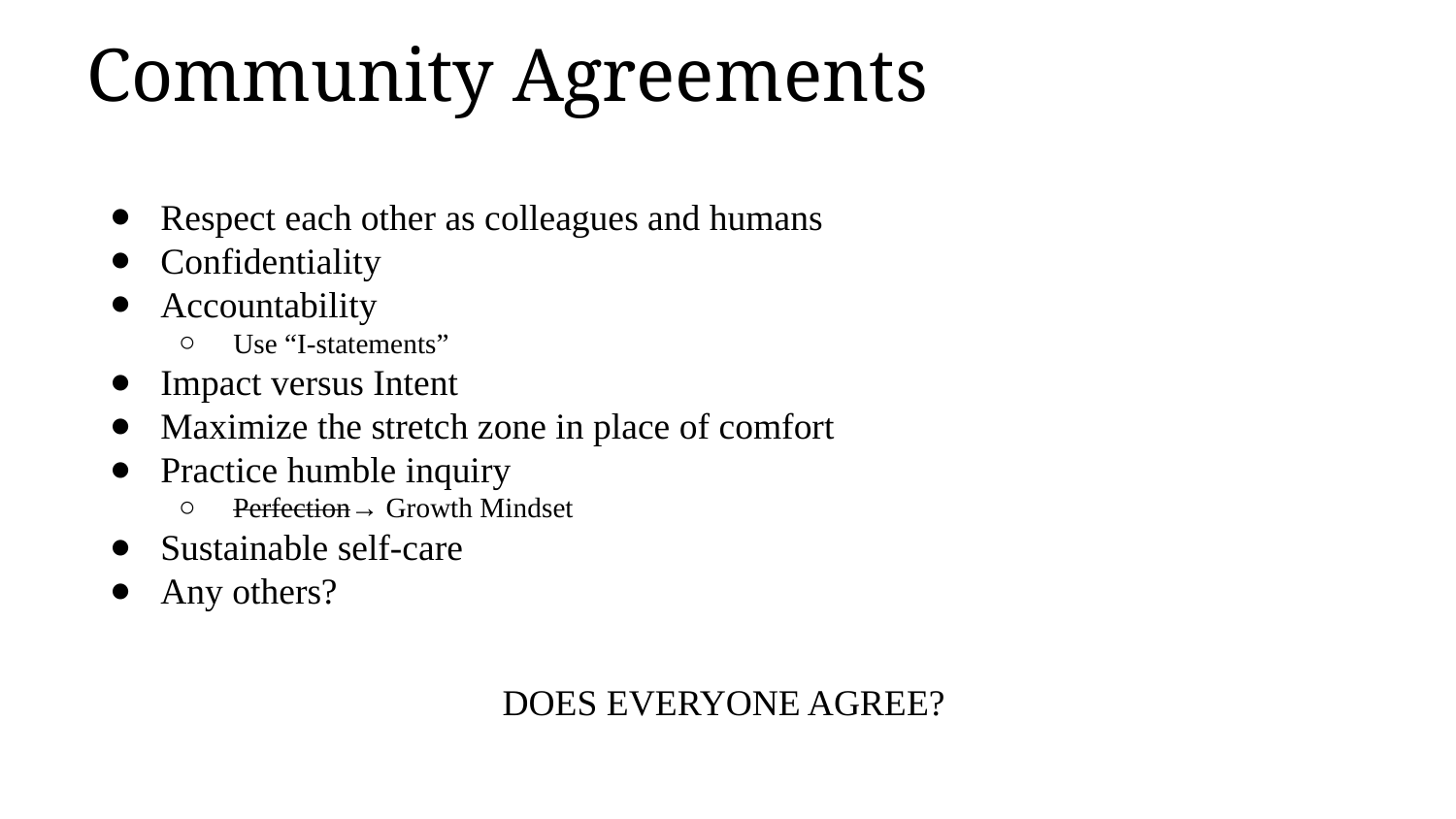

# Community Agreements
Respect each other as colleagues and humans
Confidentiality
Accountability
Use “I-statements”
Impact versus Intent
Maximize the stretch zone in place of comfort
Practice humble inquiry
Perfection→ Growth Mindset
Sustainable self-care
Any others?
DOES EVERYONE AGREE?

## Slide 4
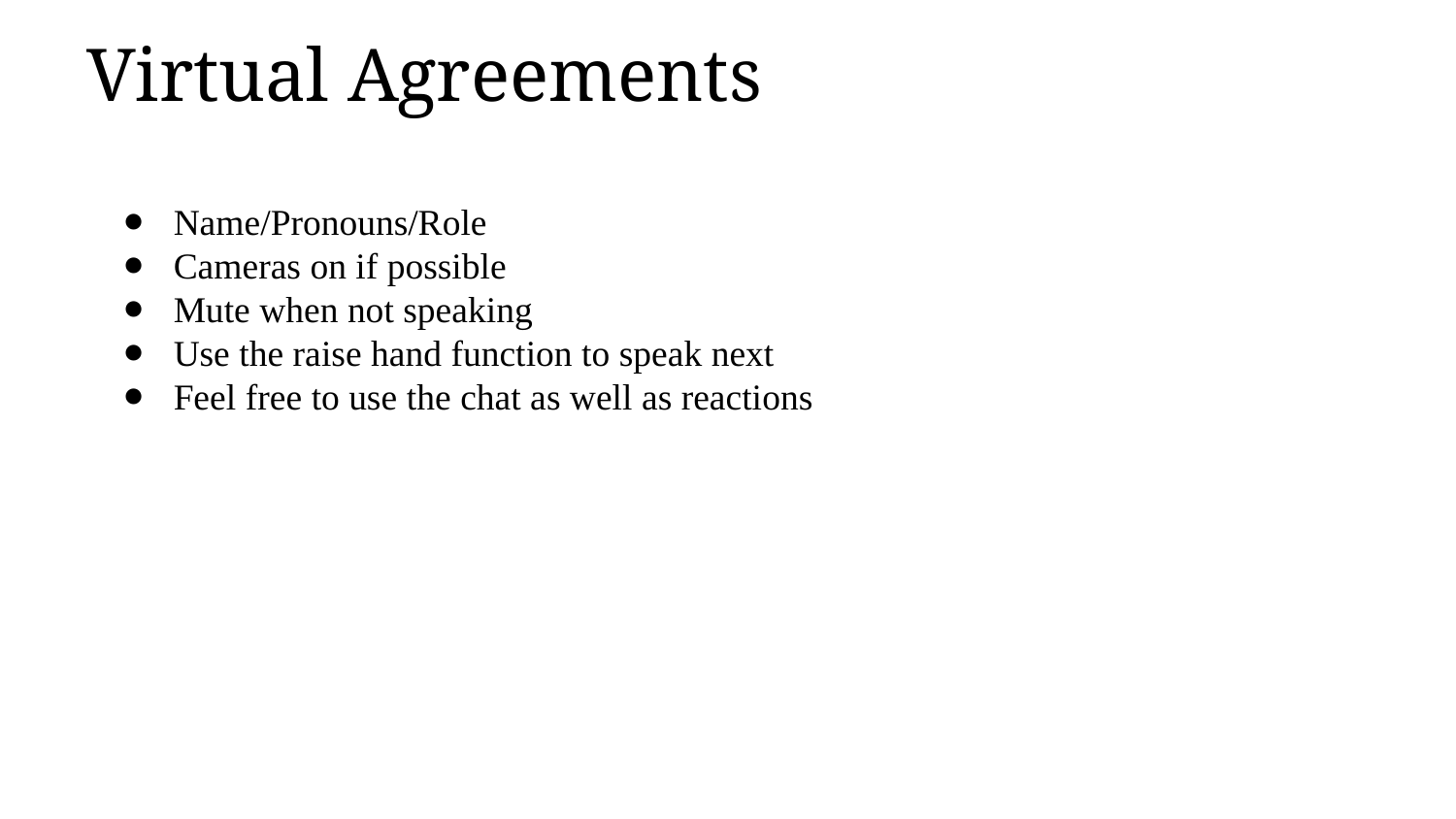

# Virtual Agreements
Name/Pronouns/Role
Cameras on if possible
Mute when not speaking
Use the raise hand function to speak next
Feel free to use the chat as well as reactions

## Slide 5
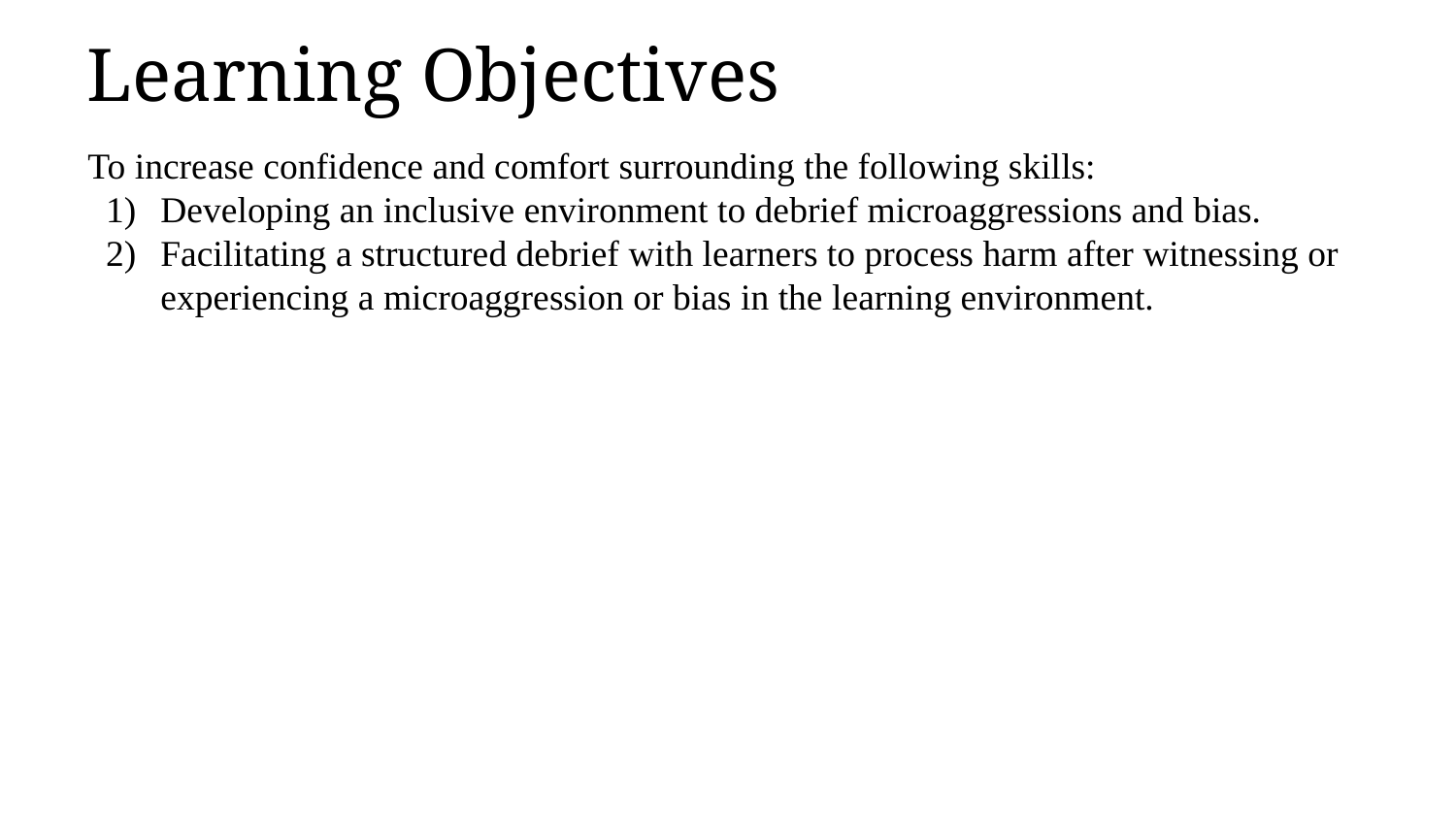

# Learning Objectives
To increase confidence and comfort surrounding the following skills:
Developing an inclusive environment to debrief microaggressions and bias.
Facilitating a structured debrief with learners to process harm after witnessing or experiencing a microaggression or bias in the learning environment.

## Slide 6
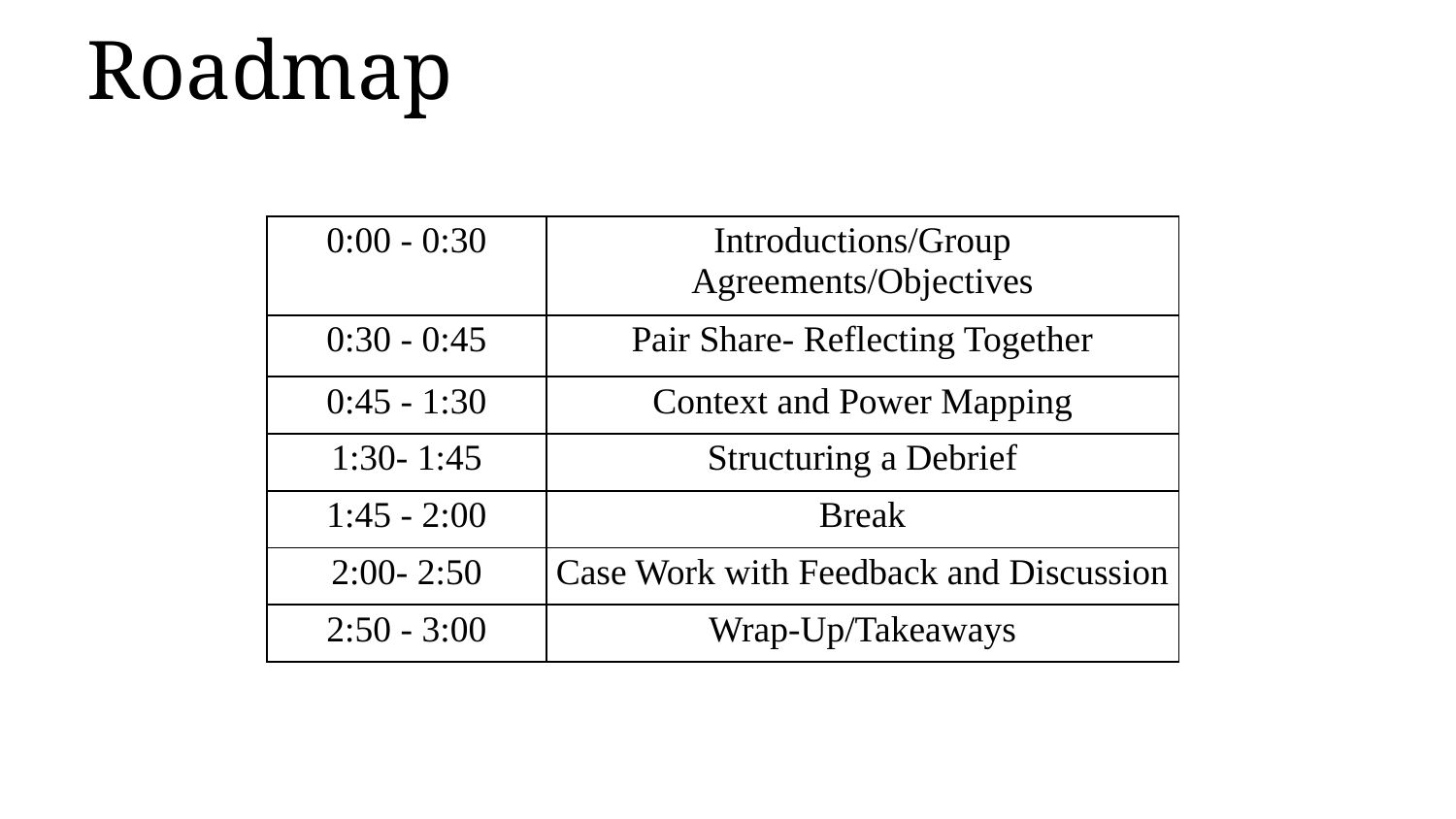

# Roadmap
| 0:00 - 0:30 | Introductions/Group Agreements/Objectives |
| --- | --- |
| 0:30 - 0:45 | Pair Share- Reflecting Together |
| 0:45 - 1:30 | Context and Power Mapping |
| 1:30- 1:45 | Structuring a Debrief |
| 1:45 - 2:00 | Break |
| 2:00- 2:50 | Case Work with Feedback and Discussion |
| 2:50 - 3:00 | Wrap-Up/Takeaways |

## Slide 7
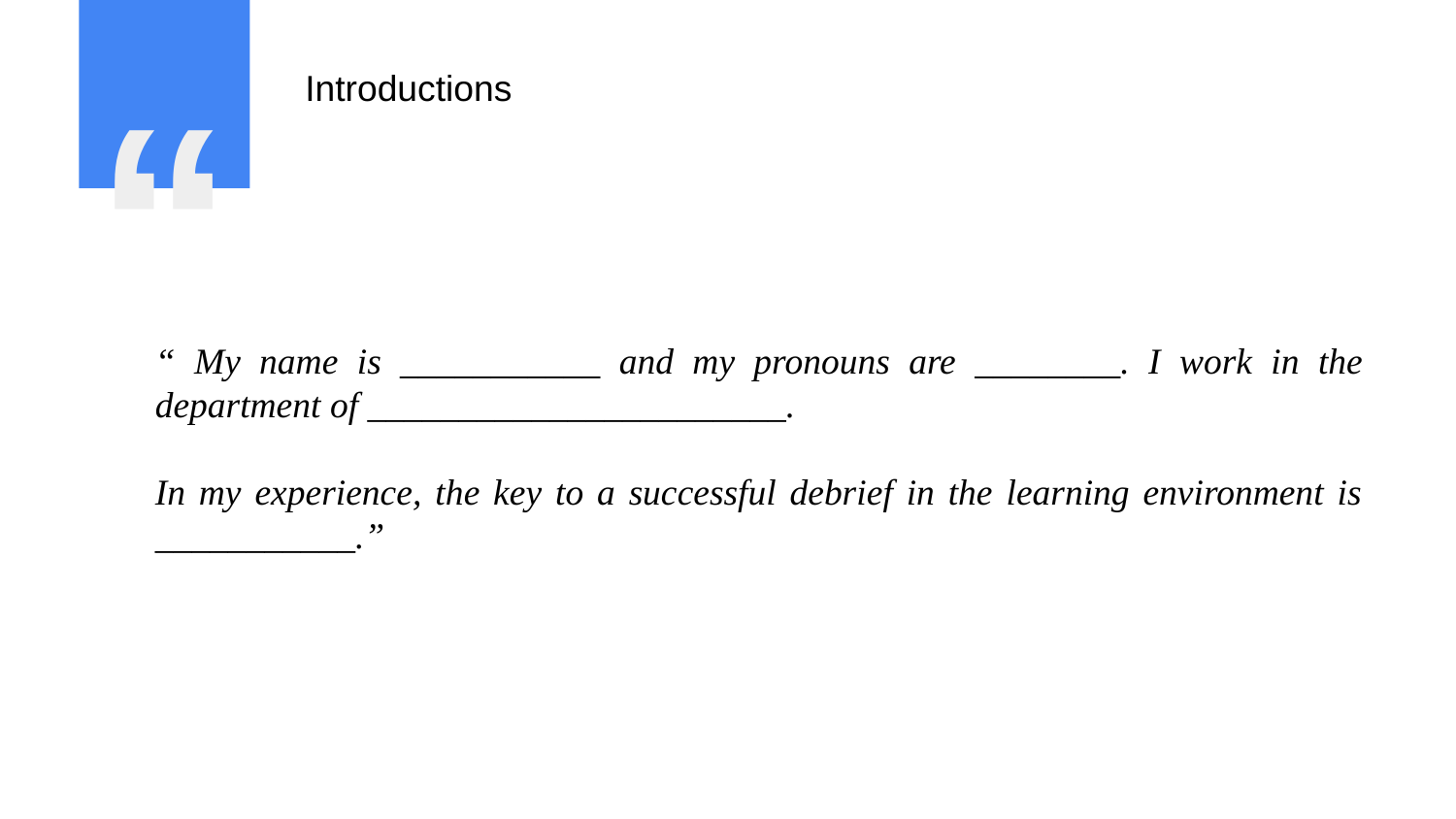

Introductions
“ My name is ___________ and my pronouns are ________. I work in the department of _______________________.
In my experience, the key to a successful debrief in the learning environment is ___________.”

## Slide 8
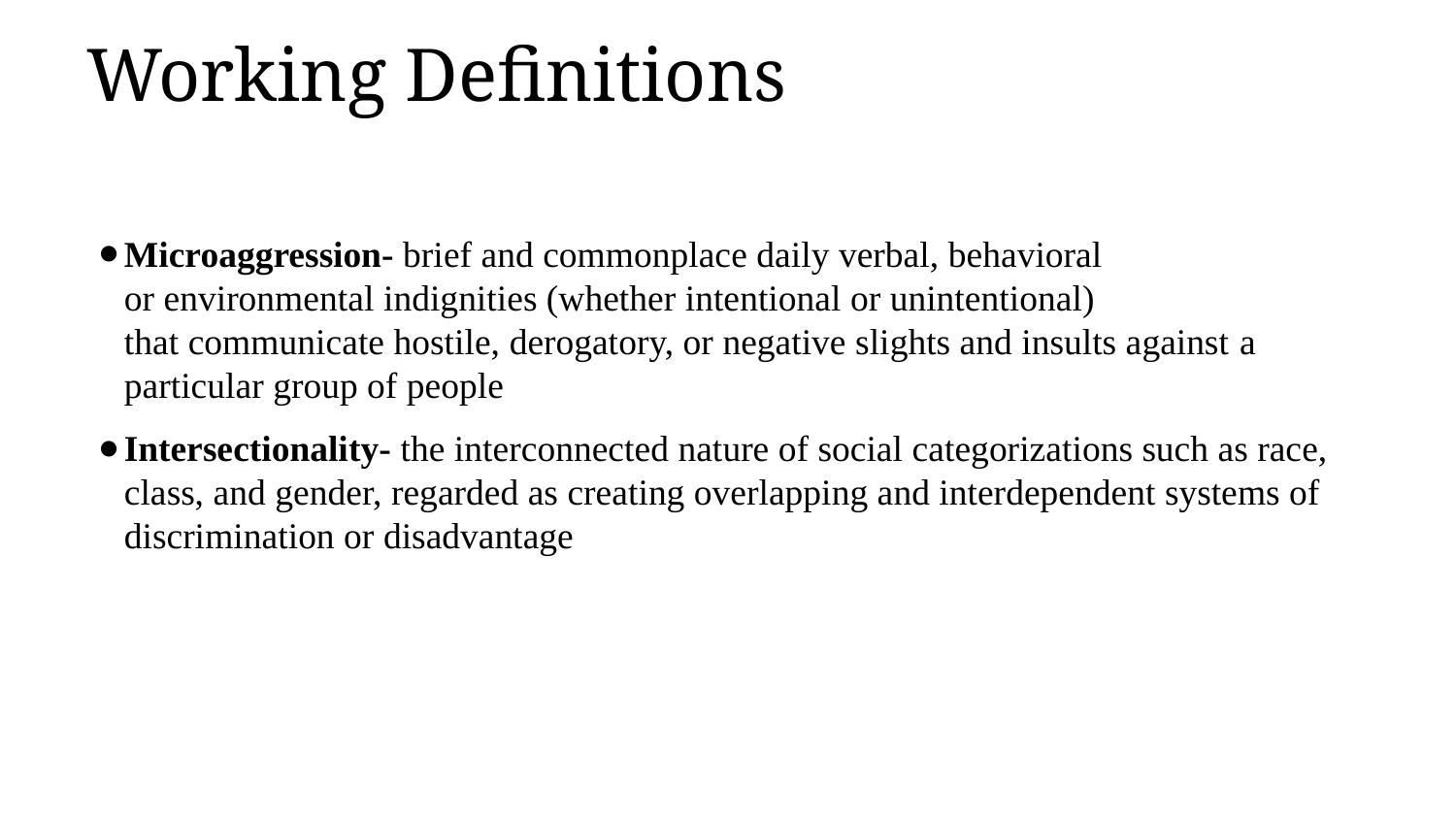

# Working Definitions
Microaggression- brief and commonplace daily verbal, behavioral or environmental indignities (whether intentional or unintentional) that communicate hostile, derogatory, or negative slights and insults against ​a particular group of people
Intersectionality- the interconnected nature of social categorizations such as race, class, and gender, regarded as creating overlapping and interdependent systems of discrimination or disadvantage​

## Slide 9
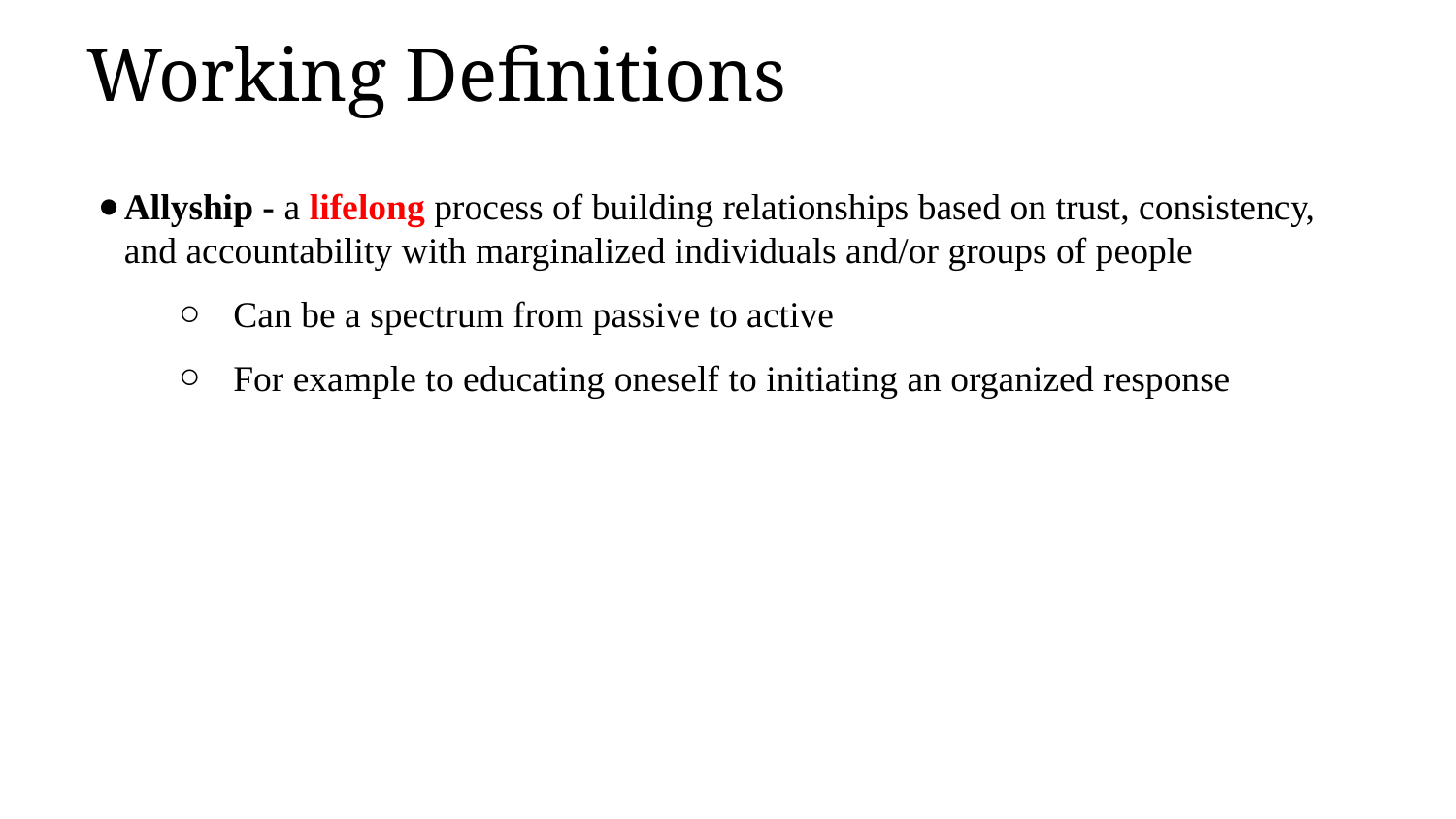

# Working Definitions
Allyship - a lifelong process of building relationships based on trust, consistency, and accountability with marginalized individuals and/or groups of people
Can be a spectrum from passive to active
For example to educating oneself to initiating an organized response

## Slide 10
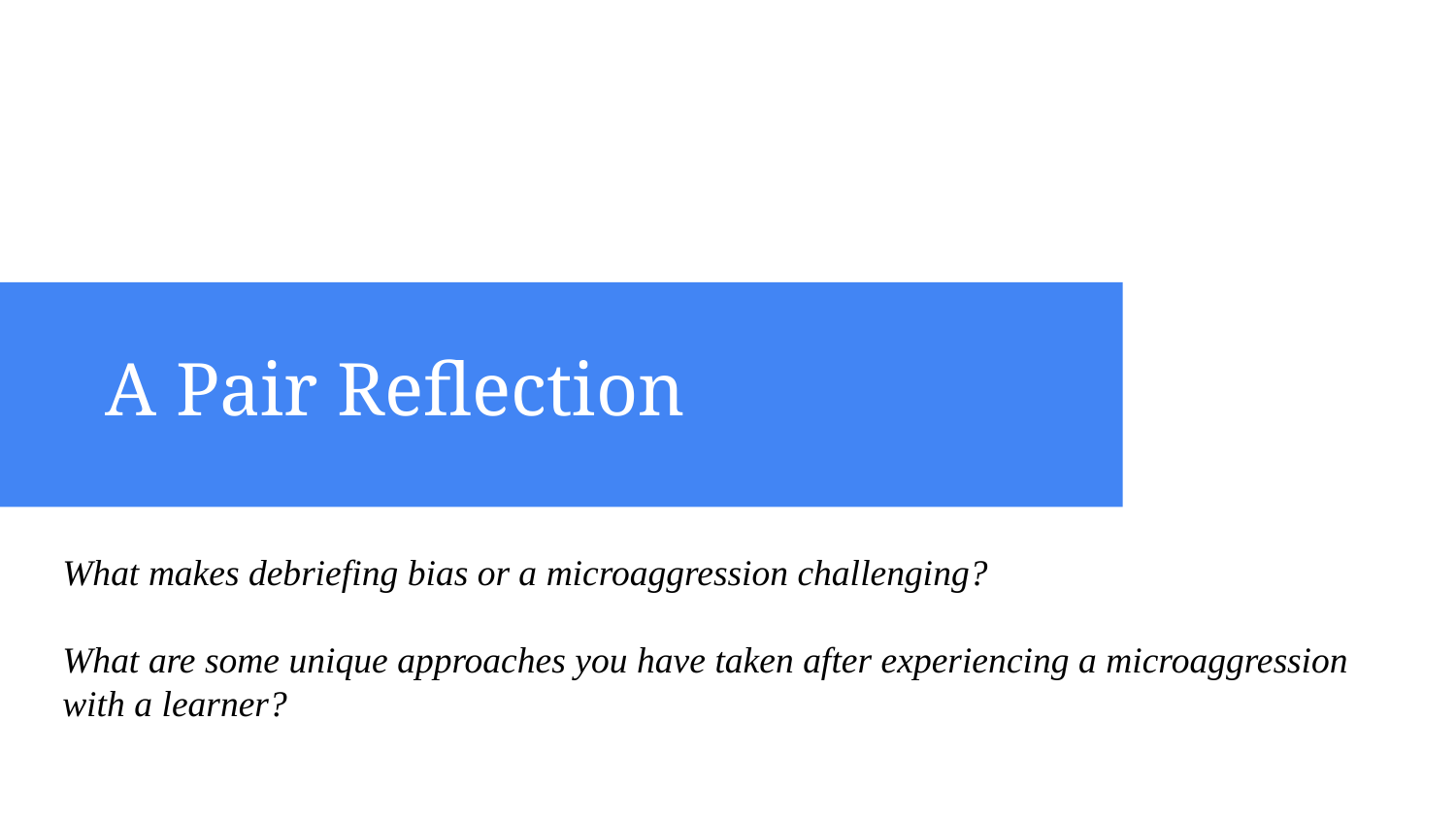

# A Pair Reflection
What makes debriefing bias or a microaggression challenging?
What are some unique approaches you have taken after experiencing a microaggression with a learner?

## Slide 11
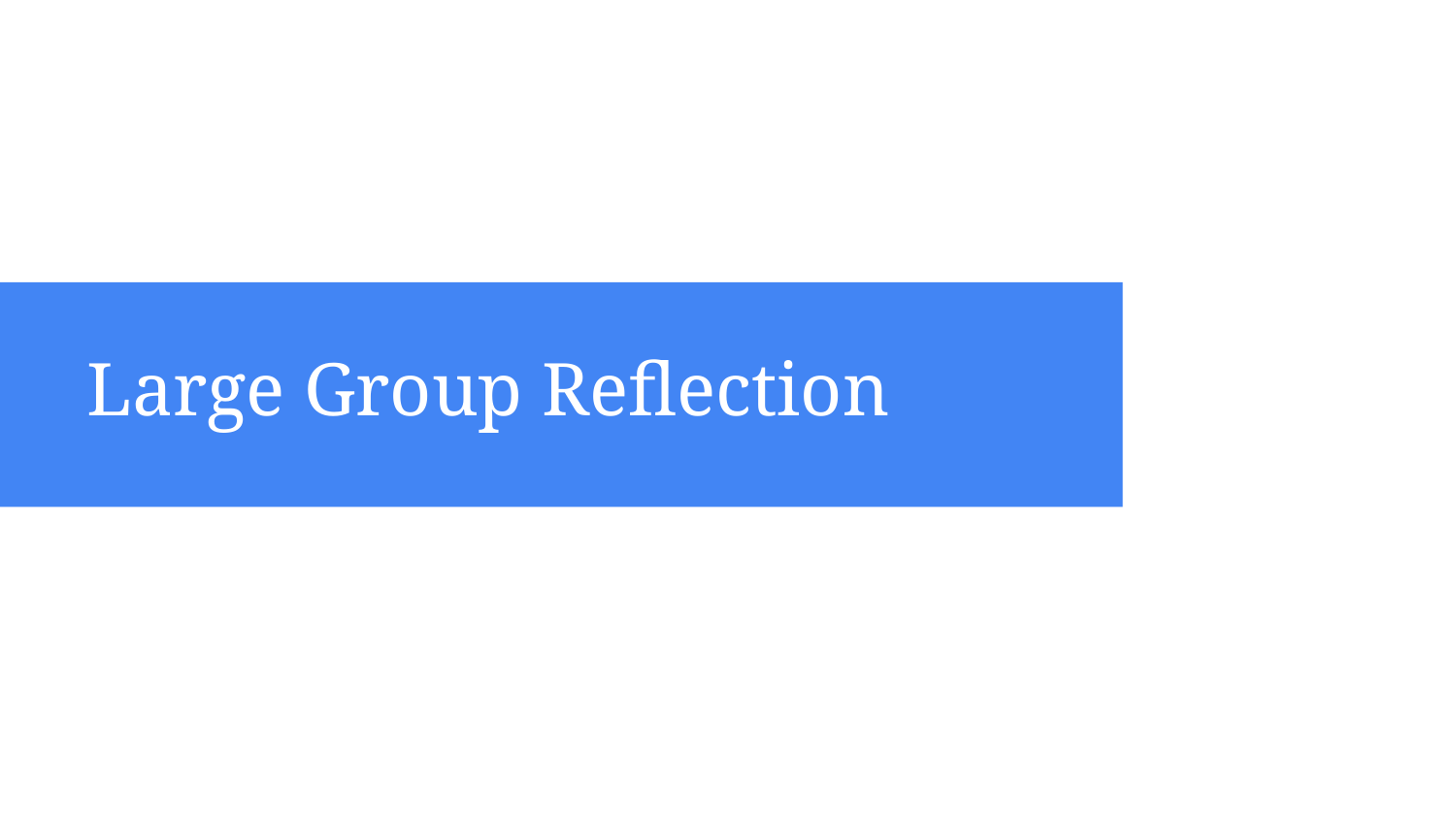

# Large Group Reflection

## Slide 12
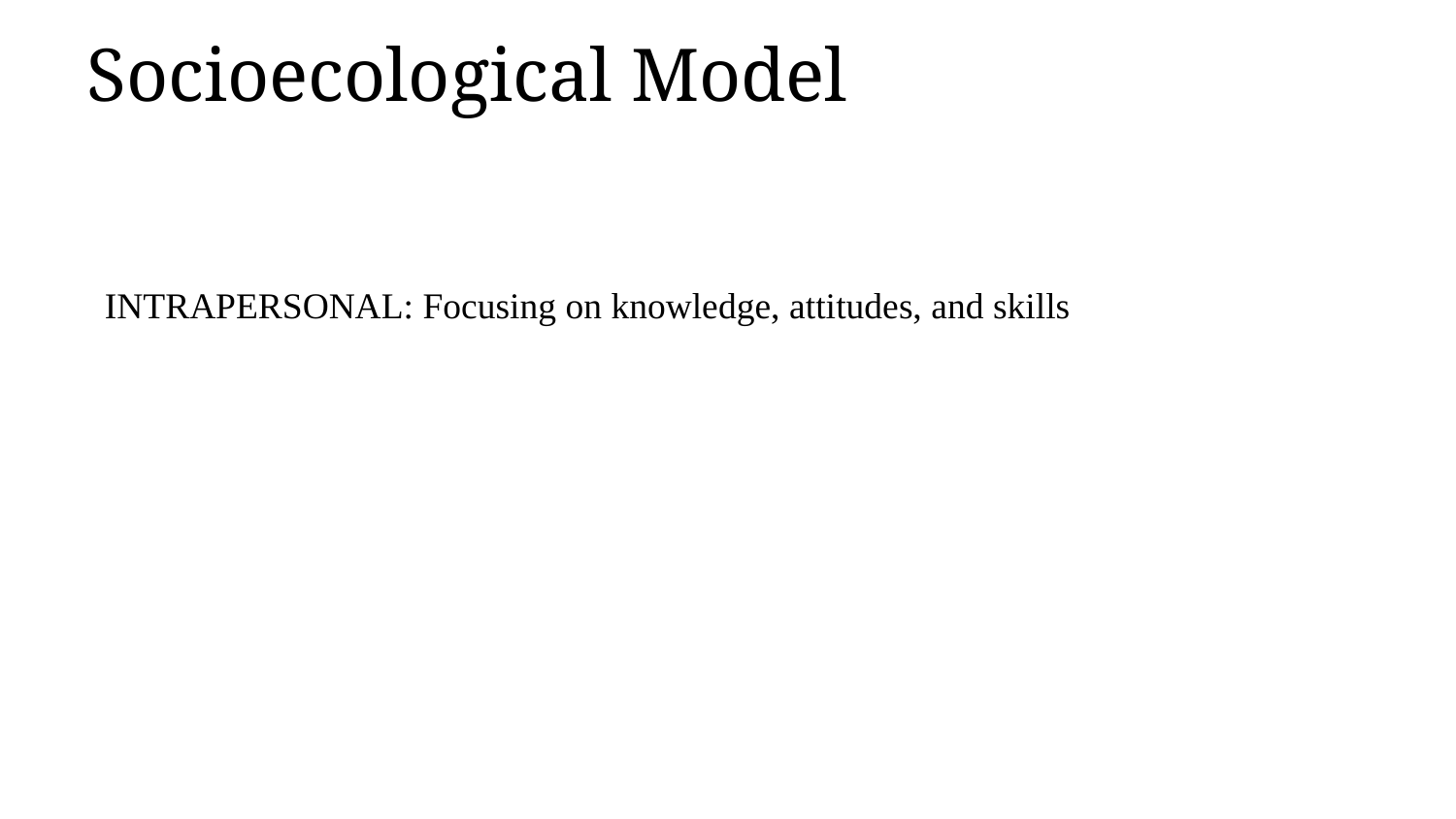

# Socioecological Model
INTRAPERSONAL: Focusing on knowledge, attitudes, and skills

## Slide 13
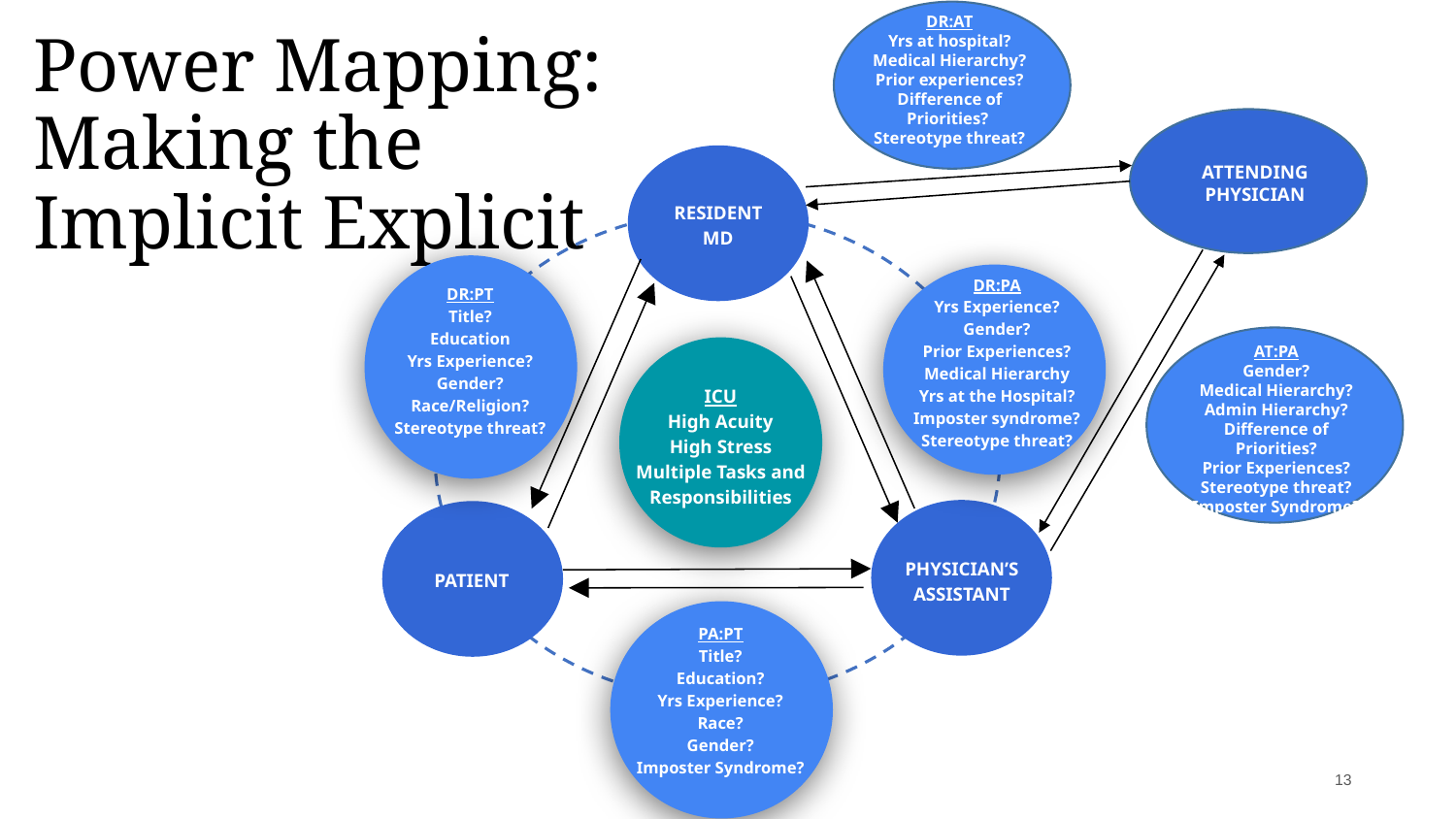

DR:AT
Yrs at hospital?
Medical Hierarchy?
Prior experiences?
Difference of Priorities?
Stereotype threat?
# Power Mapping: Making the Implicit Explicit
RESIDENT MD
ATTENDING PHYSICIAN
DR:PT
Title?
Education
Yrs Experience?
Gender?
Race/Religion?
Stereotype threat?
DR:PA
Yrs Experience?
Gender?
Prior Experiences?
Medical Hierarchy
Yrs at the Hospital?
Imposter syndrome?
Stereotype threat?
AT:PA
Gender?
Medical Hierarchy?
Admin Hierarchy?
Difference of Priorities?
Prior Experiences?
Stereotype threat?
Imposter Syndrome?
ICU
High Acuity
High Stress
Multiple Tasks and Responsibilities
PHYSICIAN’S ASSISTANT
PATIENT
PA:PT
Title?
Education?
Yrs Experience?
Race?
Gender?
Imposter Syndrome?
‹#›

## Slide 14
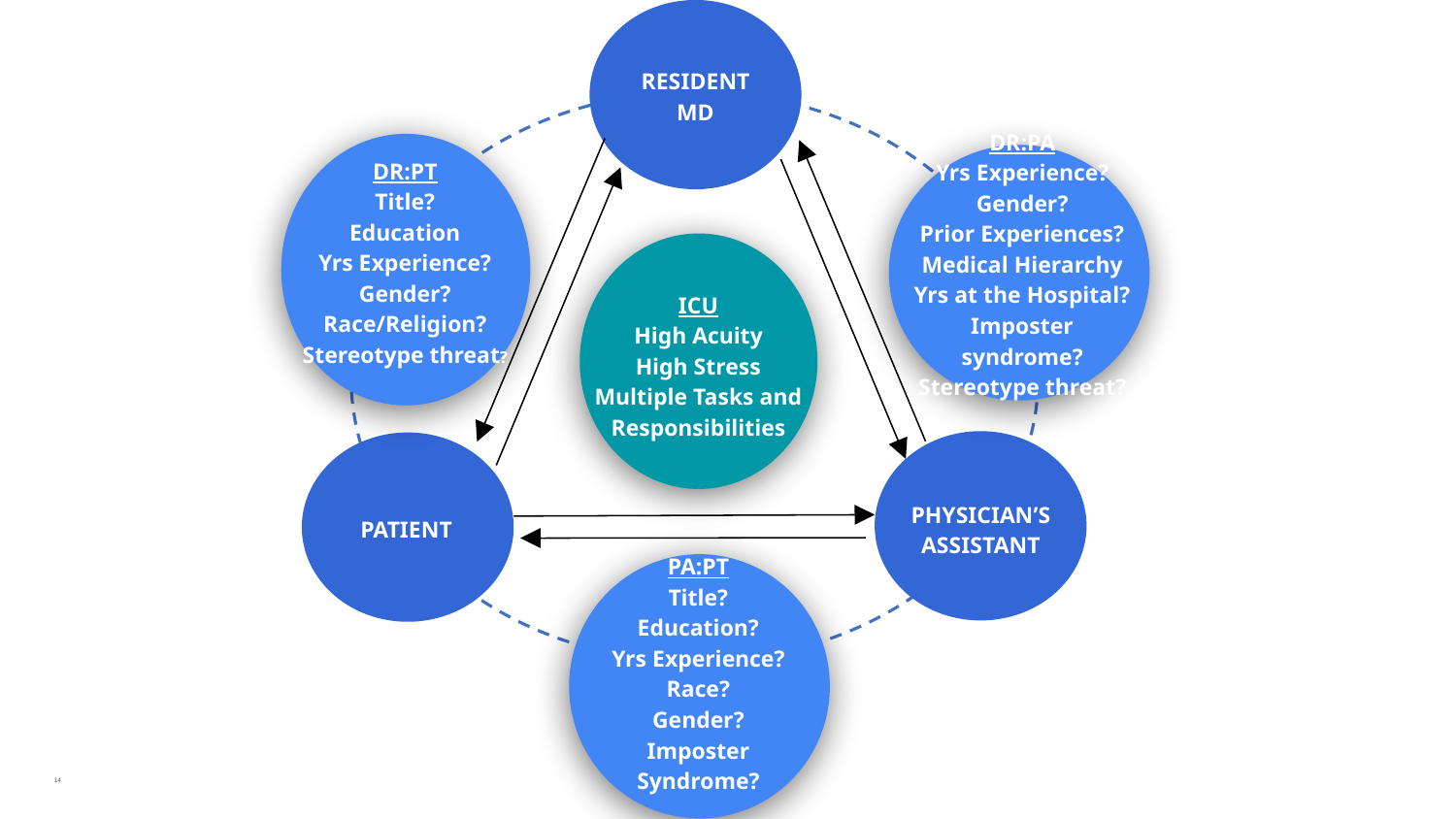

RESIDENT MD
DR:PT
Title?
Education
Yrs Experience?
Gender?
Race/Religion?
Stereotype threat?
DR:PA
Yrs Experience?
Gender?
Prior Experiences?
Medical Hierarchy
Yrs at the Hospital?
Imposter syndrome?
Stereotype threat?
ICU
High Acuity
High Stress
Multiple Tasks and Responsibilities
PHYSICIAN’S ASSISTANT
PATIENT
PA:PT
Title?
Education?
Yrs Experience?
Race?
Gender?
Imposter Syndrome?
‹#›

## Slide 15
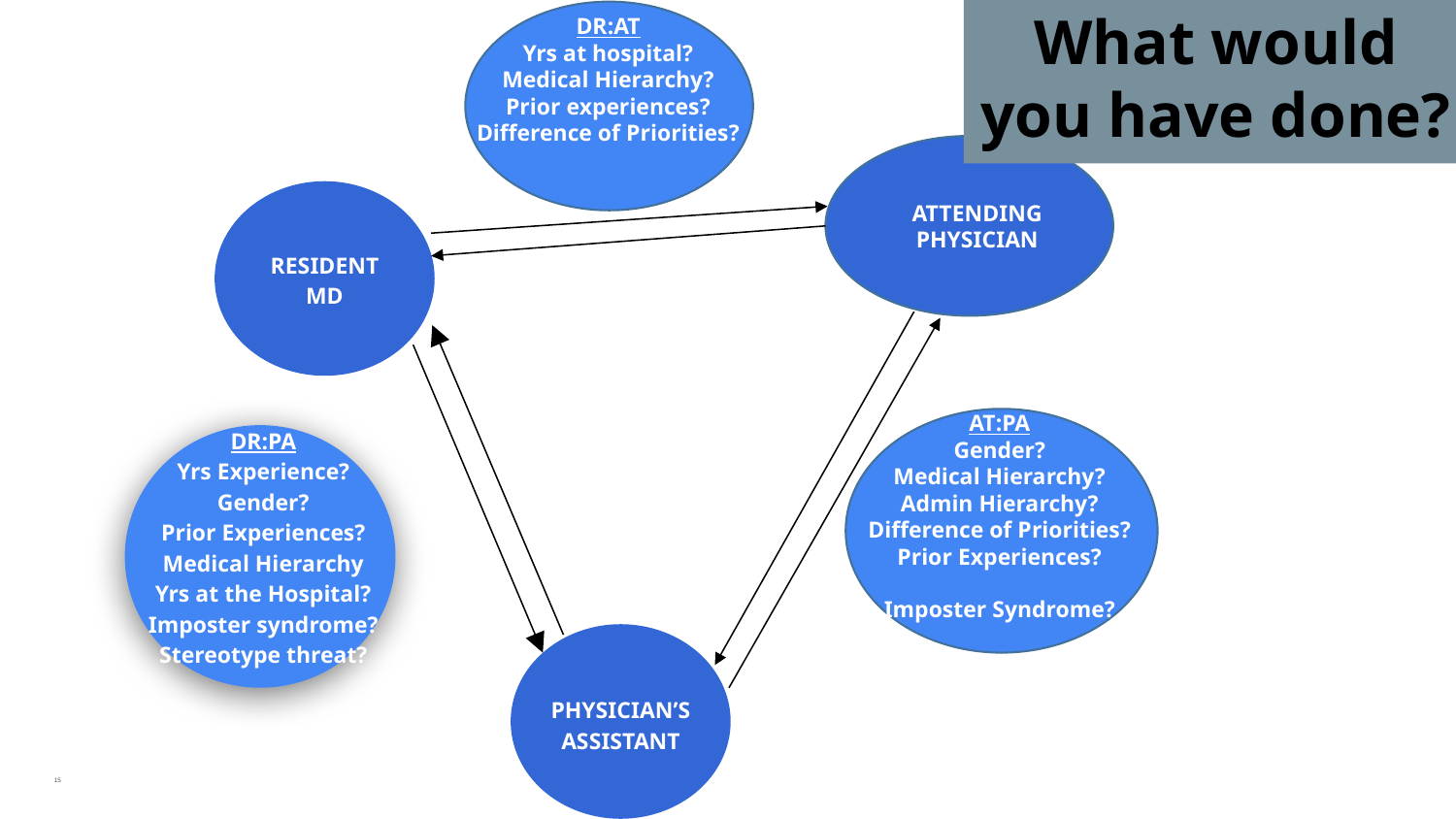

What would you have done?
DR:AT
Yrs at hospital?
Medical Hierarchy?
Prior experiences?
Difference of Priorities?
RESIDENT MD
ATTENDING PHYSICIAN
AT:PA
Gender?
Medical Hierarchy?
Admin Hierarchy?
Difference of Priorities?
Prior Experiences?
Imposter Syndrome?
DR:PA
Yrs Experience?
Gender?
Prior Experiences?
Medical Hierarchy
Yrs at the Hospital?
Imposter syndrome?
Stereotype threat?
PHYSICIAN’S ASSISTANT
‹#›

## Slide 16
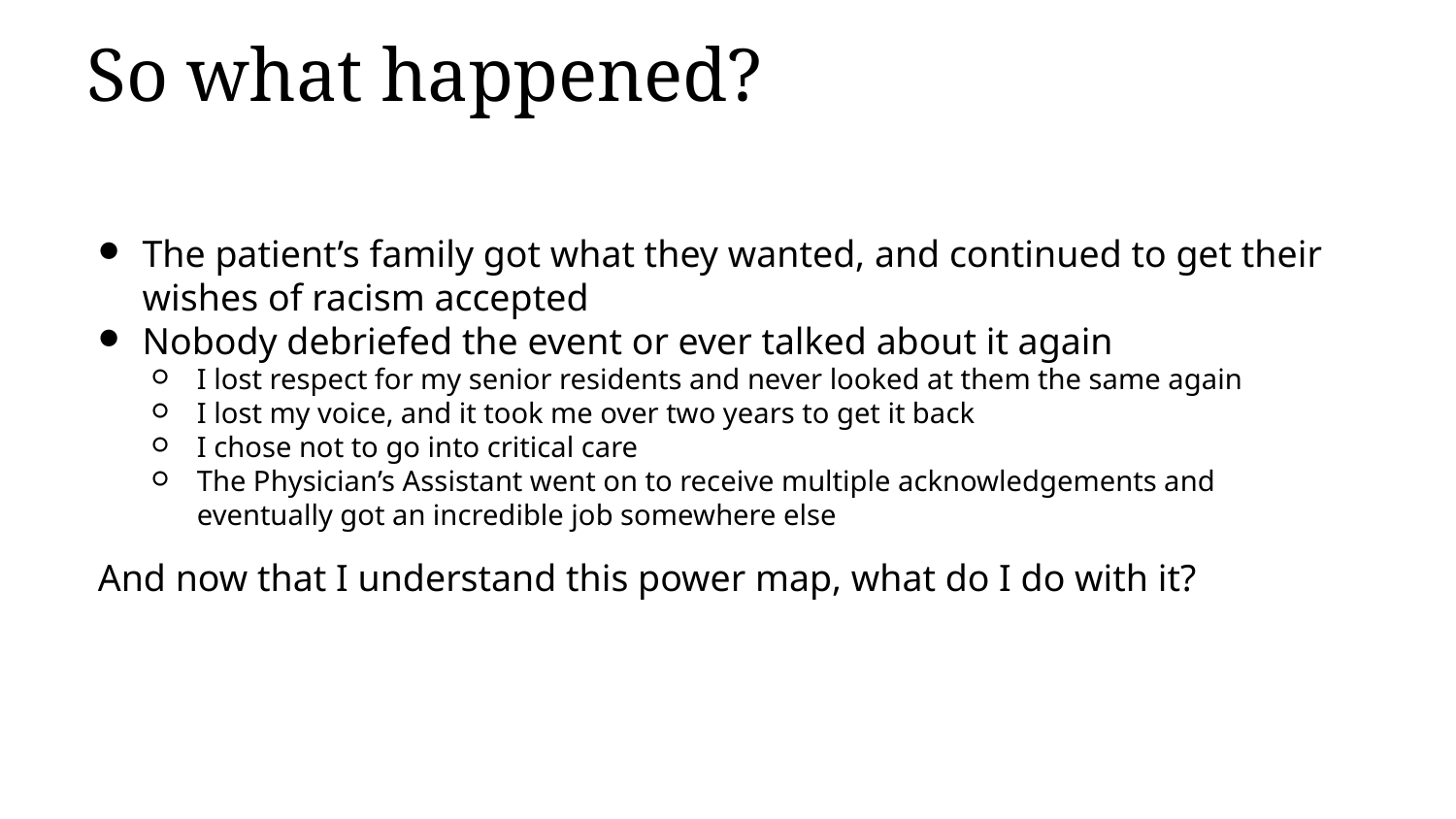

# So what happened?
The patient’s family got what they wanted, and continued to get their wishes of racism accepted
Nobody debriefed the event or ever talked about it again
I lost respect for my senior residents and never looked at them the same again
I lost my voice, and it took me over two years to get it back
I chose not to go into critical care
The Physician’s Assistant went on to receive multiple acknowledgements and eventually got an incredible job somewhere else
 And now that I understand this power map, what do I do with it?

## Slide 17
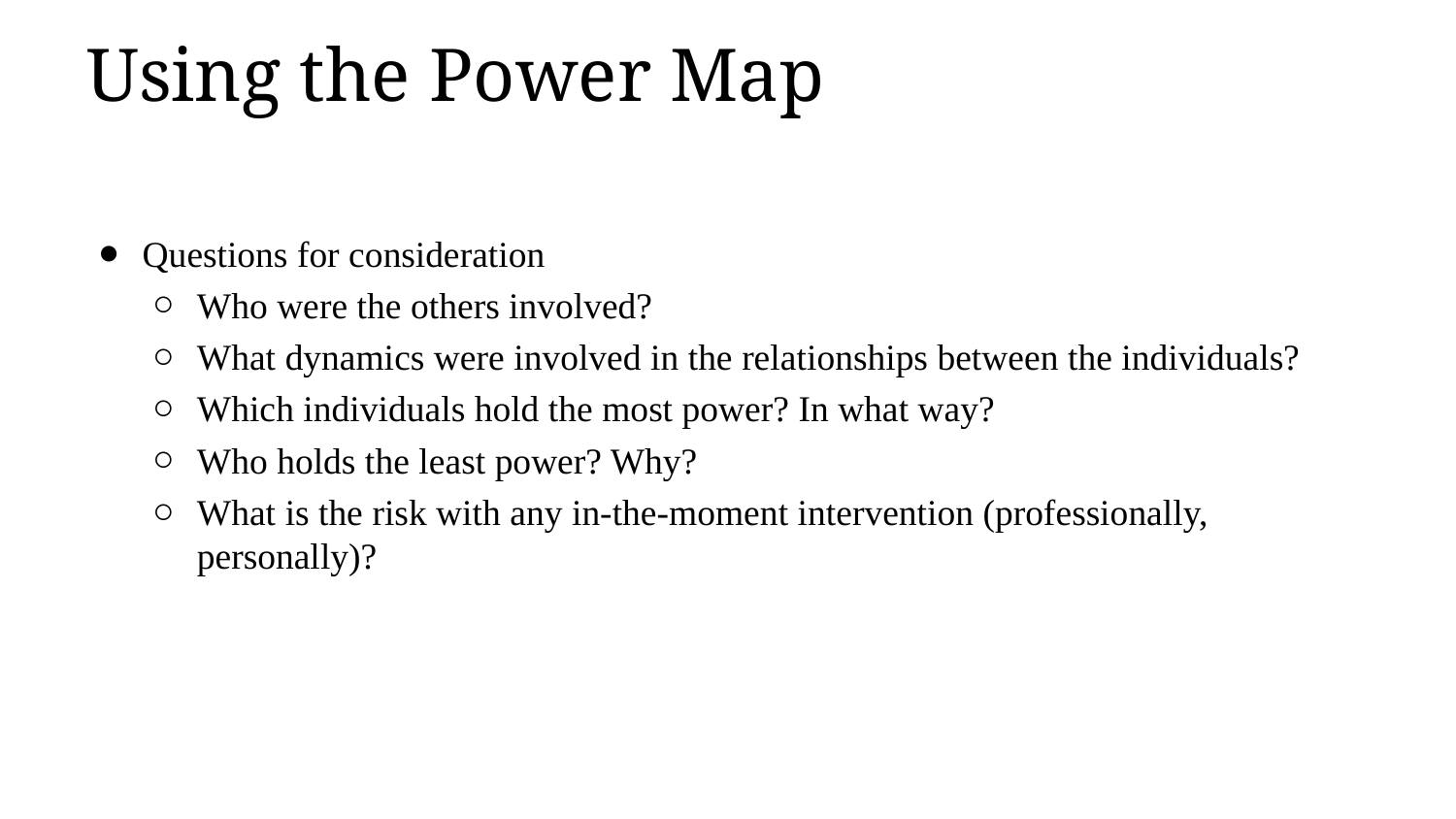

# Using the Power Map
Questions for consideration
Who were the others involved?
What dynamics were involved in the relationships between the individuals?
Which individuals hold the most power? In what way?
Who holds the least power? Why?
What is the risk with any in-the-moment intervention (professionally, personally)?

## Slide 18
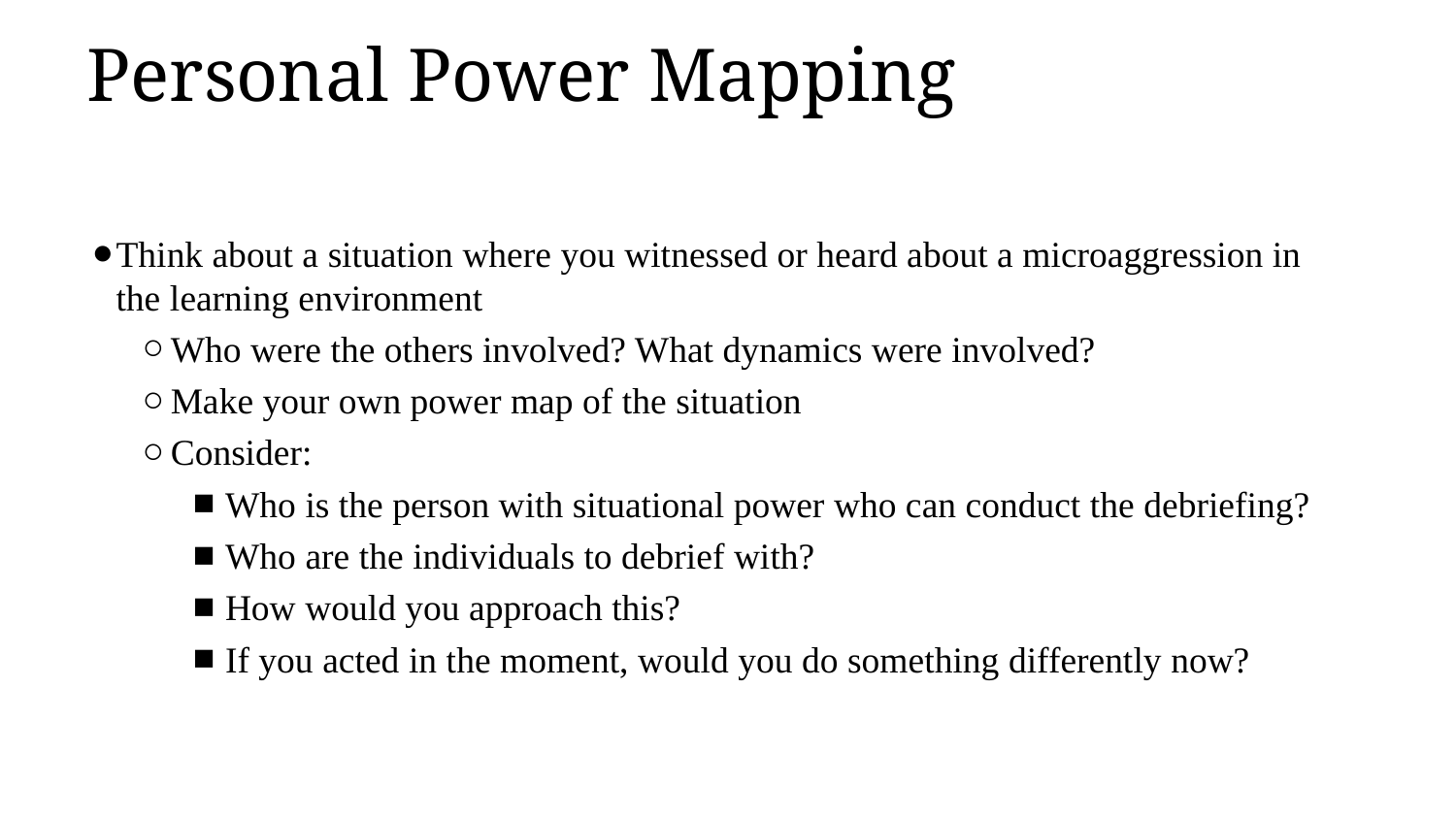

# Personal Power Mapping
Think about a situation where you witnessed or heard about a microaggression in the learning environment
Who were the others involved? What dynamics were involved?
Make your own power map of the situation
Consider:
Who is the person with situational power who can conduct the debriefing?
Who are the individuals to debrief with?
How would you approach this?
If you acted in the moment, would you do something differently now?

## Slide 19
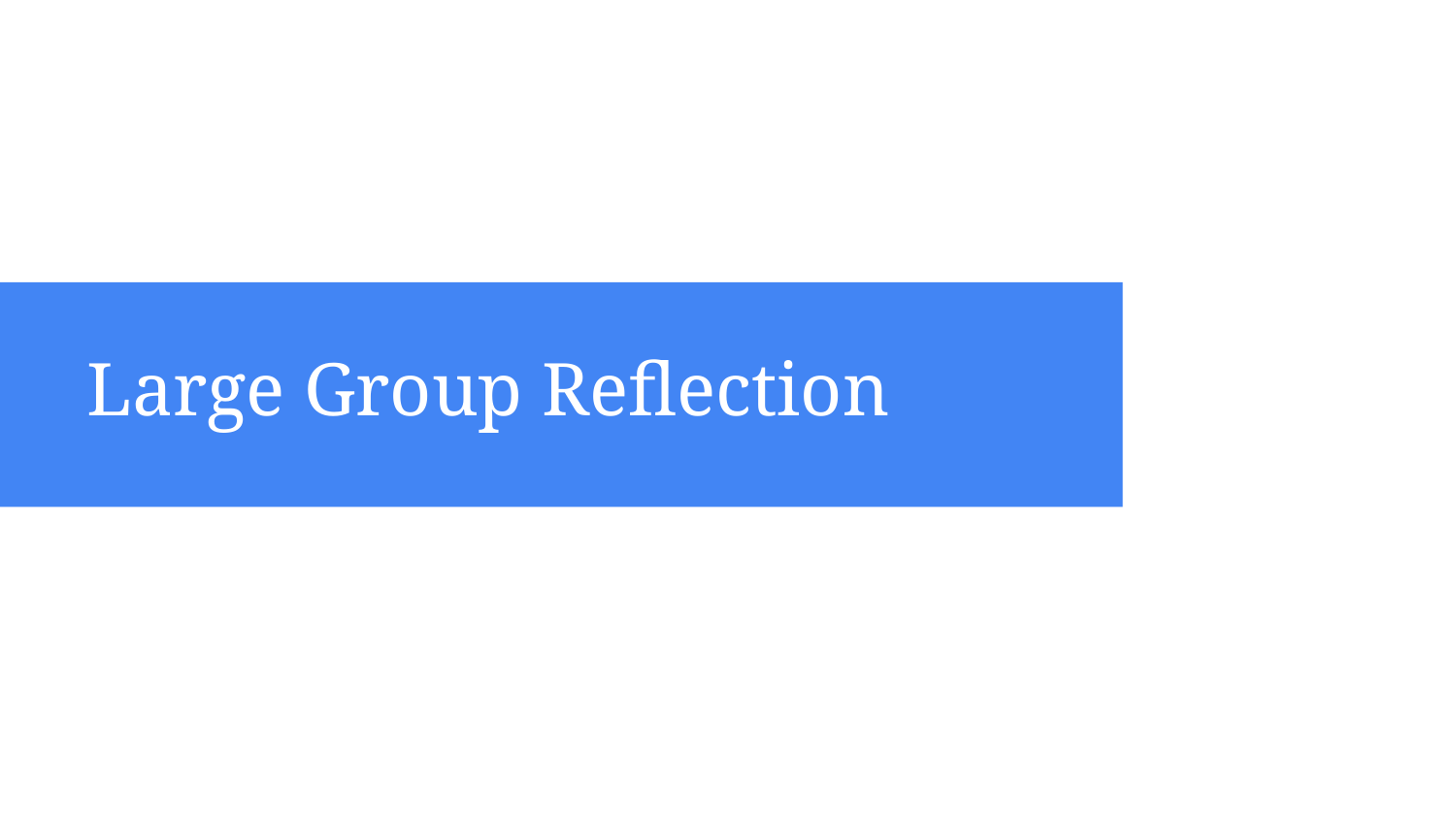

# Large Group Reflection

## Slide 20
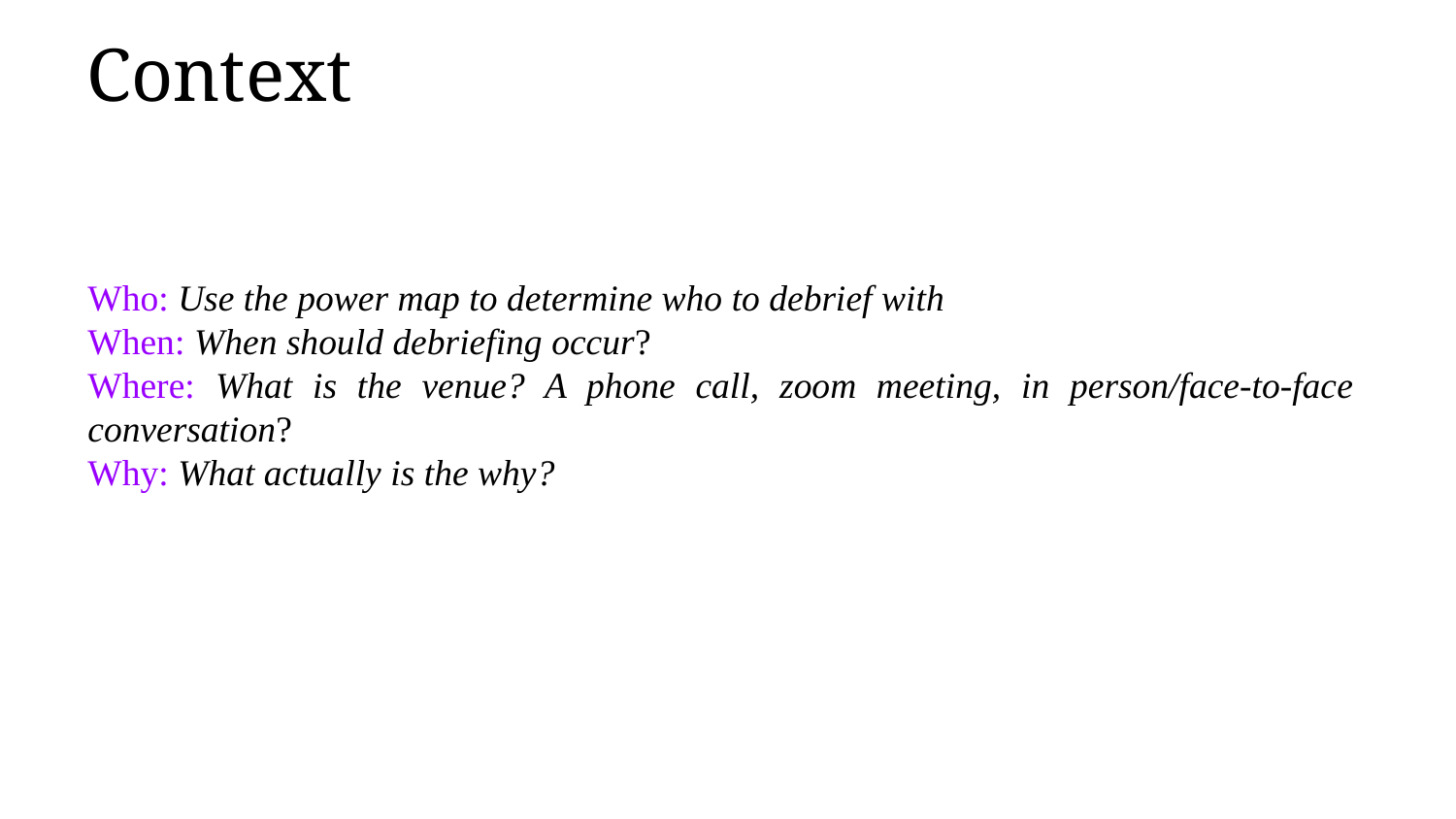

# Context
Who: Use the power map to determine who to debrief with
When: When should debriefing occur?
Where: What is the venue? A phone call, zoom meeting, in person/face-to-face conversation?
Why: What actually is the why?

## Slide 21
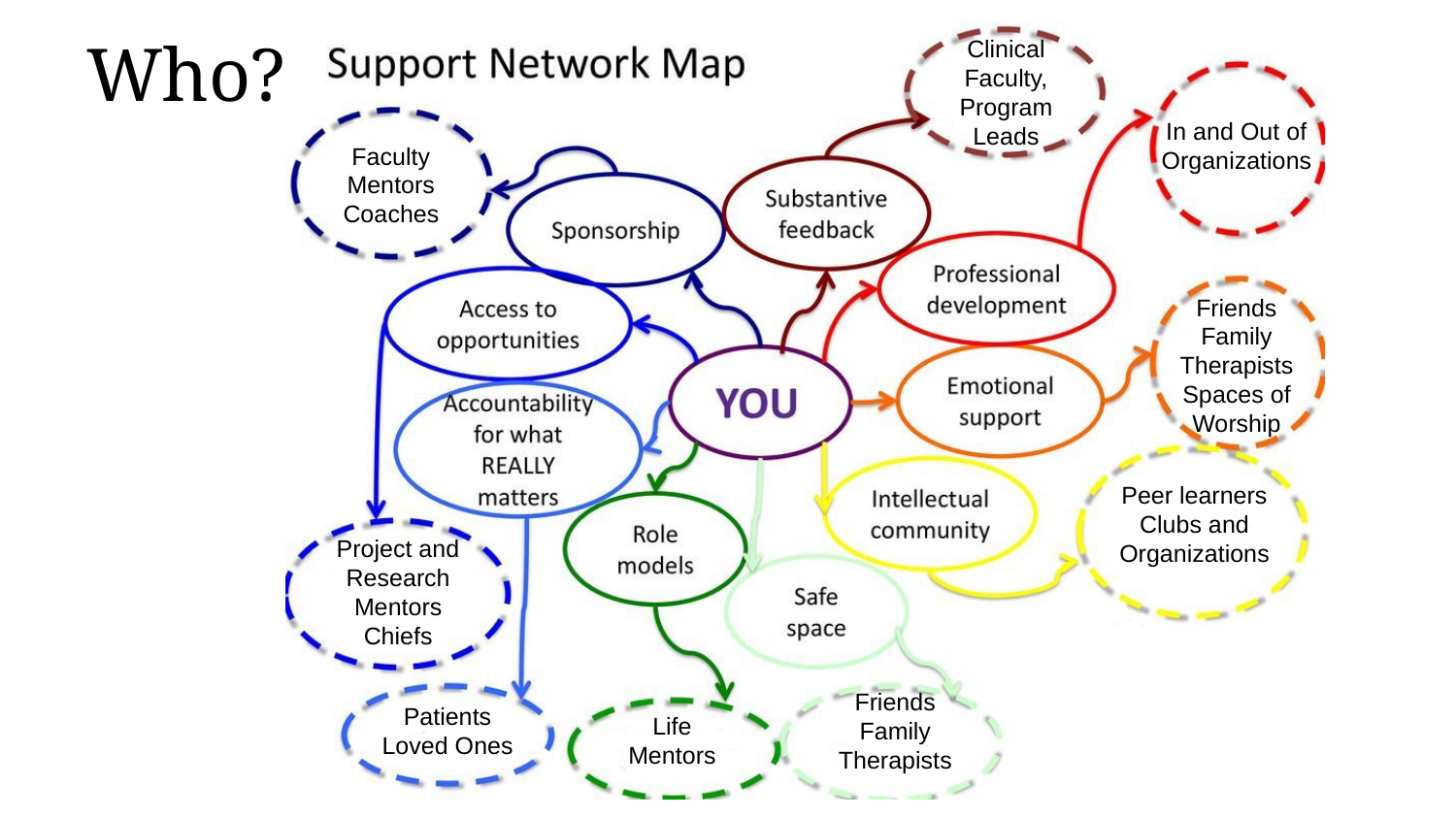

Clinical Faculty, Program Leads
In and Out of Organizations
Faculty
Mentors
Coaches
Friends
Family
Therapists
Spaces of Worship
Peer learners
Clubs and Organizations
Project and Research Mentors
Chiefs
Friends
Family
Therapists
Patients
Loved Ones
Life Mentors
# Who?

## Slide 22
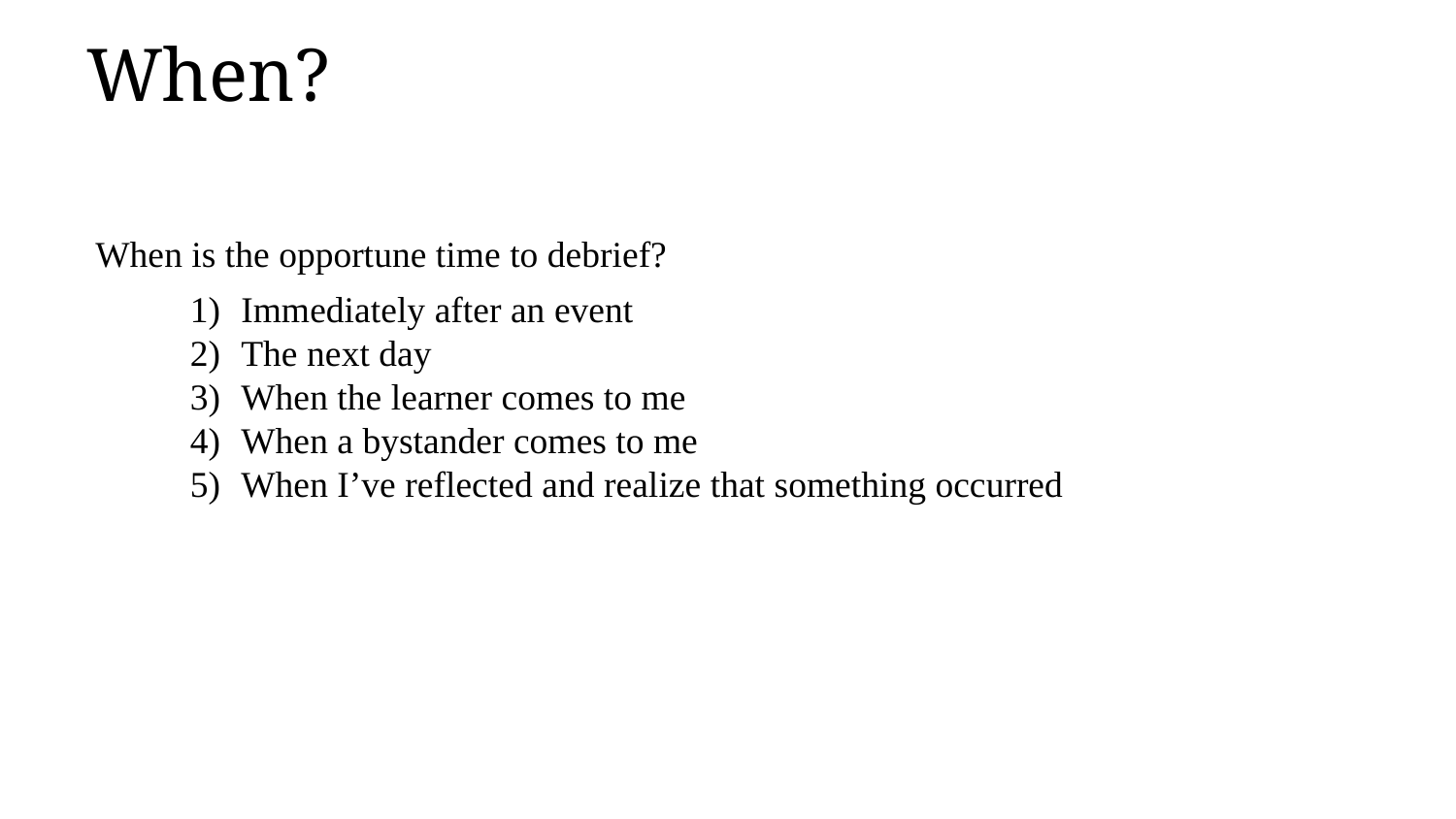

# When?
When is the opportune time to debrief?
Immediately after an event
The next day
When the learner comes to me
When a bystander comes to me
When I’ve reflected and realize that something occurred

## Slide 23
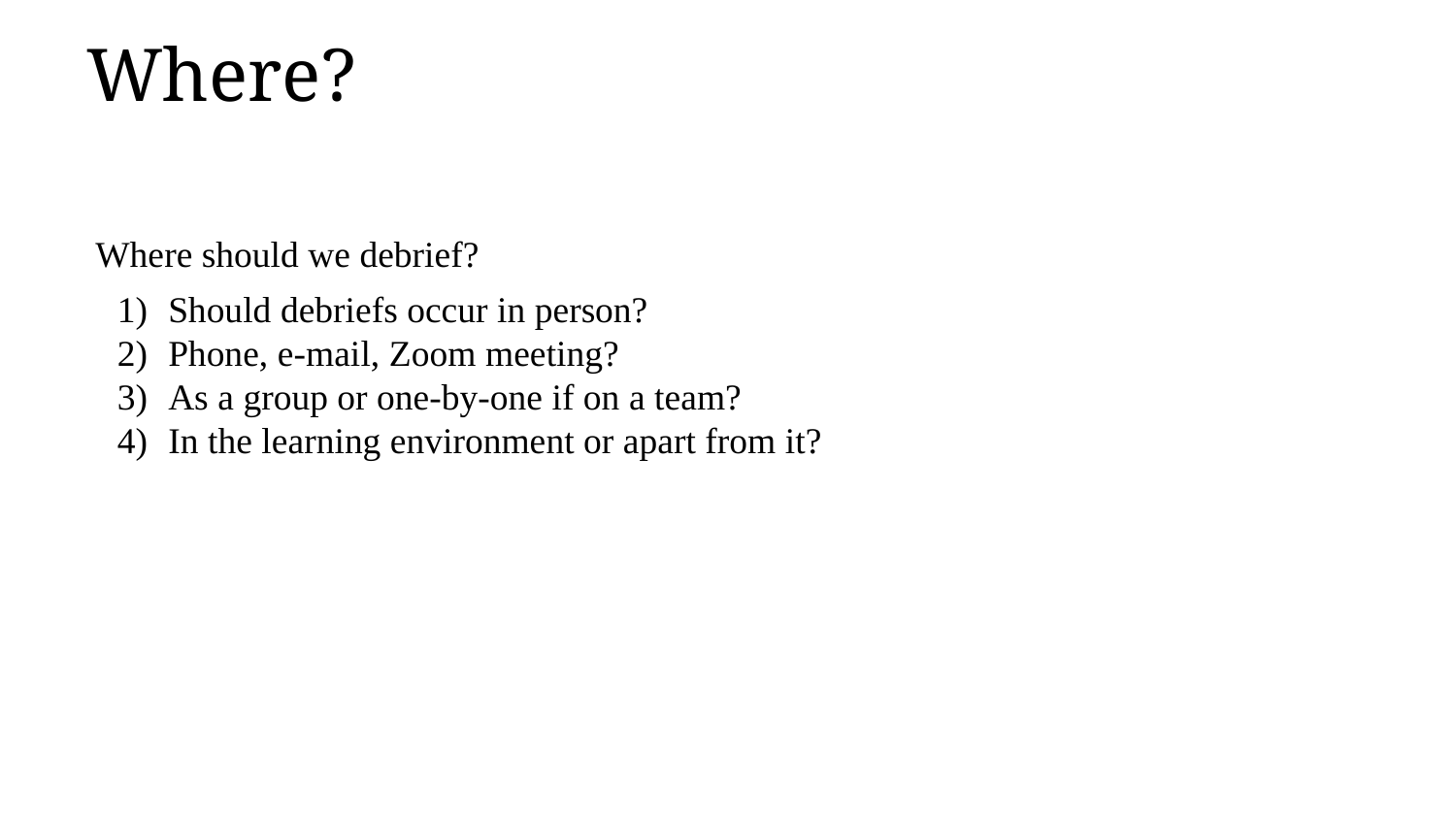

# Where?
Where should we debrief?
Should debriefs occur in person?
Phone, e-mail, Zoom meeting?
As a group or one-by-one if on a team?
In the learning environment or apart from it?

## Slide 24
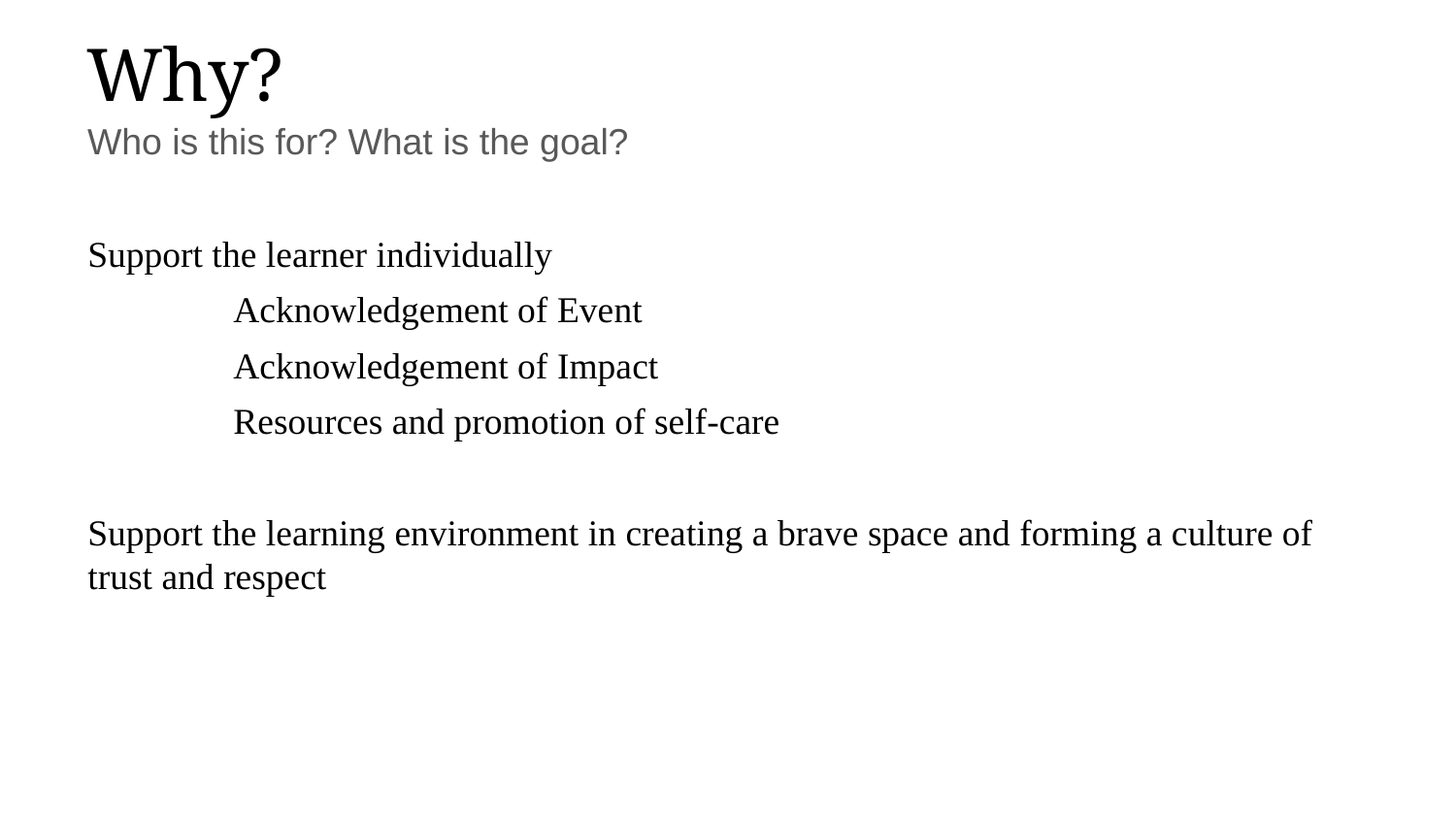

# Why?
Who is this for? What is the goal?
Support the learner individually
	Acknowledgement of Event
	Acknowledgement of Impact
	Resources and promotion of self-care
Support the learning environment in creating a brave space and forming a culture of trust and respect

## Slide 25
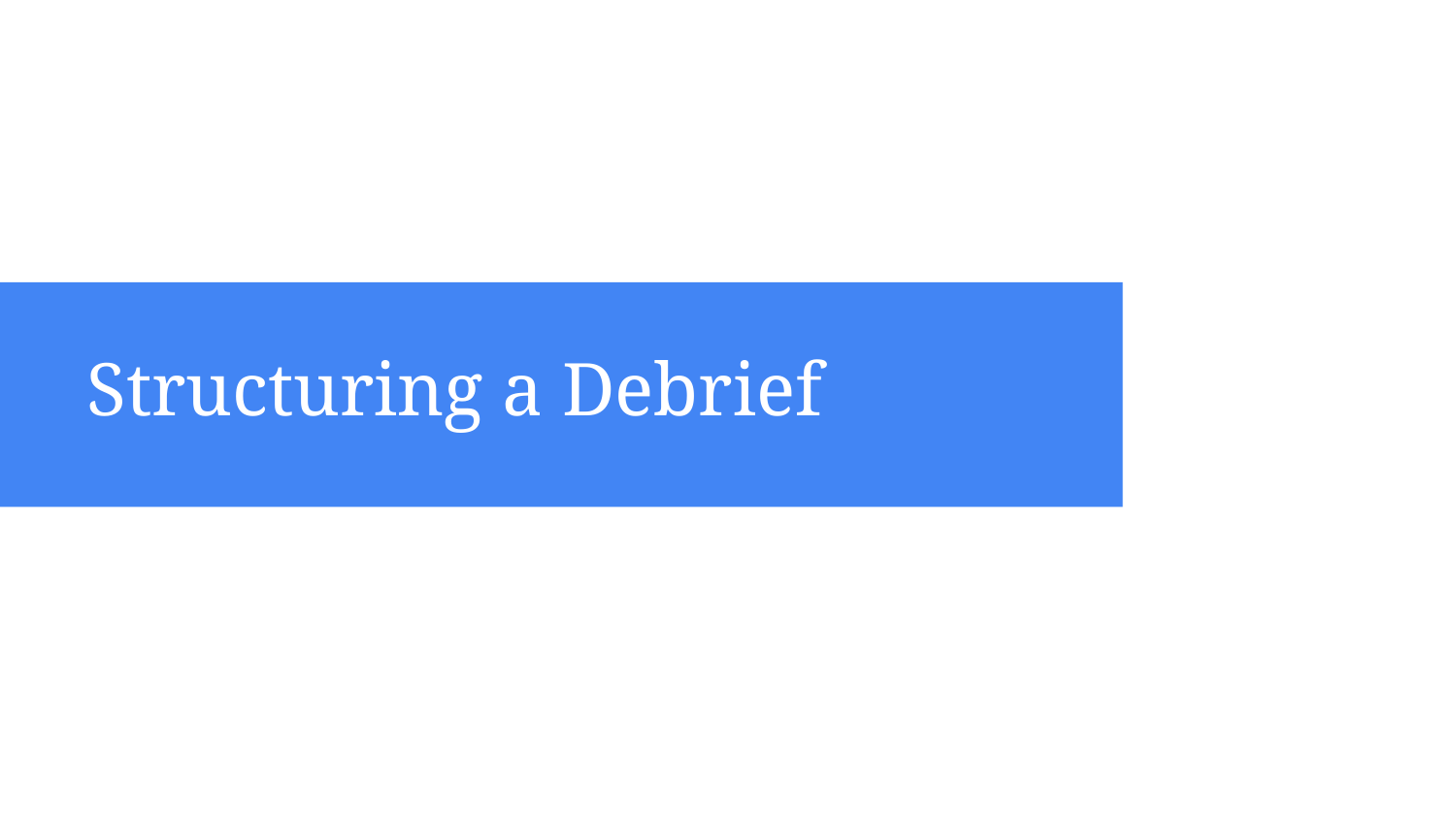

# Structuring a Debrief

## Slide 26
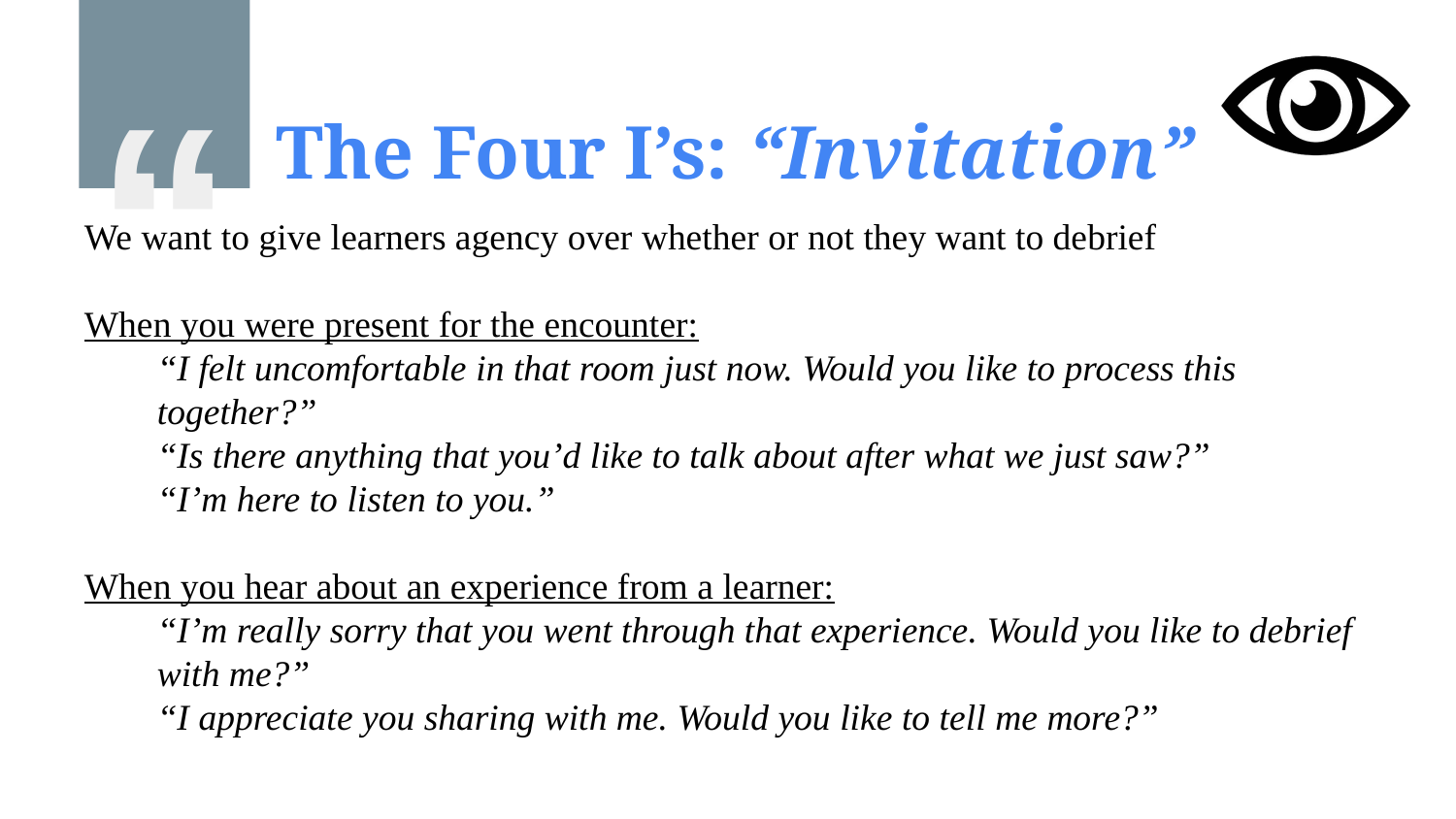

The Four I’s: “Invitation”
We want to give learners agency over whether or not they want to debrief
When you were present for the encounter:
“I felt uncomfortable in that room just now. Would you like to process this
together?”
“Is there anything that you’d like to talk about after what we just saw?”
“I’m here to listen to you.”
When you hear about an experience from a learner:
“I’m really sorry that you went through that experience. Would you like to debrief
with me?”
“I appreciate you sharing with me. Would you like to tell me more?”

## Slide 27
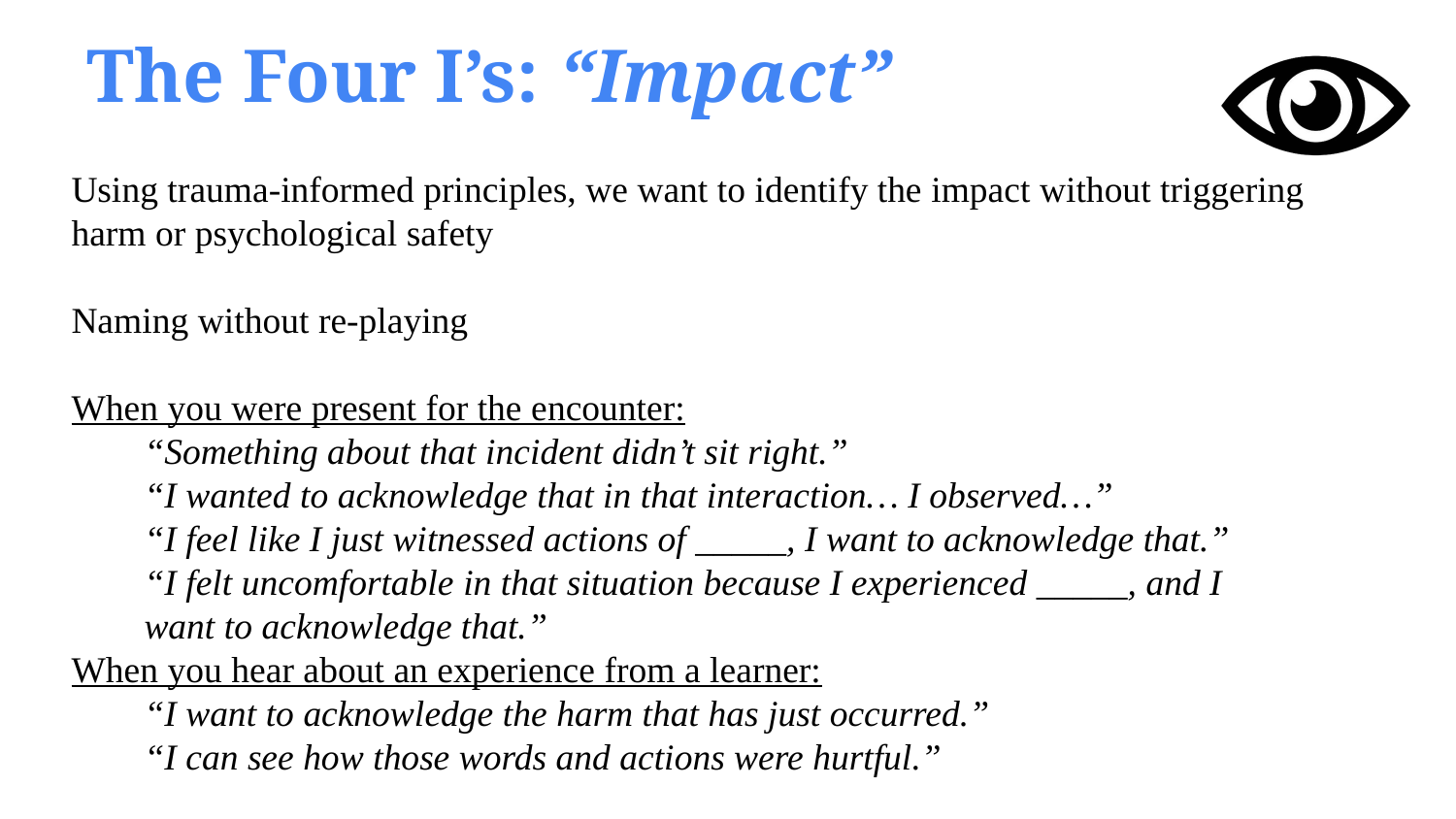

# The Four I’s: “Impact”
Using trauma-informed principles, we want to identify the impact without triggering harm or psychological safety
Naming without re-playing
When you were present for the encounter:
“Something about that incident didn’t sit right.”
“I wanted to acknowledge that in that interaction… I observed…”
“I feel like I just witnessed actions of _____, I want to acknowledge that.”
“I felt uncomfortable in that situation because I experienced _____, and I
want to acknowledge that.”
When you hear about an experience from a learner:
“I want to acknowledge the harm that has just occurred.”
“I can see how those words and actions were hurtful.”

## Slide 28
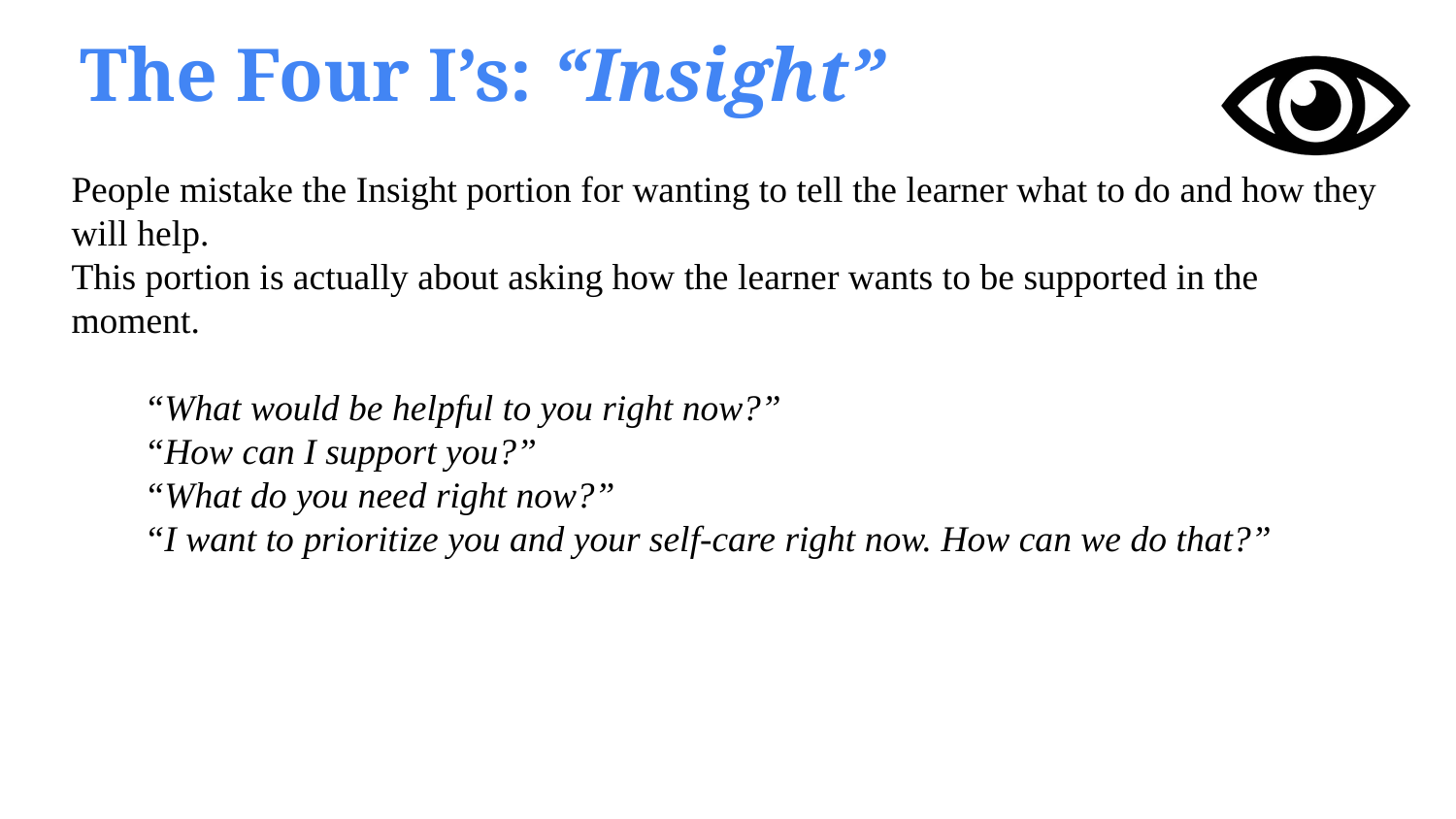

# The Four I’s: “Insight”
People mistake the Insight portion for wanting to tell the learner what to do and how they will help.
This portion is actually about asking how the learner wants to be supported in the moment.
“What would be helpful to you right now?”
“How can I support you?”
“What do you need right now?”
“I want to prioritize you and your self-care right now. How can we do that?”

## Slide 29
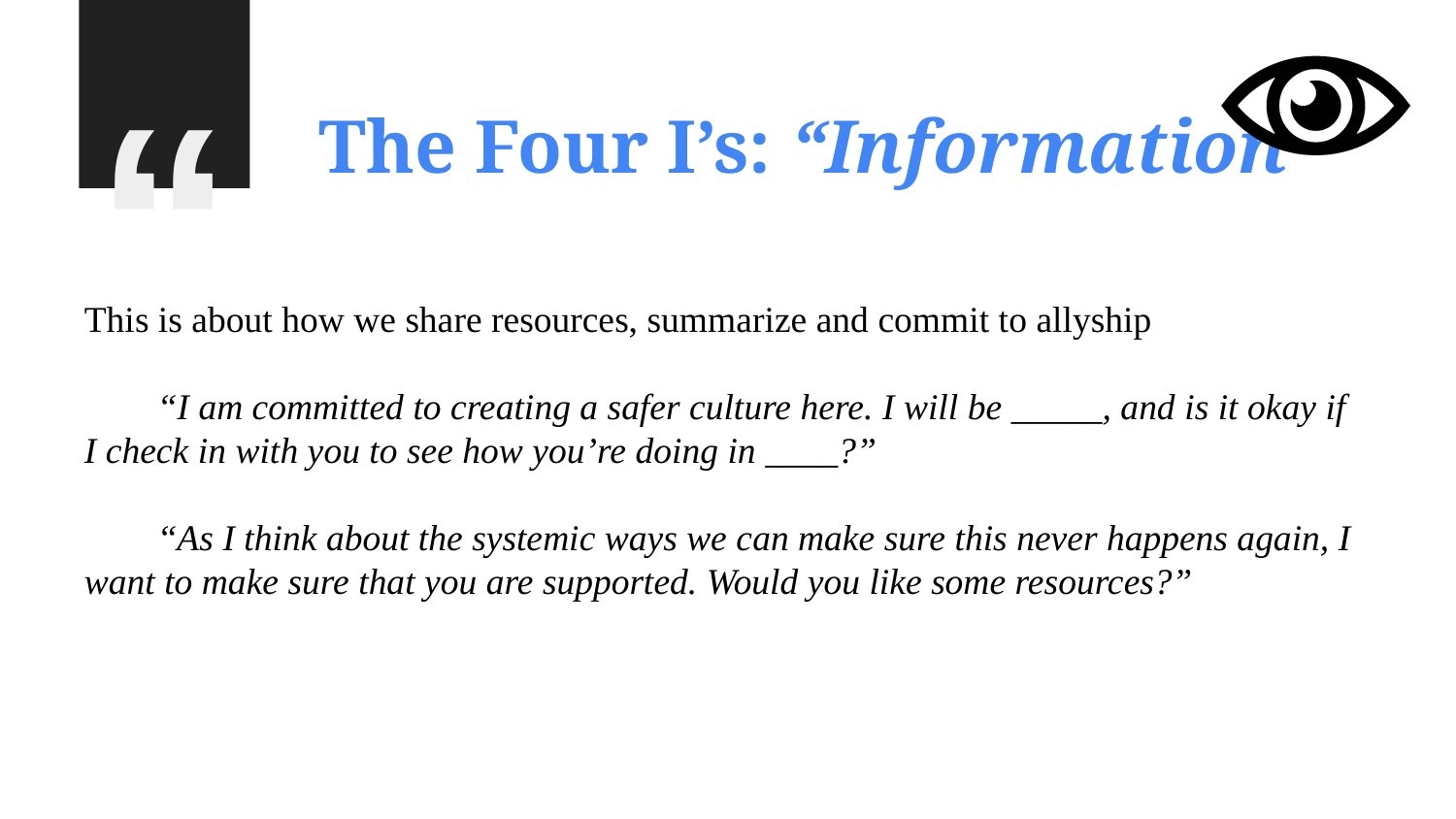

The Four I’s: “Information”
This is about how we share resources, summarize and commit to allyship
“I am committed to creating a safer culture here. I will be _____, and is it okay if I check in with you to see how you’re doing in ____?”
“As I think about the systemic ways we can make sure this never happens again, I want to make sure that you are supported. Would you like some resources?”

## Slide 30
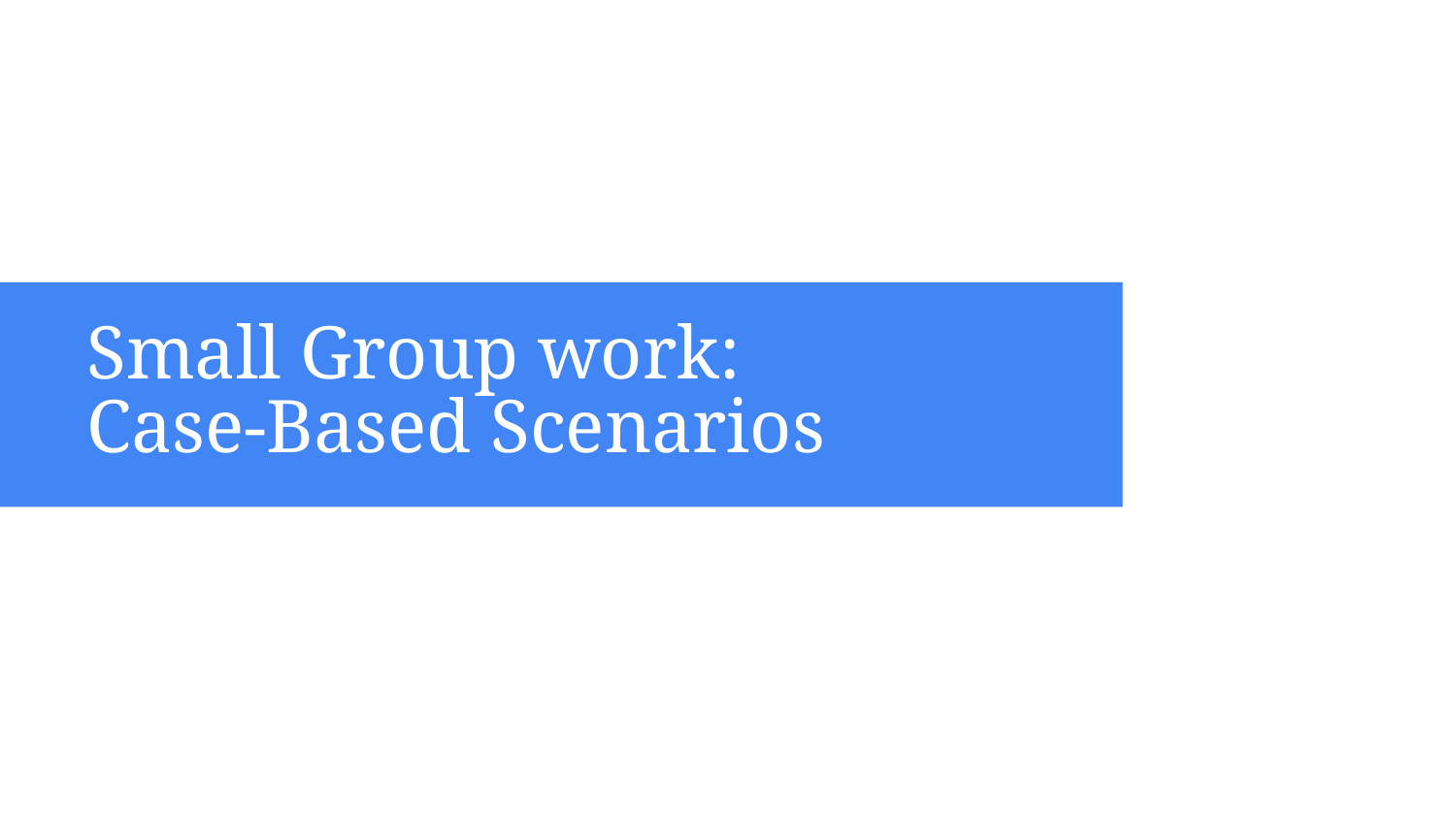

# Small Group work:
Case-Based Scenarios

## Slide 31
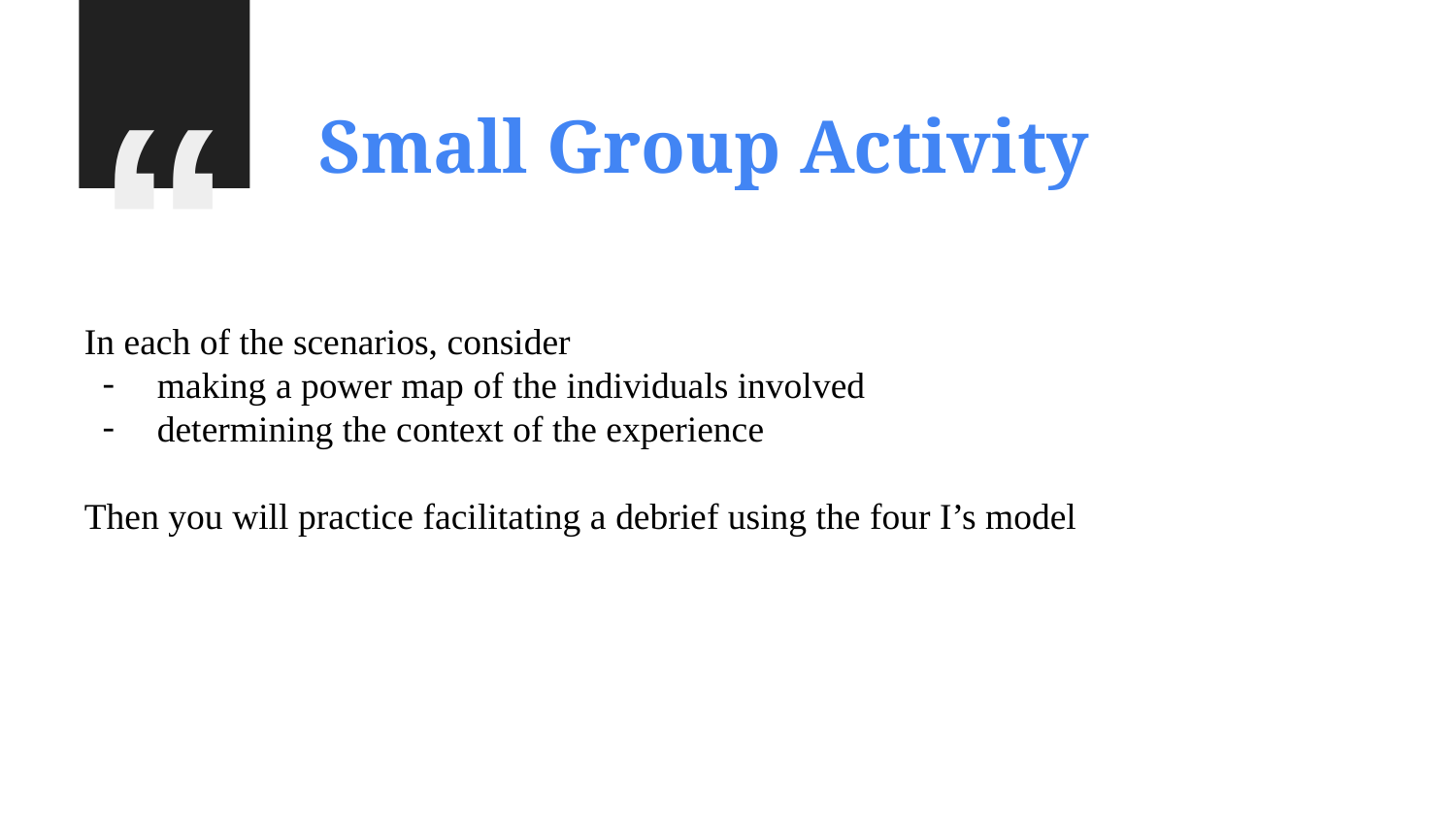

Small Group Activity
In each of the scenarios, consider
making a power map of the individuals involved
determining the context of the experience
Then you will practice facilitating a debrief using the four I’s model

## Slide 32
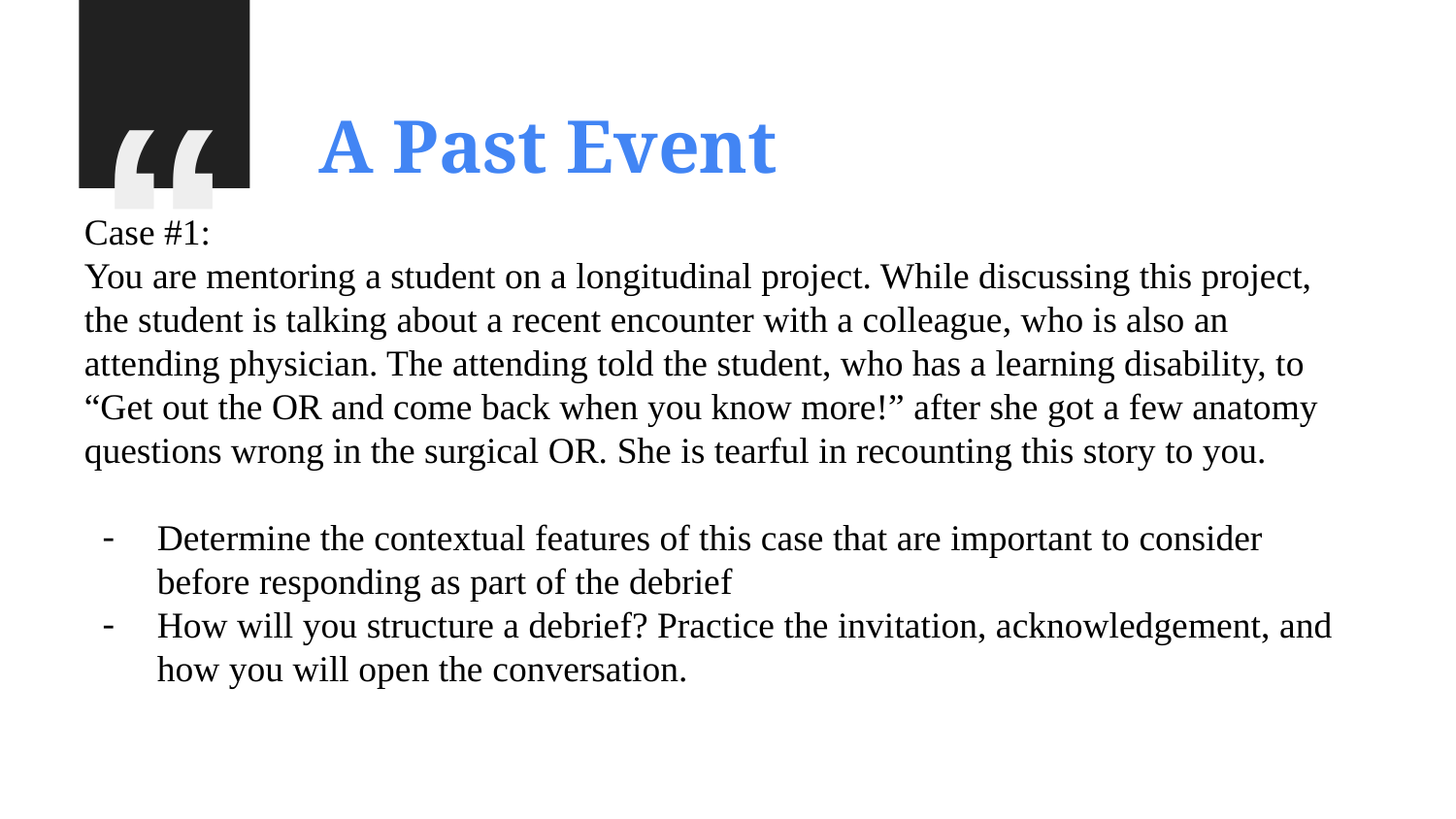

A Past Event
Case #1:
You are mentoring a student on a longitudinal project. While discussing this project, the student is talking about a recent encounter with a colleague, who is also an attending physician. The attending told the student, who has a learning disability, to “Get out the OR and come back when you know more!” after she got a few anatomy questions wrong in the surgical OR. She is tearful in recounting this story to you.
Determine the contextual features of this case that are important to consider before responding as part of the debrief
How will you structure a debrief? Practice the invitation, acknowledgement, and how you will open the conversation.

## Slide 33
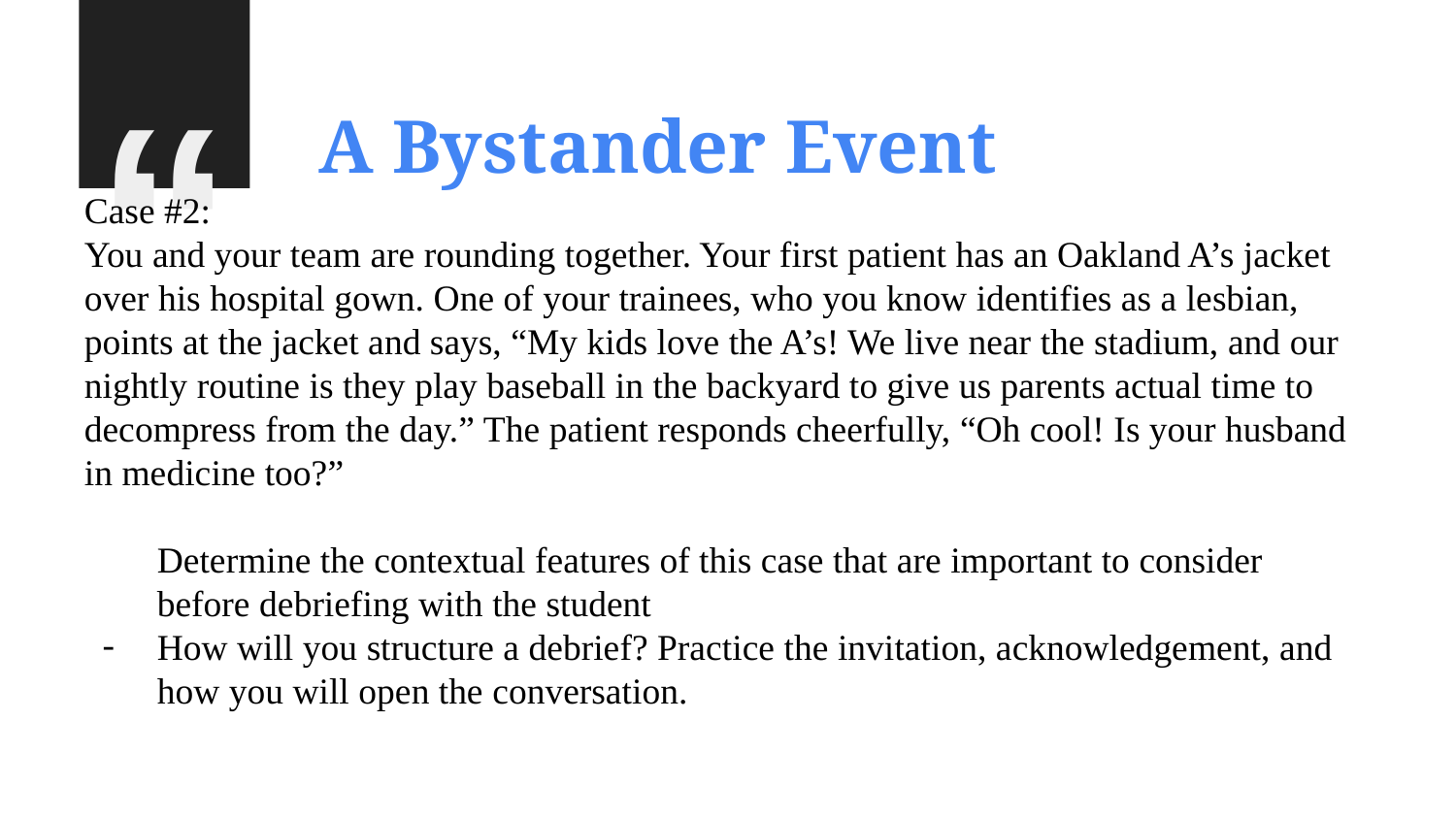

A Bystander Event
Case #2:
You and your team are rounding together. Your first patient has an Oakland A’s jacket over his hospital gown. One of your trainees, who you know identifies as a lesbian, points at the jacket and says, “My kids love the A’s! We live near the stadium, and our nightly routine is they play baseball in the backyard to give us parents actual time to decompress from the day.” The patient responds cheerfully, “Oh cool! Is your husband in medicine too?”
Determine the contextual features of this case that are important to consider before debriefing with the student
How will you structure a debrief? Practice the invitation, acknowledgement, and how you will open the conversation.

## Slide 34
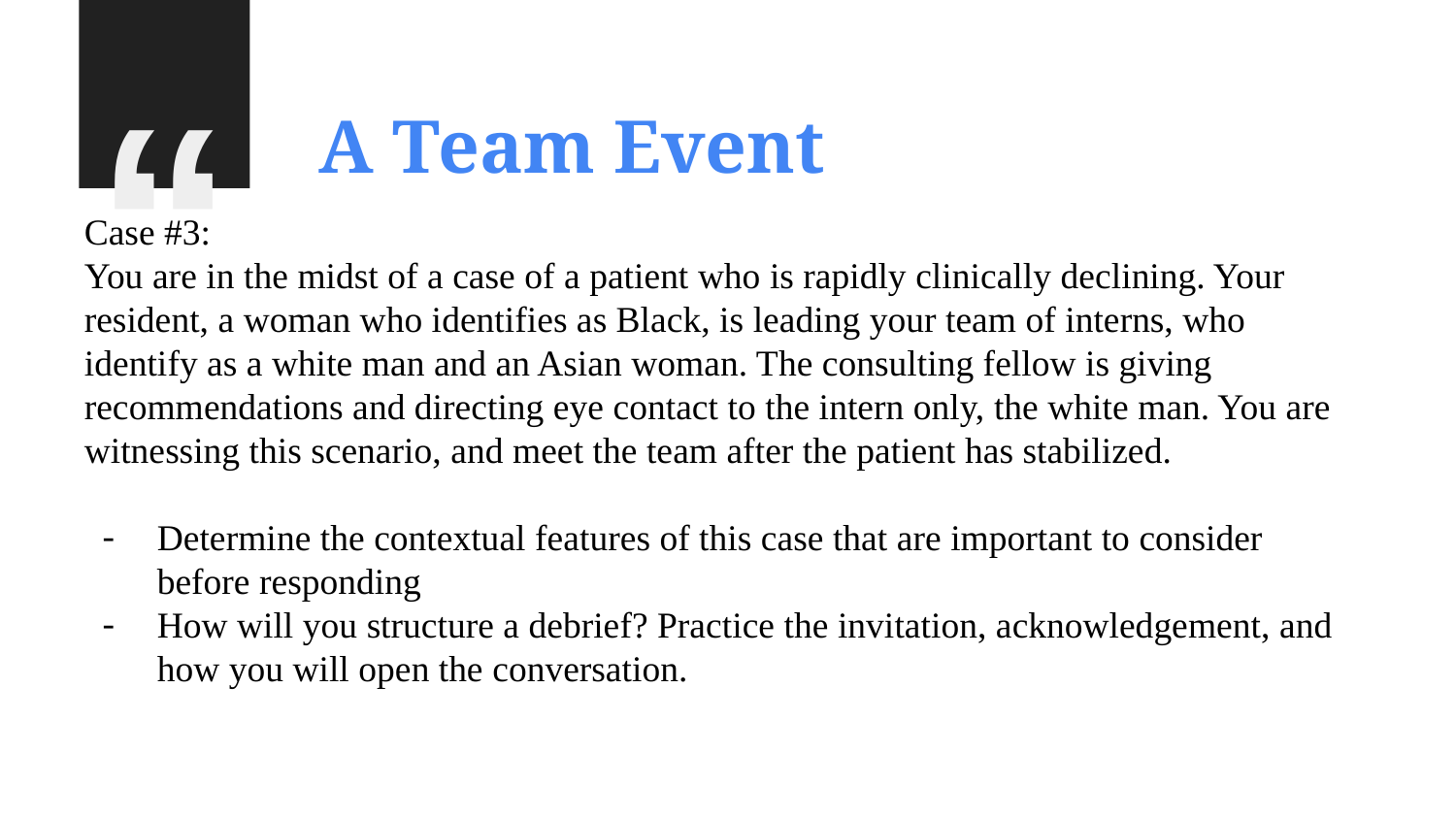

A Team Event
Case #3:
You are in the midst of a case of a patient who is rapidly clinically declining. Your resident, a woman who identifies as Black, is leading your team of interns, who identify as a white man and an Asian woman. The consulting fellow is giving recommendations and directing eye contact to the intern only, the white man. You are witnessing this scenario, and meet the team after the patient has stabilized.
Determine the contextual features of this case that are important to consider before responding
How will you structure a debrief? Practice the invitation, acknowledgement, and how you will open the conversation.

## Slide 35
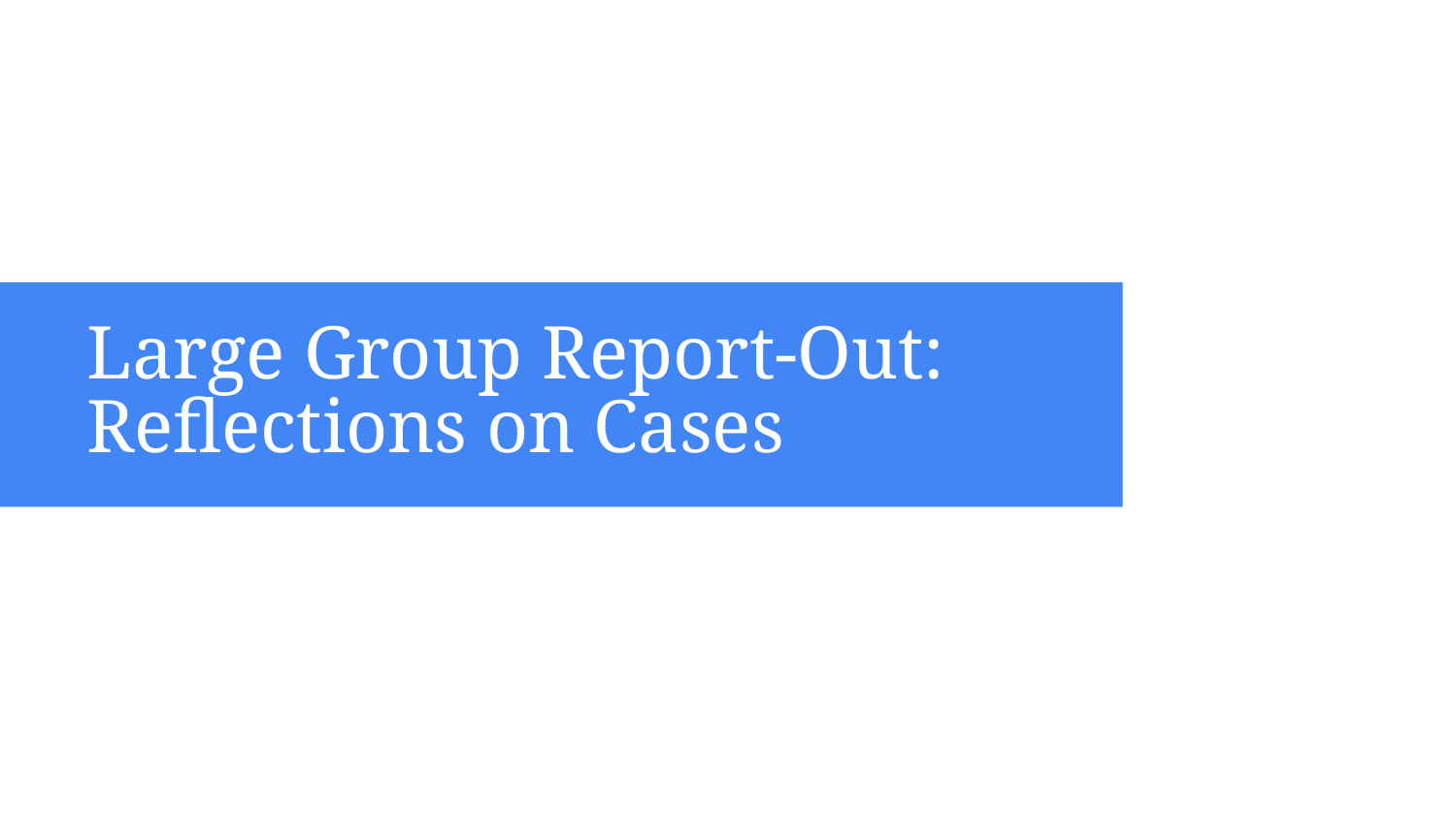

# Large Group Report-Out:
Reflections on Cases

## Slide 36
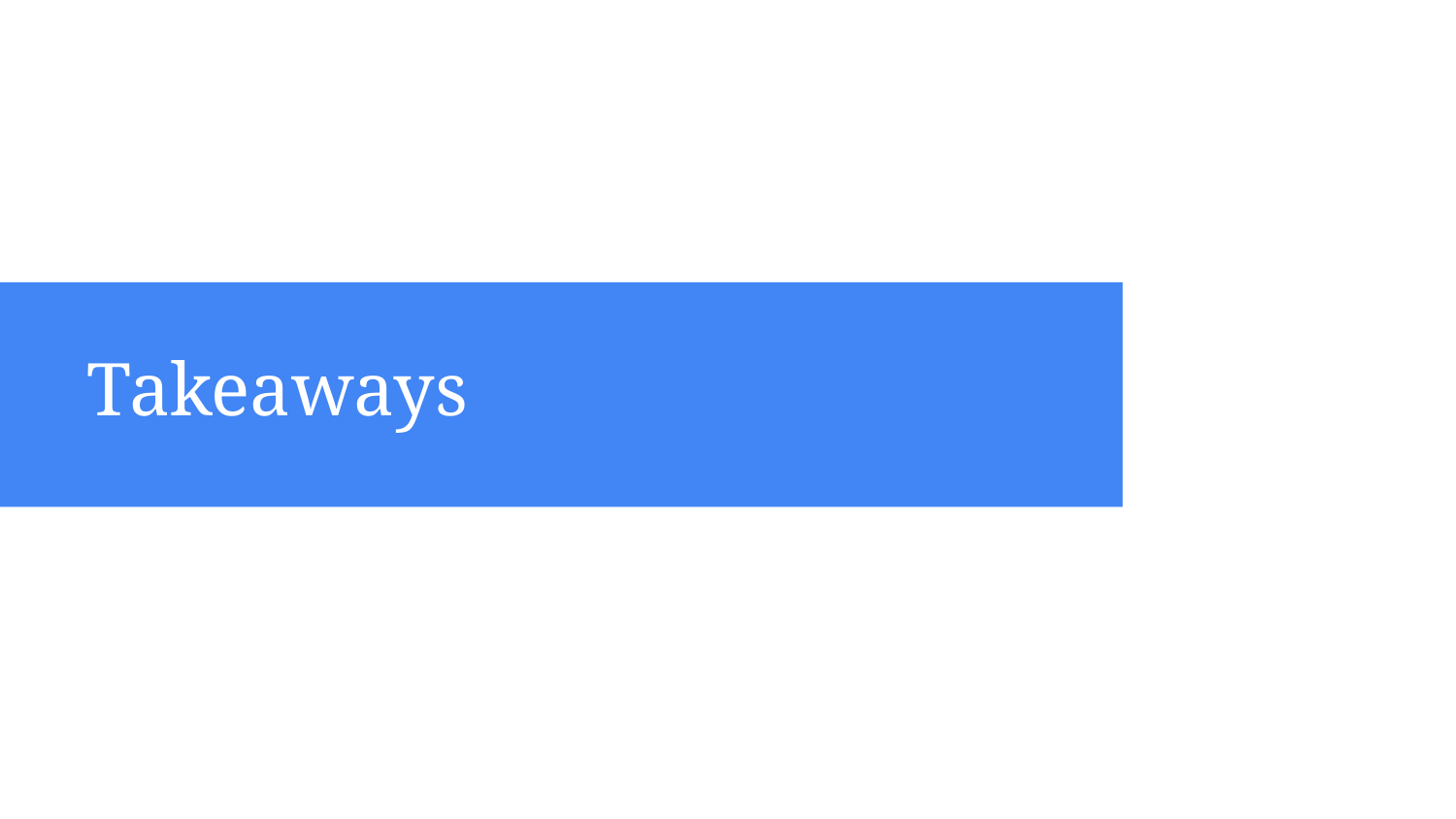

# Takeaways

## Slide 37
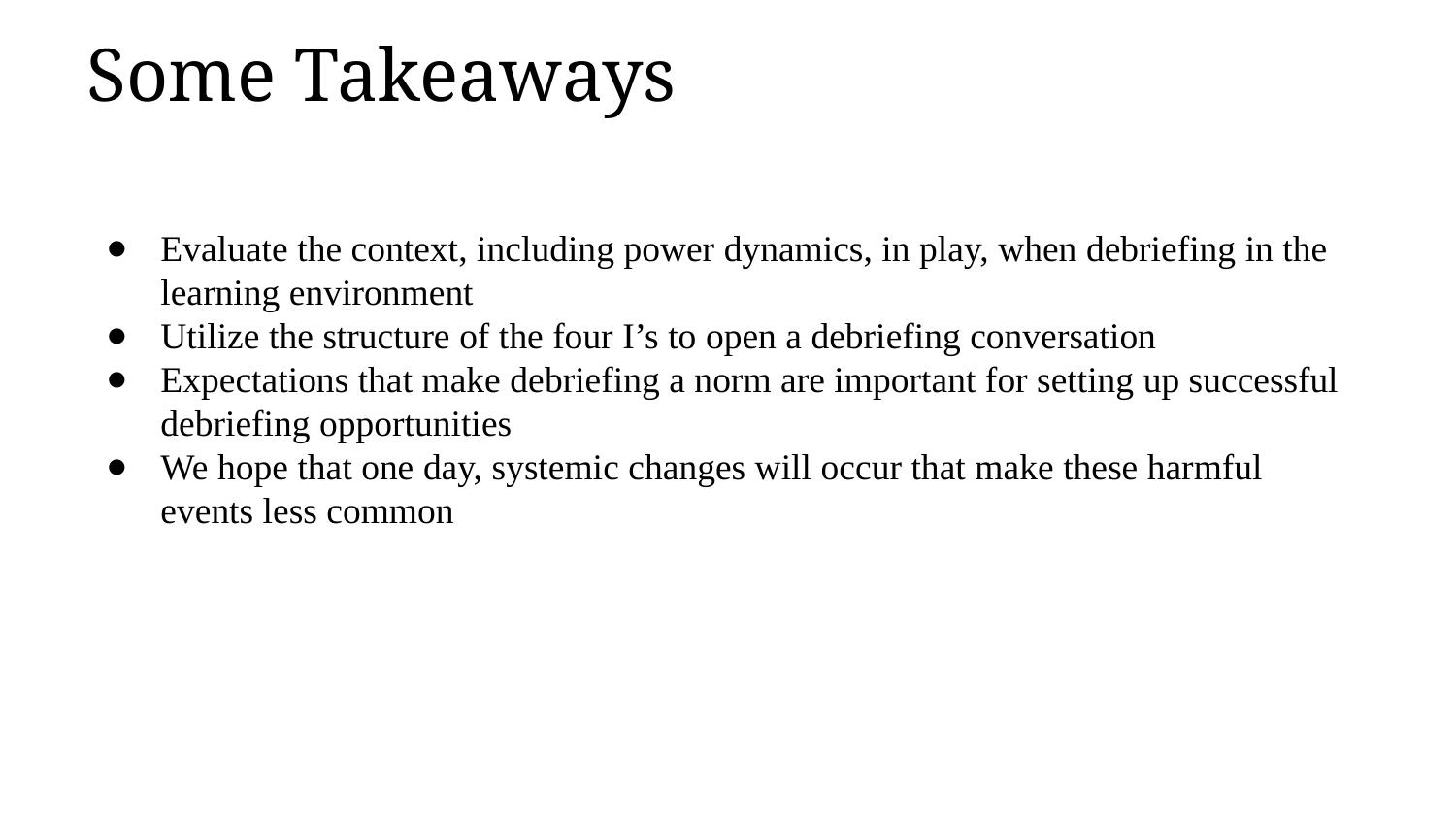

# Some Takeaways
Evaluate the context, including power dynamics, in play, when debriefing in the learning environment
Utilize the structure of the four I’s to open a debriefing conversation
Expectations that make debriefing a norm are important for setting up successful debriefing opportunities
We hope that one day, systemic changes will occur that make these harmful events less common

## Slide 38
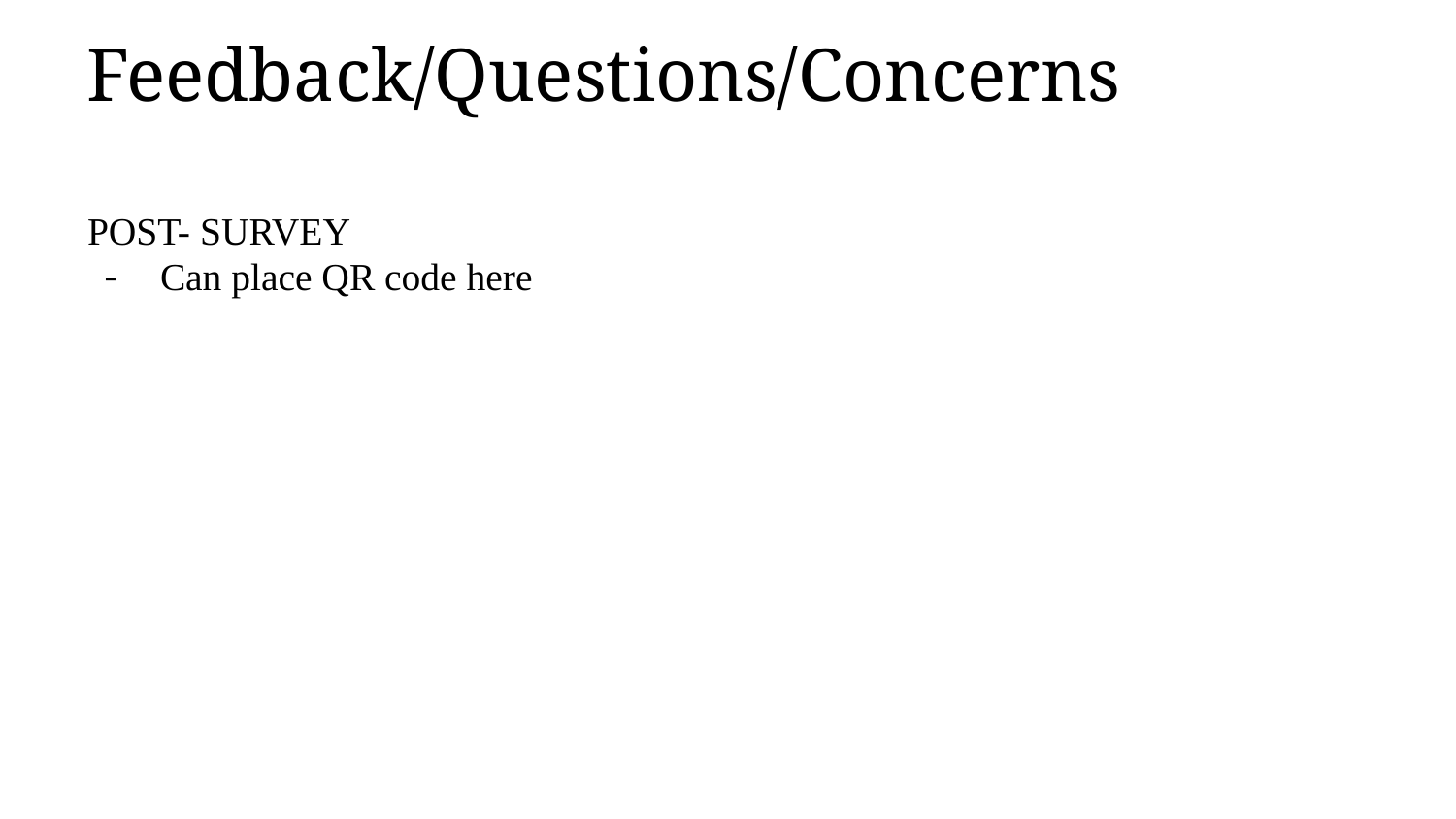

# Feedback/Questions/Concerns
POST- SURVEY
Can place QR code here
