# Supplementary material for: Acknowledging and Addressing Microaggressions: A Virtual Experiential Learning Approach for Faculty
Source: MedEdPORTAL. 2024 Sep 4;20:11436. doi: 10.15766/mep_2374-8265.11436 (PMC11374130; doi:10.15766/mep_2374-8265.11436)
Supplement: Supplementary file 1 — Sample Flier.pptxWorkshop 1 - Slides.pptxWorkshop 1 - Facilitator GuideWorkshop 1 - Participant Handout.docxWorkshop 1 - Pre- and Postsurvey.docxWorkshop 2 - Slides.pptxWorkshop 2 - Facilitator Guide.docxWorkshop 2 - Participant Handout.docxWorkshop 2 - Pre- and Postsurvey.docxWorkshop 3 - Slides.pptxWorkshop 3 - Facilitator Guide.docxWorkshop 3 - Participant Handout.docxWorkshop 3 - Pre- and Postsurvey.docxWorkshop 4 - Slides.pptxWorkshop 4 - Facilitator Guide.docxWorkshop 4 - Participant Handout.docxWorkshop 4 - Pre- and Postsurvey.docx [file mep_2374-8265.11436-s001.zip › O. Workshop 4 - Facilitator Guide.docx]

**Faculty Microaggressions Curriculum**

***Debriefing Bias and Microaggressions with Learners/Trainees/Teams***

Workshop #4

Dates:

Time: 3 hours

Where:

Lead Facilitator:

This is the fourth of a four-part series developed for faculty leaders in Graduate Medical education surrounding the topic of microaggressions. In order to meet learners where they are, earlier workshops will focus on learning and expanding introductory skills surrounding microaggressions, and will lead gradually to more advanced skills such as apologizing to learners when harm has been experienced, setting expectations in the learning environment, and debriefing microaggressions experienced by learners.

Today, we will focus on debriefing bias and microaggressions with learners, trainees, or teams. We will help develop a verbal script to utilize when debriefing what has occurred.

This workshop is particularly useful for those who work frequently with trainees or medical students.

**Learning Objectives:**

To increase confidence and comfort surrounding the following skills:

1. Developing an inclusive environment to debrief microaggressions and bias.
2. Facilitating a structured debrief with learners to process harm after witnessing or experiencing a microaggression or bias in the learning environment.

**Workshop Agenda:**

| **Time** | **Topic** | **Participants** |
| --- | --- | --- |
| 0:00 - 0:30 | Introductions/Group Agreements/Objectives | Large Group-Lead facilitator |
| 0:30 - 0:45 | Pair Share- Reflecting Together | Large Group |
| 0:45 - 1:30 | Context and Power Mapping | Large Group |
| 1:30- 1:45 | Structuring a Debrief | Large Group |
| 1:45 - 2:00 | Break |  |
| 2:00- 2:50 | Case Work with Feedback and Discussion | Small Group |
| 2:50 - 3:00 | Wrap-Up/Takeaways | Large Group |

Faculty lead for this workshop will begin the session with introduction of the workshop and community agreements. Participants will be invited to add additional agreements for the working session.

**Introductions/Community Agreements/Ice Breakers (30 minutes)**

Lead facilitator for this workshop will begin the session with introduction of the workshop and proposed community agreements. Participants voluntarily agree to this set of operational and behavioral agreements to build trust in this learning space and engage in productive work together. Participants will be invited to add additional agreements for the working session.

Community Agreements

•Respect each other as colleagues and humans

•Confidentiality

- Stories stay within our task force meetings, and lessons may leave the room. Especially when we are talking about specific cases related to our trainees and other divisional members/leaders

•Accountability

- We hold each other accountable for our words, actions, and impact
- We hold each other accountable for adhering to our group agreements

•Use “I” statements

- Our opinions and stories are our own, and we will not make blanket statements about others

•Impact versus intent

- We will recognize the difference between IMPACT and INTENT
- We will hold ourselves and others accountable by acknowledging IMPACT of words and actions when we see/feel/hear it

•Maximize the STRETCH ZONE!

- When we do this work, we are maximally efficient in the STRETCH ZONE, a brave space where we can (and should!) be uncomfortable, yet also be productive and learn
- When we label people and shame others, it puts people in the PANIC ZONE, and decreases buy-in to continue our mission

•Ask questions and invite other perspectives continually (i.e. humble inquiry)

- Replace the idea of perfection with a growth mindset, a lifelong journey of learning, effort, and persistence

•Prioritize self-care

- We understand that there may be stories, descriptions of events, and content that may be triggering or re-traumatizing to experience
- In a community of practice, we encourage care for our own mental health, in whatever sustainable form in which that takes shape
- We aim to be present in support of each other during challenging moments

Virtual Agreements can help create guidelines for operating in a virtual learning environment as well.

•Name/Pronouns/Role

- We invite participants to re-title their virtual presence with their name, pronunciation of name, pronouns (if comfortable sharing), and role as faculty

•Cameras on if possible

- To simulate in-person interactions, we invite participants to have cameras on throughout the educational workshop
- We understand that this is not possible based on physical location, background distractions, and internet access/bandwidth

•Mute when not speaking

- To encourage active listening and one speaker talking at a time, we promote the use of the mute button when not speaking
- This also makes closed-captions for accessibility more feasible

•Use the raise hand function to speak next

- This helps facilitate one speaker at a time and participants not speaking over each other, preventing miscommunication

•Feel free to use the chat and reactions

- For those who prefer written expression to verbal expression, this is an opportunity to engage and share perspectives

** It is important to try to include the learners in establishing these agreements. One tactic is collaborating together on coming up with specific categories of norms for conversations. Examples include brainstorming together norms for topics such as how to respect others and yourself, accepting challenge and discomfort, and keeping an open mind. It is also important that each participant agrees to the set of community agreements before moving forward with the work. This can be done virtually through a reaction, a chat agreement, or gestures in the virtual room.

Tools for further ideas:

<https://radcliffe-harvard-edu-prod.s3.amazonaws.com/8b8bef3c-2b23-4771-9847-625fc015adc4/LeveragingNormsforChallengingConversationsFINAL-ua.pdf>

<https://guidetoteaching.newschool.org/community-agreements/>

Following community agreements, each participant will have an opportunity to introduce themselves and the answer to an ice breaker:

*“ My name is ___________. I use ___________ pronouns. I work in the department of _______________________.*

*In my experience, the key to a successful debrief in the learning environment is ___________.”*

A brief review of working definitions is then presented (these have all been presented in the first workshop).

Working Definitions

- **Microaggression-** brief and commonplace daily verbal, behavioral or environmental indignities (whether intentional or unintentional) that communicate hostile, derogatory, or negative slights and insults against ​a particular group of people. It is important to note that the term micro refers to interactions between individuals, not the impact on the individuals, which can be immense and feel very “macro” to recipients of microaggressions. The term was coined by Dr. Chester Pierce, a Harvard psychiatrist who became the founding president of Black Psychiatrists of America, and we pay respect to this incredible physician who brought public attention to the everyday racism faced in America in the 1960s (Williams, 2019).
- **Intersectionality-** the interconnected nature of social categorizations such as race, class, and gender, regarded as creating overlapping and interdependent systems of discrimination or disadvantage​
- **Allyship -** a lifelong process of building relationships based on trust, consistency, and accountability with marginalized individuals and/or groups of people

**Pair Share- Reflection Questions (15 minutes)**

Start with separating the group into pairs for a reflection surrounding debriefing bias with learners.

*What makes debriefing bias or a microaggression challenging?*

*What are some unique approaches you have taken after experiencing a microaggression with a learner?*

In the large group reflection, facilitators can ask the group what is unique about debriefing bias. They can also ask what makes this process uncomfortable and how we can overcome this discomfort.

When teaching content related to diversity, equity, and inclusion (DEI), we commonly refer to the social ecological

model (Golden et al, 2020) of health, understanding that health outcomes and health disparities are a result of intrapersonal attitudes and interpersonal relationships, along with the institutions, systems, culture, and policies that surround us. Today we will focus on intrapersonal attitudes and then interpersonal relationships between faculty and learners.

**Intrapersonal: Context and Power Mapping Activity (45 minutes)**

Think about a situation where you witnessed or heard about a microaggression in the learning environment. In the concept of power mapping (see NEA resource in references), we identify key individuals or organizations that shape influence. Adapted to anti oppression learning by Drs. Wu and St-Hilaire (Wu et al., 2019), this concept can help us process the key individuals in a witnessed microaggression along with the possible dynamics that may have played a part in the outcome. Power mapping is a process of identifying the different types of privilege or marginalization that a person holds as an individual and their power in relation to others while being empathetic to the context of their current situation. Power mapping also draws attention to the intersections of identity and the nuances that come into play depending on the social context of the individuals interacting.

Participants will review an example from a facilitator. This is a teaching example, but facilitators can present this using their own personal examples. In this particular example, an intern (identifying as BIPOC) was the recipient of a microaggression, in which a family member made a racist comment and asked that the intern leave the room when a patient was undergoing an extubation in the ICU. The intern proceeded to tell her two senior residents and the attending physician in the intensive care unit. Without discussion, the intern was replaced by the white physician’s assistant. In this scenario, the event was never debriefed or acknowledged with the intern nor with the patient’s or their family member. The impact is clarified for the participants in a separate slide. As we map out this activity, we ask ourselves who the individuals were in the scenario, what relationships they have to each other, and how their individual power can be leveraged to create change.

- - Who were the others involved?
  - What dynamics were involved in the relationships between the individuals?
  - Which individuals hold the most power? In what way?
  - Who holds the least power? Why?
  - What is the risk with any in-the-moment intervention (professionally, personally)?

Participants will now be invited to make their own power map of a situation including a microaggression or instance of bias that they experienced.

- - Consider:
    - Who is the person with situational power who can conduct the debriefing?
    - Who are the individuals to debrief with?
    - How would you approach this?
    - If you acted in the moment, would you do something differently now?

In a large group reflection aft the individual power-mapping activity, participants are asked:

- Does mapping the experience help you understand the relationships between individuals?
- How did you empathize with some of the individuals involved?
- What would you have done differently?

**Structuring a Debrief (15 minutes)**

**Contextual Factors when Debriefing**

It is key to think about the social context before conducting a debrief

Who: *Use the power map to determine who to debrief with; understand your station in the learner’s support network*

When: *Ideally as time-sensitive as possible*

Where: *Ideally an in-person face-to-face conversation*

Why: *What actually is the why? Who is this for?*

**WHO**

When we think about support systems, it is also important to understand our social standing and station in the experience. Power mapping helps us to accomplish this. When debriefing with your learners, asking who you are to these individuals and who else they have in their support network can be useful to understand why they might not choose to turn to you for support. This support map examines who else learners may have in their support network. As we decentralize ourselves, we must not assume that we are the only source of support they have, nor are we always in their safe space.

- How do we acknowledge that?
- Why and when would they choose you?
- What kind of power dynamics are in place when debriefing with you?
- What do they get from you?

**WHEN**

Debriefing is like feedback, it must be done in a timely manner. Consider debriefing immediately after you notice that a microaggression has occurred. Ask learners about when they would want to debrief. Know that there are times when you may wait a little bit longer and that there isn’t always a correct answer, but sometimes even if it is late, addressing the bias or microaggression is better than not saying anything at all.

Open questions to the audience

- How do you know when to debrief?
- How do you decide?

**WHERE**

Determining where to debrief can lead to variable answers. In general, you may want a place of privacy that is away from the busyness of the clinical environment. Before debriefing as a group, you may consider some one-to-one interactions with individual learners. Avoid email or text debriefs as they are vulnerable to miscommunication and are less interactive than other venues. In-person is likely best, followed by virtual or by phone.

Open question to the audience

- How do you decide where?

**WHY**

The largest value of the debriefing process is understanding its impact. Our goal as faculty members is to support our learners, shed light on events that impact their learning, lives, and trajectory, and support sustainable care for themselves in the learning environments and their workplaces. We also want to create a culture of trust and respect so that learners will continue to feel a sense of belonging.


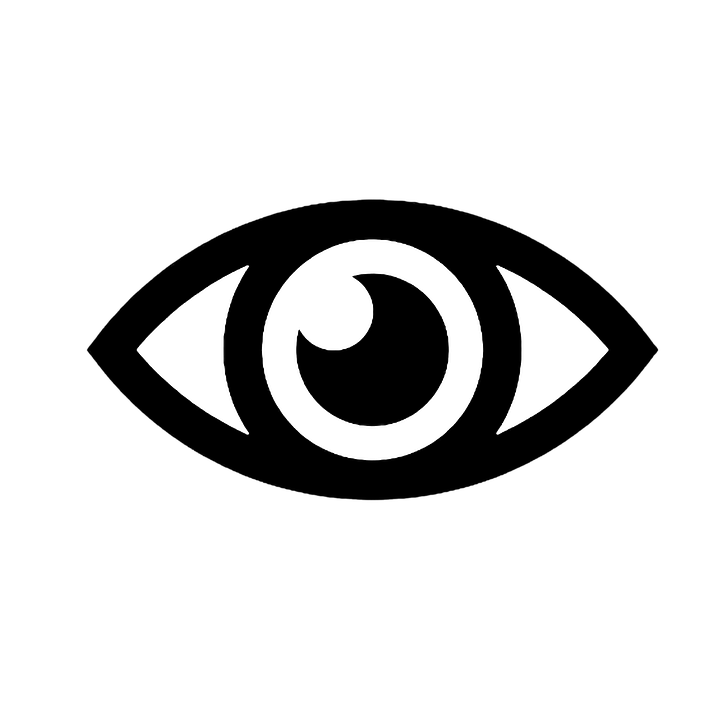


The Four I’s of Debriefing:

**Invitation**

We want to give learners agency over whether or not they want to debrief by inviting them to debrief first.

When you were present for the encounter:

*“I felt uncomfortable in that room just now. Would you like to process this together?”*

*“Is there anything that you’d like to talk about after what we just saw?”*

*“I’m here to listen to you.”*

When you hear about an experience from a learner:

*“I’m really sorry that you went through that experience. Would you like to debrief with me?”*

*“I appreciate you sharing with me. Would you like to tell me more?”*

**Impact**

In a trauma-informed manner, it is important to identify the impact without re-triggering.

We can practice naming impact without replaying it.

When you were present for the encounter:

*“Something about that incident didn’t sit right.”*

*“I wanted to acknowledge that in that interaction… I observed…”*

*“I feel like I just witnessed actions of _____, I want to acknowledge that.”*

*“I felt uncomfortable in that situation because I experienced _____, and I*

*want to acknowledge that.”*

When you hear about an experience from a learner:

*“I want to acknowledge the harm that has just occurred.”*

*“I can see how those words and actions were hurtful.”*

Making bold statements without having to reiterate the situation or the words can be helpful. This is more of a means to be trauma informed. Then you pause and hold space for more if the learner wants to talk more about impact.

**Insight**

People mistake the insight portion for wanting to tell the learner how they can help.

This portion is actually about asking how the learner wants to be supported in the moment.

*“What would be helpful to you right now?”*

*“How can I support you?”*

*“What do you need right now?”*

*“I want to prioritize you and your self-care right now. How can we do that?”*

**Information**

This is about how we share resources, summarize and commit to allyship

*“I am committed to creating a safer culture here. I will be _____, and is it okay if I check in with you to see*

*how you’re doing in ____?”*

*“As I think about the systemic ways we can make sure this never happens again, I want to make sure that you*

*are supported. Would you like some resources?”*

**Case Work with Feedback and Discussion (50 minutes)**

For each of these scenarios,

- Determine the contextual features of this case that are important to consider before responding as part of the debrief
- How will you structure a debrief? Practice the invitation, acknowledgement, and how you will open the conversation.

If desired, small groups can choose to review their power map activity or another experience and consider how to structure a debrief with learners with some of the skills from this workshop.

Scenario 1:

You are mentoring a student on a longitudinal project. While discussing this project, the student is talking about a recent encounter with a colleague, who is also an attending physician. The attending told the student, who has a learning disability, to “Get out of the OR and come back when you know more!” after she got a few anatomy questions wrong in the surgical OR. She is tearful in recounting this story to you.

Scenario 2:

You and your team are rounding together. Your first patient has an Oakland A’s jacket over his hospital gown. One of your trainees, who you know identifies as a lesbian, points at the jacket and says, “My kids love the A’s! We live near the stadium, and our nightly routine is they play baseball in the backyard to give us parents actual time to decompress from the day.” The patient responds cheerfully, “Oh cool! Is your husband in medicine too?”

Scenario 3:

You are in the midst of a case of a patient who is rapidly clinically declining. Your resident, a woman who identifies as Black, is leading your team of interns, who identify as a white man and an Asian woman. The consulting fellow is giving recommendations and directing eye contact to the intern only, the white man. You are witnessing this scenario, and meet the team after the patient has stabilized.

In large group reflection after these cases, facilitators can ask the participants the following questions:

- What did you get out of doing these activities today?
- Where do you think you might implement this?
- What do you foresee as still being challenges?

**Takeaways (10 minutes)**

After inviting takeaways from the participants, facilitators can offer some takeaways from the workshop:

- Evaluate the context, including power dynamics, in play, when debriefing in the learning environment
- Utilize the structure of the four I’s to open a debriefing conversation
- Expectations that make debriefing a norm are important for setting up successful debriefing opportunities
- We hope that one day, systemic changes will occur that make these harmful events less common

References for this workshop:

1. Polk, W., & El-Amin, A. (2016). *Leveraging Norms for Challenging Conversations*. <https://radcliffe-harvard-edu-prod.s3.amazonaws.com/8b8bef3c-2b23-4771-9847-625fc015adc4/LeveragingNormsforChallengingConversationsFINAL-ua.pdf>
2. The New School. (2020). *Community Agreements*. Guide to teaching and learning. <https://guidetoteaching.newschool.org/community-agreements/>
3. Williams, M. T. (2019). Microaggressions: Clarification, evidence, and impact. *Perspectives on Psychological Science*, *15*(1), 3–26. <https://doi.org/10.1177/1745691619827499>
4. Golden, T. L., & Wendel, M. L. (2020). Public health’s next step in advancing equity: Re-evaluating epistemological assumptions to move social determinants from theory to practice. *Frontiers in Public Health*, *8*. <https://doi.org/10.3389/fpubh.2020.00131>
5. National Education Association.. *Power Mapping 101*. NEA. <https://www.nea.org/professional-excellence/student-engagement/tools-tips/power-mapping-101>
6. Wu, D., Saint-Hilaire, L., Pineda, A., Hessler, D., Saba, G. W., Salazar, R., & Olayiwola, N. (2019). The efficacy of an antioppression curriculum for health professionals. *Family Medicine*, *51*(1), 22–30. <https://doi.org/10.22454/fammed.2018.227415>
