# Supplementary material for: Acknowledging and Addressing Microaggressions: A Virtual Experiential Learning Approach for Faculty
Source: MedEdPORTAL. 2024 Sep 4;20:11436. doi: 10.15766/mep_2374-8265.11436 (PMC11374130; doi:10.15766/mep_2374-8265.11436)
Supplement: Supplementary file 1 — Sample Flier.pptxWorkshop 1 - Slides.pptxWorkshop 1 - Facilitator GuideWorkshop 1 - Participant Handout.docxWorkshop 1 - Pre- and Postsurvey.docxWorkshop 2 - Slides.pptxWorkshop 2 - Facilitator Guide.docxWorkshop 2 - Participant Handout.docxWorkshop 2 - Pre- and Postsurvey.docxWorkshop 3 - Slides.pptxWorkshop 3 - Facilitator Guide.docxWorkshop 3 - Participant Handout.docxWorkshop 3 - Pre- and Postsurvey.docxWorkshop 4 - Slides.pptxWorkshop 4 - Facilitator Guide.docxWorkshop 4 - Participant Handout.docxWorkshop 4 - Pre- and Postsurvey.docx [file mep_2374-8265.11436-s001.zip › P. Workshop 4 - Participant Guide.docx]

**Faculty Microaggressions Curriculum**

**Debriefing Bias and Microaggressions with Learners/Trainees/Teams**

**Skills Handout**

**Learning Objectives**

To increase confidence and comfort surrounding the following skills:

1. Developing an inclusive environment to debrief microaggressions and bias.
2. Facilitating a structured debrief with learners to process harm after witnessing or experiencing a microaggression or bias in the learning environment.

Pair Reflection Questions

*What makes debriefing bias or a microaggression challenging?*

*What are some unique approaches you have taken after experiencing a microaggression with a learner?*

Power Map Activity

Think about a situation where you witnessed or heard about a microaggression in the learning environment. In the concept of power mapping (see NEA resource in references), we identify key individuals or organizations that shape influence. Adapted to anti oppression learning by Drs. Wu and St-Hilaire (Wu et al., 2019), this concept can help us process the key individuals in a witnessed microaggression along with the possible dynamics that may have played a part in the outcome. Power mapping is a process of identifying the different types of privilege or marginalization that a person holds as an individual and their power in relation to others while being empathetic to the context of their current situation. Power mapping also draws attention to the intersections of identity and the nuances that come into play depending on the social context of the individuals interacting. As we map out this activity, we ask ourselves who the individuals were in the scenario, what relationships they have to each other, and how their individual power can be leveraged to create change.

Think about a situation where you witnessed or heard about a microaggression in the learning environment

- - Who were the others involved?
  - What dynamics were involved in the relationships between the individuals?
  - Which individuals hold the most power? In what way?
  - Who holds the least power? Why?
  - What is the risk with any in-the-moment intervention (professionally, personally)?

Make your own power map of the situation

- - Consider:
    - Who is the person with situational power who can conduct the debriefing?
    - Who are the individuals to debrief with?
    - How would you approach this?
    - If you acted in the moment, would you do something differently now?

It is key to think about the social context before conducting a debrief

Who: *Use the power map to determine who to debrief with; understand your station in the learner’s support network*

When: *Ideally as time-sensitive as possible*

Where: *Ideally an in-person face-to-face conversation*

Why: *What actually is the why? Who is this for?*

The Four I’s of Debriefing:

**Invitation**

We want to give learners agency over whether or not they want to debrief by inviting them to debrief first.

When you were present for the encounter:

*“I felt uncomfortable in that room just now. Would you like to process this together?”*

*“Is there anything that you’d like to talk about after what we just saw?”*

*“I’m here to listen to you.”*

When you hear about an experience from a learner:

*“I’m really sorry that you went through that experience. Would you like to debrief with me?”*

*“I appreciate you sharing with me. Would you like to tell me more?”*

**Impact**

In a trauma-informed manner, it is important to identify the impact without re-triggering.

We can practice naming impact without replaying it.

When you were present for the encounter:

*“Something about that incident didn’t sit right.”*

*“I wanted to acknowledge that in that interaction… I observed…”*

*“I feel like I just witnessed actions of _____, I want to acknowledge that.”*

*“I felt uncomfortable in that situation because I experienced _____, and I*

*want to acknowledge that.”*

When you hear about an experience from a learner:

*“I want to acknowledge the harm that has just occurred.”*

*“I can see how those words and actions were hurtful.”*

Making bold statements without having to reiterate the situation or the words can be helpful. This is more of a means to be trauma informed. Then you pause and hold space for more if the learner wants to talk more about impact.

**Insight**

People mistake the insight portion for wanting to tell the learner how they can help.

This portion is actually about asking how the learner wants to be supported in the moment.

*“What would be helpful to you right now?”*

*“How can I support you?”*

*“What do you need right now?”*

*“I want to prioritize you and your self-care right now. How can we do that?”*

**Information**

This is about how we share resources, summarize and commit to allyship

*“I am committed to creating a safer culture here. I will be _____, and is it okay if I check in with you to see*

*how you’re doing in ____?”*

*“As I think about the systemic ways we can make sure this never happens again, I want to make sure that you*

*are supported. Would you like some resources?”*

Case Based Scenarios

For each of these scenarios,

- Determine the contextual features of this case that are important to consider before responding as part of the debrief
- How will you structure a debrief? Practice the invitation, acknowledgement, and how you will open the conversation.

Scenario 1:

You are mentoring a student on a longitudinal project. While discussing this project, the student is talking about a recent encounter with a colleague, who is also an attending physician. The attending told the student, who has a learning disability, to “Get out of the OR and come back when you know more!” after she got a few anatomy questions wrong in the surgical OR. She is tearful in recounting this story to you.

Scenario 2:

You and your team are rounding together. Your first patient has an Oakland A’s jacket over his hospital gown. One of your trainees, who you know identifies as a lesbian, points at the jacket and says, “My kids love the A’s! We live near the stadium, and our nightly routine is they play baseball in the backyard to give us parents actual time to decompress from the day.” The patient responds cheerfully, “Oh cool! Is your husband in medicine too?”

Scenario 3:

You are in the midst of a case of a patient who is rapidly clinically declining. Your resident, a woman who identifies as Black, is leading your team of interns, who identify as a white man and an Asian woman. The consulting fellow is giving recommendations and directing eye contact to the intern only, the white man. You are witnessing this scenario, and meet the team after the patient has stabilized.
