# Supplementary material for: Acknowledging and Addressing Microaggressions: A Virtual Experiential Learning Approach for Faculty
Source: MedEdPORTAL. 2024 Sep 4;20:11436. doi: 10.15766/mep_2374-8265.11436 (PMC11374130; doi:10.15766/mep_2374-8265.11436)
Supplement: Supplementary file 1 — Sample Flier.pptxWorkshop 1 - Slides.pptxWorkshop 1 - Facilitator GuideWorkshop 1 - Participant Handout.docxWorkshop 1 - Pre- and Postsurvey.docxWorkshop 2 - Slides.pptxWorkshop 2 - Facilitator Guide.docxWorkshop 2 - Participant Handout.docxWorkshop 2 - Pre- and Postsurvey.docxWorkshop 3 - Slides.pptxWorkshop 3 - Facilitator Guide.docxWorkshop 3 - Participant Handout.docxWorkshop 3 - Pre- and Postsurvey.docxWorkshop 4 - Slides.pptxWorkshop 4 - Facilitator Guide.docxWorkshop 4 - Participant Handout.docxWorkshop 4 - Pre- and Postsurvey.docx [file mep_2374-8265.11436-s001.zip › Q. Workshop 4 - Pre- and Postsurvey.docx]

**Microaggressions Workshop #4: *Debriefing Bias and Microaggressions with Learners/Trainees/Teams* PRE-SURVEY**

**Do you consent to using your responses as part of the research surrounding this work? ?**

_____ **YES**, you may use my responses in the research study.

_____ **NO**, you may NOT use my responses in the research study.

**What is your participant ID?** _______________ **(only asked if individual participates in study)**

2-digit birth**DAY** + last 2 letters of birth **CITY** + first initial of each **PARENT** in alphabetical order (use X if unknown)

E.g., Participant ID for a person born on July **09** in Tope**ka** whose parents are **K**yle and **S**am is **09KAKS**

**Demographic Information**

1. **My GME affiliated role is in the department of ____________**
2. **What is your race/ethnicity? (check all that apply)**

_____ American Indian or Alaska Native _____ White

_____ Asian _____ Multi-race/Ethnicity

_____ Black or African American _____ Unknown/Prefer not to say

_____ LatinX _____ Other/self-describe: _______

_____ Native Hawaiian or Other Pacific Islander

1. **What gender do you identify with?**

_____ Female _____ Other (please specify): _______

_____ Male ____ Prefer not to state

_____ Transgender female

_____ Transgender male

_____ Genderqueer/gender non-conforming

1. **Other identities that I hold related to my leadership/teaching role (free text):**
2. **Previous workshops I have participated in include: (Drop down boxes)**
3. **Acknowledging and Naming Microaggressions: Virtual 8/30**
4. **Acknowledging and Naming Microaggressions: Virtual 9/20**
5. **Apologizing when you have done harm: Virtual 10/11**
6. **Apologizing when you have done harm: Virtual 11/10**
7. **Setting Expectations for Learners/Trainees/Teams: 01/31**
8. **Setting Expectations for Learners/Trainees/Teams: 02/24**
9. **This is my first workshop**

**To what extent do you agree with these statements? (check one per row)**

|  | **Strongly Disagree** | **Disagree** | **Neutral** | **Agree** | **Strongly Agree** |
| --- | --- | --- | --- | --- | --- |
| It is important to debrief microaggressions when they have occurred in the learning environment with teams/trainees. |  |  |  |  |  |
| I consistently debrief microaggressions with learners in the clinical environment |  |  |  |  |  |
| **Effective debriefing skills** | **Strongly Disagree** | **Disagree** | **Neutral** | **Agree** | **Strongly Agree** |
| I am confident in my ability to organize a debrief when processing harm with learners. |  |  |  |  |  |
| I can identify power dynamics at play when debriefing a microaggression with learners. |  |  |  |  |  |
| I can identify when, where and with whom a debrief should occur. |  |  |  |  |  |
| I am confident in my ability to create an inclusive environment when debriefing bias and microaggressions in the learning environment. |  |  |  |  |  |

**My biggest barriers *debriefing bias and microaggressions with learners/trainees/teams* in the learning environment include:**

**Microaggressions Workshop #3: *Setting Expectations for Learners/Trainees/Teams* POST-SURVEY**

**To what extent do you agree with these statements? (check one per row)**

|  | **Strongly Disagree** | **Disagree** | **Neutral** | **Agree** | **Strongly Agree** |
| --- | --- | --- | --- | --- | --- |
| It is important to debrief microaggressions when they have occurred in the learning environment with teams/trainees. |  |  |  |  |  |
| I consistently debrief microaggressions with learners in the clinical environment |  |  |  |  |  |
| **Effective Debriefing Skils** | **Strongly Disagree** | **Disagree** | **Neutral** | **Agree** | **Strongly Agree** |
| I am confident in my ability to organize a debrief when processing harm with learners. |  |  |  |  |  |
| I can identify power dynamics at play when debriefing a microaggression with learners. |  |  |  |  |  |
| I can identify when, where and with whom a debrief should occur. |  |  |  |  |  |
| I am confident in my ability to create an inclusive environment when debriefing bias and microaggressions in the learning environment. |  |  |  |  |  |

1. What was the **most useful** part of this workshop? Why?
2. What was the **least useful** part of this workshop? Why?
3. What would you **change** about this workshop? Why?
4. Please feel free to offer feedback to your facilitators. Did they create an inclusive learning environment? What did they do well? What could they do better?
5. Commit to one personal change you will make to create a more inclusive learning environment after this workshop:
